# Single Cell/Nucleus Transcriptomics Comparison in Zebrafish and Humans Reveals Common and Distinct Molecular Responses to Alzheimer’s Disease

**DOI:** 10.3390/cells11111807

**Published:** 2022-05-31

**Authors:** Mehmet Ilyas Cosacak, Prabesh Bhattarai, Philip L. De Jager, Vilas Menon, Giuseppe Tosto, Caghan Kizil

**Affiliations:** 1 German Center for Neurodegenerative Diseases (DZNE) Dresden, Helmholtz Association, Tatzberg 41, 01307 Dresden, Germany; mehmet.cosacak@dzne.de (M.I.C.); pb2886@cumc.columbia.edu (P.B.); 2 Taub Institute for Research on Alzheimer’s Disease and the Aging Brain, Vagelos College of Physicians and Surgeons, Columbia University Irving Medical Center, Columbia University, 630 W 168th St., New York, NY 10032, USA; pld2115@cumc.columbia.edu (P.L.D.J.); vm2545@cumc.columbia.edu (V.M.); 3 Department of Neurology, Vagelos College of Physicians and Surgeons, Columbia University Irving Medical Center, Columbia University, 710 W 168th St., New York, NY 10032, USA; 4 Center for Translational and Computational Neuroimmunology, Department of Neurology, Columbia University Irving Medical Center, Columbia University, 630 W 168th St., New York, NY 10033, USA; 5 Gertrude H. Sergievsky Center, Vagelos College of Physicians and Surgeons, Columbia University Irving Medical Center, Columbia University, 710 W 168th St., New York, NY 10033, USA

**Keywords:** zebrafish, human, adult brain, fetal brain, telencephalon, Alzheimer’s disease, single cell RNA sequencing, single nuclear RNA sequencing, astroglia, microglia, neuroregeneration

## Abstract

Neurogenesis is significantly reduced in Alzheimer’s disease (AD) and is a potential therapeutic target. Contrary to humans, a zebrafish can regenerate its diseased brain, and thus is ideal for studying neurogenesis. To compare the AD-related molecular pathways between humans and zebrafish, we compared single cell or nuclear transcriptomic data from a zebrafish amyloid toxicity model and its controls (N = 12) with the datasets of two human adult brains (N = 10 and N = 48 (Microglia)), and one fetal brain (N = 10). Approximately 95.4% of the human and zebrafish cells co-clustered. Within each cell type, we identified differentially expressed genes (DEGs), enriched KEGG pathways, and gene ontology terms. We studied synergistic and non-synergistic DEGs to point at either common or uniquely altered mechanisms across species. Using the top DEGs, a high concordance in gene expression changes between species was observed in neuronal clusters. On the other hand, the molecular pathways affected by AD in zebrafish astroglia differed from humans in favor of the neurogenic pathways. The integration of zebrafish and human transcriptomes shows that the zebrafish can be used as a tool to study the cellular response to amyloid proteinopathies. Uniquely altered pathways in zebrafish could highlight the specific mechanisms underlying neurogenesis, which are absent in humans, and could serve as potential candidates for therapeutic developments.

## 1. Introduction

Alzheimer’s disease (AD) is the most common neurodegenerative disorder and the leading form of dementia in humans [1,2,3,4,5]. AD remains incurable. There is an unmet need for new scientific knowledge and out-of-the-box approaches that can help design novel therapeutic interventions. Not only a neuronal disease, AD also includes a complex interplay of multiple cell types, such as immune cells [2,6,7], the neurovascular niche [1,8], neural stem cells [9,10], astroglia [11,12], and oligodendrocytes [13,14], among others. The loss of neurons—which is relatively a late symptom of the disease—might be the consequence of the yet-elusive earlier pathological causes and disease-modifying mechanisms that remain underexplored. Identifying these causes early enough to revert them may help to design a robust intervention for mitigating or averting the effects of AD.

Recent findings have shown that neurogenesis is significantly reduced in patients with AD [15,16]. Blocking adult neurogenesis in an AD mouse model exacerbated neuronal loss and cognitive impairment, while inducing neurogenesis together with brain-derived neurotrophic-factor-improved cognition [17]. Impaired neurogenesis in early-stage AD and MCI (mild cognitive impairment) patients raises the possibility that stimulating the inherent neurogenesis potential in human brains could be a therapeutic target [16,18,19]. Thus, a plausible strategy for neurodegenerative diseases could be to induce endogenous cell types with stem or progenitor cell properties (such as certain subsets of glia) to generate new cells to replace lost neurons. However, this approach requires a better understanding of the factors that positively and negatively impact neurogenesis, such as the genetic variants that may alter the neural stem cell response and neurogenesis.

Animal models that successfully regenerate lost neurons present a new avenue to study neurogenesis in AD. Zebrafish are a promising option because, unlike mammals, they can successfully regenerate injured parts of their brains [20,21,22,23,24,25,26,27,28,29]. Thus, a better understanding of the parallels and differences between human cell types and zebrafish cells, with their neural regeneration capacity and established disease-related models [30,31], can provide insight into how neurons can regenerate and functionally integrate into the circuitry that has been dysregulated due to the disease.

The cerebroventricular microinjection of amyloid-beta-42 (Aβ-42), the presence of which is a hallmark of AD in humans, into an adult zebrafish brain [29,32] successfully recapitulates the pathological and cellular AD-like changes in humans, including the aggregation of amyloid peptides, increased inflammation, synaptic degeneration, and cell death. This is followed by cognitive decline and memory deficits, which are common AD symptoms [33,34]. However, in contrast to humans [15,16], zebrafish neural stem cells enhance their proliferation and generate new neurons [9,29] in the presence of these pathologies. The neurons survive and integrate into the existing circuitry, suggesting that the zebrafish can be used as a useful experimental model to investigate neuronal regeneration. The mechanisms identified in zebrafish could be used for clinical applications in human brains, as exemplified in recent studies that employed zebrafish as a comparative functional genomics tool for human AD [35,36]. Similarly, the genes associated with AD in humans can be tested for their functionality in the adult zebrafish brain. Here, we aimed to uncover the shared mechanisms between our zebrafish model and human AD cohorts to prioritize genes and pathways underlying AD in both species, as well as the genes and pathways unique to zebrafish. This animal model could highlight the neuroregenerative features that are absent in human brains. Here, we developed an analytical pipeline for comparing molecular transcriptomics datasets in zebrafish and humans, leveraging publicly available and in-house single cell/nucleus RNA-sequencing data. 

## 2. Results

**Figure 1:**
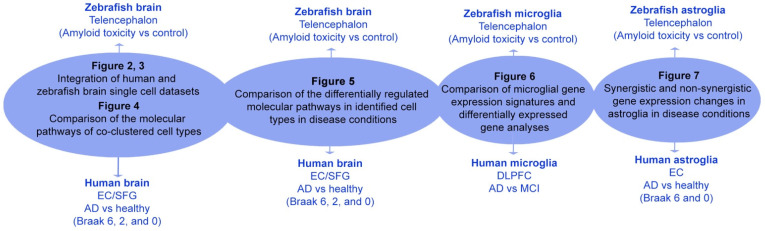
Overall study scheme. Summary of the single cell dataset comparison, conclusions drawn, and respective figures.

In this study, we compared different single cell datasets from human and zebrafish brains in Alzheimer’s disease and toxicity conditions. The main comparisons, conclusions drawn from the comparisons, corresponding figures, demographics, and a tabular summary are shown in Figure 1 and Table 1. 

### 2.1. Integrated Clustering of Zebrafish and Human Cells

**Figure 2:**
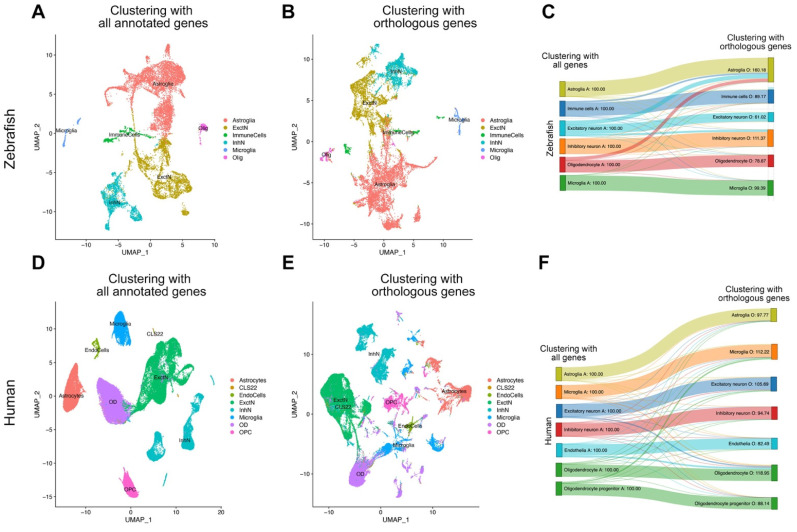
Transition analysis between cell clusters. (**A**) tSNE plot showing the main cell types in zebrafish when all genes annotated in zebrafish are used for clustering. (**B**) tSNE plot showing the main cell types in zebrafish when only the genes orthologous to humans are used for clustering. (**C**) Transition diagram between (**A**,**B**). When human orthologous genes are used, majority of the cell types remain in their clusters, with slight exception of a subset of oligodendrocytes, excitatory neurons, and inhibitory neurons that start clustering in astroglia. (**D**) tSNE plot showing the main cell types in humans when all genes annotated in humans are used for clustering. (**E**) tSNE plot showing the main cell types in humans when only the genes orthologous to zebrafish are used for clustering. (**F**) Transition diagram between (**D**,**E**). When zebrafish orthologous genes are used, the vast majority of the cell types remain in their clusters.

A cross-species genomics comparison is affected by the level of evolutionary conservation of orthologous genes. Zebrafish genes contain orthologs to more than 70% of the human genes [43], yet humans and zebrafish contains different genes. Therefore, using the entire set of genes in an organism for single cell clustering could give different results than only using the orthologous genes of the species with which the comparison is being made. To determine if our integration approach using orthologous genes would alter the clustering fidelity, we performed a transition analysis for clustering results in both conditions (entire set of genes per organisms versus orthologous genes only) (Figure 2). We found that in clustering zebrafish and human cells using all the annotated genes vs. clustering using orthologous genes only, the vast majority of the cells remain in the same cluster identity (e.g., astroglia remain in astroglia cluster) (89.2% in humans, 86.6% in zebrafish) (Figure 2C,F). This proves the reliability of the data integration and the subsequent clustering approach. In zebrafish, the major change was the transition from excitatory neurons to astrocytes when human orthologous genes were used (26.7% of excitatory neurons, 7.1% of all cells; Figure 2C). Additionally, a portion of the zebrafish cells in the excitatory neuron cluster also transited into inhibitory neuron clusters when human orthologous genes were used (13.0% of excitatory neurons, 3.4% of all cells; Figure 2C). This major transition could point towards a set of biological mechanisms in zebrafish that specify early neuronal progenitors in astroglia stages through specific programs or genes that do not have orthologs in humans. Similarly, in humans, when zebrafish orthologous genes were used, there was a mutual transition between oligodendrocytes and excitatory neurons (5.91% of oligodendrocytes started to cluster in excitatory neurons, 9.36% of excitatory neurons started clustering in oligodendrocytes; Figure 2F). This could suggest the presence of multipotent progenitors for excitatory neurons and oligodendrocytes that might be delineated by different molecular programs in humans and zebrafish, given that such progenitors were shown before [44,45,46]. Our comparative integration pathway and transition analyses showed that the majority of the cell types can be reliably identified by using orthologues genes common to humans and zebrafish. Minor transitions could delineate the evolutionary divergence in the different transitory stages of individual cell types, as well as the molecular pathways or genes that pertain to those physiological identities.

**Figure 3:**
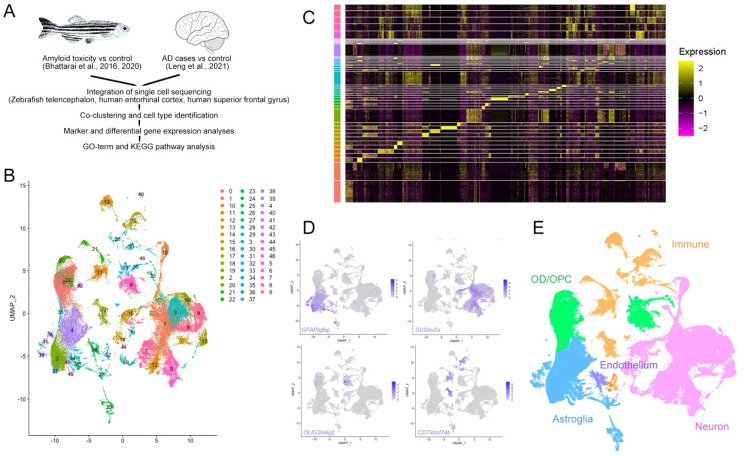
Integration of single cell transcriptomics data from zebrafish and human brains in AD. (A) Schematic work pipeline for integration of open-access datasets from [29,37,40,41,42,43,44,45,46,47,48,49]. (B) tSNE plot that co-localizes and clusters human and zebrafish cells. (C) Heat map of the marker genes of the identified clusters. (D) Exemplary gene expression for cell clusters: *GFAP* for astroglia, *SV2* for neurons, *OLIG2* for oligodendrocytes, and *CD74* for immune cells. (E) Colored cell type identification tSNE for human and zebrafish composite single cell clustering. See Figures S1 and S2 and Data S1.

**Figure 4:**
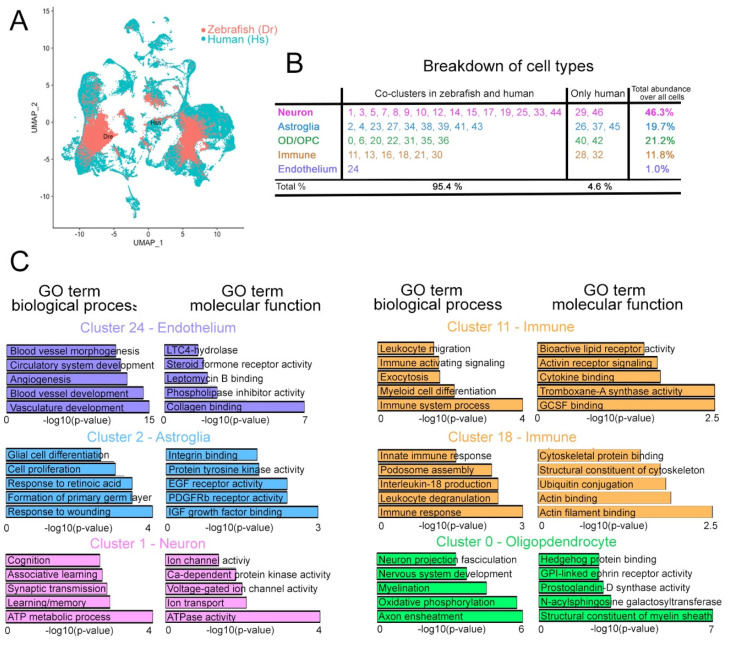
Analyses of the composite human–zebrafish single cell clusters. (**A**) Color-coded breakdown of the cells on the composite tSNE plot. Green: human cells; red: zebrafish cells. (**B**) Table showing the cluster numbers, identities, their co-clustering status in human and zebrafish, and the abundance of cells in those clusters. 95.4% of all cells on the composite tSNE plot can be co-clustered in humans and zebrafish. 4.6% of all cells are only in human clusters. (**C**) GO term enrichment graphs for the representative endothelial, neuronal, astroglial, immune, and neuronal clusters. See Figures S3 and S4, Datas S2 and S3.

Our cross-species integration analyses based on orthologous genes revealed 47 distinct cell clusters (Figure 3A,B and Figure S1) with distinct marker gene expression patterns (Figure 3C and Figure S2). We identified four major cell groups by using marker genes (*GFAP* for astroglia, *SV2* for neurons, *OLIG2* for oligodendrocyte progenitors and oligodendrocytes, and *CD74* for immune cells; Figure 3D,E Supplementary Data S1). Overall, more than 95% of all cells from human brains and zebrafish telencephalon were successfully grouped into clusters containing cells from both species. We focused on these composite clusters for our downstream analyses (Figure 4A and Figure S3). We found that 15 neuronal clusters (45.4% of all cells), 9 astroglial clusters (18.1% of all cells), 7 OPC/OD clusters (20.7% of all cells), 6 immune clusters (10.2% of all cells), and the endothelial cluster (1.0% of all cells) included cells from both human and zebrafish (Figure 4B). In contrast, we found two neuronal, three astroglial, two OPC/OD, and two immune cell clusters that contained only human cells; together, these nine clusters comprised only 4.6% of the total cells (Figure 4B). 

Next, we determined the molecular functions and biological processes associated with the genes expressed in these six composite clusters (Figure 4C). The biological processes in cluster 1 (neurons) include learning, memory, synaptic transmission, learning, and cognition, and the molecular functions include ion transport, voltage-gated ion channel activity, and calcium-dependent kinase activity (Supplementary Data S3), which are among the classical neuronal physiological processes [47,48,49]. The biological processes enriched in cluster 2 (astroglia) are consistent with the diverse roles of glial cells including differentiation, response to injuries, and cell proliferation. Insulin growth factor signaling, epidermal growth factor signaling, integrin binding, and tyrosine kinase activity are among the molecular functions enriched in this astroglial cluster (Supplementary Data S3), and they are processes known to regulate astroglial activity in vertebrates [11,30,50,51]. For the immune cell clusters 11 and 18, zebrafish and human clusters are enriched in immune-system-related processes such as immune response, leukocyte activity, and proinflammatory cytokine signaling (Figure 4C, Supplementary Data S3). Molecular functions in immune clusters are also characteristic and include cytokine signaling, migratory behavior, cytoskeletal dynamics, and GCSF responsiveness (Figure 4C) [52,53]. Cluster 24 (endothelia) is enriched for processes including vasculature development and circulation-related biological processes, consistent with general endothelial functions (Figure 4C, Supplementary Data S3). The marker genes for this cluster are enriched for functions that include collagen binding, steroid hormone activity, and the leukotriene signaling pathway, which are important characteristics of endothelia [54,55]. Finally, the OPC/OD cluster-0-enriched marker genes are involved in nerve fasciculation, myelination, and axon ensheathment, as well as relevant molecular functions such as GPI-linked ephrin signaling, prostaglandin synthesis, sphingosine signaling, and myelination (Figure 4C, Supplementary Data S3) [13,46,56]. These findings demonstrate that zebrafish and human cells can be reliably integrated using the methodology we established. 

### 2.2. Differential Expression Analyses between AD and Controls in Zebrafish vs. Human 

**Figure 5:**
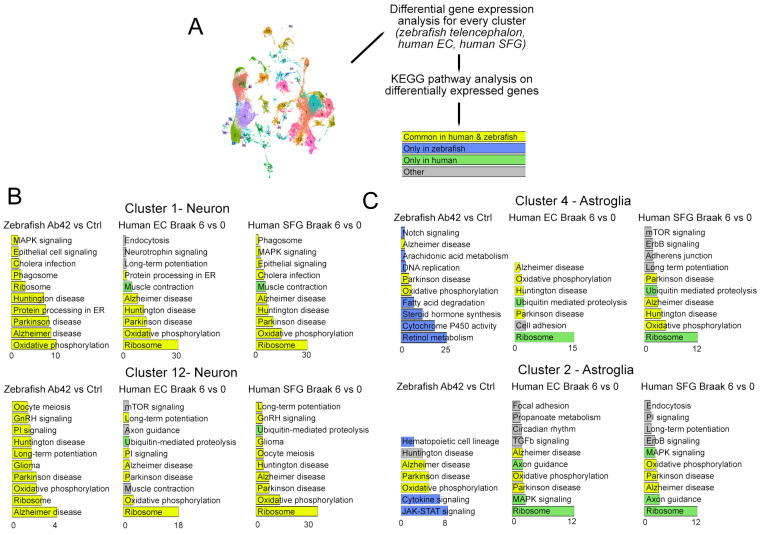
Analysis of the differentially expressed genes in specific cell clusters. (**A**) Schematic representation of the analysis pipeline. Human cell clusters from EC and SFG are compared between Braak Stage 6 and 0, and zebrafish cell clusters were compared between amyloid-beta-42 injection versus controls. The common KEGG pathways for differentially expressed genes are shown in yellow, zebrafish-specific hits are blue, and human-specific hits are in green. The other category includes the hits that are present only in one human brain region, but not in the other. (**B**) Neuronal and astroglial cell clusters are compared for the KEGG pathway changes. Strikingly, the neuronal clusters in human and zebrafish respond to AD in a highly similar fashion in terms of altered KEGG pathways (**B**), while astroglia have more species-specific responses than common (**C**). See Datas S4 and S5.

We then investigated how the molecular response of the adult human and zebrafish cell clusters compare to each other in AD. For this, we compared the identified cell clusters from the zebrafish telencephalon, human EC (entorhinal cortex), and human SFG (superior frontal gyrus) separately, and determined the differentially expressed genes (DEGs) between the disease and control conditions (Figure 5, Supplementary Data S4). For instance, cluster 1 (neurons) had 801 differentially expressed genes in zebrafish and 1823 genes in human EC (Supplementary Data S4). Out of these, 198 genes were common across species and 117 showed the same directionality (i.e., “synergistic DEG”). Among the synergistic and non-synergistic DEG genes, we identified few AD-known loci (i.e., genes prioritized by previous large GWAS and sequencing studies for AD). For instance, *MEF2C*, a protective factor against neurodegeneration [57], is among the synergistically upregulated DEGs in neurons in both organisms (human: logFC = 0.335; *p* = 0.0067; zebrafish: logFC: 0.297, *p* = 0.0421). On the other hand, *RBFOX1*—an RNA-binding protein found as top signal that is a recent GWAS for amyloid load in AD and involved in amyloid clearance [58]—is a non-synergistic DEG. In humans, the expression of *RBFOX1* is reduced, while in zebrafish neurons, it is upregulated, which might imply a more efficient protein clearance response in zebrafish compared to humans. 

To determine the molecular pathways affected in zebrafish and humans after AD, we performed a KEGG pathway analysis of the DEGs in humans (control vs. AD, EC and SFG) and zebrafish (amyloid toxicity vs. control) (Figure 5A, Supplementary Data S5), and categorized the statistically significant pathways according to their presence in both humans and zebrafish (yellow), only in zebrafish (blue), and only in humans (green) (Figure 5B). We observed that AD pathways are consistently enriched in the neuronal clusters 1 and 12 in zebrafish and humans. Similarly, we found that the majority of the KEGG pathway terms for DEGs in zebrafish neuronal clusters 1 and 12 are also present in the human brain (Figure 5B). These included ribosomes, phagosome, protein processing in the endoplasmic reticulum, oxidative phosphorylation, and long-term potentiation, which are all implicated in AD [59,60,61,62]. However, when we compared the astroglial cluster (cluster 4), the synergistic KEGG pathway representation in the neuronal clusters changed dramatically. In astroglia, we observed more species-specific pathways (Figure 4B). The common pathways affected in zebrafish and human astroglia include oxidative phosphorylation and AD. Zebrafish showed changes in pathways such as JAK-STAT signaling, cytokine-signaling retinol metabolism, steroid signaling, fatty acid degradation, DNA replication, arachidonic acid metabolism, and Notch signaling, while humans showed ribosome, axon guidance, and proteolysis-related terms (Figure 5B, Supplementary Data S5). 

### 2.3. Comparison of Microglia between Zebrafish and Humans

**Figure 6:**
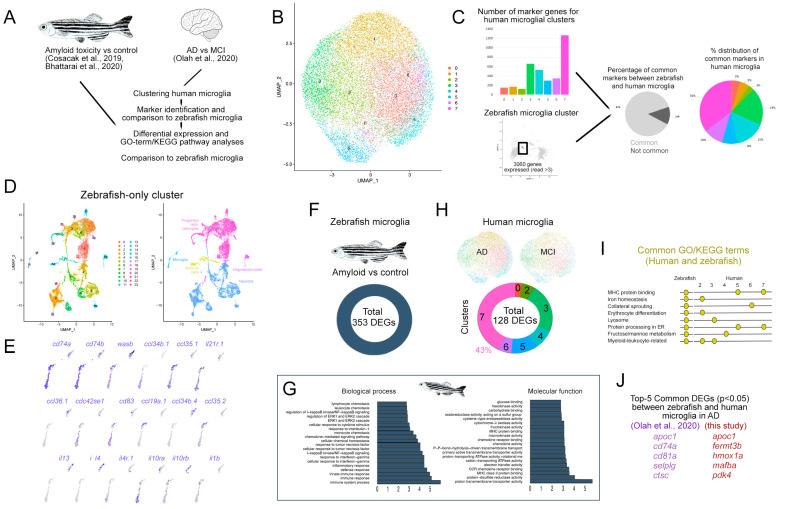
Comparison of human and zebrafish microglia. (**A**) Schematic representation of the analysis pipeline. Open access datasets from [38,40,41] (**B**) Clustering of the human microglia single cell sequencing data. (**C**) Comparison of the number of marker genes in microglial clusters, pie charts for the percentage of common marker genes of zebrafish microglia and human microglia, and the distribution of the common genes to individual human microglial clusters. (**D**) Clustering of zebrafish single cell sequencing dataset, both alone and color-coded tSNE plots for cell types. (**E**) Zebrafish immune cell clusters and representative gene expressions. (**F**) Differentially expressed gene analyses in zebrafish microglia and human microglia in AD. (**G**) Representative graphs of the biological process and molecular functions of the differentially expressed genes in zebrafish microglia in the AD model. (**H**) Clustering of human microglia dataset [38] from Alzheimer’s disease versus Mild cognitive impairment patients and differentially expressed gene numbers. (**I**) Representative GO terms and KEGG pathways that are common in human and zebrafish microglia. (**J**) Comparison of the top five common differentially expressed genes in zebrafish [40,41] and human microglia [38] in AD. See Datas S6–S11.

We clustered the live microglia single cell sequencing from human AD patients (Figure 6A) [38] and identified eight microglial clusters (Figure 6B, cluster numbers do not relate to previous figures). A marker gene analysis showed that 87% of the human microglial markers (3579 genes identified by Seurat analyses, Supplementary Data S6) are common to zebrafish microglia [40,41] (Supplementary Data S7, Figure 6C), while 35% of the common markers are in human microglial cluster 7 (Figure 6C). When clustered separately, zebrafish single cell sequencing identified major cell types—neurons, astroglia, oligodendrocytes, microglia, and other immune cells—falling in multiple clusters (Figure 6D). The microglial cell population in zebrafish expresses various cytokines and receptors that are associated with microglial physiology (Figure 6E), suggesting a functional parallelism in human and zebrafish microglia. 

When we compared the amyloid-injected brains to the controls in zebrafish, we identified 353 DEGs in the microglial cell population (Figure 6F, Supplementary Data S8). A GO term analysis of the DEGs in zebrafish microglia upon AD found that a diverse range of the immune-system-related biological processes are enriched (Figure 6F, Supplementary Data S9). The molecular functions of the DEGs in zebrafish included energy metabolism, MHC protein binding, and chemokine signaling (Figure 6G). To determine the DEGs in human microglia in AD versus the MCI stage, we compared the identified microglial cell clusters and found 128 DEGs in total (Figure 6H, Supplementary Data S10). Of the human microglia, 43% of the DEGs were found in cluster 7 (Figure 6D, Supplementary Data S11). A comparison of the GO term and KEGG pathway analysis in human and zebrafish microglia showed common processes and pathways such as MHC protein binding, iron homeostasis, lysosomal processes, energy metabolism, and leukocyte-related processes (Figure 6I), indicating that the microglial responses to AD in zebrafish and humans are parallel for particular molecular pathways and genes (Figure 6J). 

### 2.4. Comparison of Astroglial Clusters in Human and Zebrafish Brain in AD

**Figure 7:**
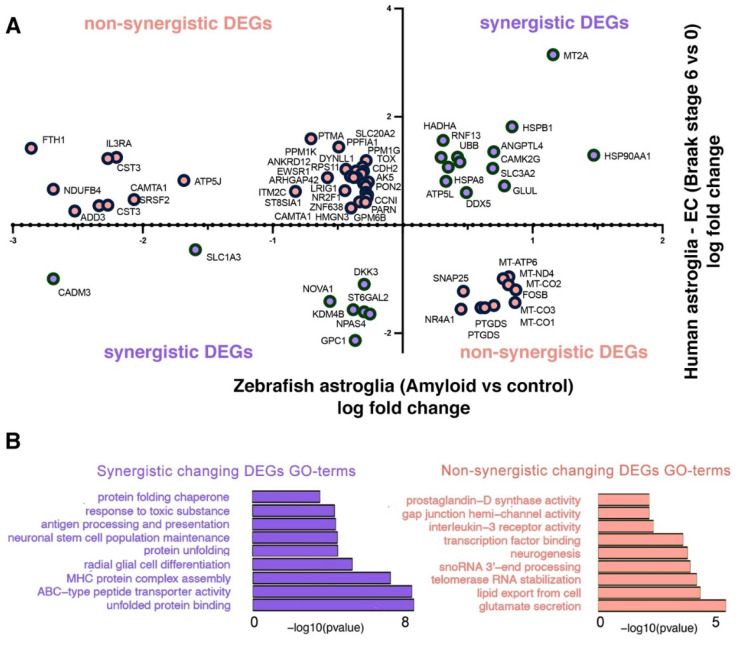
Comparison of differentially expressed genes in the human and zebrafish astroglia clusters. (**A**) Chart indicating the genes that are differentially expressed in astroglial clusters 2 and 4 in zebrafish (telencephalon, amyloid toxicity versus control) and human brains (entorhinal cortex, Braak stage 6 versus 0) when these clusters are compared within. The x-axis shows the log-fold changes for zebrafish astroglia and the y-axis denotes the log-fold changes in human astroglia. Duplicate gene names indicate their appearance in both astroglial clusters. The gene names are distributed sterically on the graph. (**B**) Selected GO terms for synergistically and non-synergistically differentially expressed genes in all astroglial clusters, combined. See Datas S4, S5 and S12.

The astroglial response to AD could have a profound association with the neurogenic outcome. Therefore, we investigated the differentially expressed genes (DEGs) in astrocyte clusters in zebrafish (amyloid vs. control) and human (entorhinal cortex, Braak stages 6 vs. 0) (cluster 2 and cluster 4 in Figure 1, Figure 2 and Figure 3) to determine the common DEGs. We found 64 genes that were common in the DEG lists of human and zebrafish astroglia: 21 genes showed a synergistic differential expression pattern in both organisms, while 43 genes were non-synergistically changed (Figure 7A, Supplementary Data S12). The synergistic DEGs yielded in GO term enrichment were related to protein quality control, neural stem cell activity, immunity-related pathways, and toxicity response (Figure 7B). The non-synergistic DEGs showed enrichment for pathways related to neurotransmitter release, RNA processing, neurogenesis, and immune-related pathways such as interleukin signaling (Figure 7B). 

### 2.5. Developing Human Brain versus Zebrafish

Neurogenesis in humans is reduced with aging [15,18,63,64,65,66], and this reduction could be due to the reduction in the neurogenic programs of the astroglial cells. If so, developing human brains and zebrafish brains should have astroglia co-clusters that would bear neurogenic markers. To test whether developing human brains could have astroglial clusters that resemble a more neurogenic state than adult human brains, we used a human brain single cell study from gestation week 18 of the fetal human hippocampus [39] (Figure S4). Here, we identified 25 cell clusters that contained 4 major cell types including neurons, astroglia, immune cells, and oligodendrocytes (Figure S4). Additionally, when a human fetal brain was compared to an adult zebrafish brain, we found a neural progenitor subcluster within the human astroglial cells (cluster 8, Figure S4) which expressed the progenitor marker *TOP2* and proliferation marker *MKI67* [67]. This cluster is not present in an adult human entorhinal cortex dataset [37] (Figure S5), despite the presence of the hippocampal neural stem/progenitor cells (the presence of *GFAP*/*SOX2*/*NES*/*ASCL1*-positive cells, Figure S5), and it indicates the gradual loss of neurogenic ability in human brains with aging. Furthermore, this clustering also suggests that the zebrafish brain might reflect the neurogenic potential of the embryonic human brain. This is particularly interesting because neurogenesis diminishes with both advancing age and AD in humans [9,16,18,19,68]. 

## 3. Discussion

We compared zebrafish and human brain gene expression at a single cell resolution and identified synergistic and non-synergistic DEGs and pathways. The former points at a common cellular response to AD pathology that can be utilized to investigate disease-associated cellular mechanisms. On the contrary, the non-synergistic DEGs and pathways highlight the different responses between zebrafish and human brains to AD pathology, such as pathways induced or suppressed by zebrafish that are required for successful neuroregeneration upon AD. Since human brains cannot elicit neural regeneration after AD, further investigation of non-synergistically differentially expressed genes and pathways could shed more light into the mechanisms uniquely activated by zebrafish, ultimately highlighting potential candidates for inducing neurogenic response in human brains. 

Our cross-species single cell transcriptomics comparison highlighted the pathways that are uniquely altered in zebrafish. Astroglial proliferation and neurogenic ability are affected by fatty acid degradation [69], and in zebrafish, the constitutively neurogenic glial cells have an active fatty acid metabolism [70,71]. Retinoic acid is related to the neuronal differentiation capacity of neural stem cells [72], and in zebrafish, retinoic acid signaling is associated with neurogenic outcome [73,74]. Notch signaling is an important determinant of neurogenesis in vertebrate brains [31] and is related to glial cell proliferation [30,75]. Arachidonic acid and its derivatives are among the key regulators of the immune system [76]. Arachidonic acid derivatives and other immune regulators are regulators of the neurogenic outcome and neuroregeneration in zebrafish [25,26,29,40,77]. These pathways are important regulators of neurogenesis and neural regeneration in the zebrafish AD model and can elicit neural regeneration in mammalian neural stem cells in a context-dependent manner [19,20,21,29,30,40,41,78,79,80]. Previous findings that the neural regeneration is prevalent in the zebrafish brain after AD, but not in human brains [16,18,19,29,40,77,81,82], suggest that the zebrafish could act as a clinically relevant animal model to understand how vertebrate brains could elicit neuro-regeneration in AD. 

Ubiquitin-mediated proteolysis and axon guidance were pathways uniquely altered in the human brains within the astroglial clusters (Figure 4C). The defects in proteolysis and the inability of axons to re-grow and establish new connections are pathological hallmarks of AD [4,83,84,85,86,87,88]. Endothelial cells are critical regulators of the neurovascular unit, together with the astroglia [1,87], and we found that these two cell types may have specific reactions to AD between zebrafish and humans, whereas neurons show similar responses (Figure 5B). Ultimately, we hypothesize that the zebrafish AD model might manifest a neuropathological response in neurons similar to that of human brains, while the response in other cell types (such as astroglia and neurovascular unit) have their own peculiarities. This could be one of the underlying reasons for the differential neuroregenerative capacities between humans and zebrafish. A plausible hypothesis we are pursuing is to learn how zebrafish can generate new neurons upon AD and maintain brain homeostasis [19,30,41,77,89,90]. 

In our comparison of single cell astroglial clusters (Figure 7, Supplementary Data S12), many neurogenesis-related genes were enriched. For instance, the diseased astrocytes in humans and zebrafish synergistically reduced *SLC1A3*, which is an amino acid transporter for glutamate uptake, contributing to the ion sink mechanism of astroglia and marking a transitory state to neurogenic lineage [91]. Similarly, the heat shock proteins *HSPB1*, *HSPA8*, and *HSP90AA1*, which belong to a family of proteins that regulate neurogenic outcome [92], are also upregulated in both organisms. *NPAS4*, a neuroprotective protein [93], is synergistically downregulated in human and zebrafish astroglia, potentially indicating a reacting state to the amyloid toxicity. Similarly, *UBB*, which is involved in abnormal toxic protein removal and protein quality control [94], is upregulated in both organisms. These results suggest that AD pathology initiates a protein clearance mechanism in both humans and zebrafish astrocytes. 

On the other hand, astroglia in both organisms displayed non-synergistic gene expression changes in several genes related to neurogenesis. Among the top differentially expressed genes (Figure 7A), we found that *PTGDS*, a mediator of the anti-inflammatory effects of astroglia [95], is significantly upregulated in zebrafish (logFC = 0.602, *p* = 0.0311) but downregulated in humans (logFC = −1.527, *p* = 0.0012). Since inflammation reduces neurogenic ability and increases gliogenic outcome [96], differential *PTGDS* function could contribute to the neurogenic outcome. Similarly, *FOSB*, which is required for adult neurogenesis in rodents [97], is downregulated in human astroglia in AD (logFC = −1.199, *p* = 0.0027) but upregulated in zebrafish (logFC = 0.871, *p* = 1.17 × 10^−8^). We found other genes, such as *ADD3*, which negatively affects the neurogenic progenitor fate [98]; *CST3*, the upregulation of which compromises the survival of neurons [99]; and *EWSR1*, a gene involved in the regulation of neural differentiation [100], are upregulated in humans (logFC= 0.259, *p* = 1.55 × 10^−16^; logFC = 0.251, *p* = 0.0001; and logFC = 0.770, *p* = 0.0397, respectively) and downregulated in zebrafish astroglia (logFC = −2.524, *p* = 0.0073; logFC = −2.203, *p* = 5.34 × 10^−75^; and logFC = 0.300, *p* = 0.0009, respectively). The GO term analyses of synergistically and non-synergistically expressed genes also verified these findings, as synergistic genes enriched pathways related to toxic protein response and glial cell differentiation pathways, while non-synergistic genes enriched the processes related to neurogenesis, neurotransmitter release, or RNA processing (Figure 7B). *LRIG1* was recently identified in an AD GWAS of east Asian ancestry [101], and the gene encodes a transmembrane protein that controls the extent of the epidermal growth factor signaling by suppressing the EGF receptor (EGFR) availability [102]. EGF signaling is important for astroglial activation and priming for neurogenesis [50], and therefore upregulation of *LRIG1* in human AD (logFC = 0.882, *p* = 0.0087) and downregulation in zebrafish (logFC = −0.353, *p* = 4.1 × 10^−6^) can point towards a differential neurogenesis response in humans and zebrafish. This hypothesis is supported by a study where bulk RNA sequencing was performed in the human entorhinal cortex by comparing symptomatic AD patients with individuals that bore the pathological hallmarks of AD, but not the clinical manifestation of dementia [103]. Here, *LRIG1* was found to be significantly upregulated in symptomatic AD vs. non-symptomatic AD patients, suggesting that neurogenic outcome in these individuals could offset the clinical manifestation of dementia. Therefore, our pipeline for cross-species DEG analyses can give unprecedented insights into the functional validation of AD GWAS/TWAS datasets for neurogenesis-related aspects. Our findings suggest that zebrafish can turn on genetic programs that lead to neurogenesis after AD-related pathology, while humans cannot. Our comparative genomic analyses could help to understand which molecular programs differ between regenerative and non-regenerative vertebrate brains, whether there are critical genes that can act as roadblocks to neuroregenerative ability in humans, and whether this understanding could lead to a therapeutic intervention for enhancing the resilience of human brains in AD. 

Besides its strengths, our study has limitations. One limitation we observed is the power of the sequencing. Although zebrafish and human cells can be integrated on a tSNE plot, the clusters where we found common marker genes (Supplementary Data S1) correspond to 62.3% of all cells. Therefore, increasing the depth of sequencing will populate the identified clusters with more cells and will help determine more marker genes in all clusters. Despite this limitation, we identified common processes that are altered upon AD in both organisms. Additionally, the publicly available and in-house single cell datasets from zebrafish are limited in number. This reduces the power for a more comprehensive comparison between zebrafish and human. The genomic annotations for humans and zebrafish in the databases are continuously updated, and every release version adds or removes certain annotations. A raw dataset annotated by using a particular genome release may not contain all the gene identifiers in another dataset that uses an older release. Therefore, the number of orthologous genes between species varies. The number of orthologs we used in this study was 14,133 out of approximately 61,000 gene identifiers from human and 16,908 out of approximately 35,000 gene identifiers from zebrafish. We determined that integration and clustering zebrafish and human single cell datasets by using all genes in these species or only orthologous genes does not affect the clustering of the main cell types into their respective cell clusters (Figure 2). Further analyses, using machine learning and non-overlapping marker genes to identify the same cell types between humans and zebrafish, may overcome the effects of variable orthologous gene identification across platforms and datasets.

## 4. Methods

### 4.1. Single Cell Transcriptomics Data 

We used single cell transcriptome data from zebrafish telencephalon and the entorhinal cortex or superior frontal gyrus of human brains and human fetus datasets. Five datasets were downloaded from the Gene Expression Omnibus repository and used in the current study: human brain datasets GSE147528 [37] and microglia datasets [38], and the zebrafish datasets GSE118577 [41], GSE124162 [40], GSE161834 [42], and GSE186874. See Table 1. 

### 4.2. Single Cell Data Analyses Using All Genes

The raw datasets for human superior frontal gyrus (SFG) and entorhinal cortex (EC) samples were downloaded from the Gene Expression Omnibus repository under the following GEO ID: GSE147528 [37]. The cells were filtered out by using DropletUtils, using 10,000 iterations and an FDR of <0.01. The cells that were used in [37] were chosen, and cells with less 200 total counts (or nCount_RNA) were removed from the analyses. Additionally, genes expressed in less than five cells were removed from the analyses. In total, 2472 were removed as they did not pass the above thresholds. The remaining cells from all samples were used for further analyses using Seurat V3.1.5 [104]. Each dataset was converted to a Seurat object, normalized, and the top 2000 variable genes were identified. The data were scaled using all genes, the nCount_RNA mitochondrial genes percentages were regressed out, and, finally, 30 PCAs (RunPCA) were identified. To integrate the datasets, the top 2000 variable genes from each dataset were used. After finding anchors (FindIntegrationAnchors), the datasets were integrated (IntegrateData). The data were scaled to 10,000 and the nCount_RNA mitochondrial genes percentages were regressed out. Then, the top 30 PCAs were calculated, and the clusters were identified using a resolution of 0.5. In total, 26 clusters (numbered from 0 to 25) were identified. We used the same settings above to perform clustering for the zebrafish datasets.

### 4.3. Main Cell Types

Following cell clustering, heat maps were generated. Cell types were inferred based on the characteristic gene expression patterns. For human datasets, the main cell types were identified by using the following markers (based on [37]): *GFAP*, *SLC1A2*, and *AQP4* (astroglia), and *MBP* and *MOG* (oligodendrocytes or OD), *PDGFRA* and *SOX10* (oligondendrocyte precursors or OPC), *CD74* and *CX3CR1* (microglia), *SLC17A7* and *CAMK2A* (excitatory neurons, or ExctN), *GAD1* and *GAD2* (inhibitory neurons or InhN), and *CLND5* and *FLT1* (endothelial cells or EndoCells). Cluster 22 was named as *CLS22*. For the zebrafish datasets, we used the markers based on [41]: *fabp7a* and *her4.1* (astroglia); *sv2a* (neurons); *aplnra*/b (oligodendrocytes); *gad1* and *gad2* (inhibitory neurons); *neurod1*, *neurod2*, and *nell2b* (excitatory neurons); *lck1* and *cd74a*/*b* (microglia); and *wasb* and *lyve1b* (immune cells).

### 4.4. Outcomes

For the single cell transcriptomics (scRNA) sequencing data, we operated two types of comparisons: brains in Braak = 6 vs. Braak < = 2 to identify AD cases and non-AD controls. In subsequent, and more conservative, secondary analyses, we restricted the samples to Braak = 6 vs. Braak = 0. In zebrafish, we compared amyloid-toxicity-induced Alzheimer’s disease to the control. 

### 4.5. Orthologous Genes

The orthologous genes between human and zebrafish were retrieved from https://www.ensembl.org/index.html (accessed on 1 March 2022). In total, 14,133 (out of 14,825 including genes with 0 counts) genes from humans and 16,908 (out of 17,373 including genes with 0 counts) genes from zebrafish had one-to-one or one-to-many orthologous genes. We created artificial gene names combining human and zebrafish orthologue genes, which was a total of 20,993 genes. Then, a new matrix from the human and zebrafish datasets was generated using the orthologous genes. The new matrix contained duplicated genes because of the one-to-many orthologues. 

### 4.6. Single Cell Data Analyses

#### 4.6.1. Preprocessing of the EC/SFG Datasets

The raw datasets were downloaded from GEO under the following accession number: GSE147528. The h5 files were converted to matrix/genes/features files using sp_sparse/sparse from the scipy Python3 library. The cells were filtered out by using DropletUtils and using 10,000 iterations and an FDR of <0.01. We selected cells that had been used in [37] and further removed cells with less than 200 transcripts. The primary human [105] datasets were downloaded from https://organoidreportcard.cells.ucsc.edu (accessed on 7 October 2021). 

#### 4.6.2. Integrating all Cells from the Zebrafish Telencephalon, Human EC/SFG, and Human Fetal Samples

After generating a new matrix based on the orthologue genes, each dataset from each sample was converted to a Seurat object (Seurat V4.0.5), the data were normalized (*Seurat*::*NormalizeData*), and the top 2000 variable genes were identified (*Seurat*::*FindVariableFeatures*). The data were scaled to 10,000 and the nCount_RNA was regressed out (*Seurat*::*ScaleData*). The top 30 PCAs were used for dimensional reduction and identifying the clusters with a resolution = 1. Then, the 2000 anchors were used to integrate all Seurat objects created above: (i) by finding the integration anchors (*Seurat*::*FindIntegrationAnchors*), (ii) by integrating the objects (*Seurat*::*IntegrateData*), (iii) using all.genes to scale the data and regress out the nCount_RNA, and (iv) calculating the top 30 PCAs and using them for dimensional reduction and identifying cell clusters by using a resolution of 0.5 and 1. We used the same options above to integrate: (i) EC/SFG datasets with zebrafish datasets, (ii) fetal hippocampal datasets with zebrafish datasets, and (iii) microglia datasets from EC/SFG [37] and DLPFC [38] with zebrafish microglia datasets. For the latter, we only used top 500 variable genes/integration anchors and the top 20 PCAs. 

#### 4.6.3. Marker Genes Analyses

We first identified the marker genes using the “*Seurat*::*FindAllMarkers*” function with the option only.pos = T. Then, we generated heatmaps/dotplots from the top 20 marker genes for each cell cluster. To identify the main cell types between the EC/SFG and zebrafish cells: (i) we used the marker genes used by [37], i.e., *GFAP* and *AQP4* for astrocytes, *MBP*/*MOB* for oligodendrocytes, *PDGFRA* for oligodendrocytes progenitor cells, *CLDN5* for endothelial cells, *GAD1*/*GAD2* for inhibitory neurons, and *SLC7A7*/*CAMK2A* for excitatory neurons; and (ii) we used the previously identified marker genes in [41] for zebrafish cell types, *fabp7a* for progenitor cells (PC), *sv2a* for neuronal cells (NN), *aplnra*/*b* for OPC/OD, and *cd74a*/*b* for immune cells. We also use the markers from zebrafish for human cells and the markers from human to zebrafish.

#### 4.6.4. The Effect of Orthologous Genes on Each Dataset

To verify if using orthologues had a dramatic effect on the main cell types and clustering in comparison to using all annotated genes in humans and zebrafish, we used the data matrices generated from the 20,993 artificial genes created from orthologues as described above. We used the same options/pipelines that were used to integrate the human and zebrafish datasets using Seurat (as explained above). 

#### 4.6.5. Differentially Expressed Genes and GO Term Analyses

To identify the differentially expressed genes, we used the *Seurat*::*FindMarkers* function and compared every sample to its corresponding control for every cluster (e.g., the AD Braak Stage 6 cluster 0 to the control patients (Braak Stage 0 or Braak Stage 2) for cluster 0.) We performed GO and KEGG pathway analyses using GOstats as described previously [41]. 

#### 4.6.6. Comparing Human Microglia and Zebrafish Microglia

To compare zebrafish and human microglia, we analyzed each dataset separately. In brief, a Seurat object was generated for each dataset, the data were normalized with NormalizeData, and 500 variable genes were identified. The data were scaled and the nCount_RNA was regressed out. The first 20 PCAs were determined, clusters were identified using a resolution of 1, and the UMAP was calculated for 2D visualization. To integrate the datasets, we used the above Seurat objects. For integration, 500 anchor features and 20 PCAs were used to identify the anchors. Data scaling, cluster identification, and UMAP detection were performed as above. To identify the DEGs in the microglia dataset, we compared the AD cases with the controls for every cluster. Enrichment analyses was performed by using GOstats.

## Figures and Tables

**Table 1:** Demographics of the datasets used in this study.

	Human	Human	Human	Zebrafish
**Region**	Adult entorhinal cortex (EC) and superior frontal gyrus (SFG)	Adult dorsolateral prefrontal cortex (DLPFC)	Embryonic telencephalon and cortex	Adult telencephalon (pallium and subpalium)
**Sequencing type**	Single nuclear RNA sequencing—mix	Single nuclear RNA sequencing—microglia	Single cell RNA sequencing—mix	Single cell RNA sequencing—mix
**Number of individuals**	10	48	1st trimester—10	12
**Number of cells**	41,578 (EC), 62,086 (SFG)	16,172 microglia nuclei	6665	15,447
**Comparison**	Alzheimer’s disease (Braak stage 2 and 6) vs. control (Braak stage 0)	Alzheimer’s disease (Braak stage 2 and 6) vs. control (Braak stage 0)	Developmental stages	Amyloid-injected versus control
**References**	[37]	[38]	[39]	[40,41,42]

## Supplementary Materials

The following supporting information can be downloaded at: https://www.mdpi.com/article/10.3390/cells11111807/s1, Figure S1: tSNE plot for integrated zebrafish and human single cell transcriptomics; Figure S2: heat map for identified clusters after integrating zebrafish and human single cell transcriptomics; Figure S3: tSNE plot for integrated zebrafish and human single cell transcriptomics showing zebrafish and human cells in color codes; Figure S4: Integration of single cell transcriptomics data from human fetal hippocampus and adult zebrafish brain; Figure S5: tSNE plots for the genes related to the hippocampal neurogenic glial populations; Data S1: Individual tSNE plots for genes used to define cell types in integrated zebrafish and human single cell transcriptomics; Data S2: Marker genes identified for human and zebrafish clusters; Data S3: GO-term analyses of the common marker genes identified for human and zebrafish co-clusters; Data S4: Differentially expressed genes in identified human and zebrafish co-clusters; Data S5: GO-term analyses on differentially expressed genes in human and zebrafish co-clusters; Data S6: Immune cell markers for identified human microglial clusters; Data S7: Genes expressed in zebrafish immune cells; Data S8: Differentially expressed genes in zebrafish microglial after amyloid toxicity compared to controls; Data S9: GO-term analyses on differentially expressed genes in zebrafish microglial after amyloid toxicity; Data S10: Differentially expressed genes in human microglial in AD compared to controls; Data S11: GO-term analyses on differentially expressed genes in human microglial after AD; Data S12: Common differentially expressed genes in astroglia of zebrafish brain and human entorhinal cortex.
