# Supplementary material for: Single Cell/Nucleus Transcriptomics Comparison in Zebrafish and Humans Reveals Common and Distinct Molecular Responses to Alzheimer’s Disease
Source: Cells. 2022 May 31;11(11):1807. doi: 10.3390/cells11111807 (PMC9180693; doi:10.3390/cells11111807)
Supplement: Supplementary file 1 [file cells-11-01807-s001.zip › Supplementary_Data/DataS1.pdf]

ENSG00000019582qqCD74qqENSDARG00000009087qqcd74a

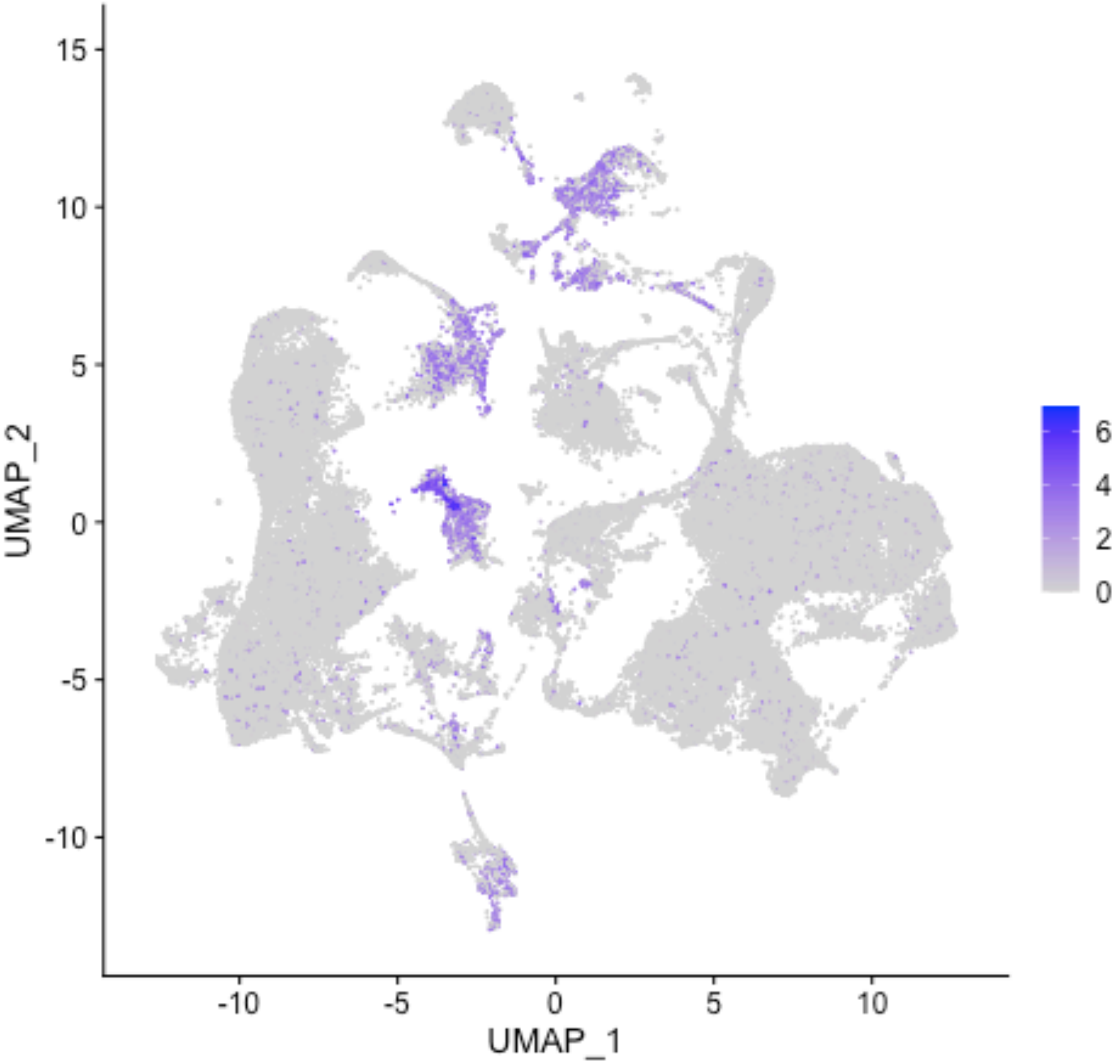

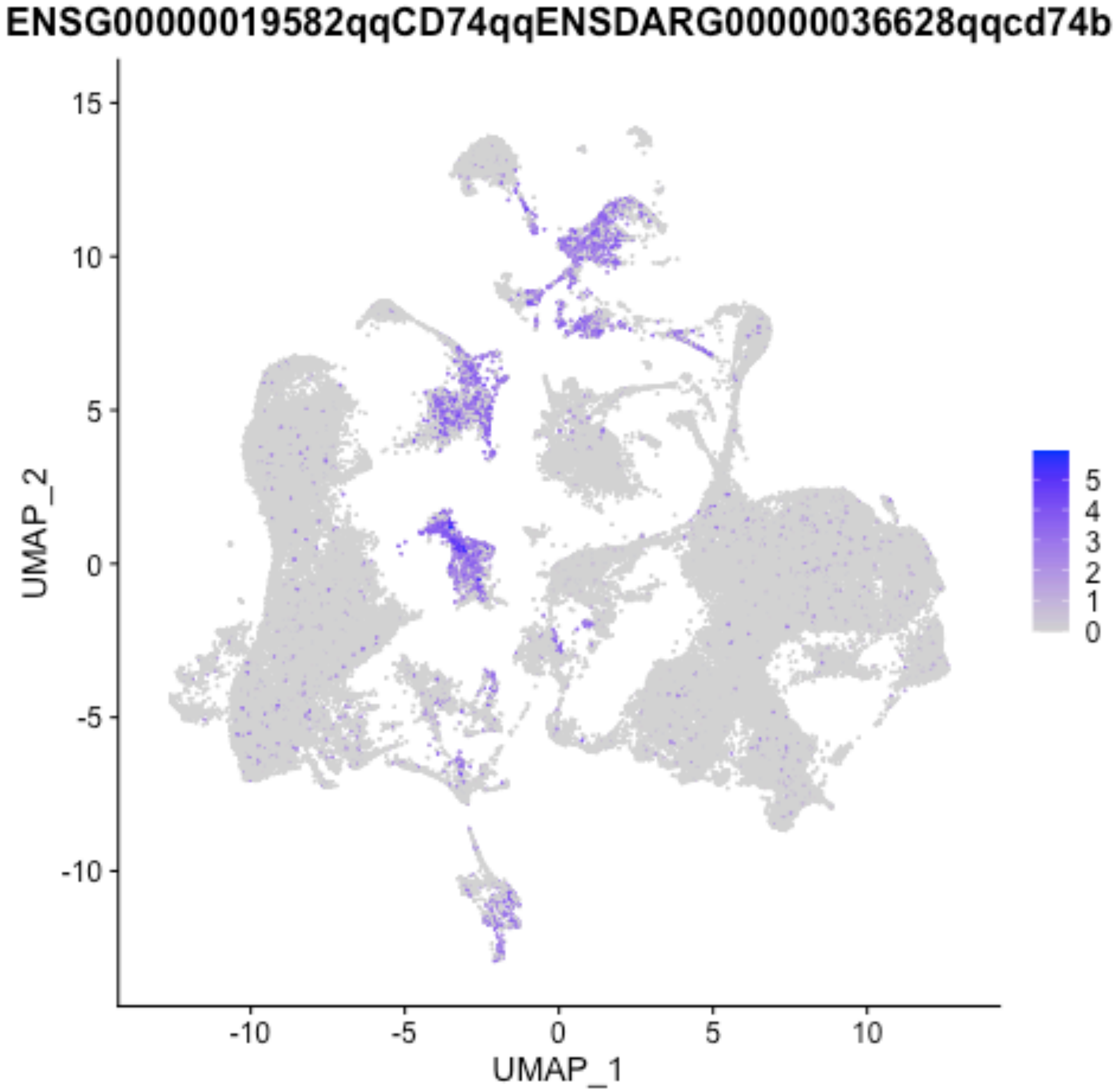
ENSG00000019582qqCD74qqENSDARG00000036628qqcd74b
UMAP_2
UMAP_1
15
10
5
0
-5
-10
-10
-5
0
5
10
5
4
3
2
1
0

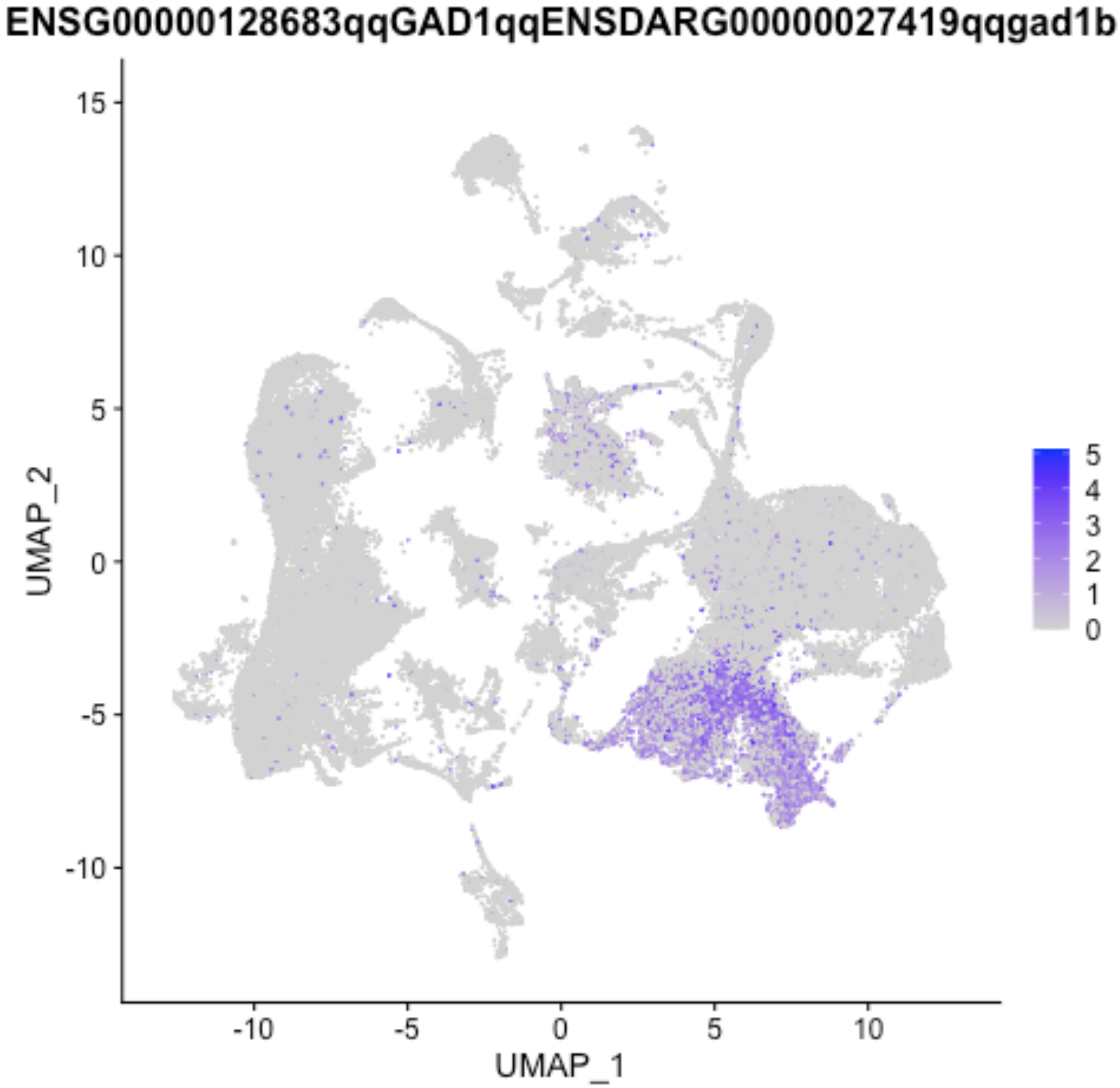
ENSG00000128683qqGAD1qqENSDARG00000027419qqgad1b
15
10
5
0
-5
-10
-10
-5
0
5
10
UMAP_1
UMAP_2
5
4
3
2
1
0

ENSG00000128683qqGAD1qqENSDARG00000093411qqgad1a

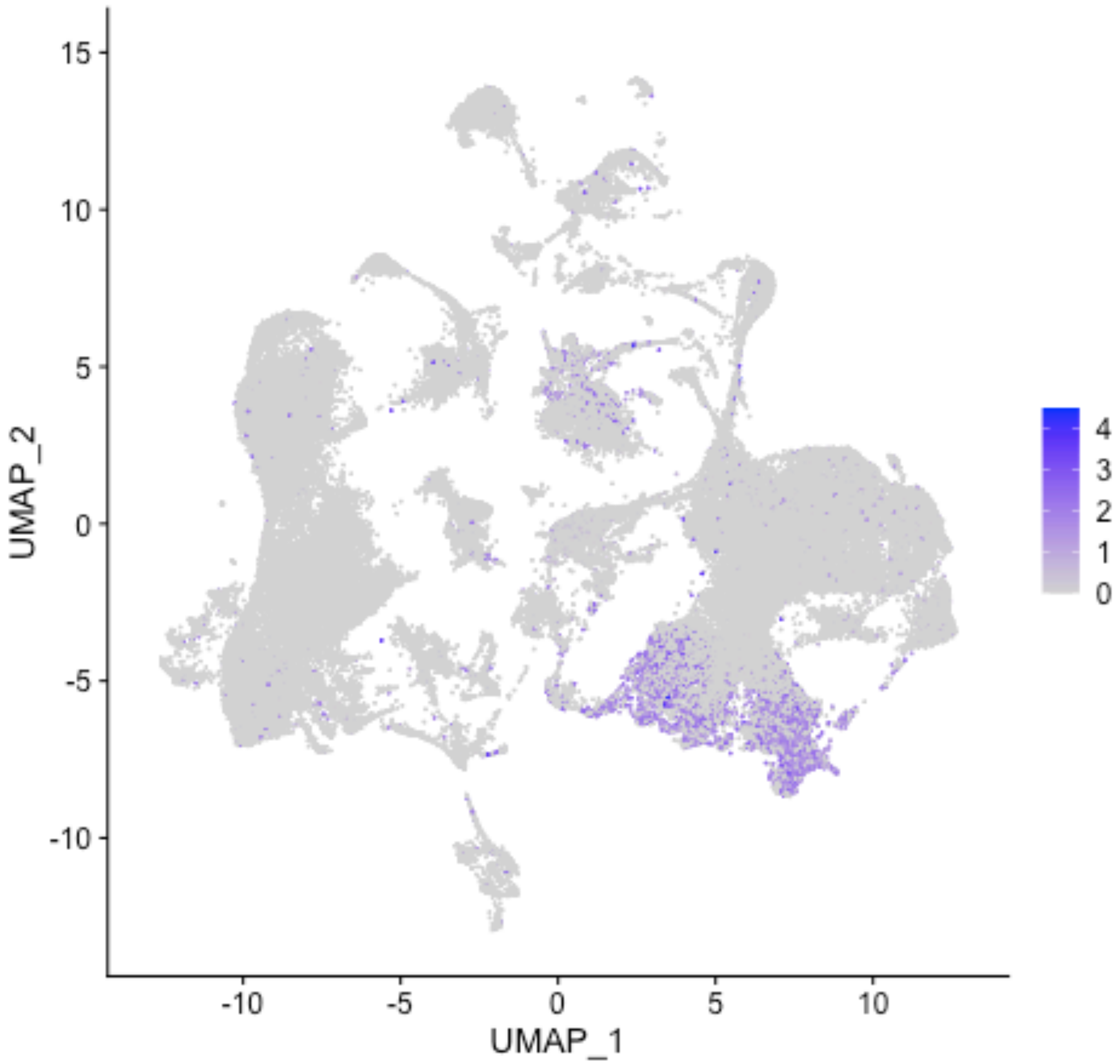

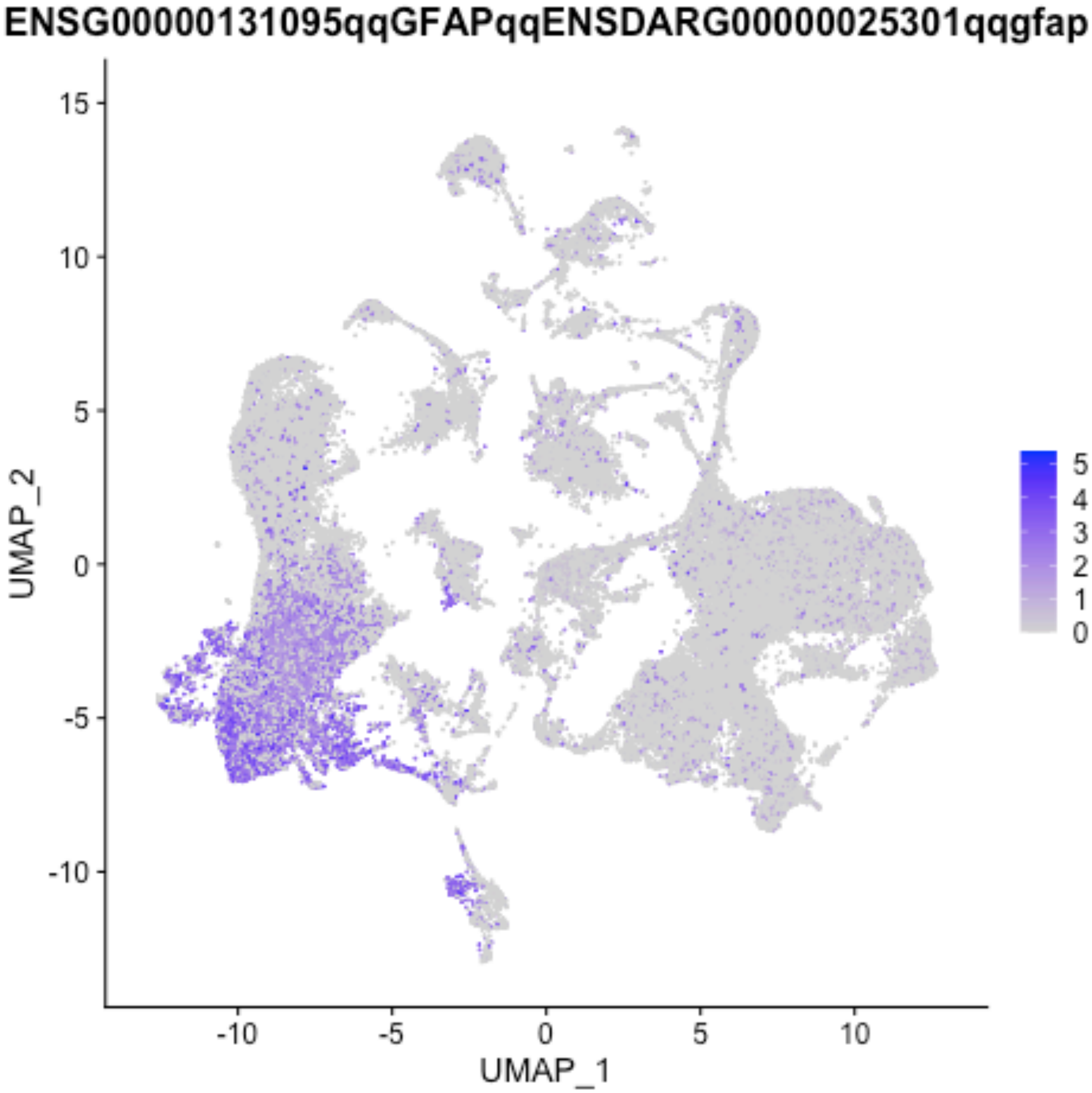
ENSG00000131095qqGFAPqqENSDARG00000025301qqgfap
UMAP_2
UMAP_1
15
10
5
0
-5
-10
-10
-5
0
5
10
5
4
3
2
1
0

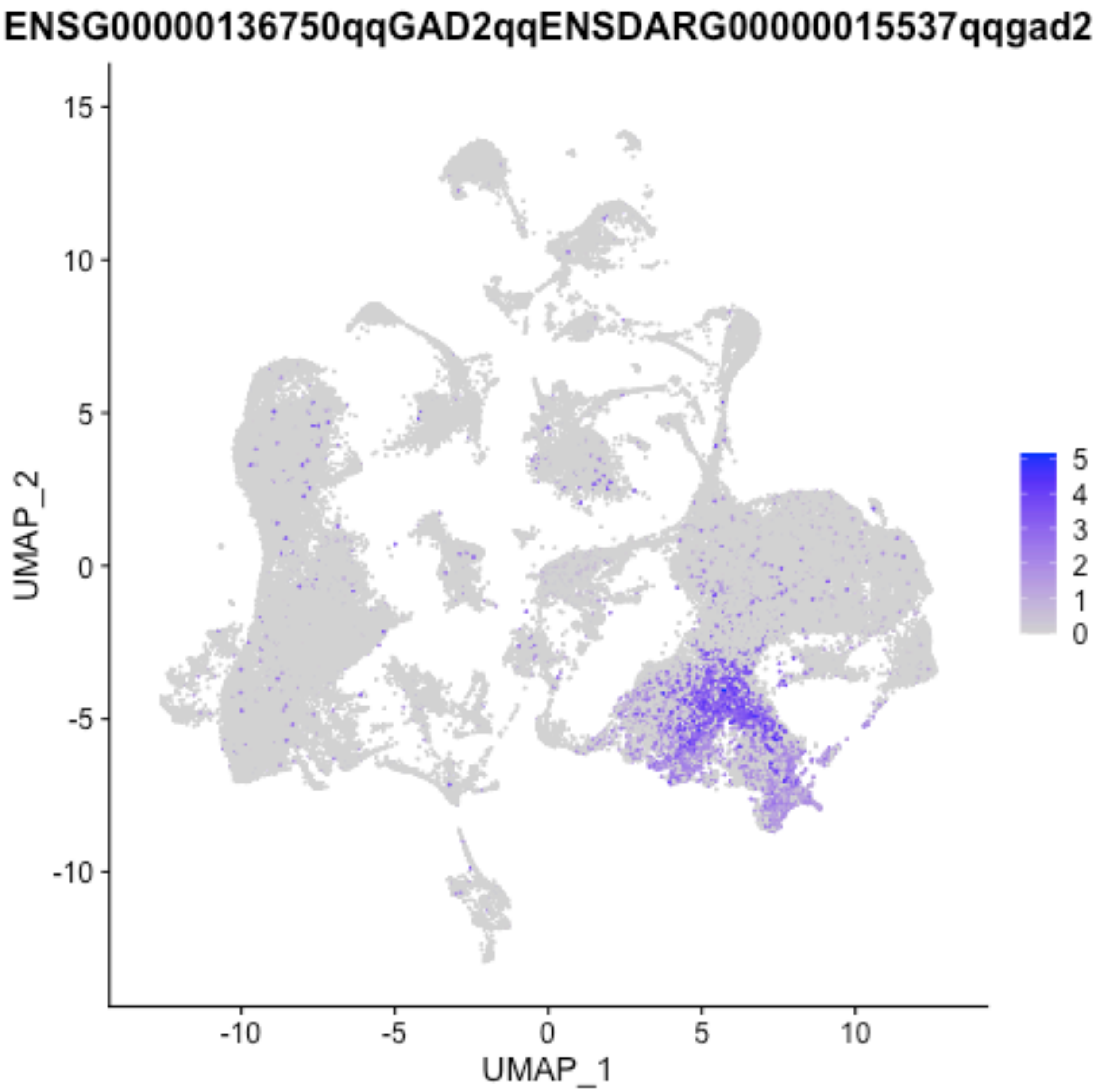

ENSG00000136750qqGAD2qqENSDARG00000015537qqgad2
15
10
5
0
-5
-10
UMAP_2
-10
-5
0
5
10
UMAP_1
5
4
3
2
1
0

ENSG00000159164qqSV2AqqENSDARG00000059945qqsv2a

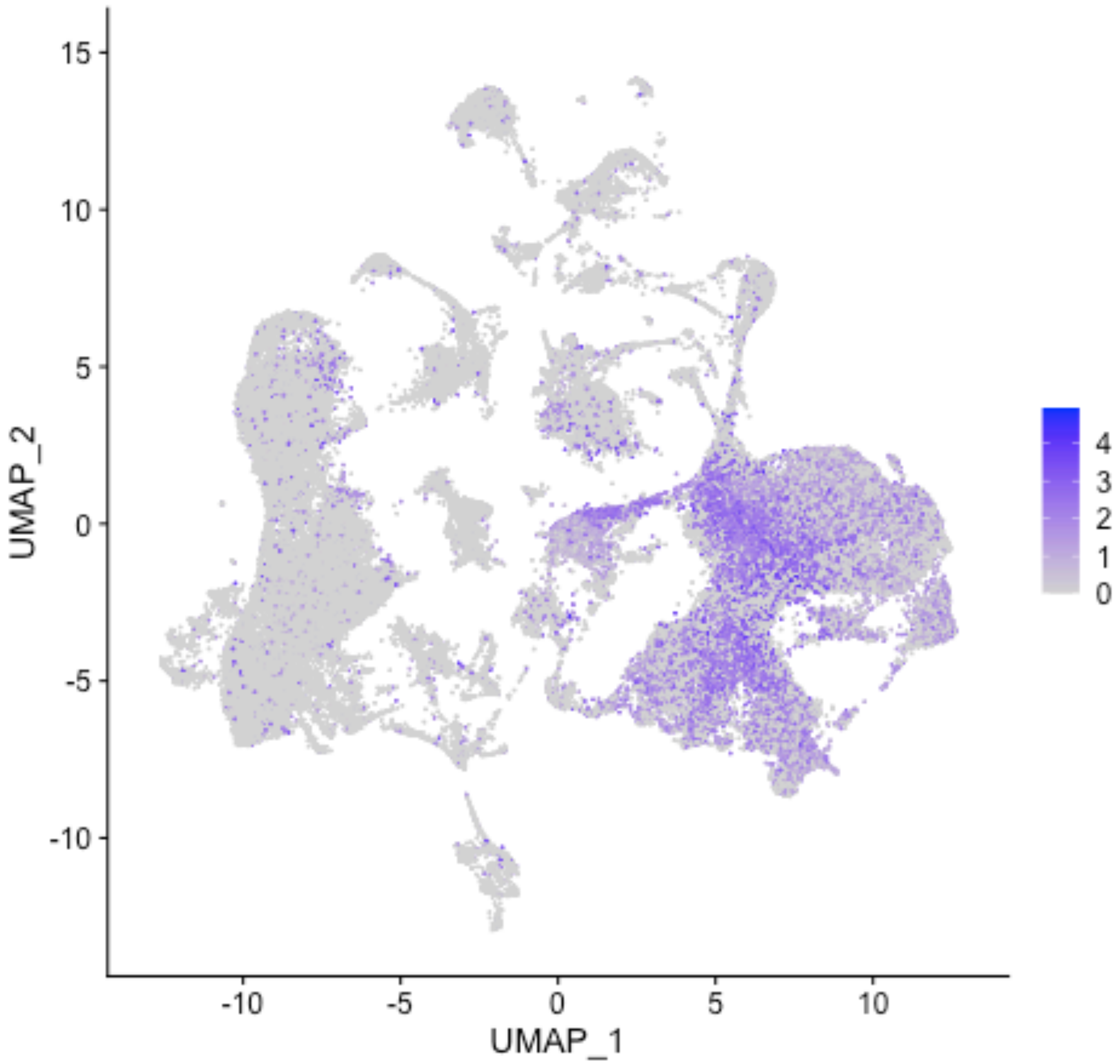

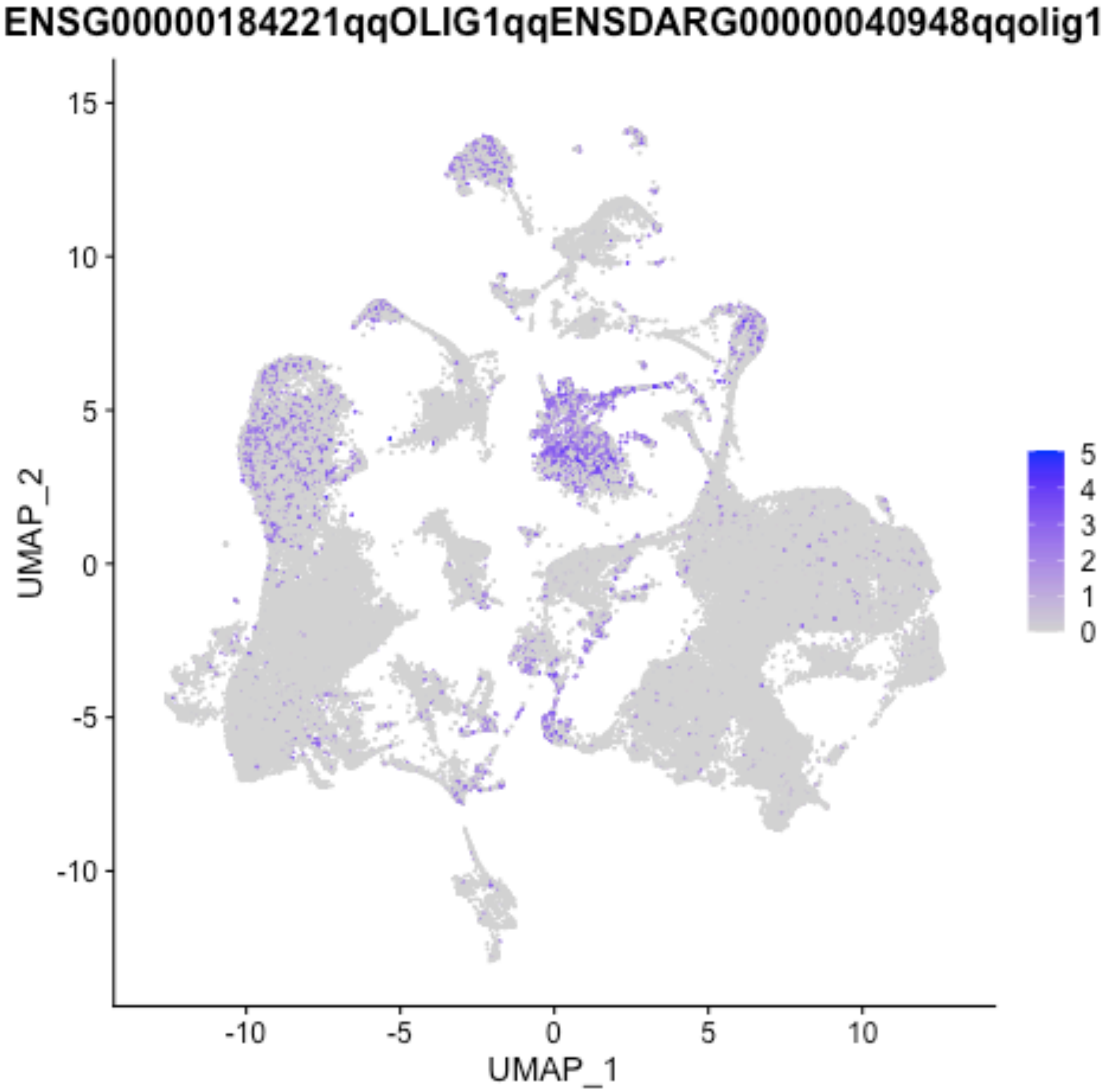
ENSG00000184221qqOLIG1qqENSDARG00000040948qqolig1
UMAP_2
UMAP_1
15
10
5
0
-5
-10
-10
-5
0
5
10
5
4
3
2
1
0

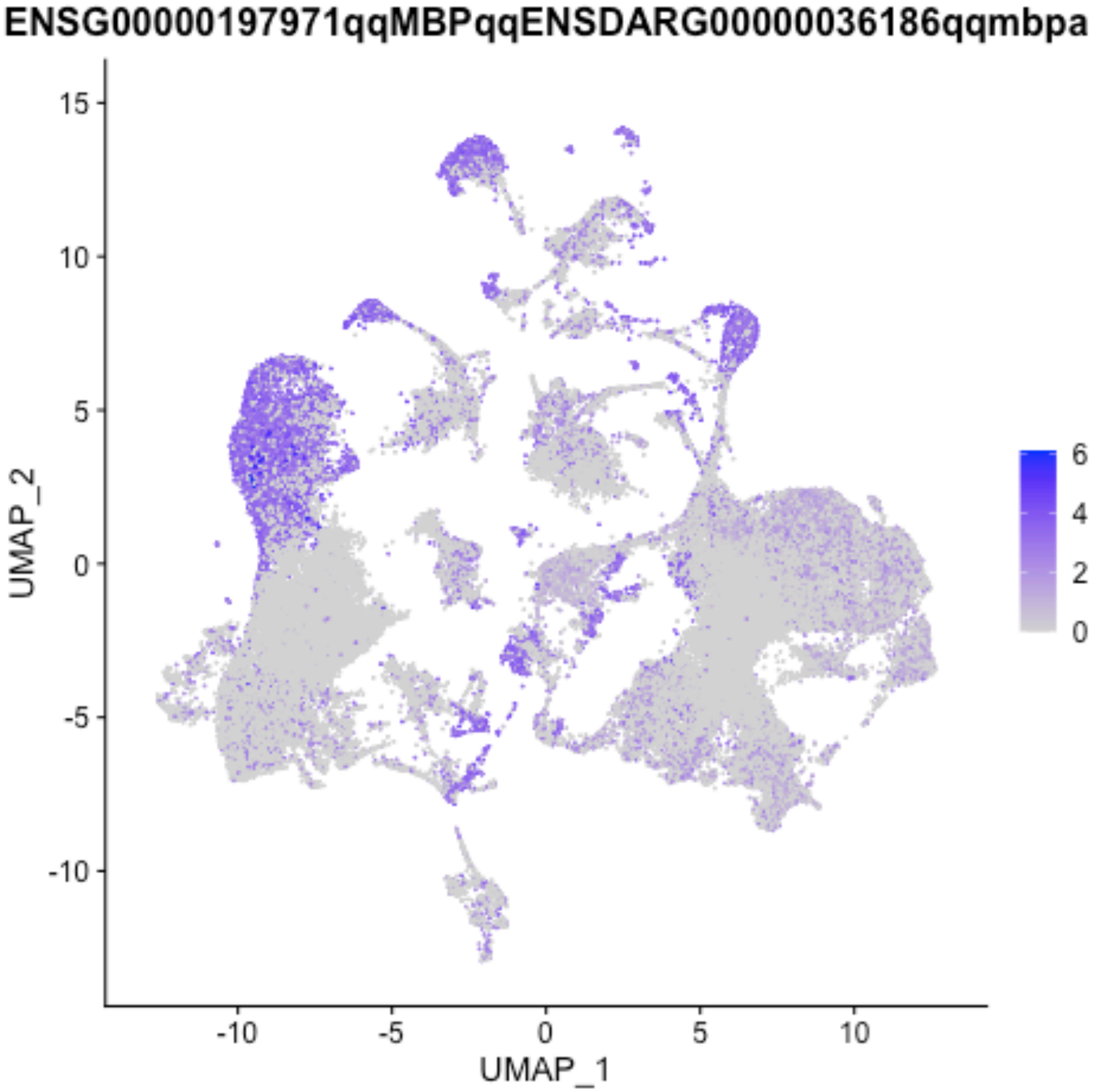
ENSG00000197971qqMBPqqENSDARG00000036186qqmbpa
UMAP_2
UMAP_1
15
10
5
0
-5
-10
-10
-5
0
5
10
6
4
2
0

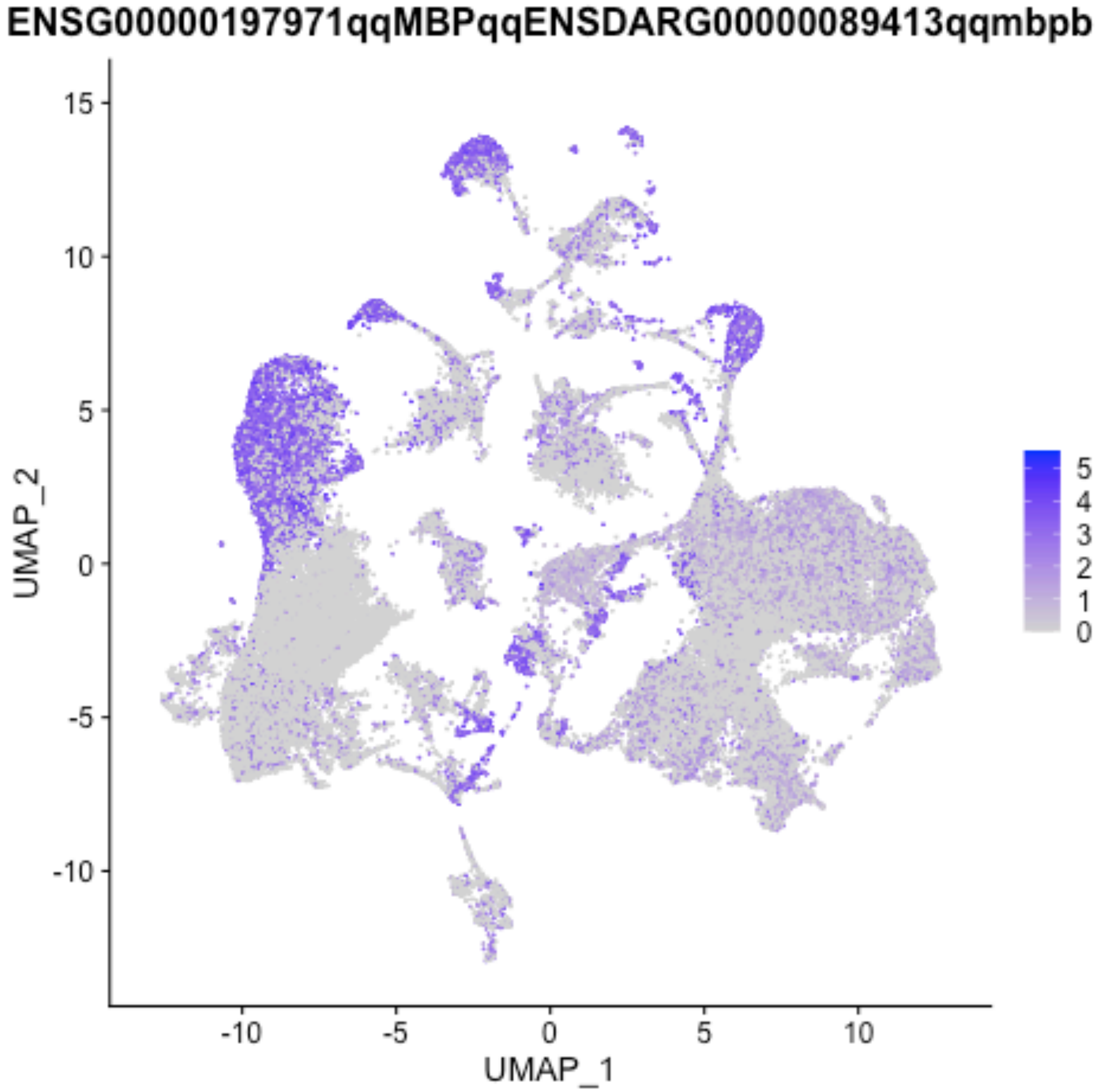
ENSG00000197971qqMBPqqENSDARG00000089413qqmbpb
UMAP_2
15
10
5
0
-5
-10
UMAP_1
-10
-5
0
5
10
5
4
3
2
1
0

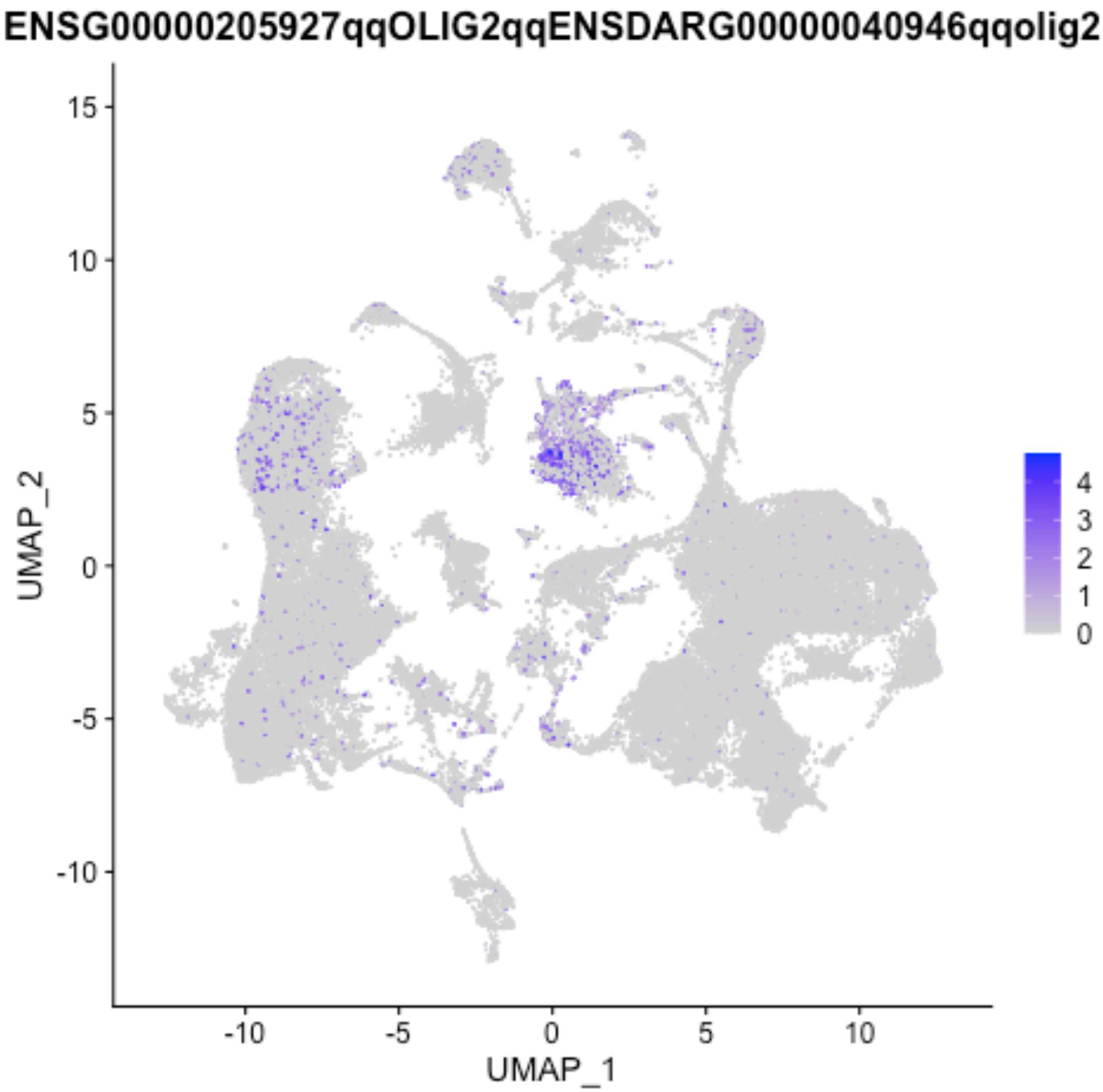
ENSG00000205927qqOLIG2qqENSDARG00000040946qqolig2
UMAP_2
UMAP_1
15
10
5
0
-5
-10
-10
-5
0
5
10
4
3
2
1
0
