# Supplementary figures and images for: Single Cell/Nucleus Transcriptomics Comparison in Zebrafish and Humans Reveals Common and Distinct Molecular Responses to Alzheimer’s Disease (part 2 of 2)
Source: Cells. 2022 May 31;11(11):1807. doi: 10.3390/cells11111807 (PMC9180693; doi:10.3390/cells11111807)

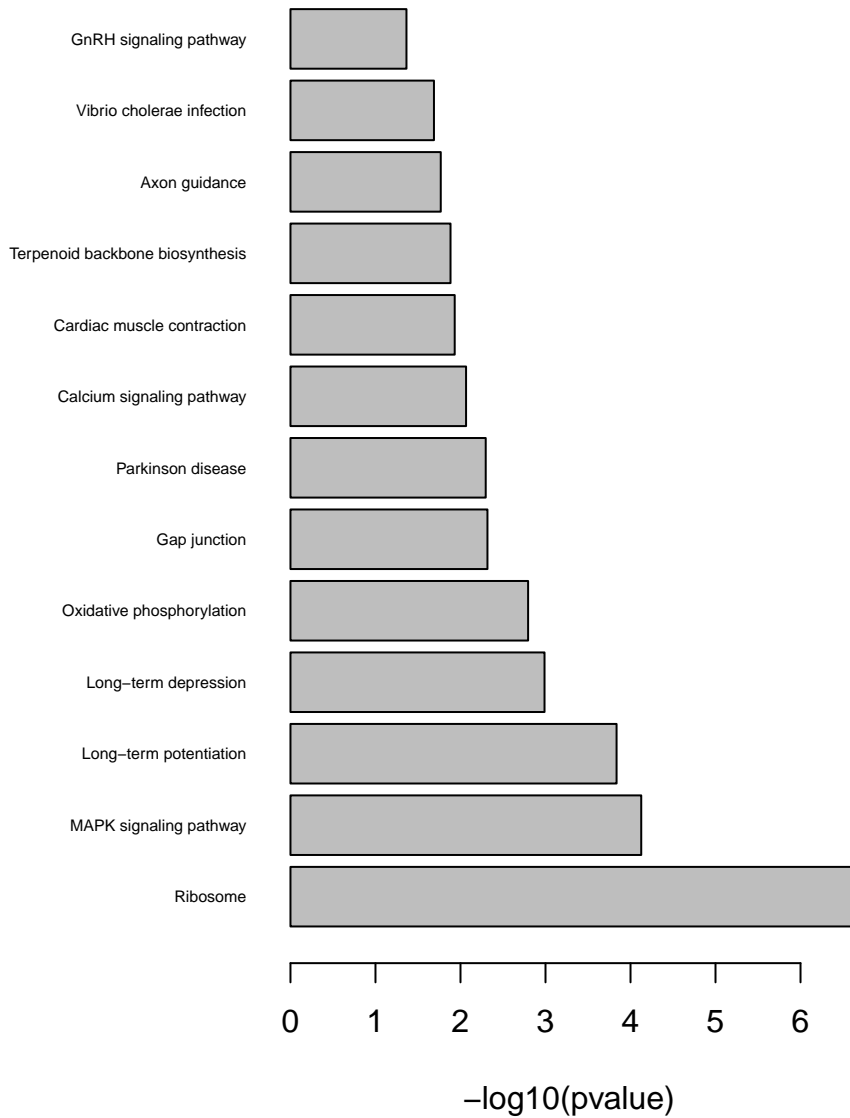

Supplement: Supplementary file 1 [file cells-11-01807-s001.zip › Supplementary_Data/DataS5/EC_cells_GOKEGGs/ECBS6_8_vs_ECBS0_8/pVal_GOstats_kegg_Up.pdf]

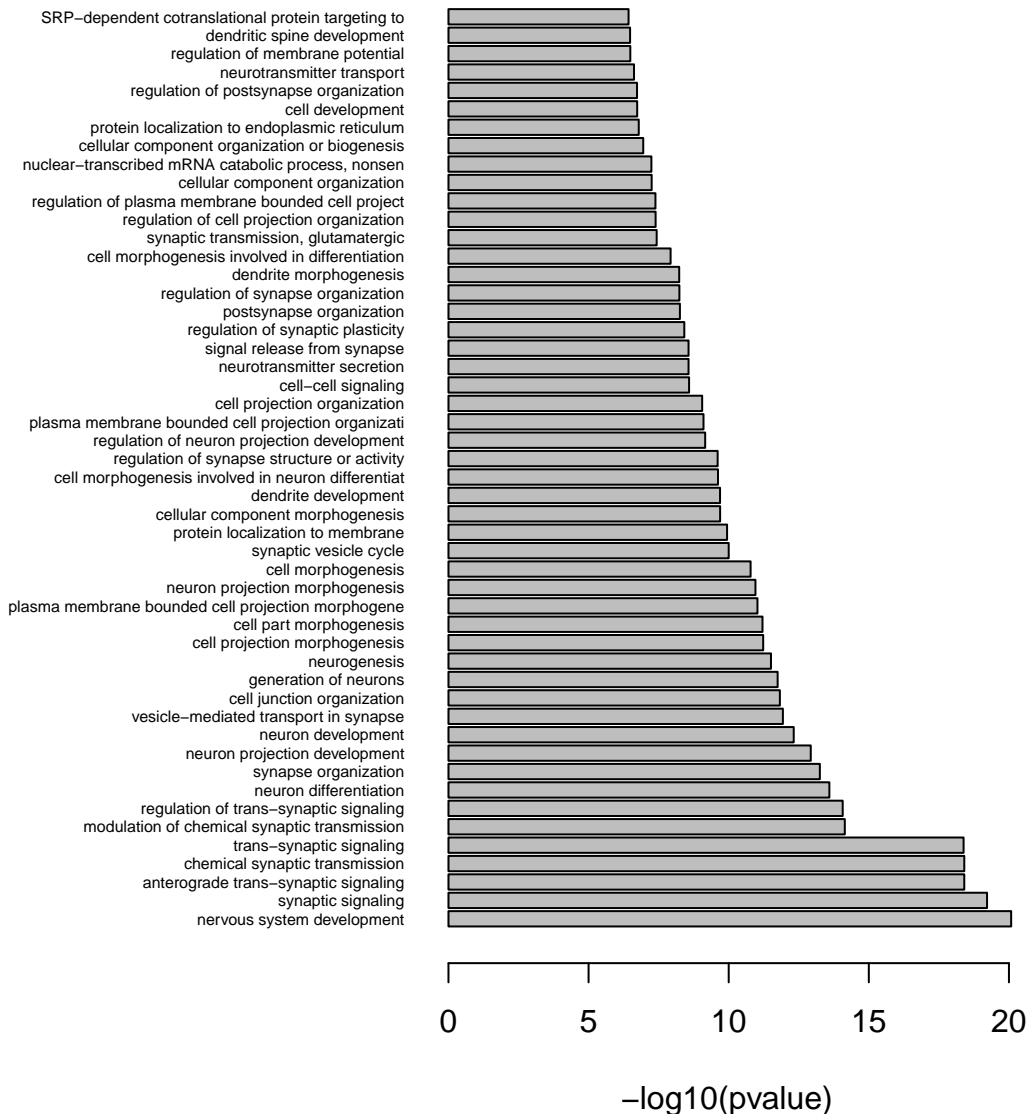

Supplement: Supplementary file 1 [file cells-11-01807-s001.zip › Supplementary_Data/DataS5/EC_cells_GOKEGGs/ECBS6_8_vs_ECBS0_8/pVal_GOstats_BP_Up_pieChart.pdf]

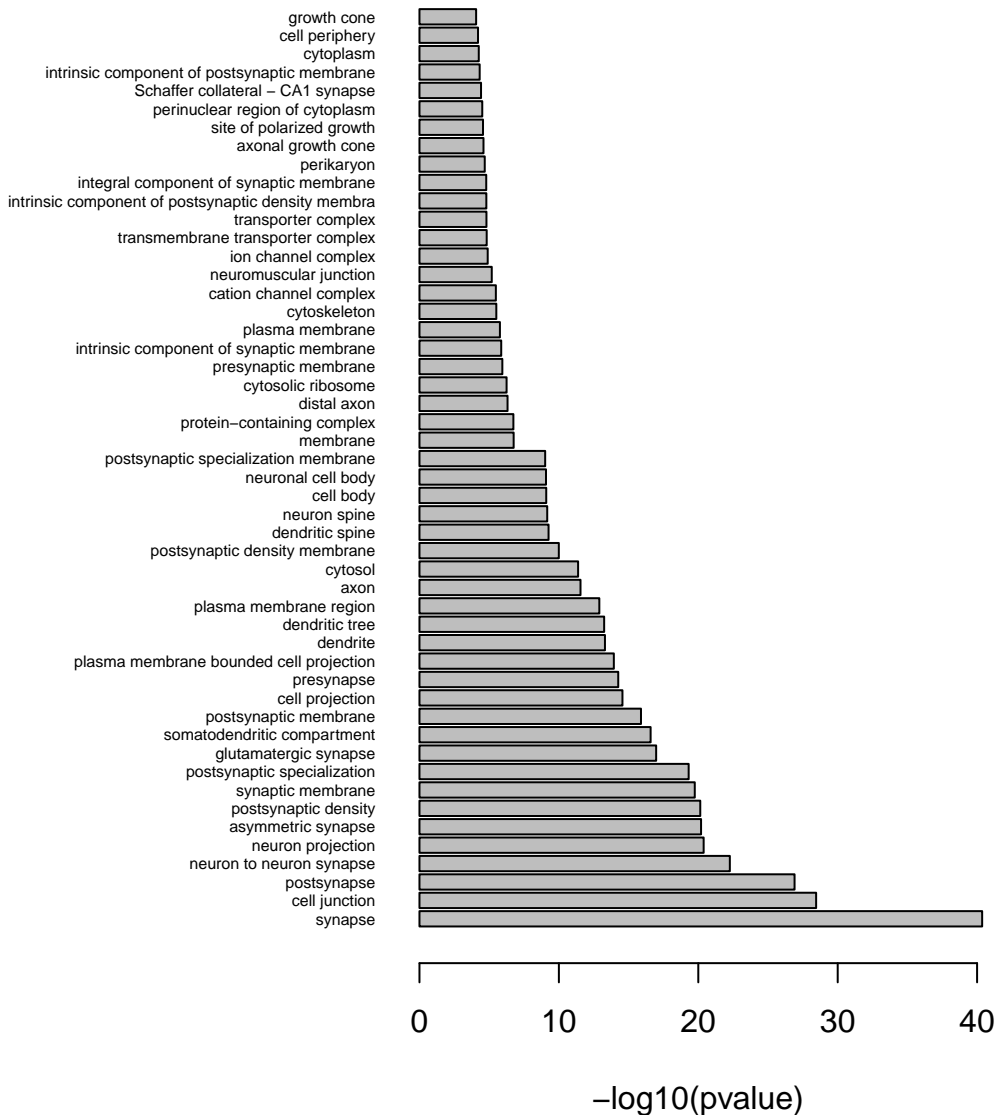

Supplement: Supplementary file 1 [file cells-11-01807-s001.zip › Supplementary_Data/DataS5/EC_cells_GOKEGGs/ECBS6_8_vs_ECBS0_8/pVal_GOstats_CC_Up_pieChart.pdf]

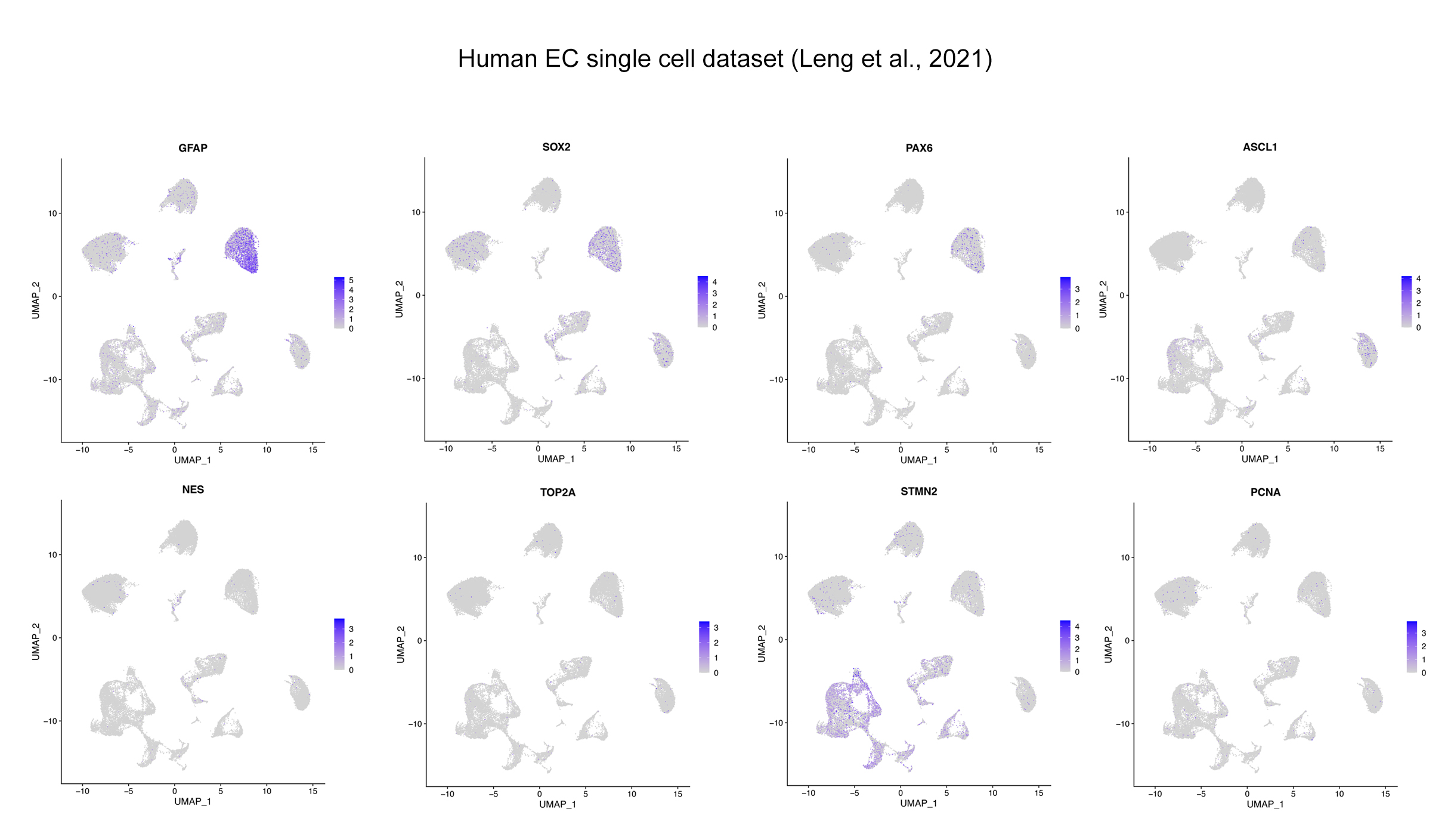

Supplement: Supplementary file 1 [file cells-11-01807-s001.zip › Supplementary_Figures/FigS5.jpg]

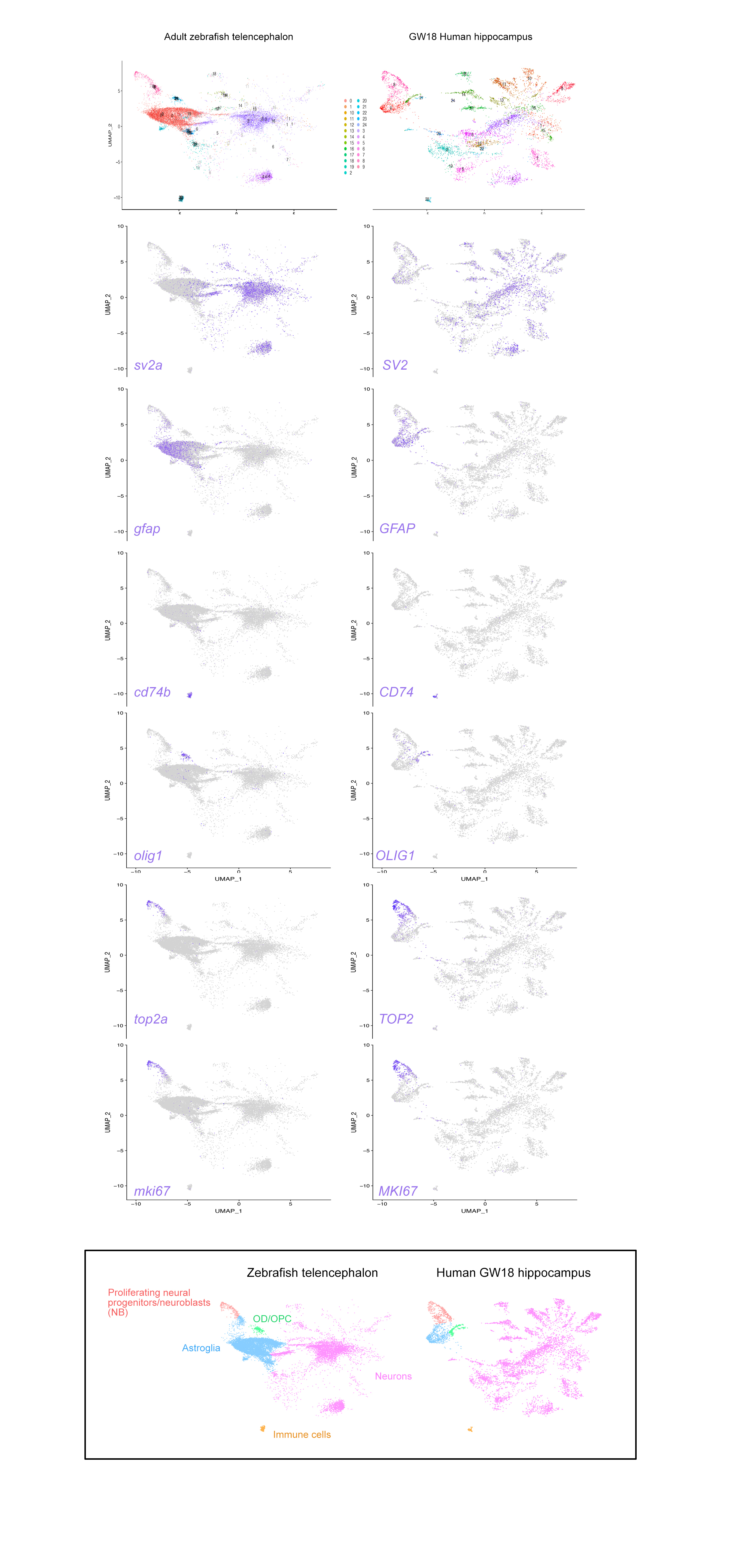

Supplement: Supplementary file 1 [file cells-11-01807-s001.zip › Supplementary_Figures/FigS4.jpg]

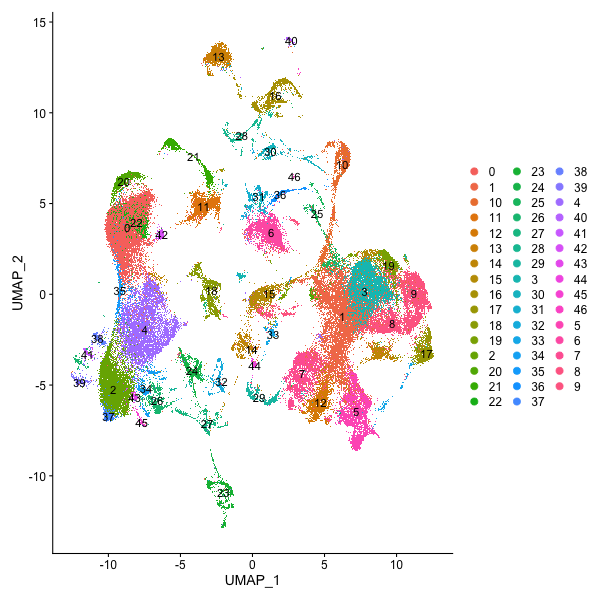

Supplement: Supplementary file 1 [file cells-11-01807-s001.zip › Supplementary_Figures/FigS1.png]

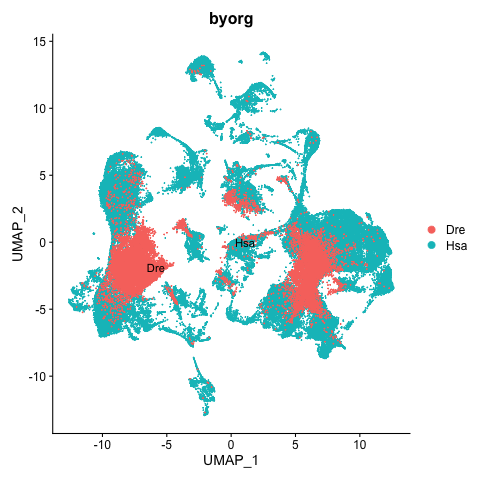

Supplement: Supplementary file 1 [file cells-11-01807-s001.zip › Supplementary_Figures/FigS3.png]
