# Supplementary figures and images for: Single Cell/Nucleus Transcriptomics Comparison in Zebrafish and Humans Reveals Common and Distinct Molecular Responses to Alzheimer’s Disease (part 1 of 2)
Source: Cells. 2022 May 31;11(11):1807. doi: 10.3390/cells11111807 (PMC9180693; doi:10.3390/cells11111807)

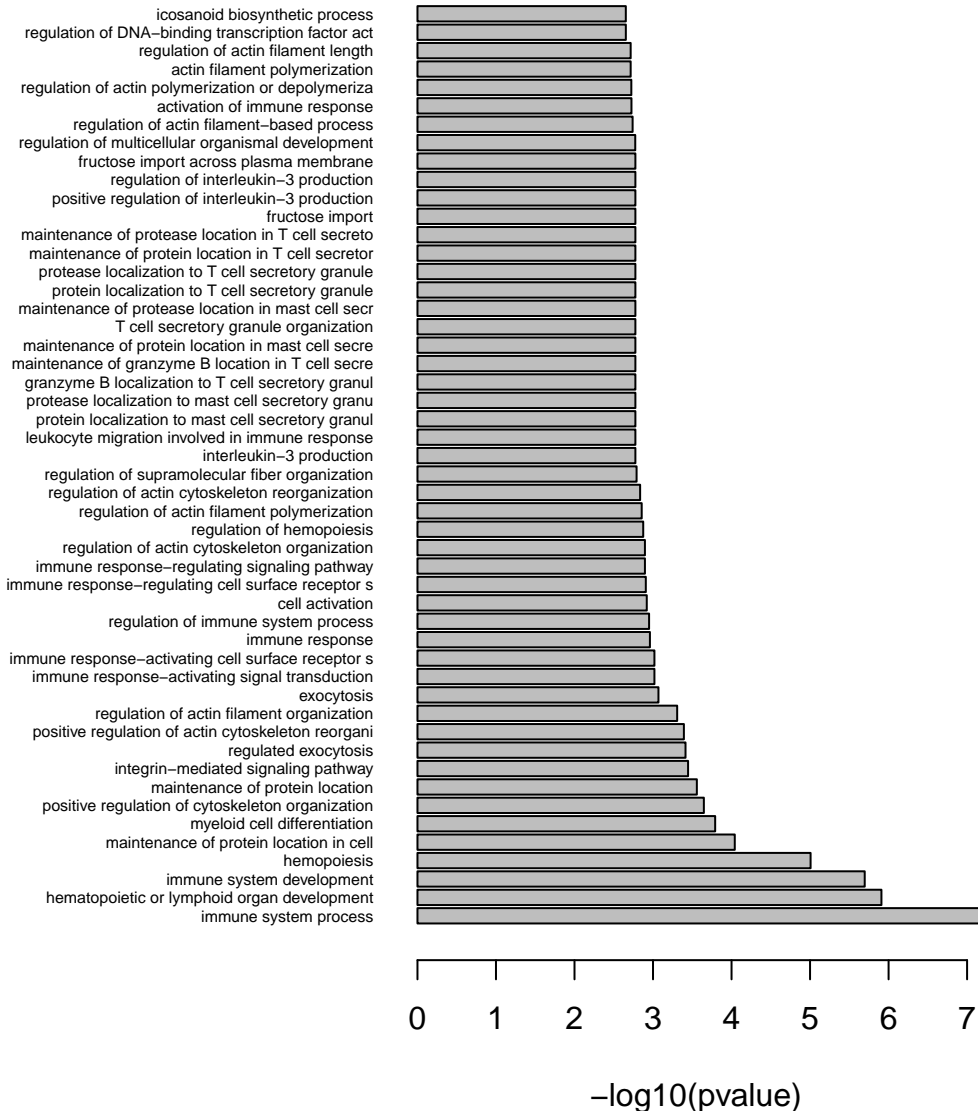

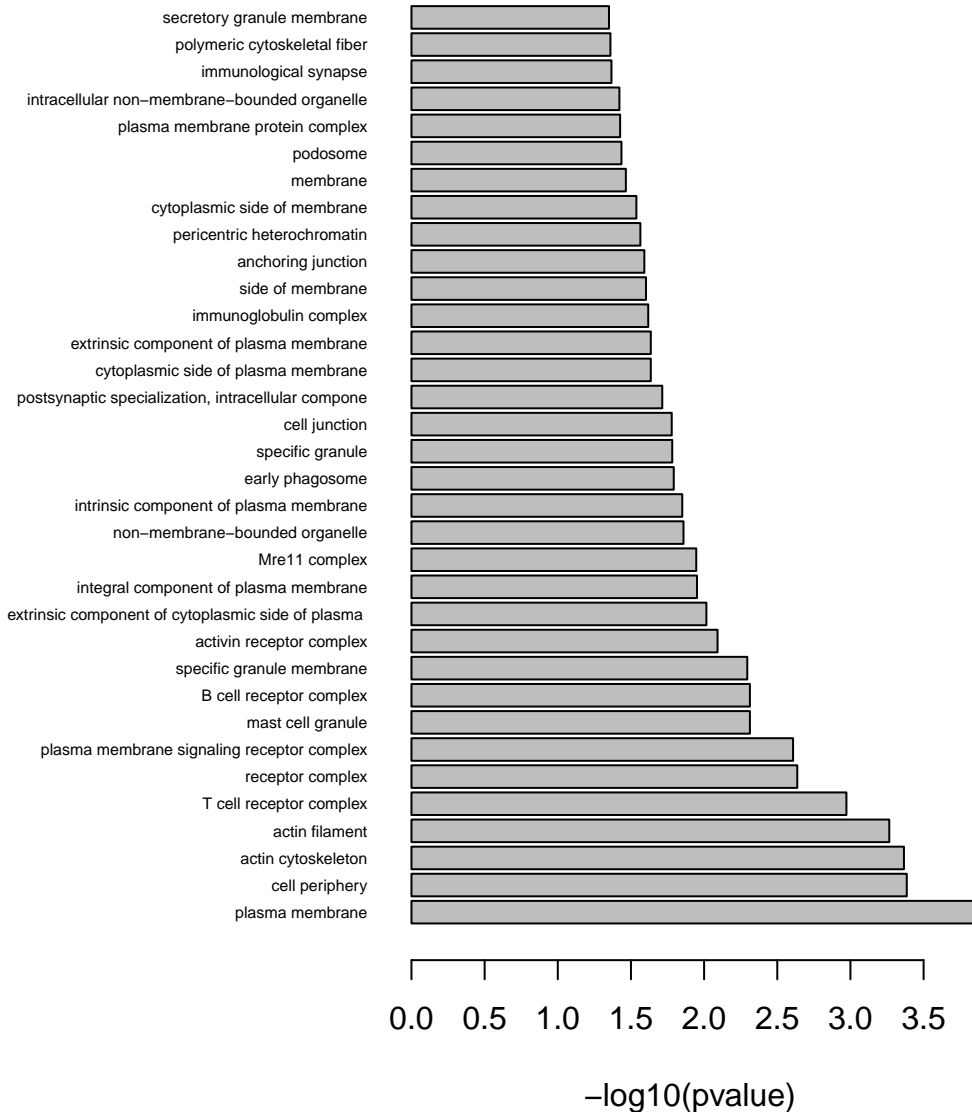

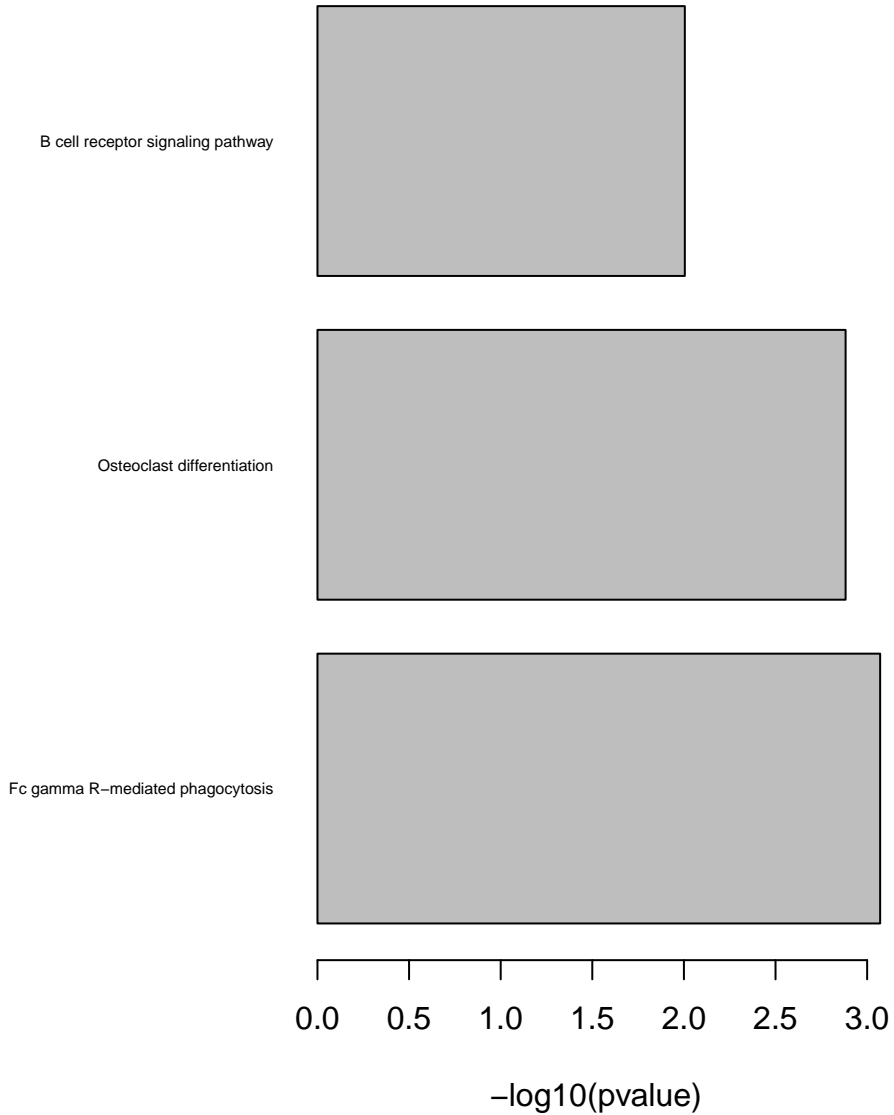

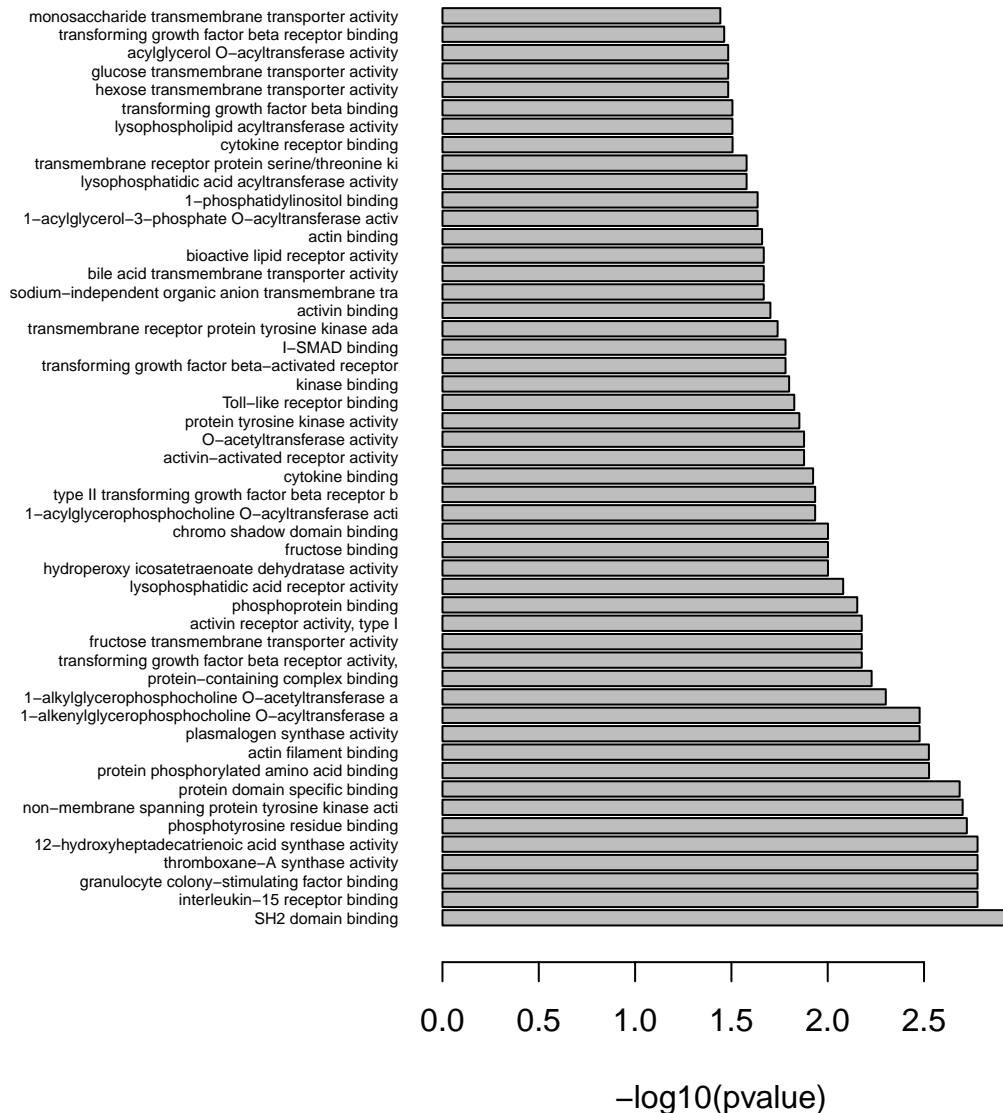

Supplement: Supplementary file 1 [file cells-11-01807-s001.zip › Supplementary_Data/DataS3/DataS3_cluster11.pdf]

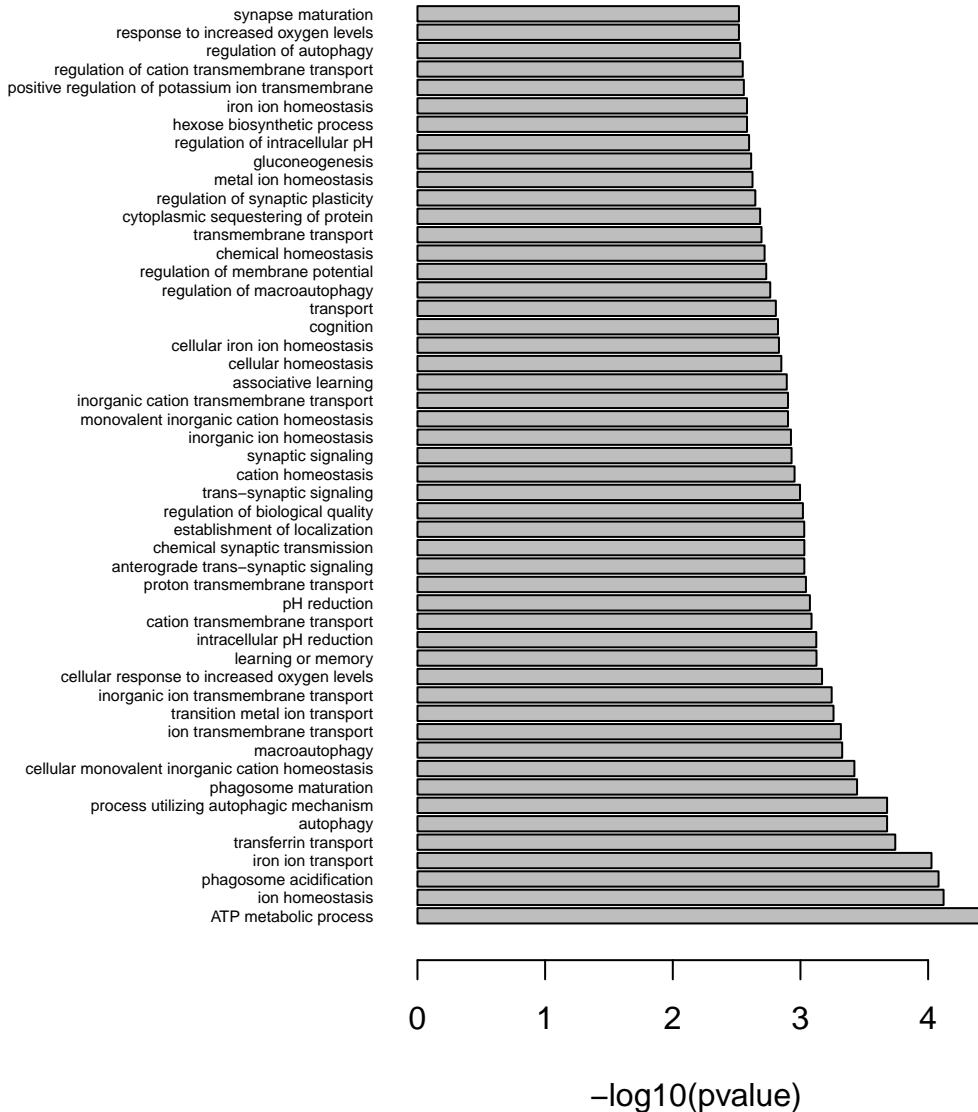

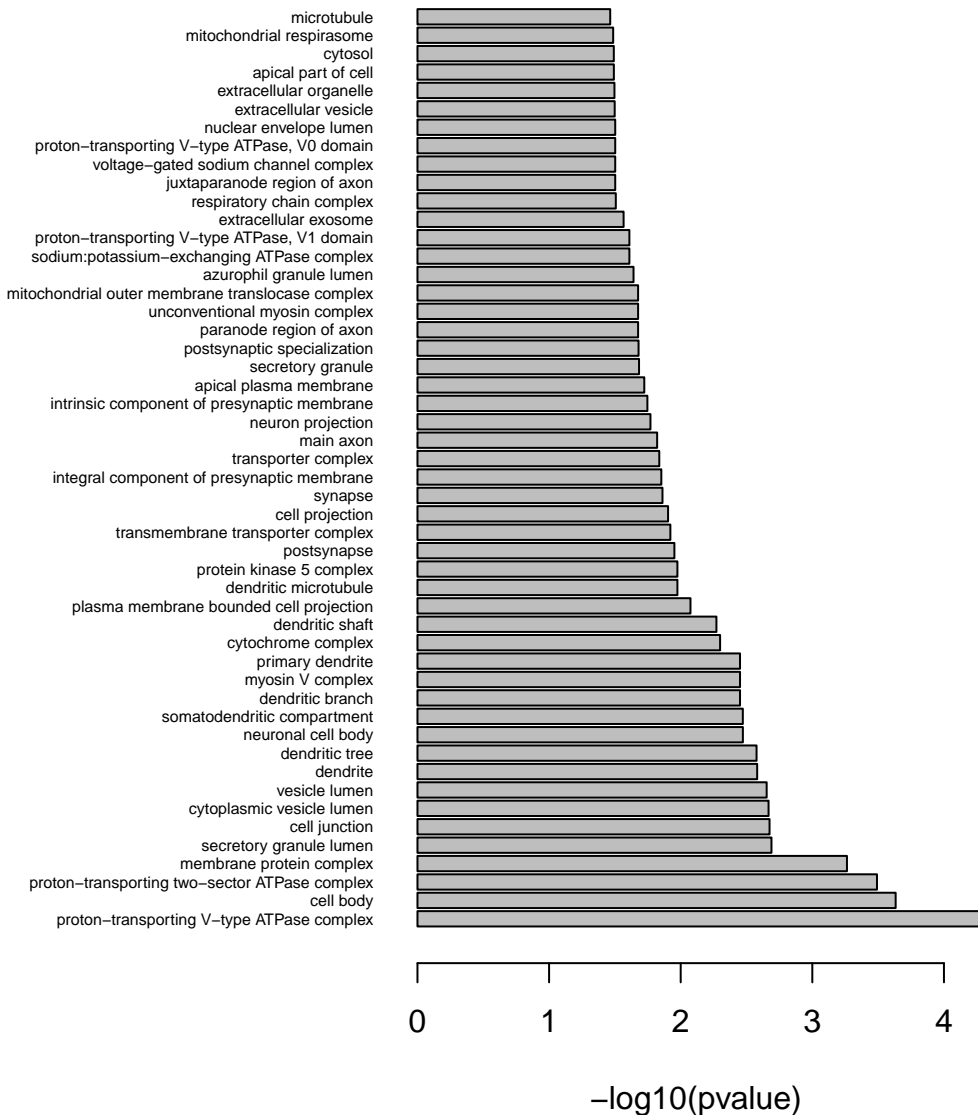

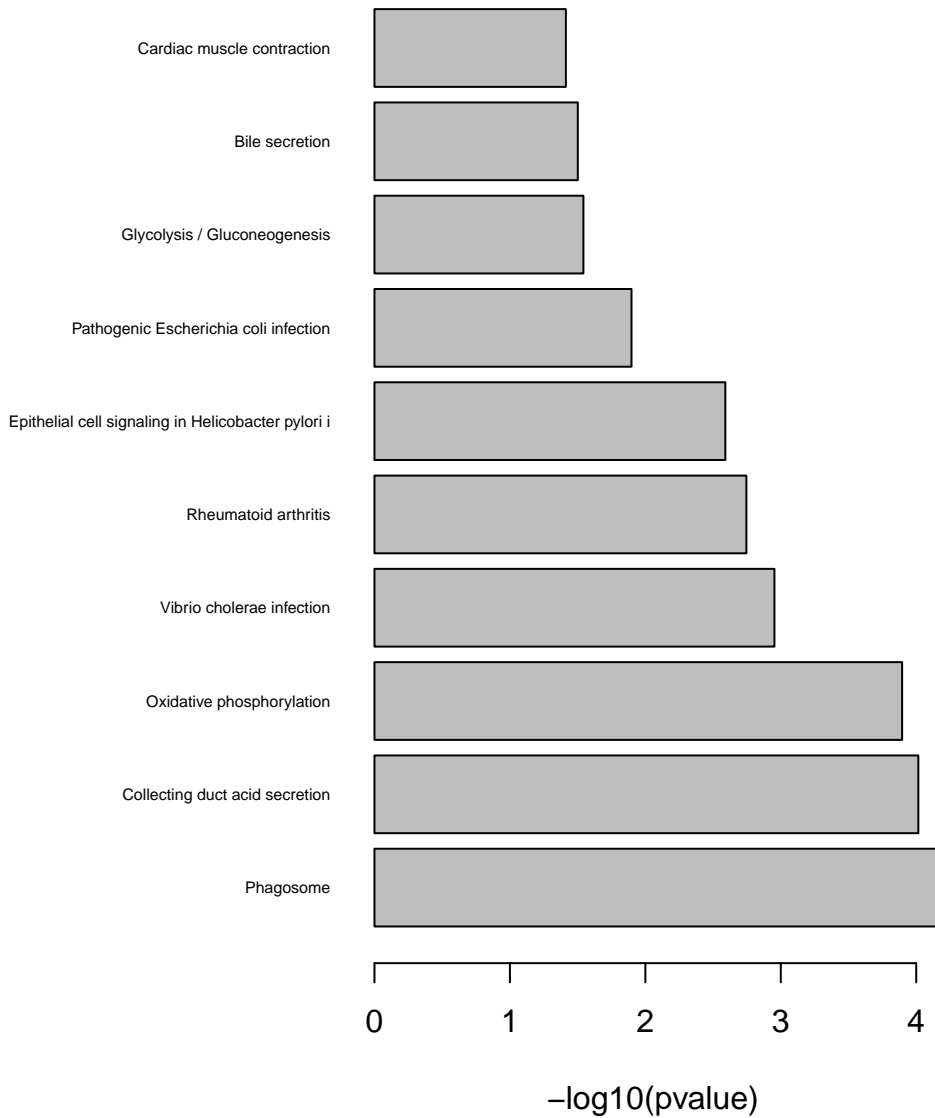

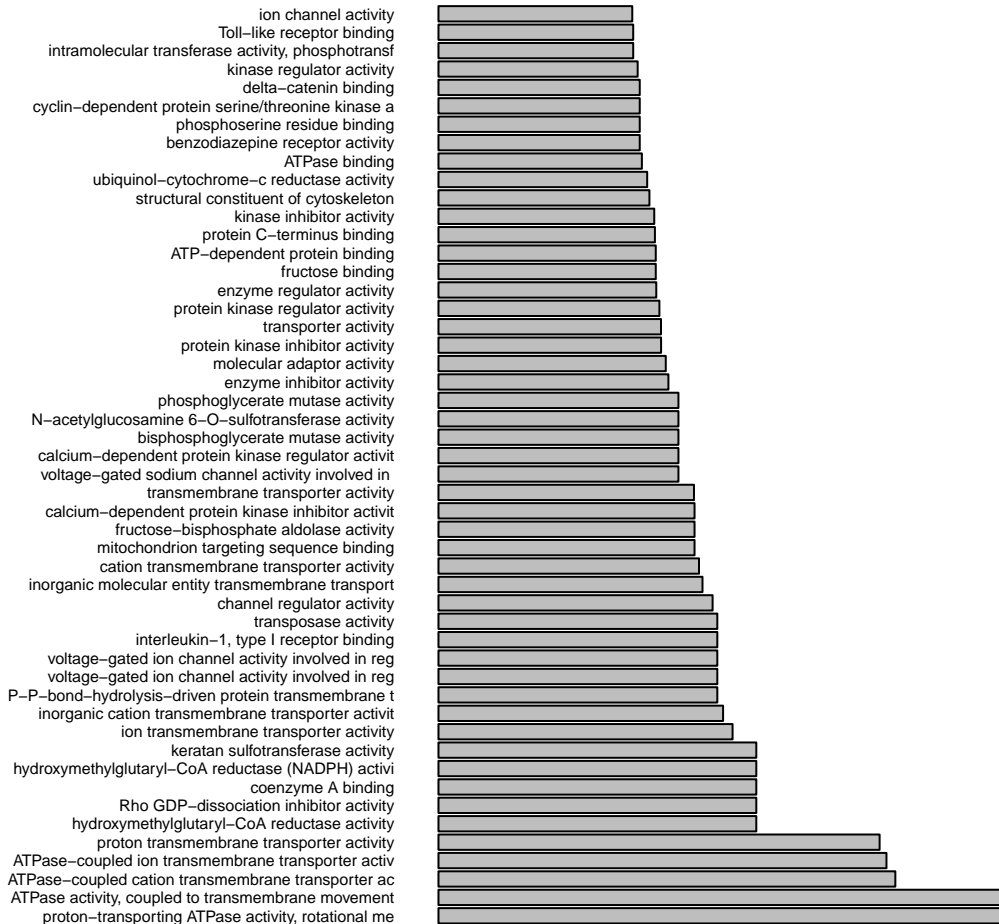

0 1 2 3 4

$-\log_{10}(\text{pvalue})$

Supplement: Supplementary file 1 [file cells-11-01807-s001.zip › Supplementary_Data/DataS3/DataS3_cluster1.pdf]

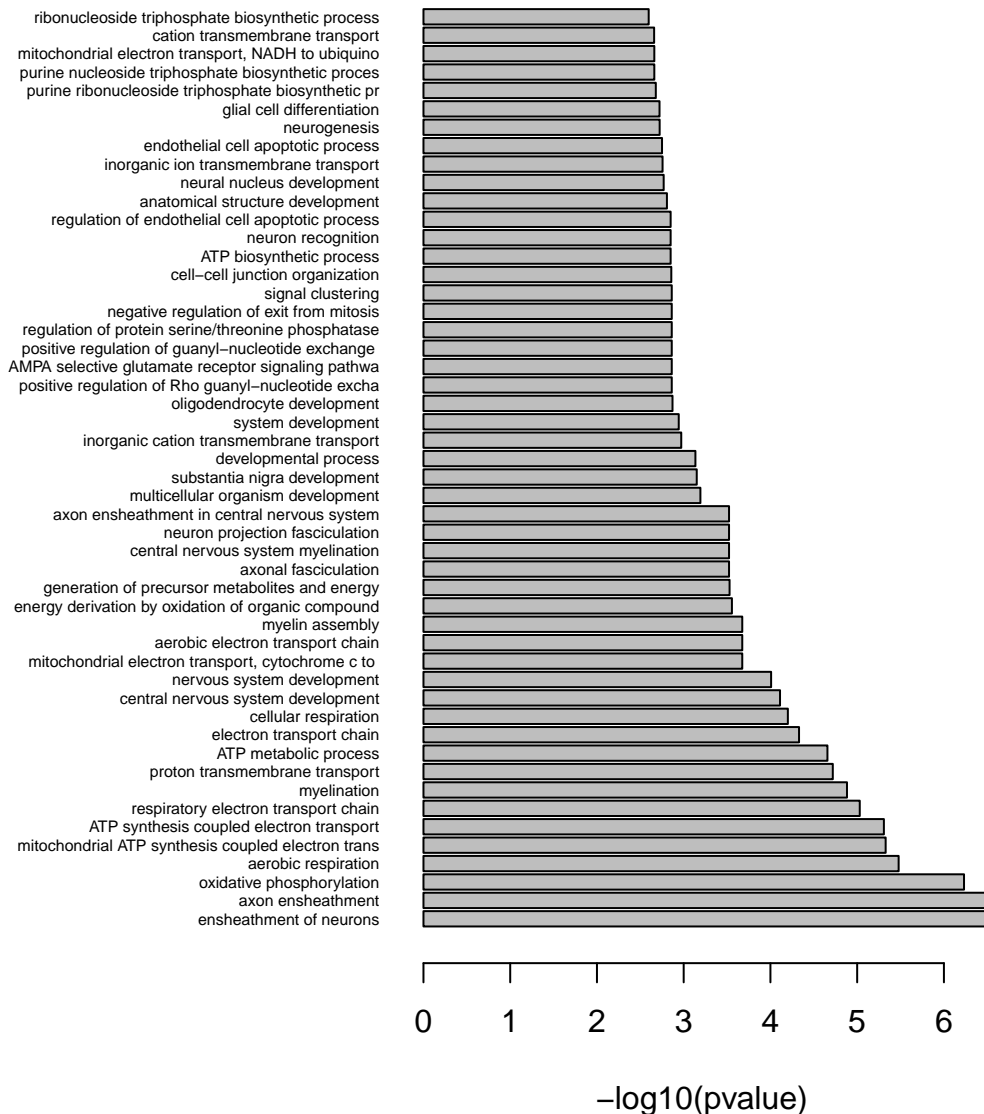

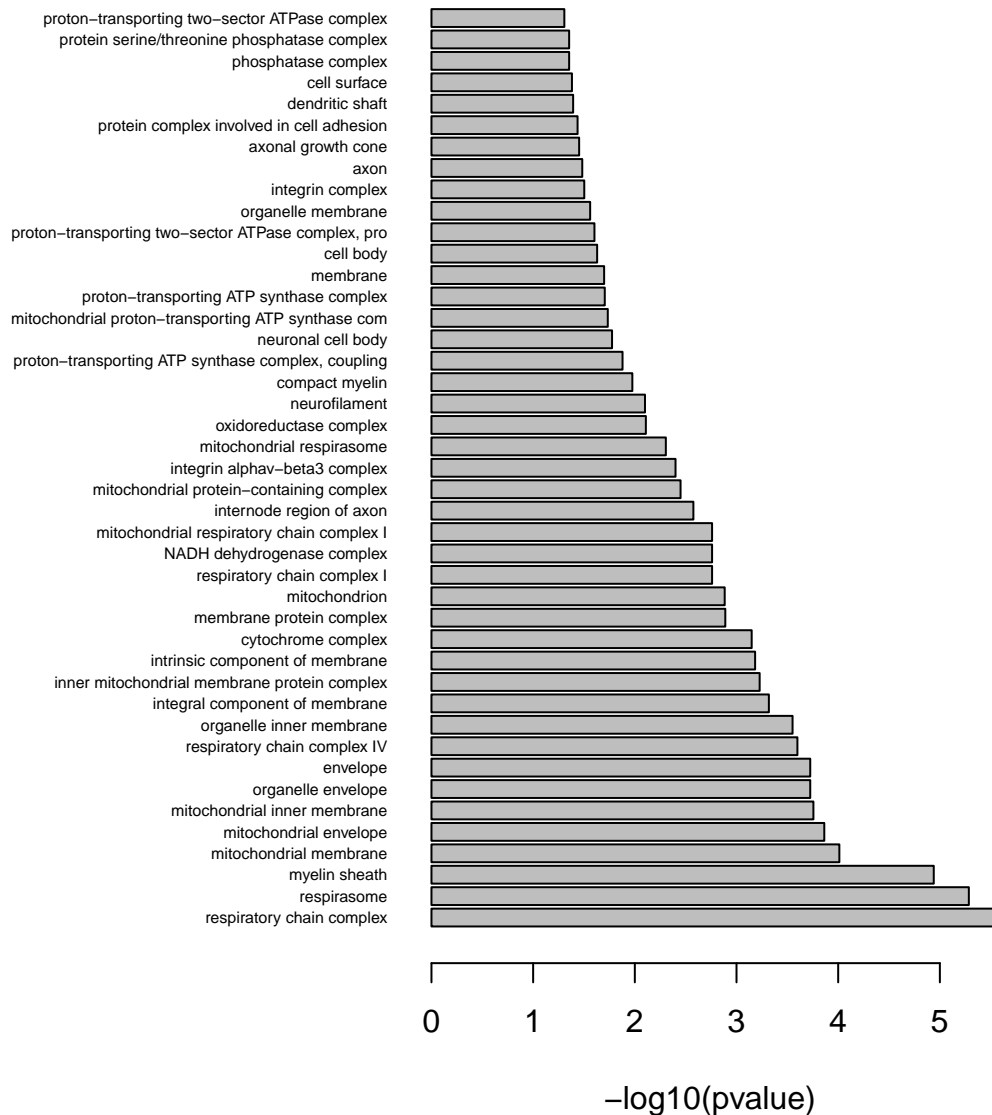

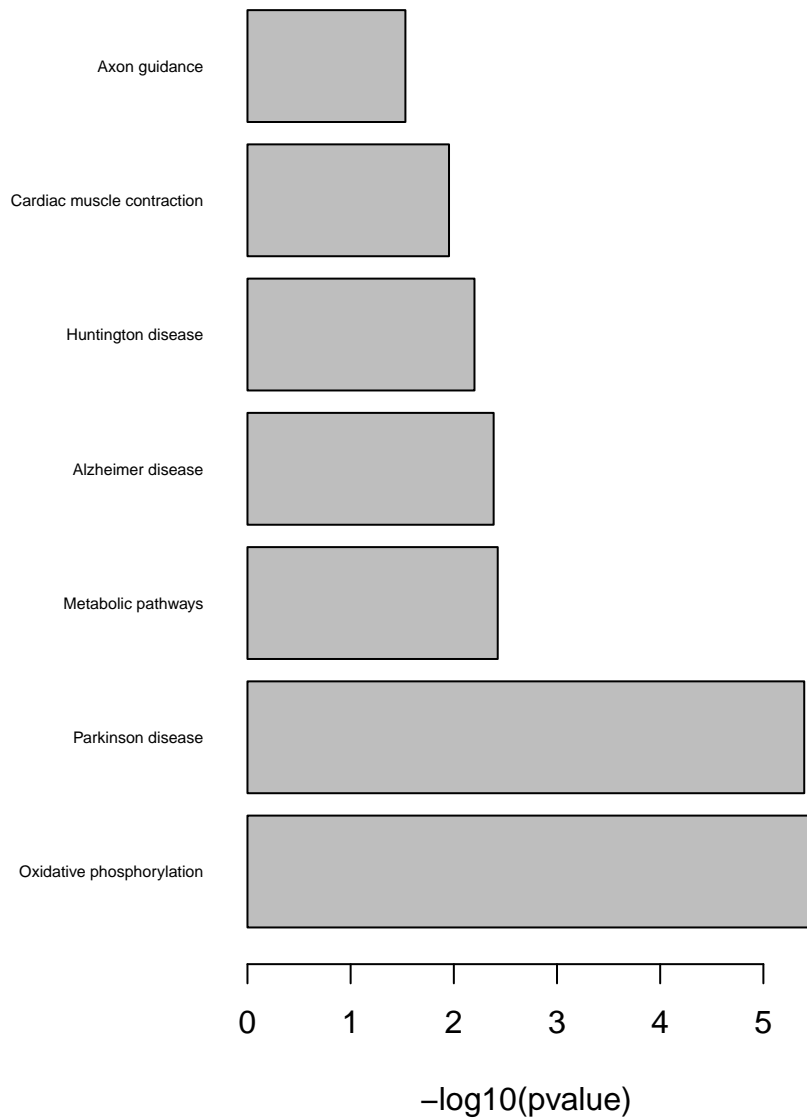

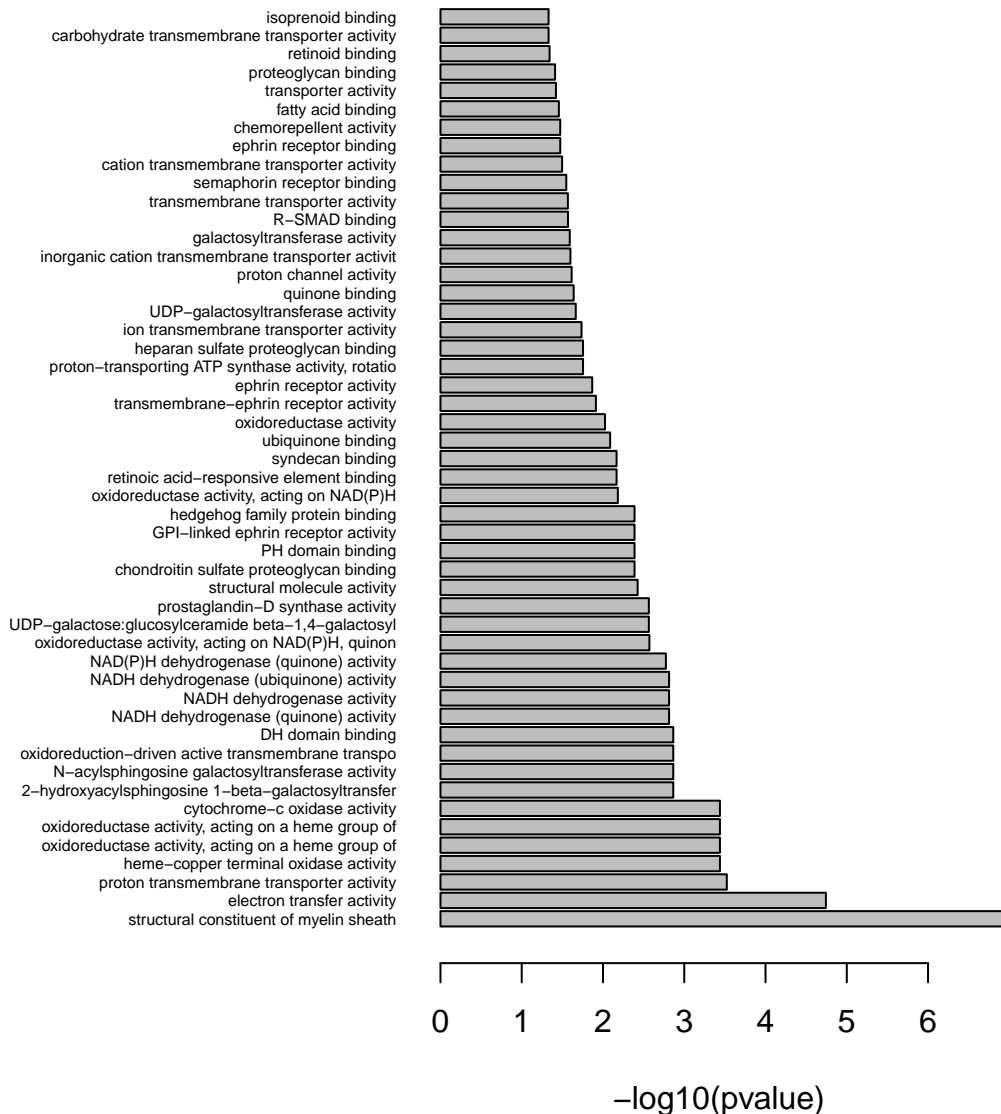

Supplement: Supplementary file 1 [file cells-11-01807-s001.zip › Supplementary_Data/DataS3/DataS3_cluster0.pdf]

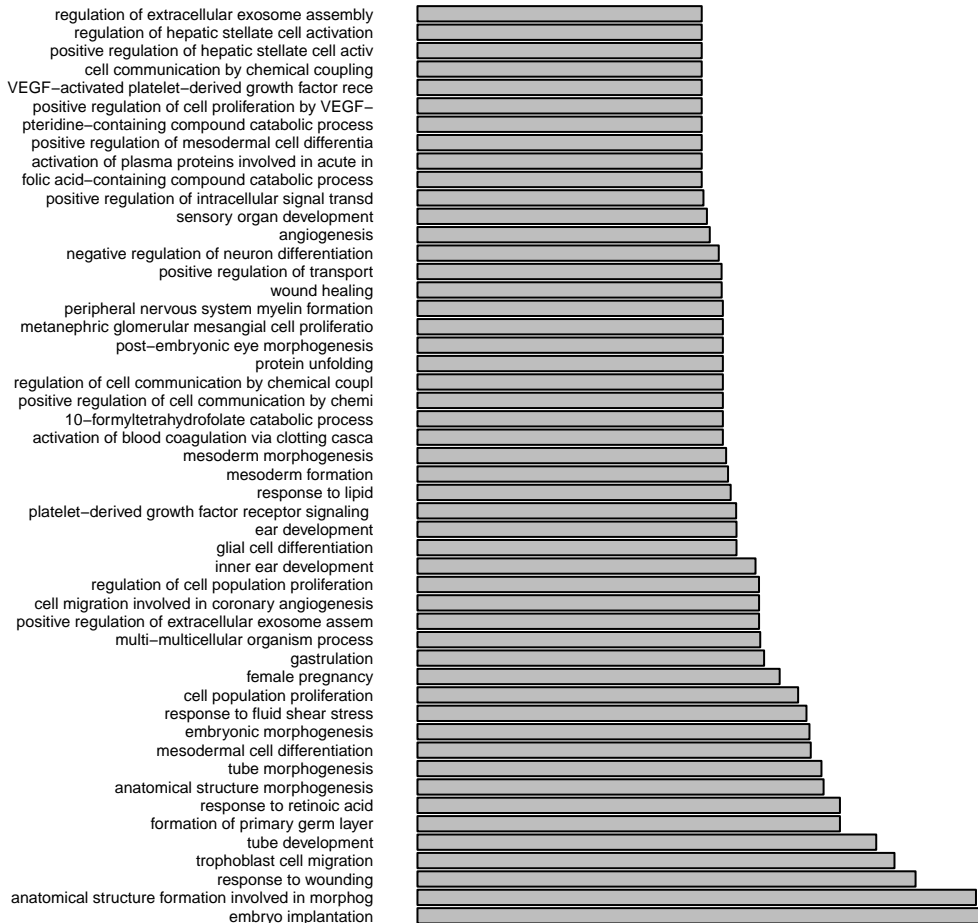

0 1 2 3 4

$-\log_{10}(\text{pvalue})$

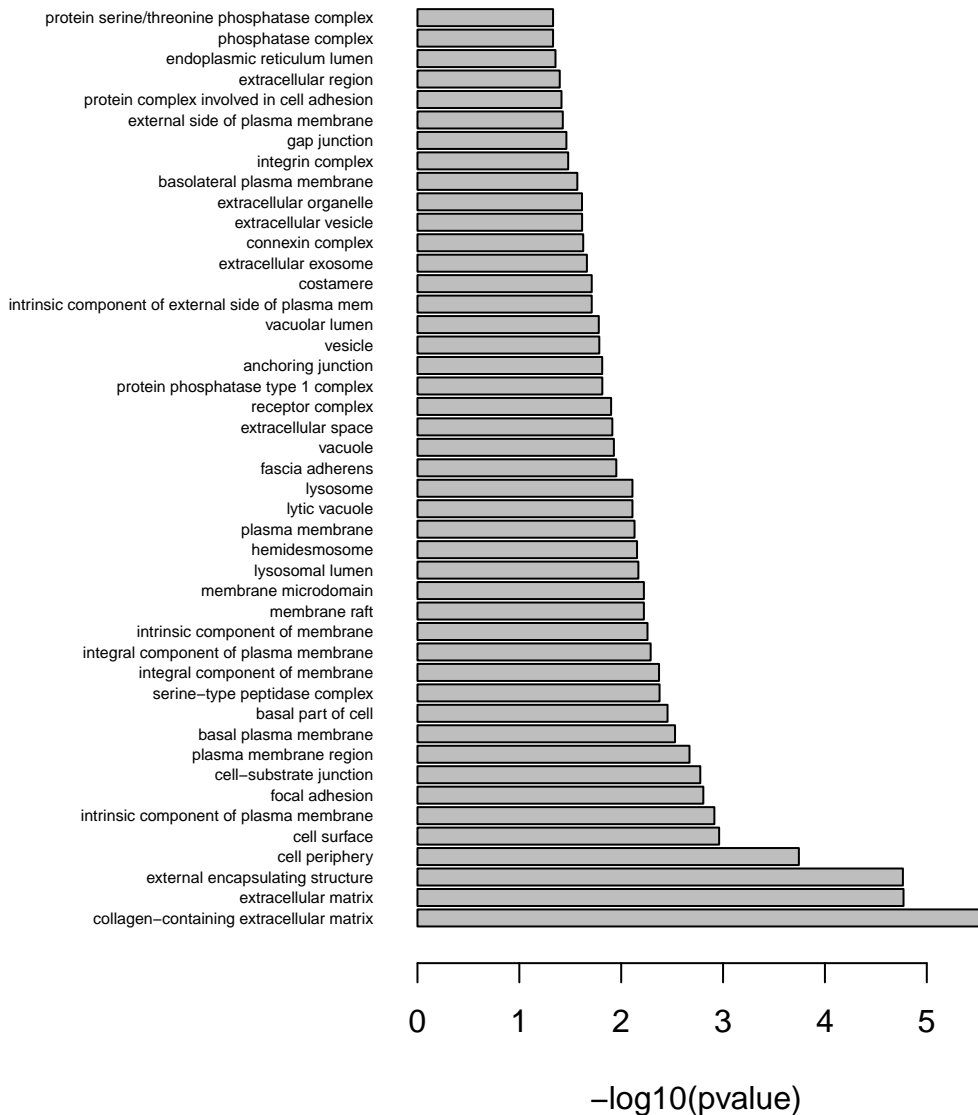

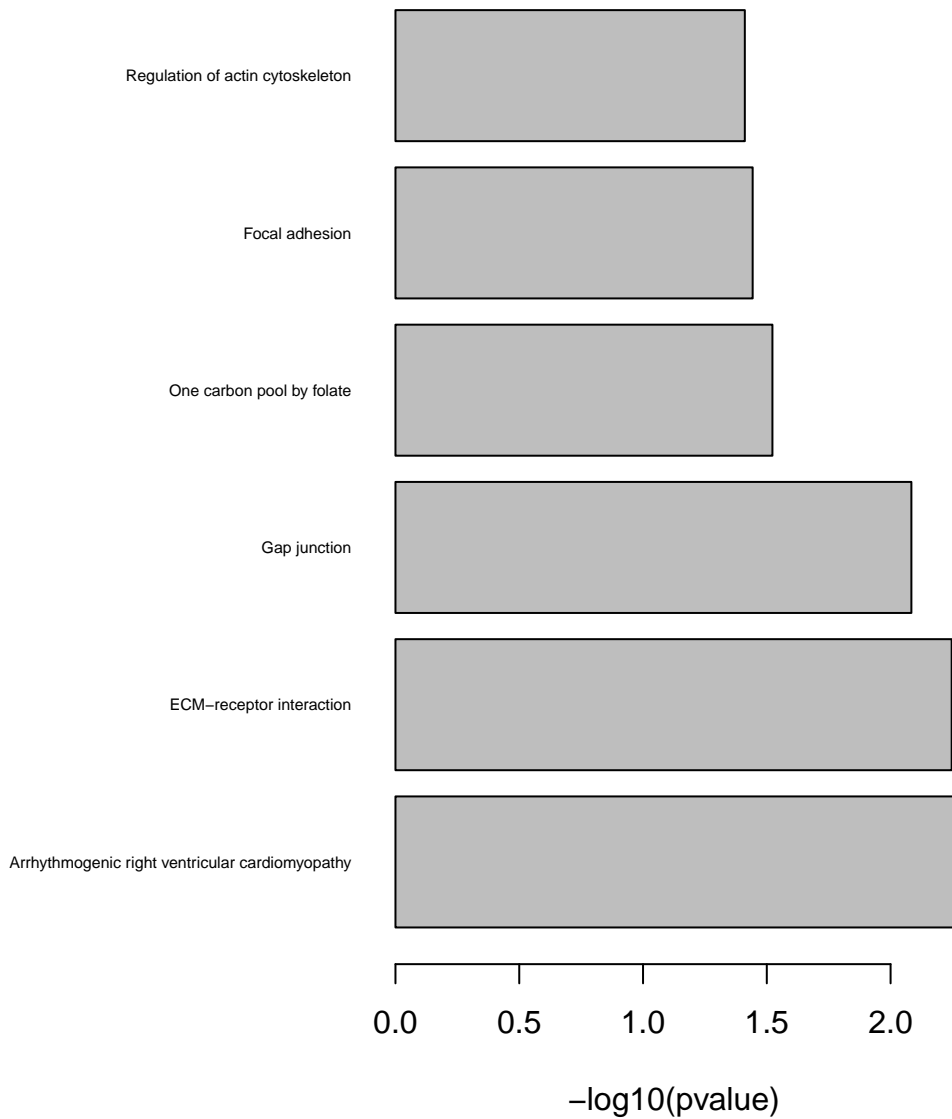

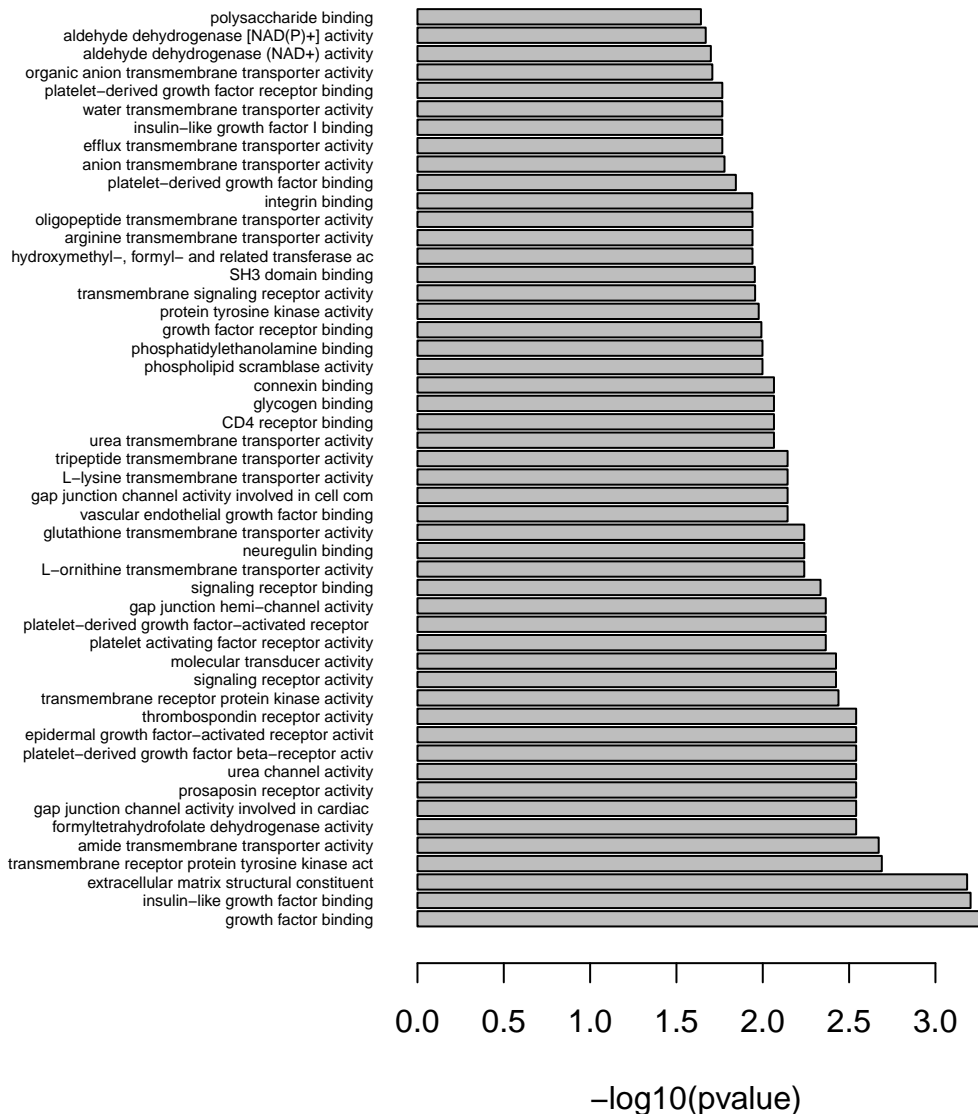

Supplement: Supplementary file 1 [file cells-11-01807-s001.zip › Supplementary_Data/DataS3/DataS3_cluster2.pdf]

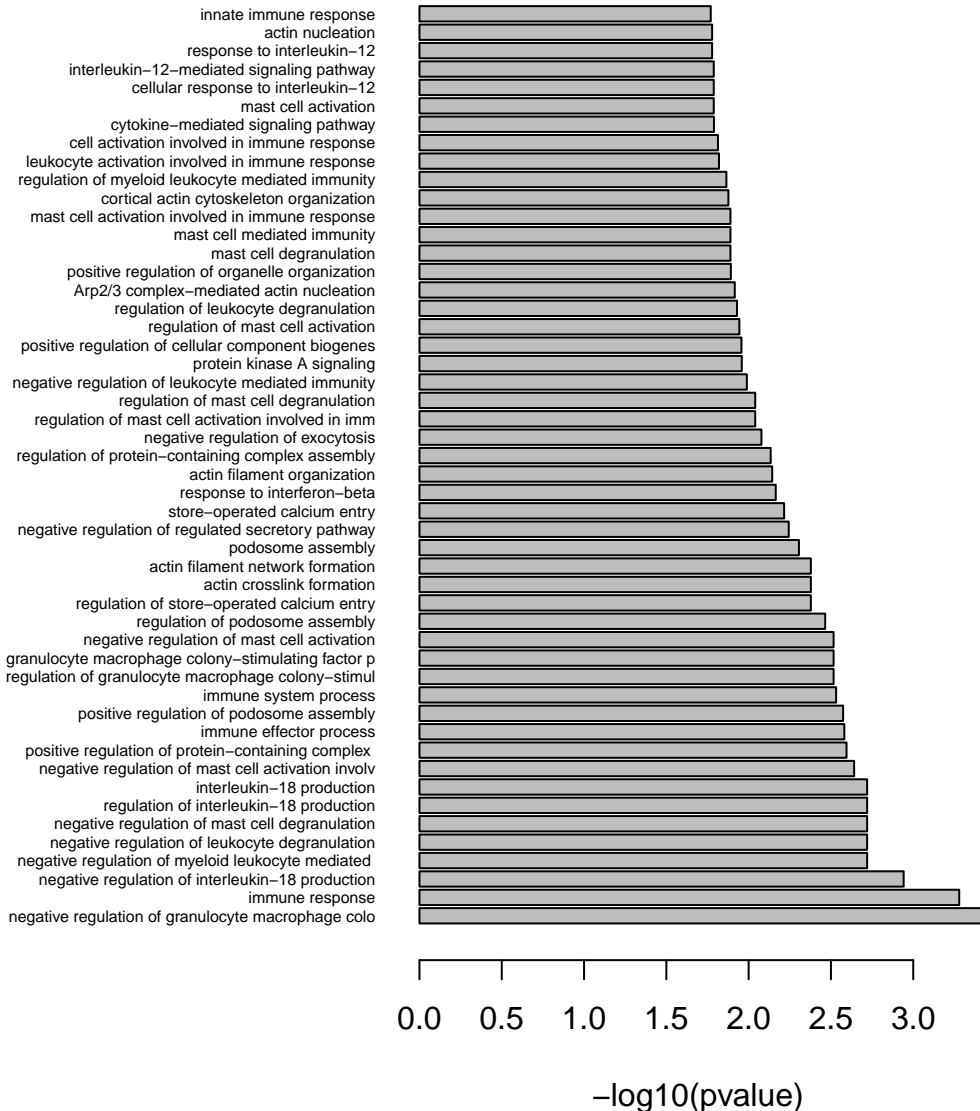

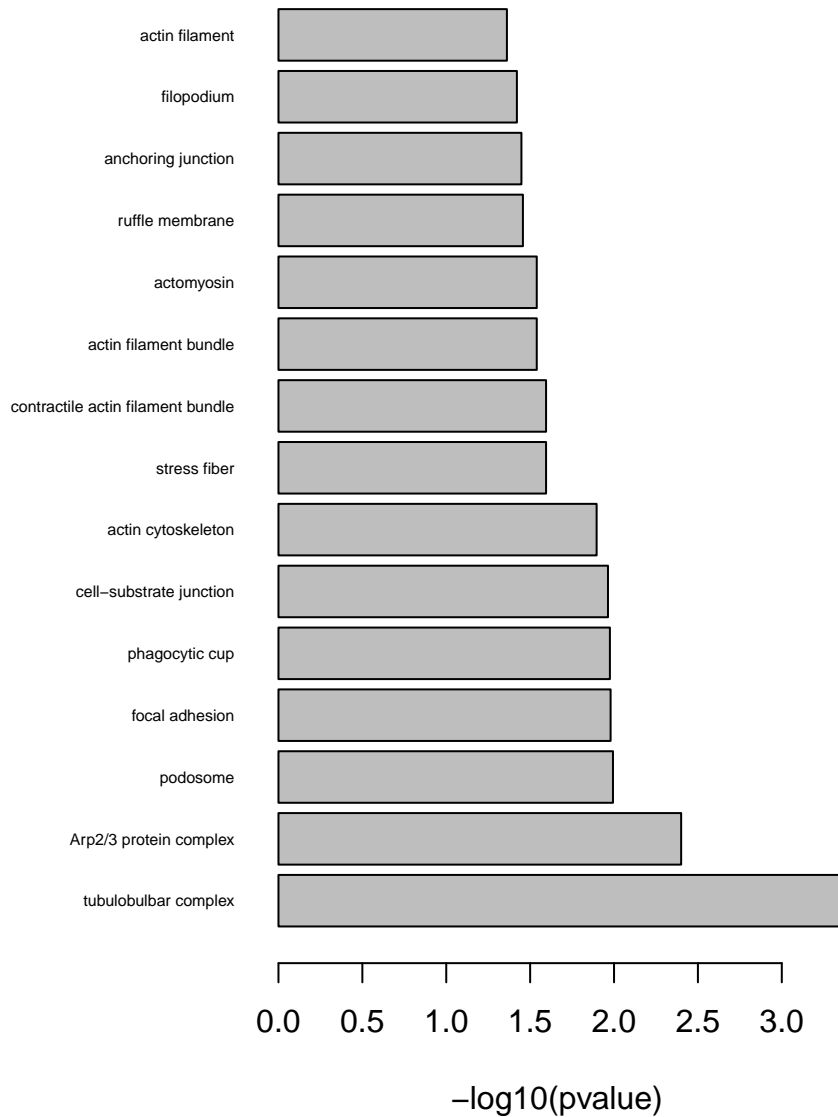

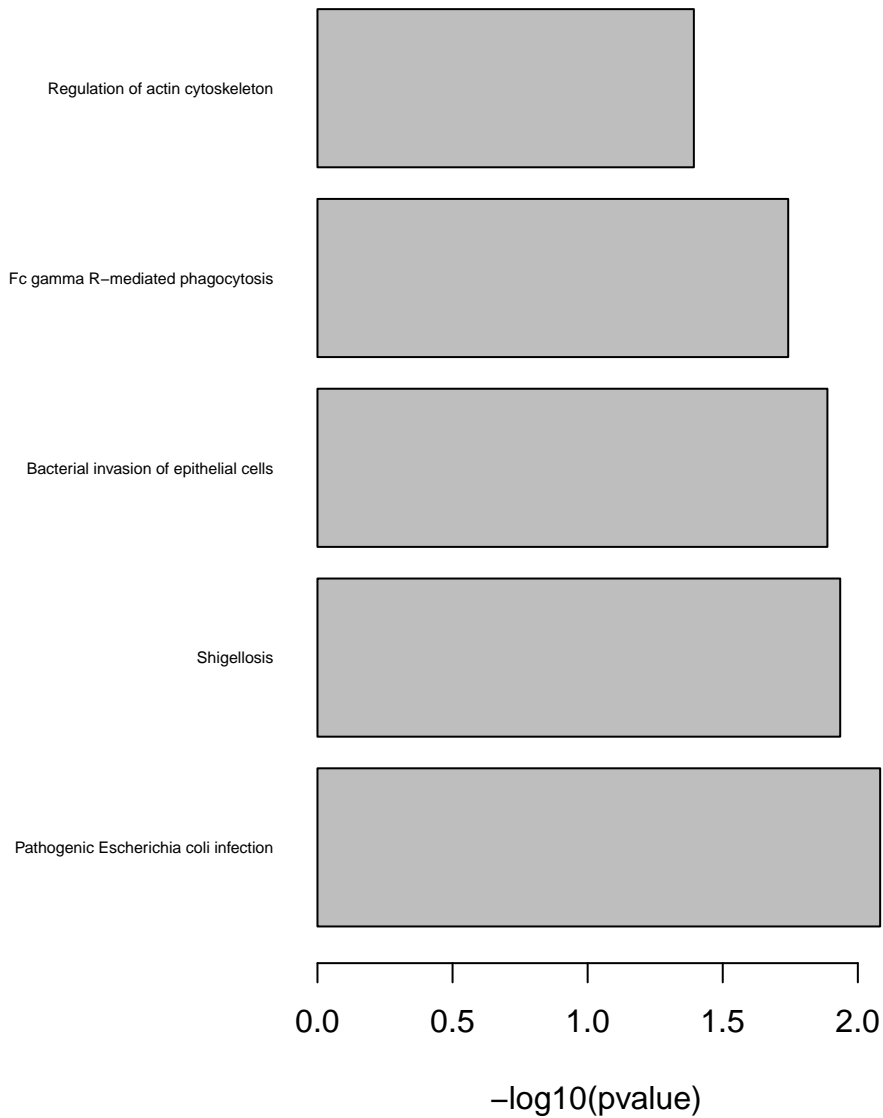

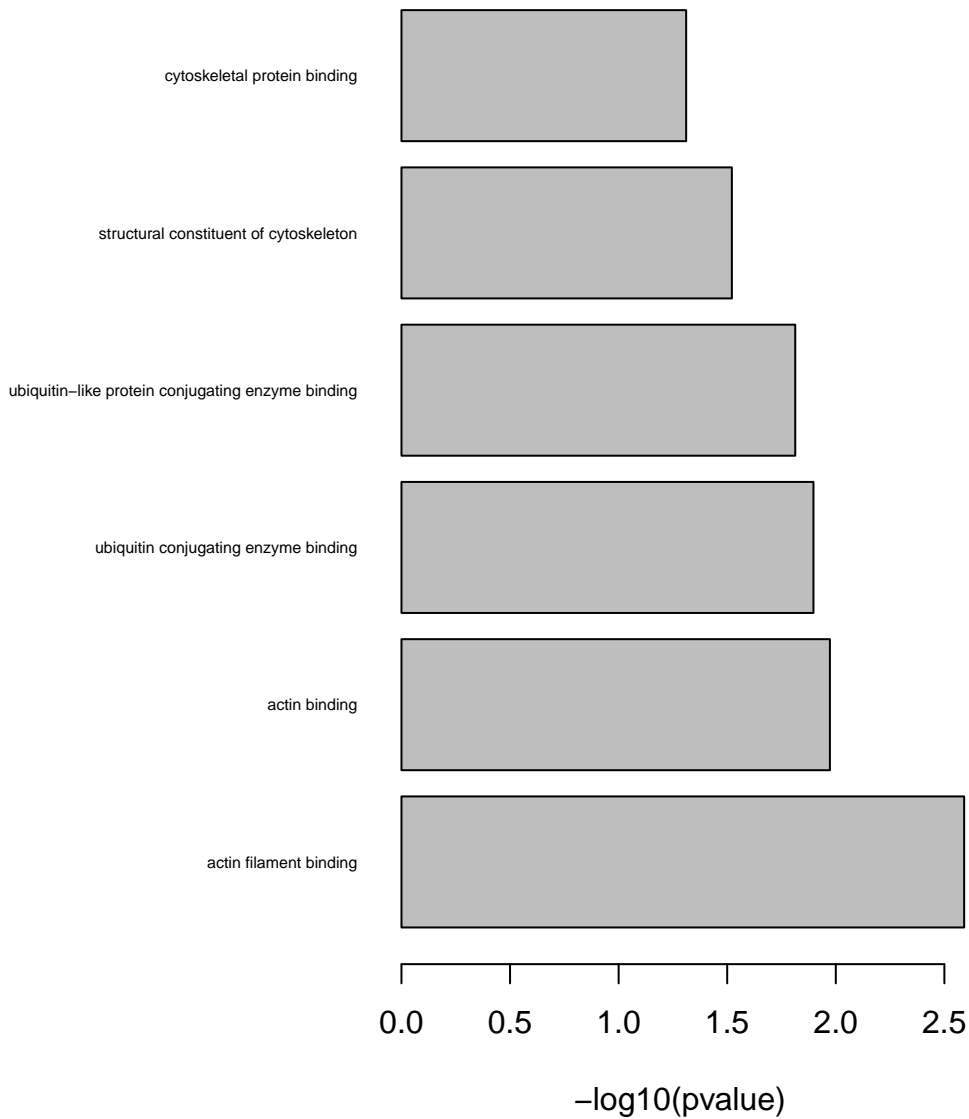

Supplement: Supplementary file 1 [file cells-11-01807-s001.zip › Supplementary_Data/DataS3/DataS3_cluster18.pdf]

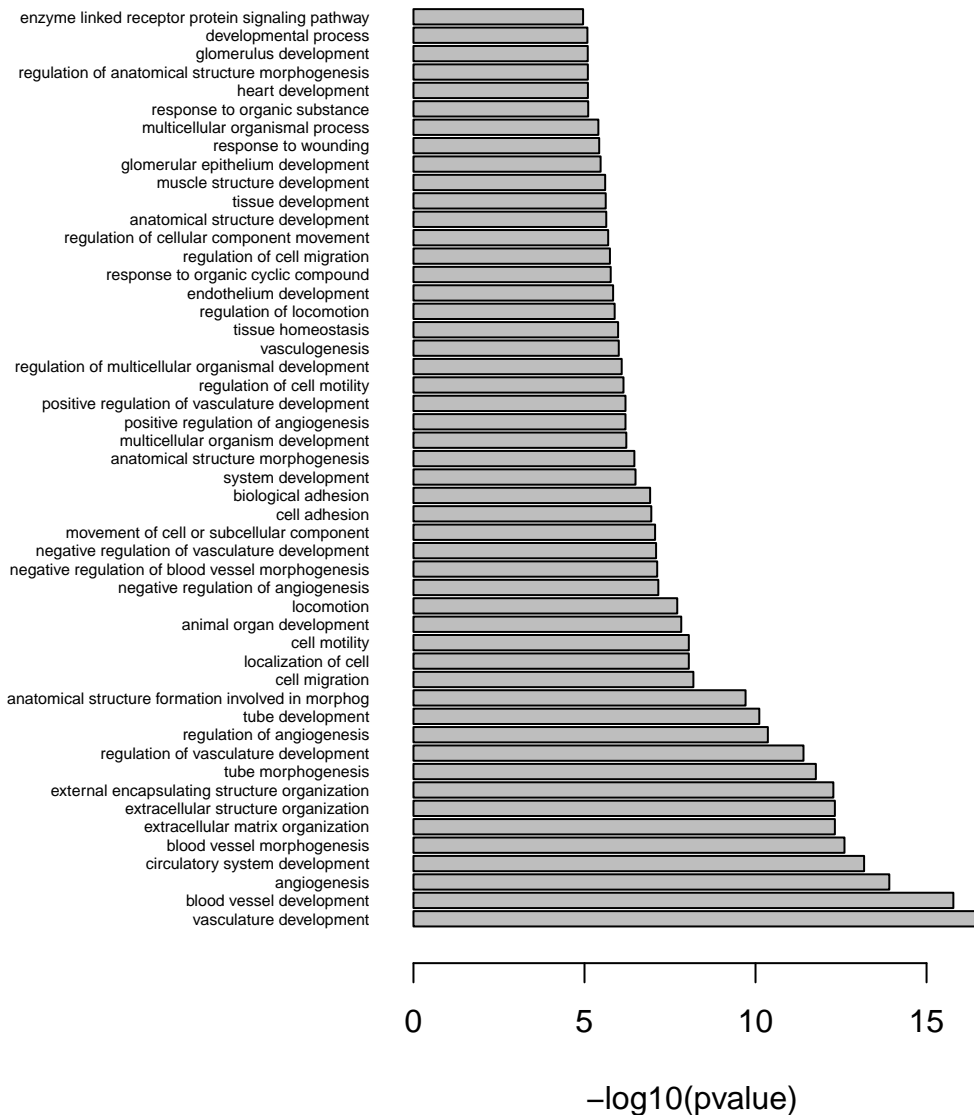

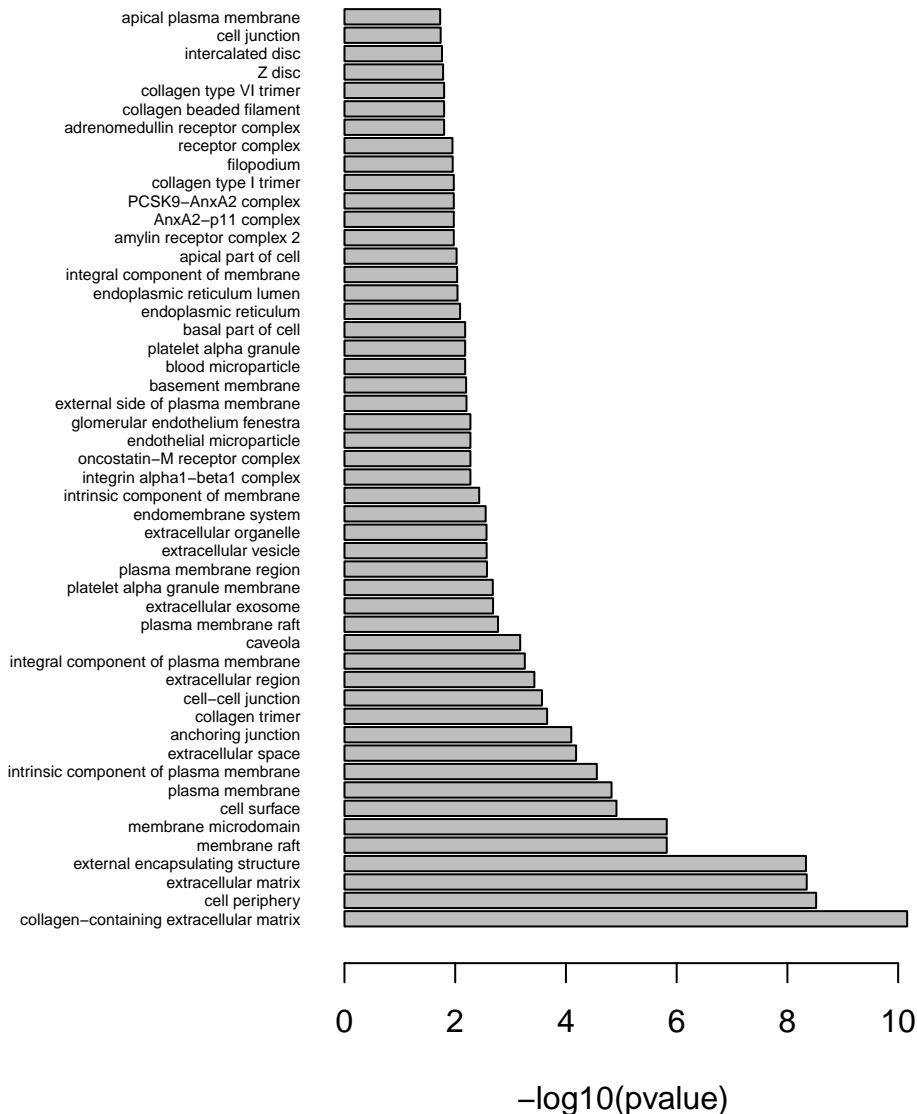

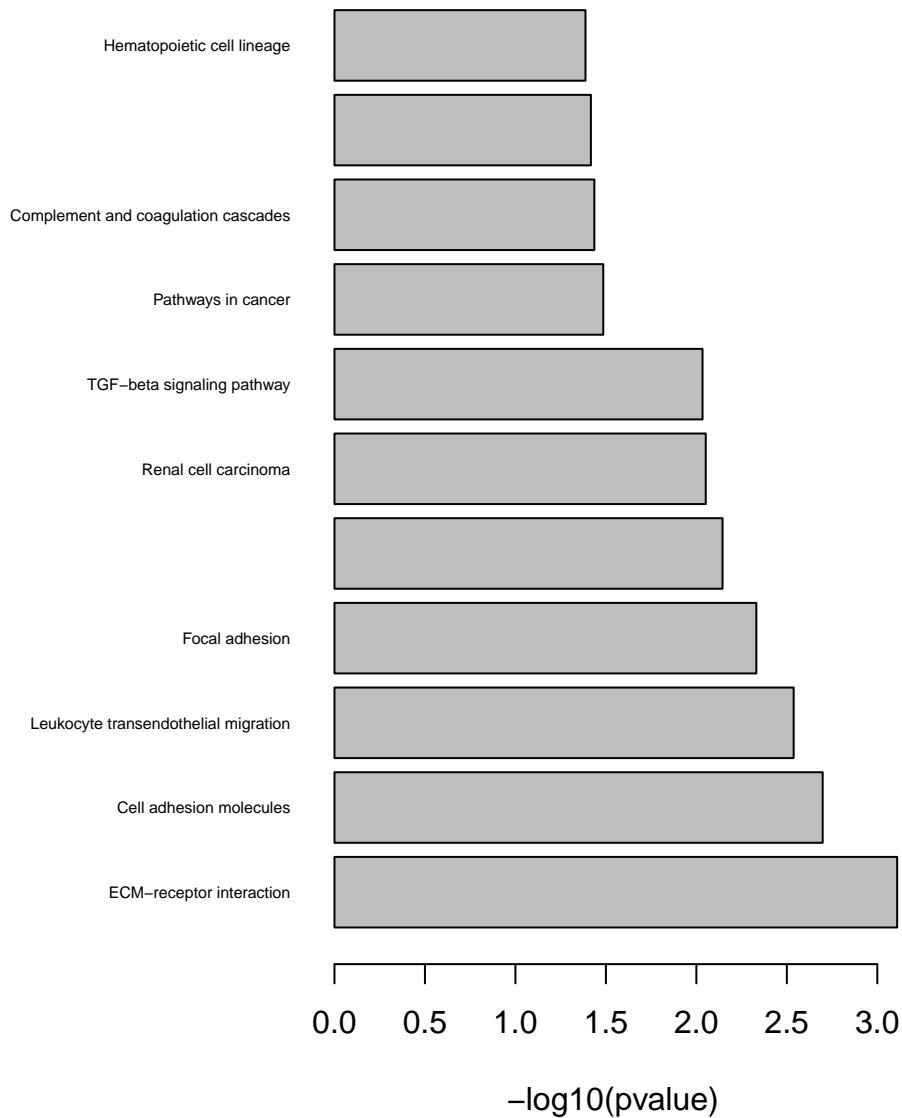

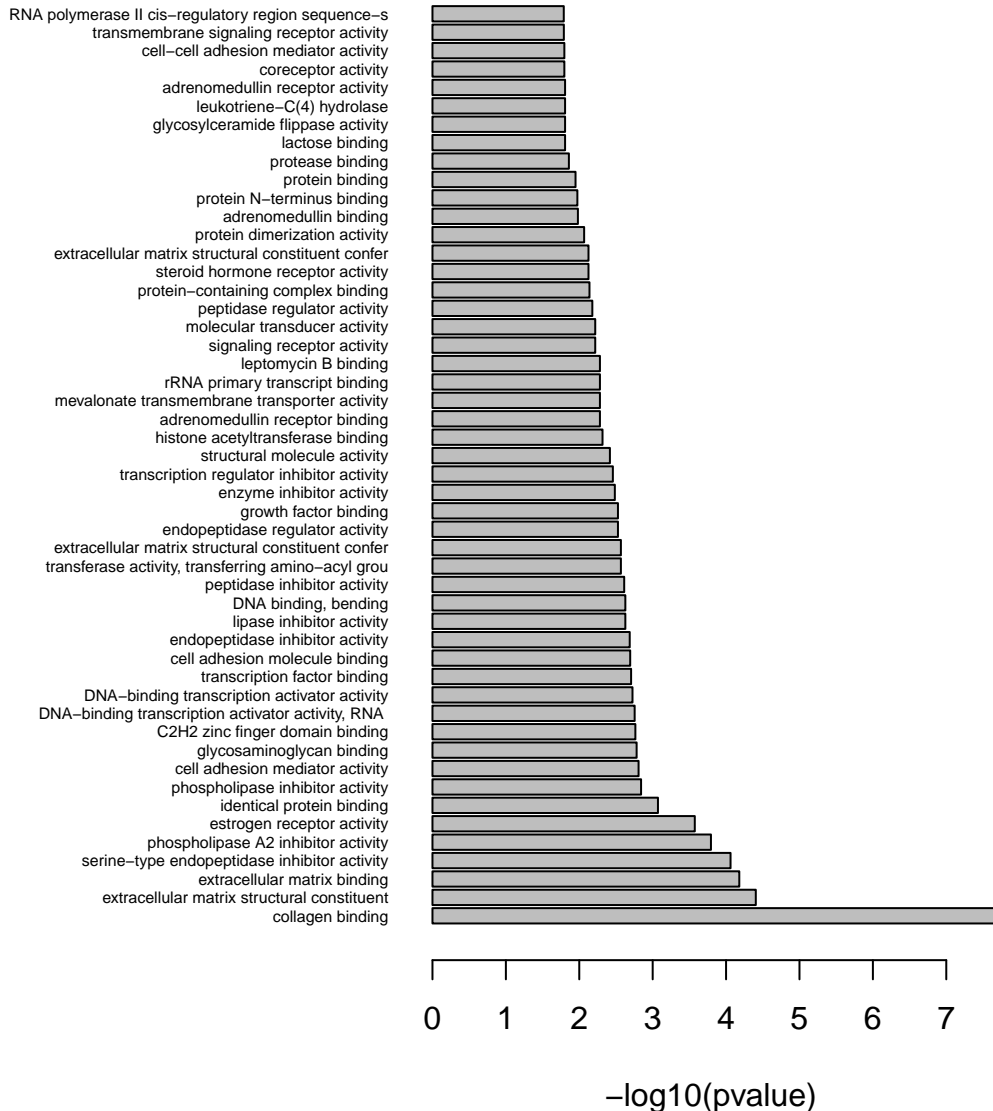

Supplement: Supplementary file 1 [file cells-11-01807-s001.zip › Supplementary_Data/DataS3/DataS3_cluster24.pdf]

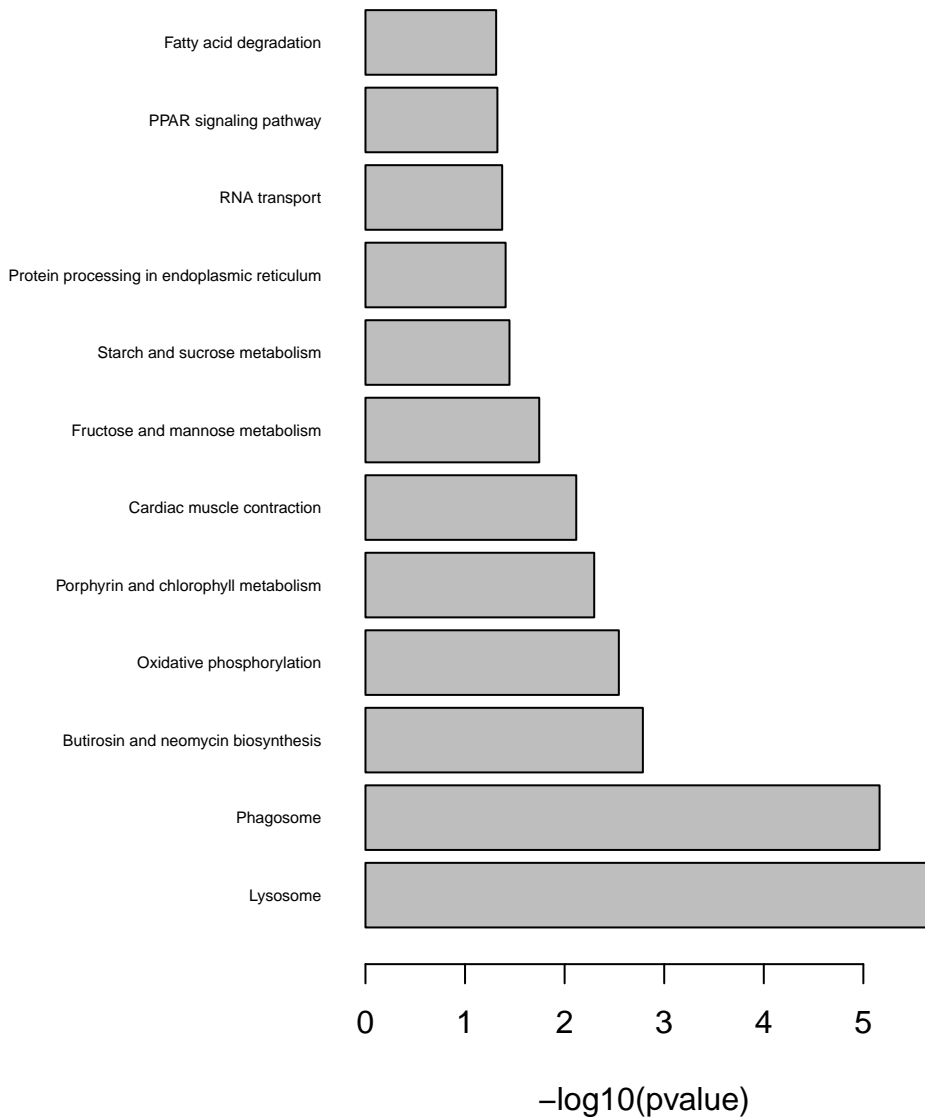

Supplement: Supplementary file 1 [file cells-11-01807-s001.zip › Supplementary_Data/DataS9/DataS9_pVal_AB42_Im_vs_PBS_Im_kegg_Up.pdf]

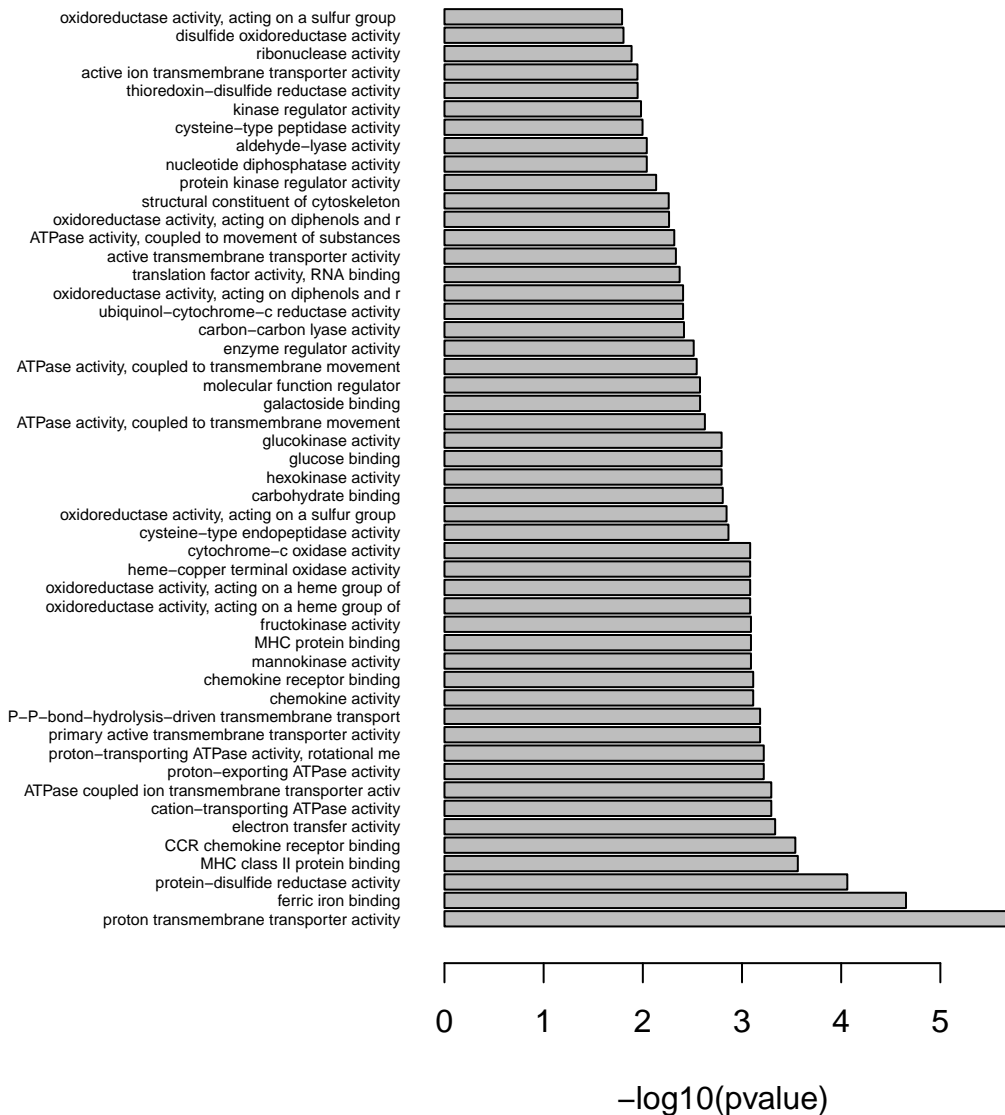

Supplement: Supplementary file 1 [file cells-11-01807-s001.zip › Supplementary_Data/DataS9/DataS9_pVal_AB42_Im_vs_PBS_Im_MF_Up.pdf]

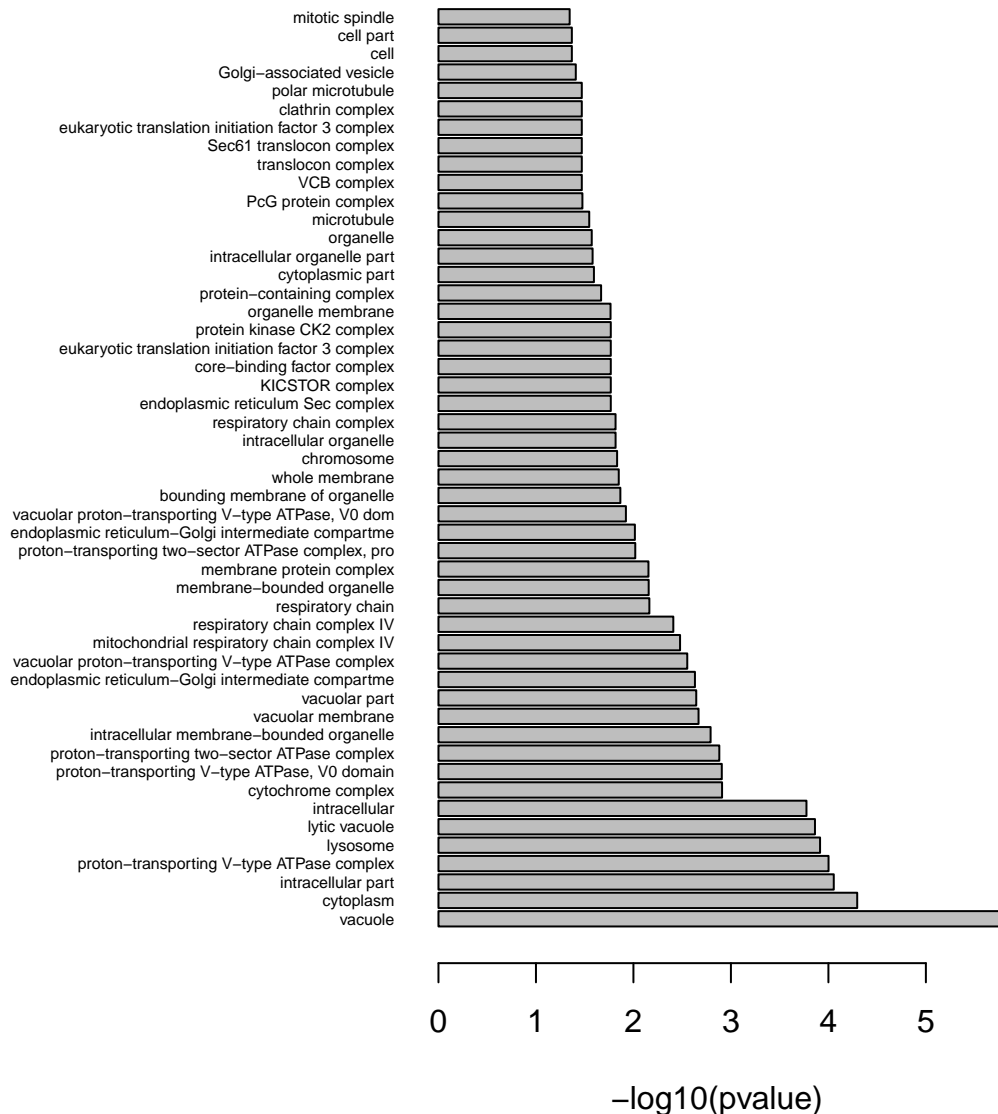

Supplement: Supplementary file 1 [file cells-11-01807-s001.zip › Supplementary_Data/DataS9/DataS9_pVal_AB42_Im_vs_PBS_Im_CC_Up.pdf]

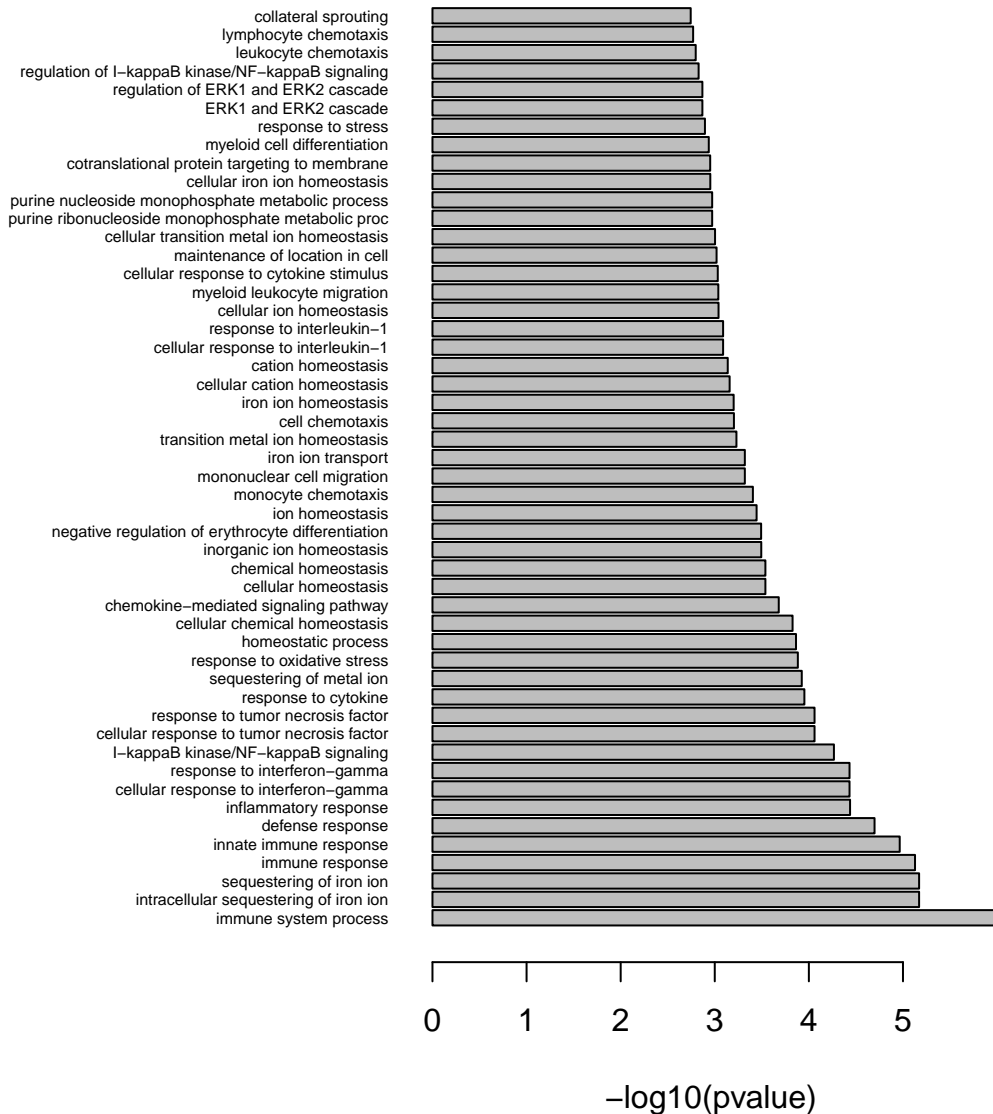

Supplement: Supplementary file 1 [file cells-11-01807-s001.zip › Supplementary_Data/DataS9/DataS9_pVal_AB42_Im_vs_PBS_Im_BP_Up.pdf]

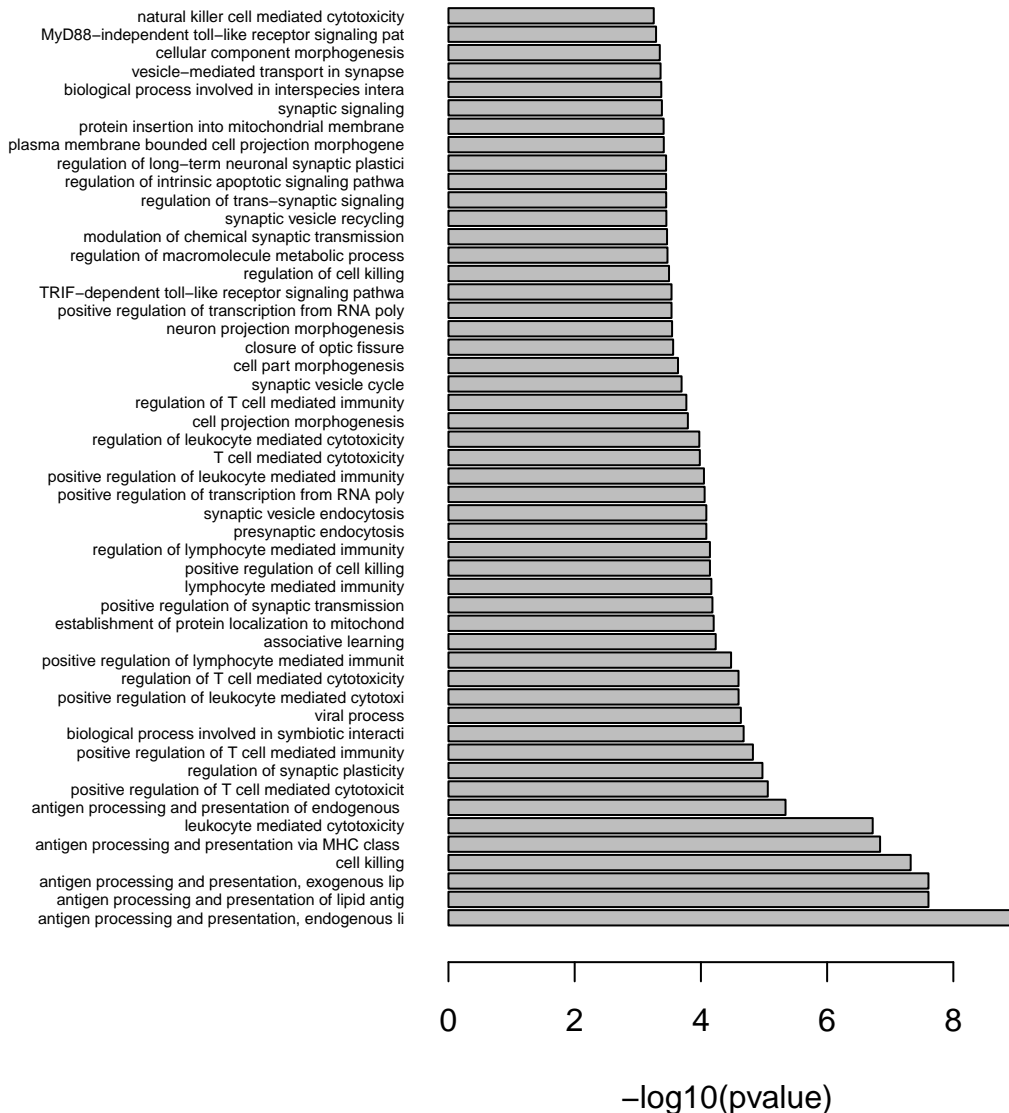

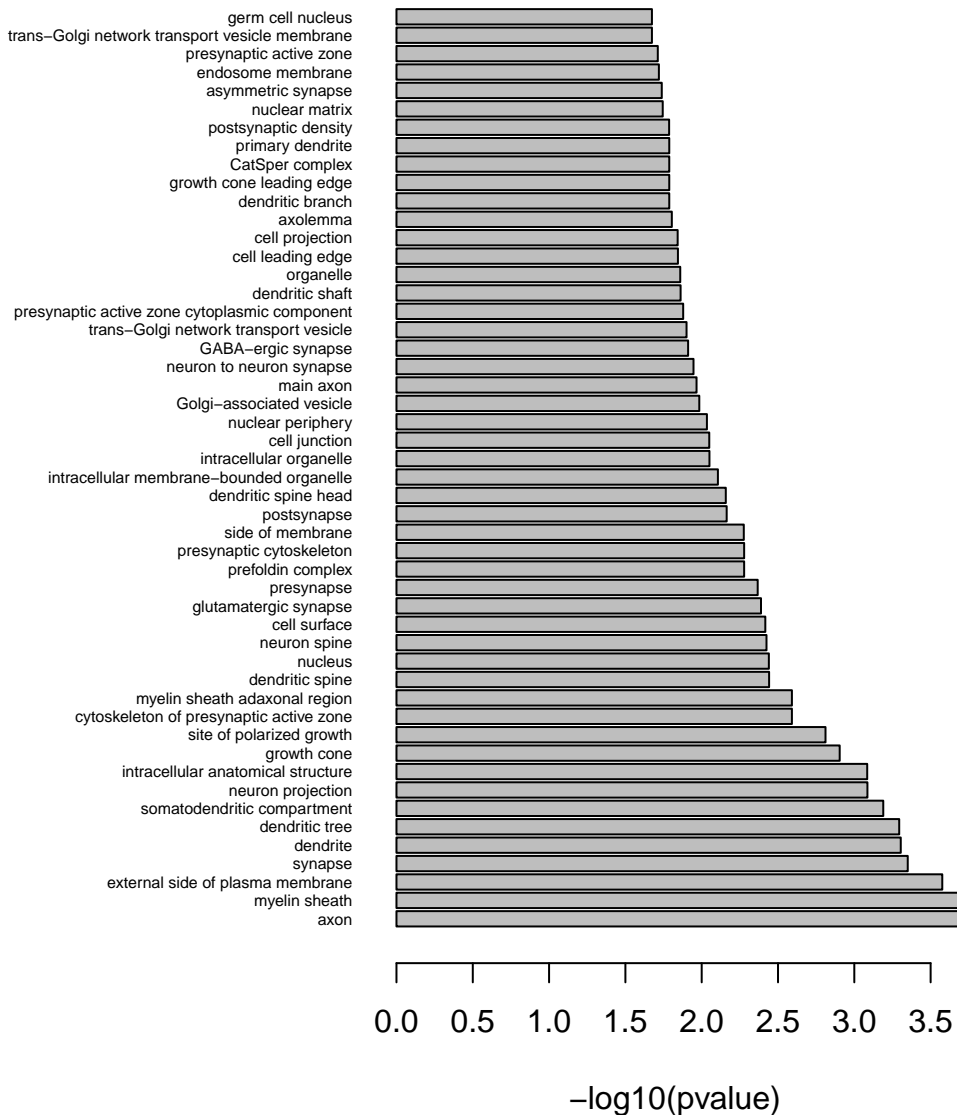

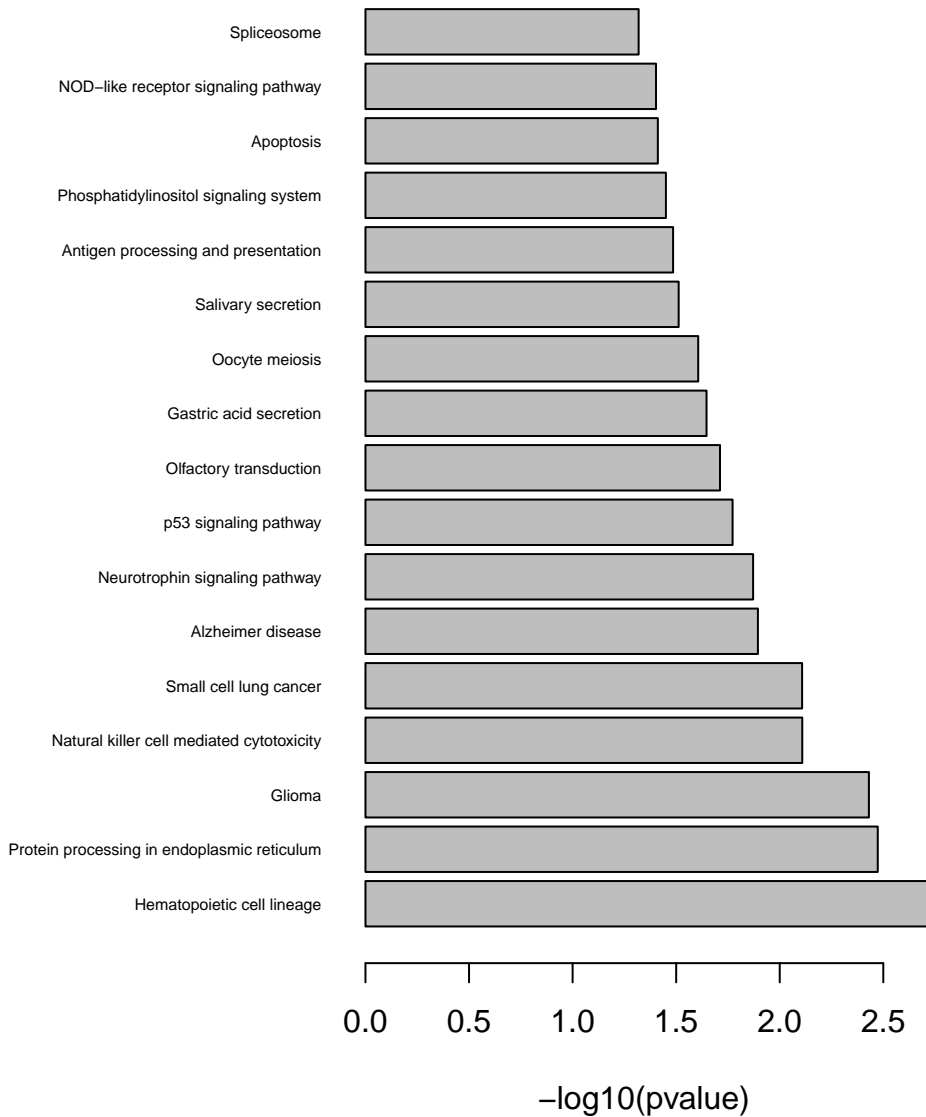

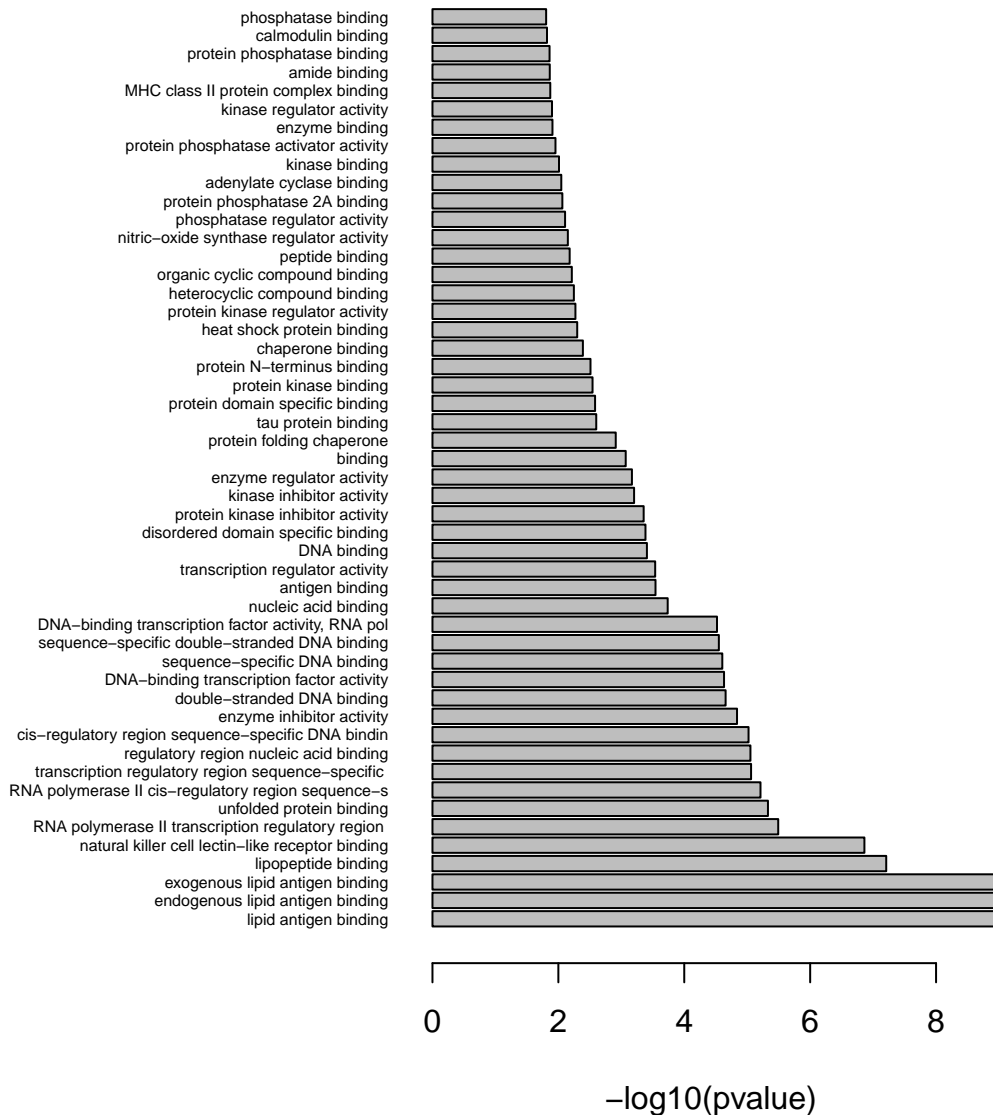

Supplement: Supplementary file 1 [file cells-11-01807-s001.zip › Supplementary_Data/DataS5/dre_cells_GOKEGGs/ab_vs_pbs_cluster14.pdf]

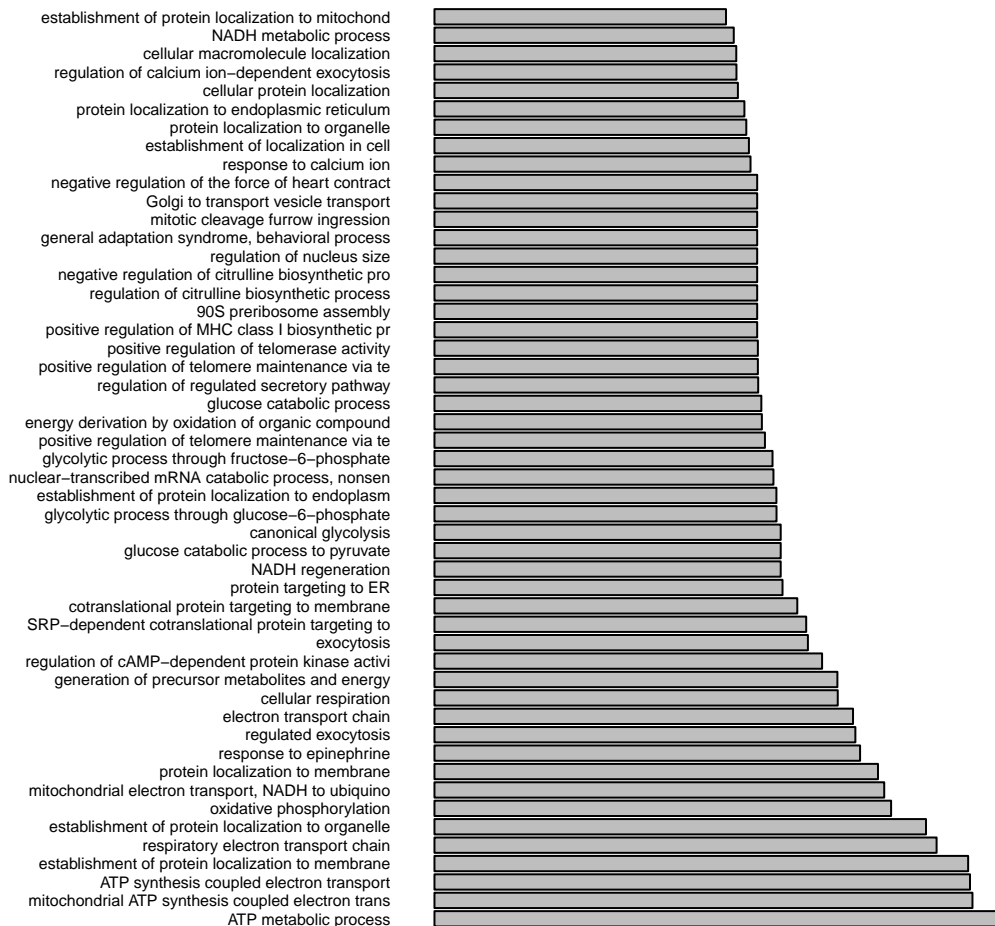

0 1 2 3 4

$-\log_{10}(\text{pvalue})$

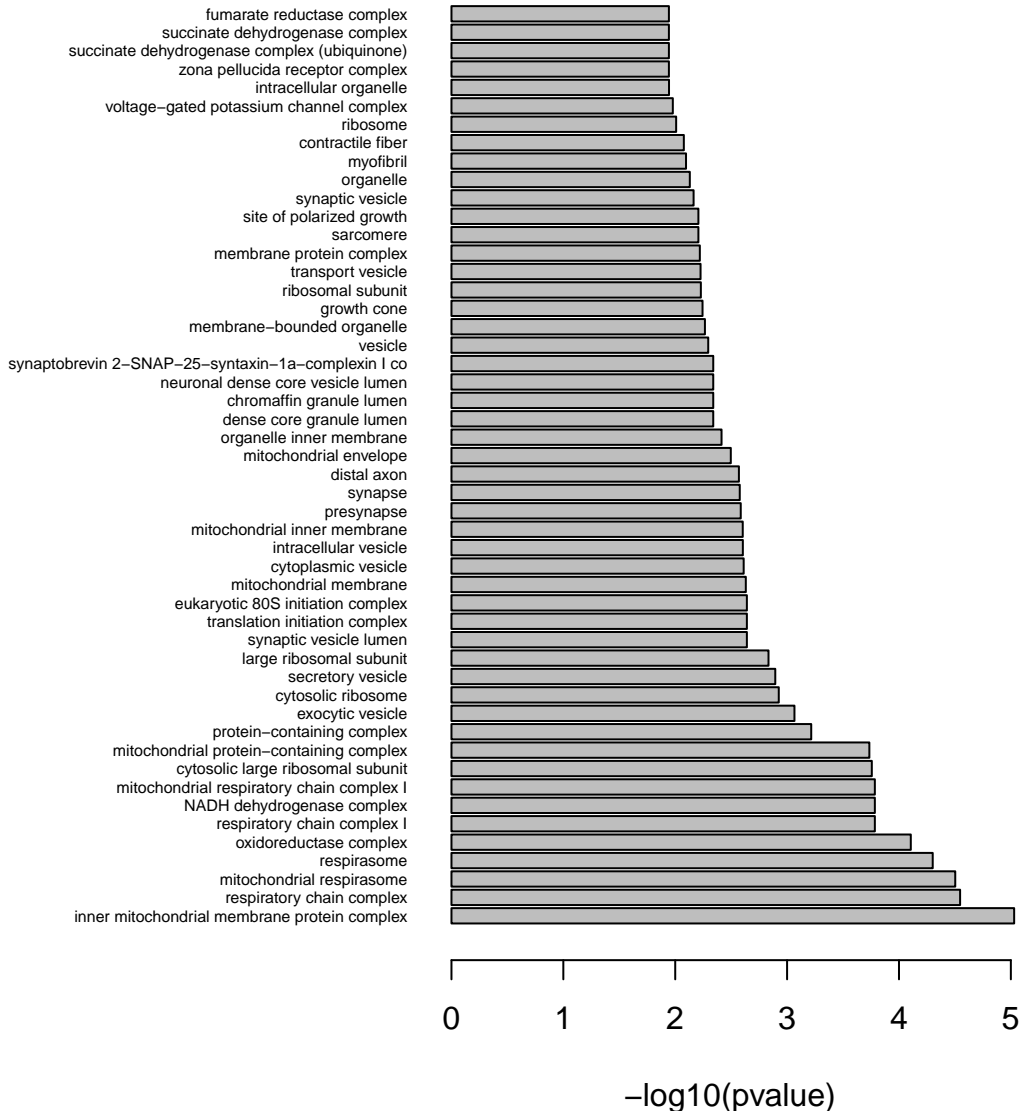

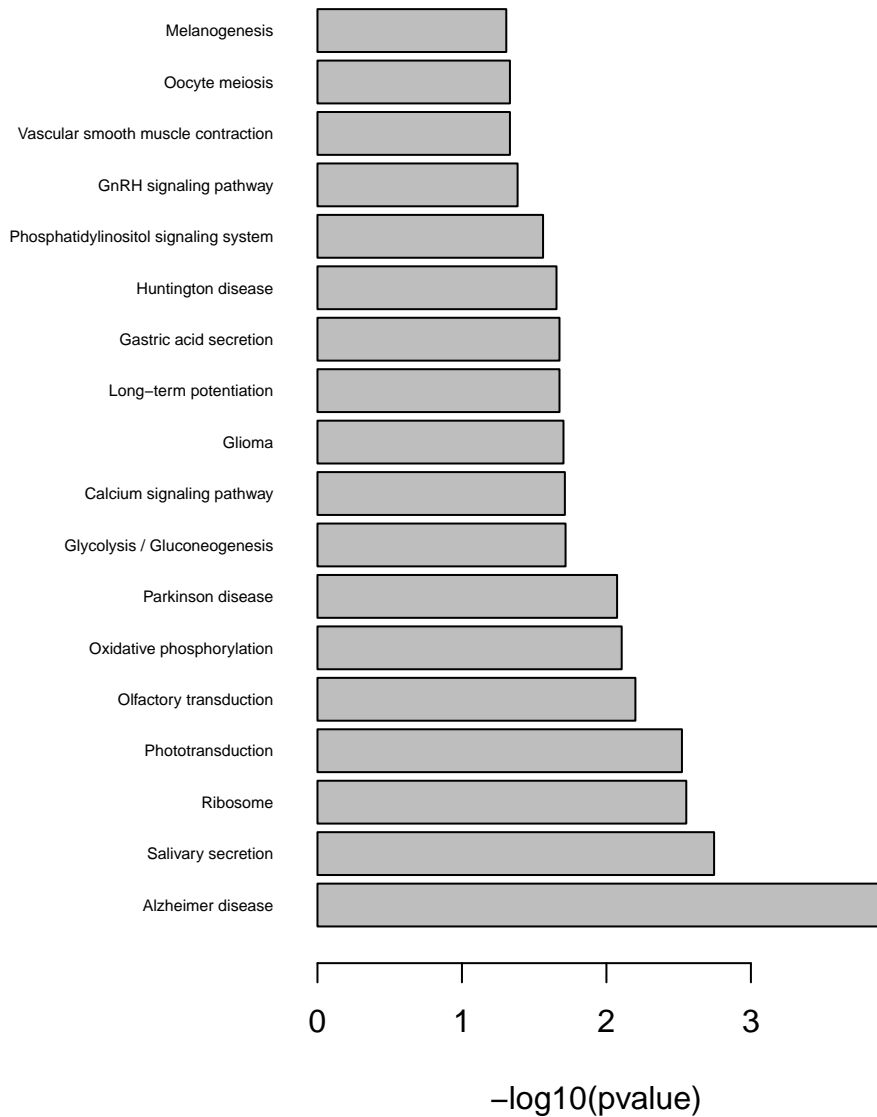

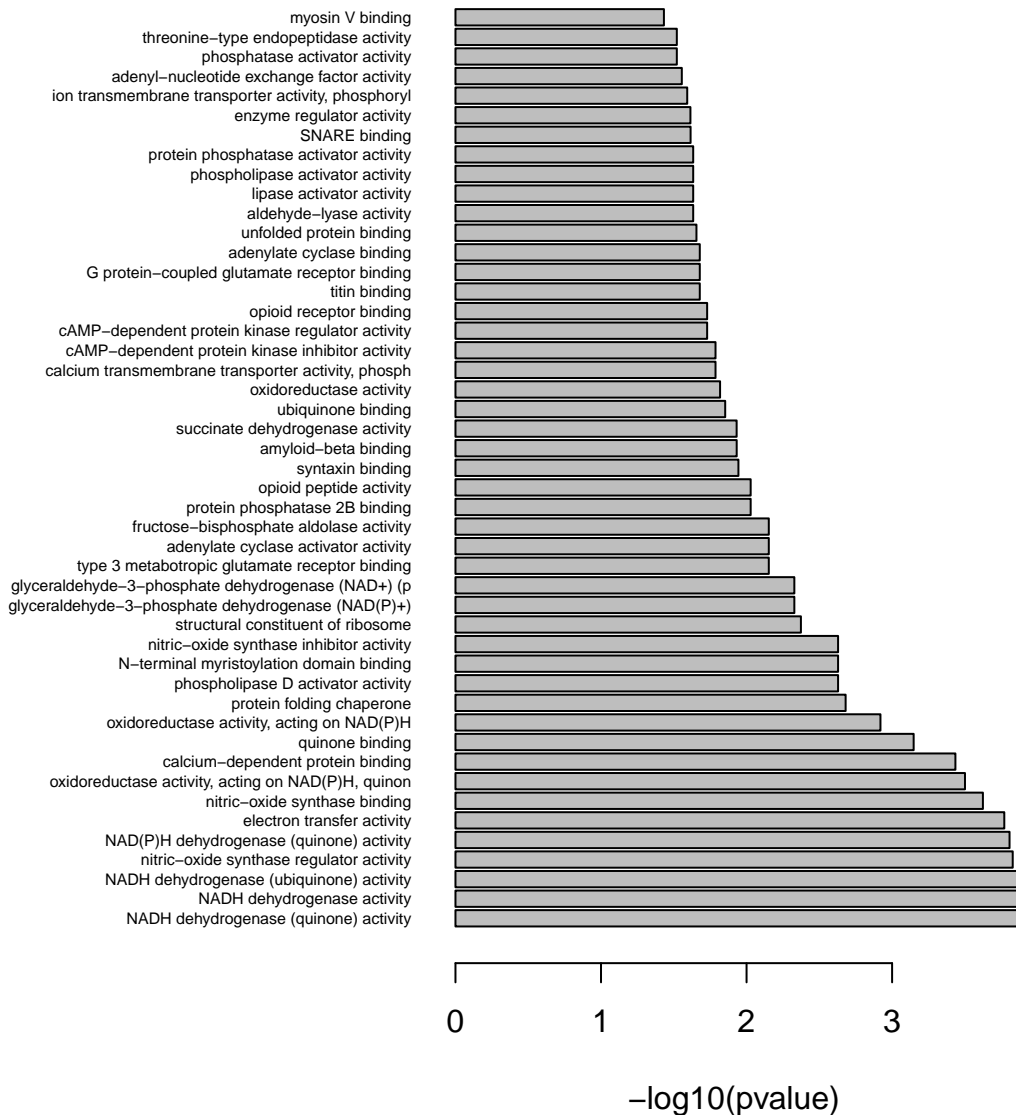

Supplement: Supplementary file 1 [file cells-11-01807-s001.zip › Supplementary_Data/DataS5/dre_cells_GOKEGGs/ab_vs_pbs_cluster12.pdf]

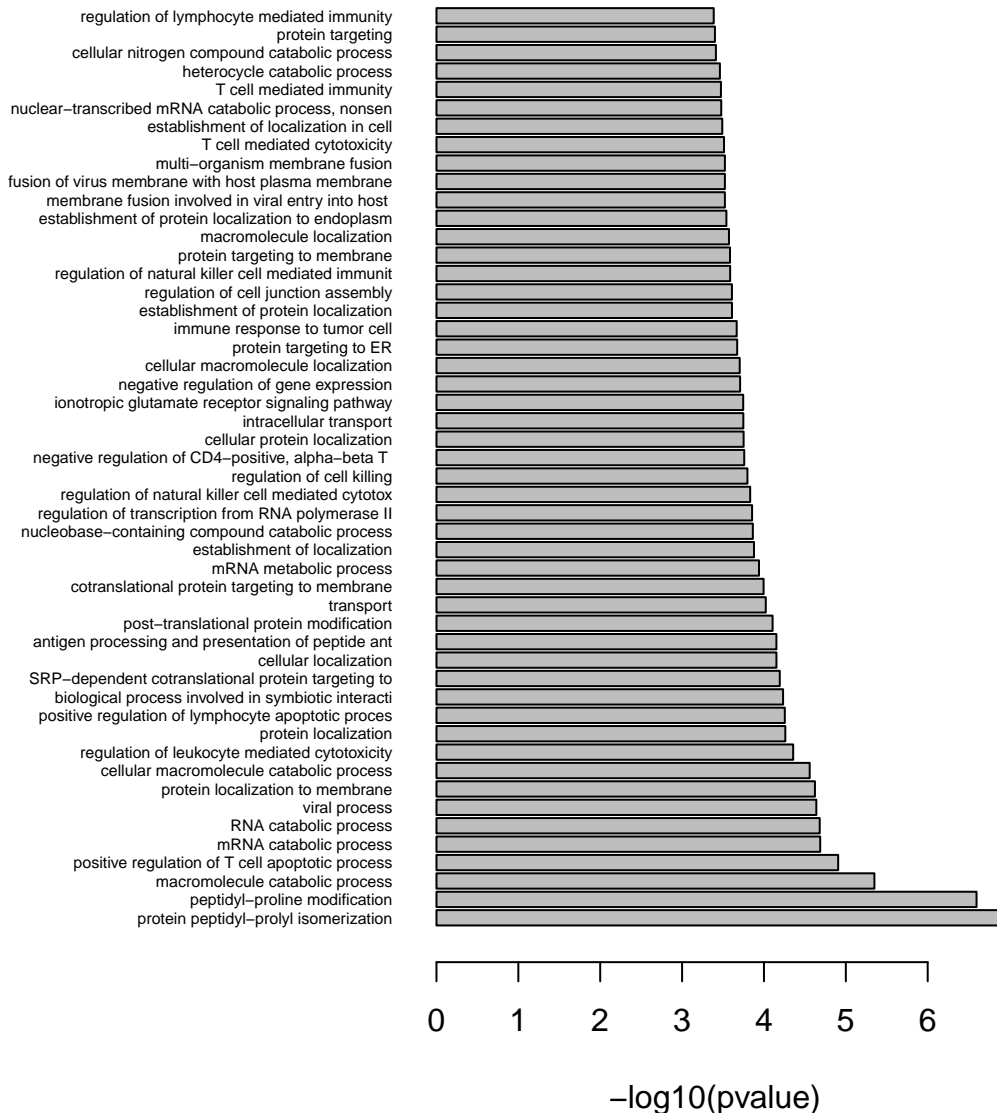

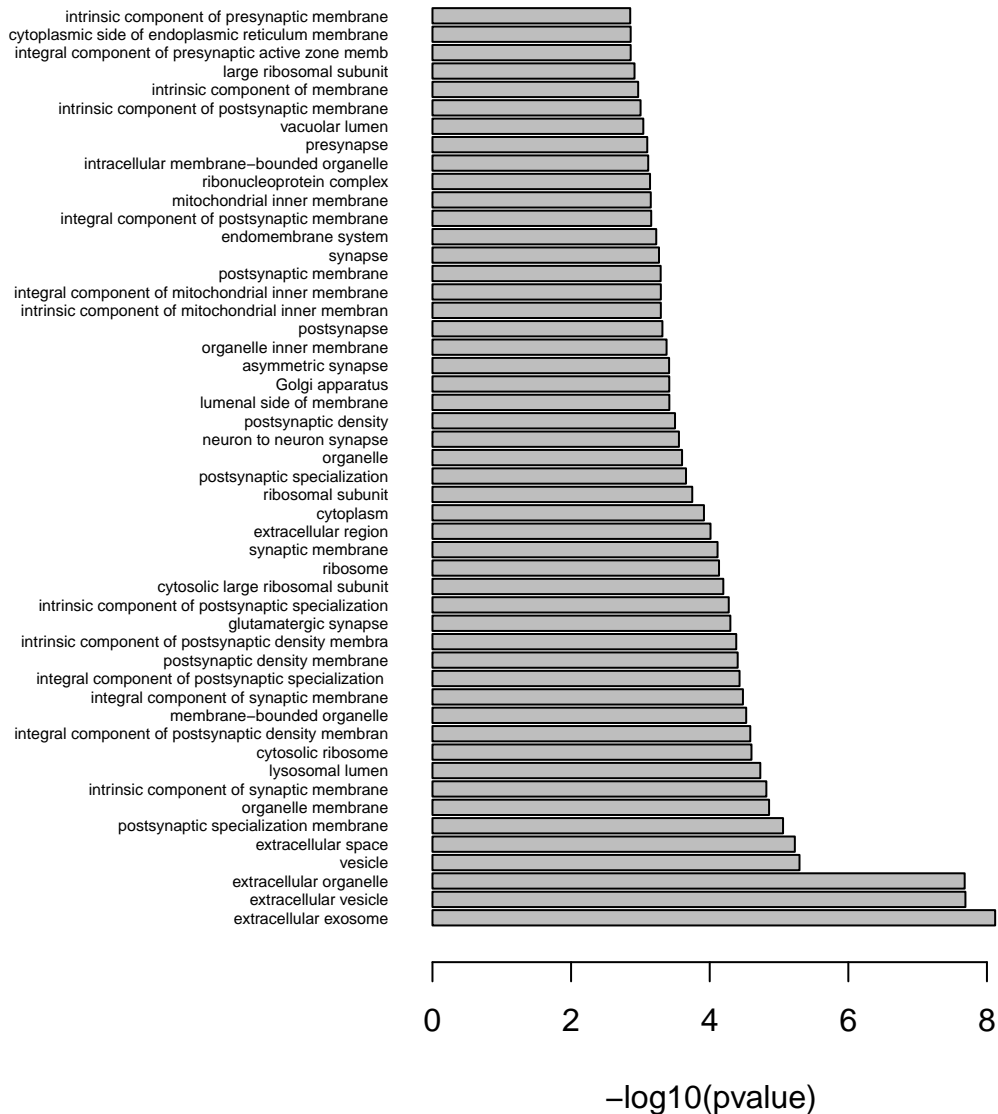

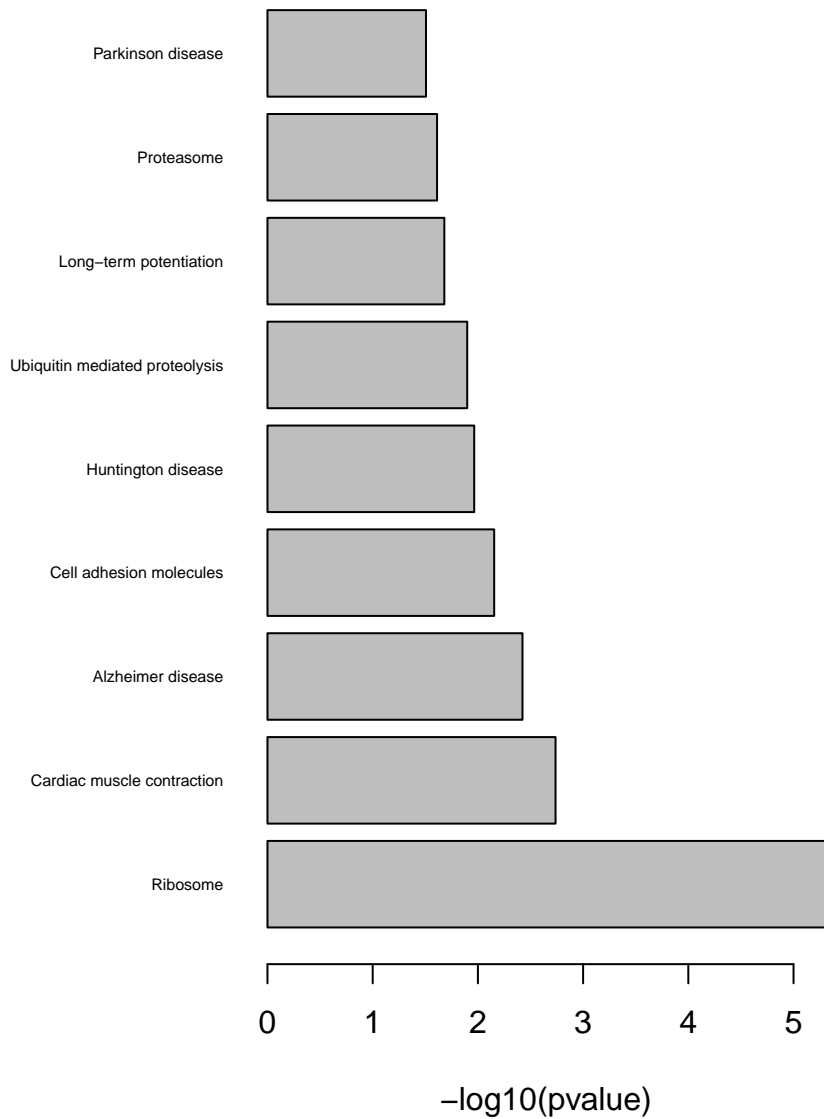

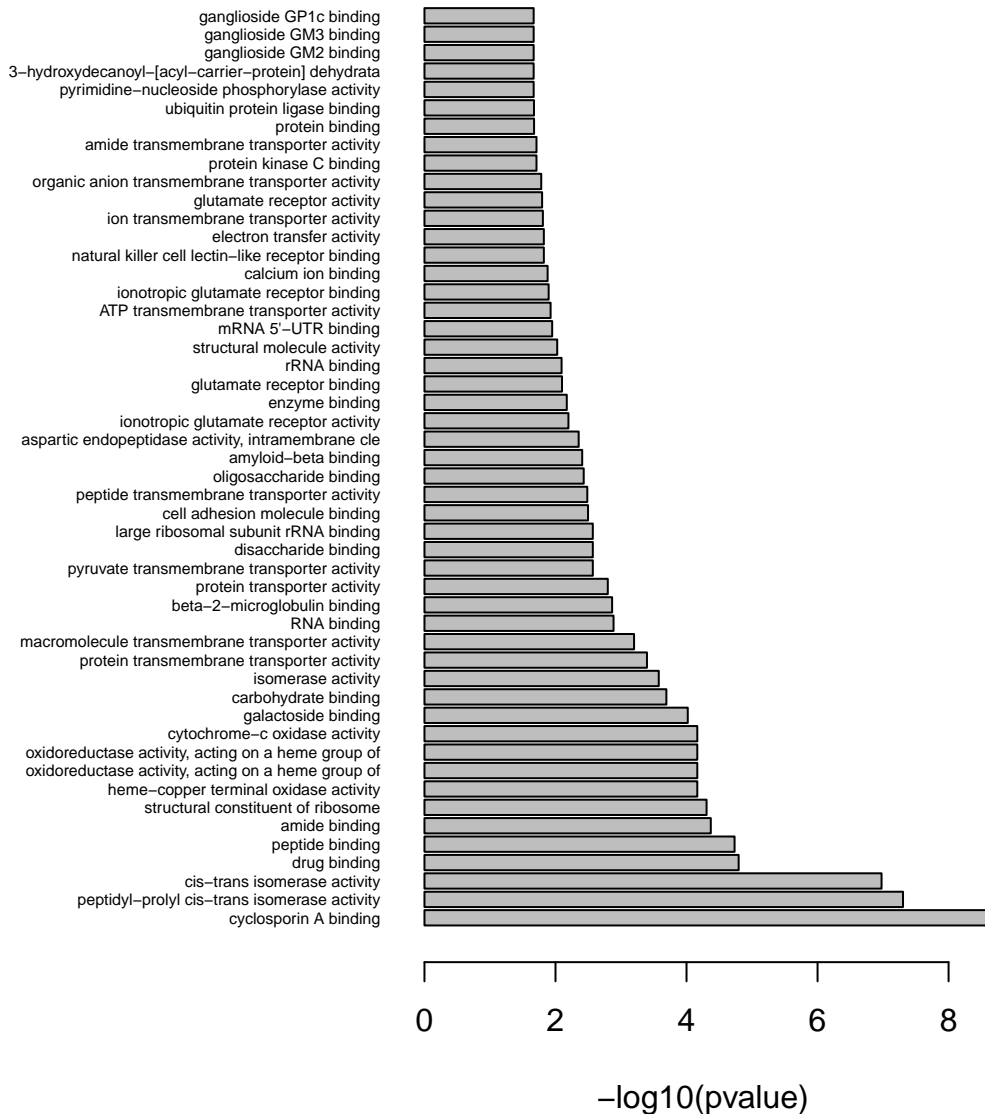

Supplement: Supplementary file 1 [file cells-11-01807-s001.zip › Supplementary_Data/DataS5/dre_cells_GOKEGGs/ab_vs_pbs_cluster6.pdf]

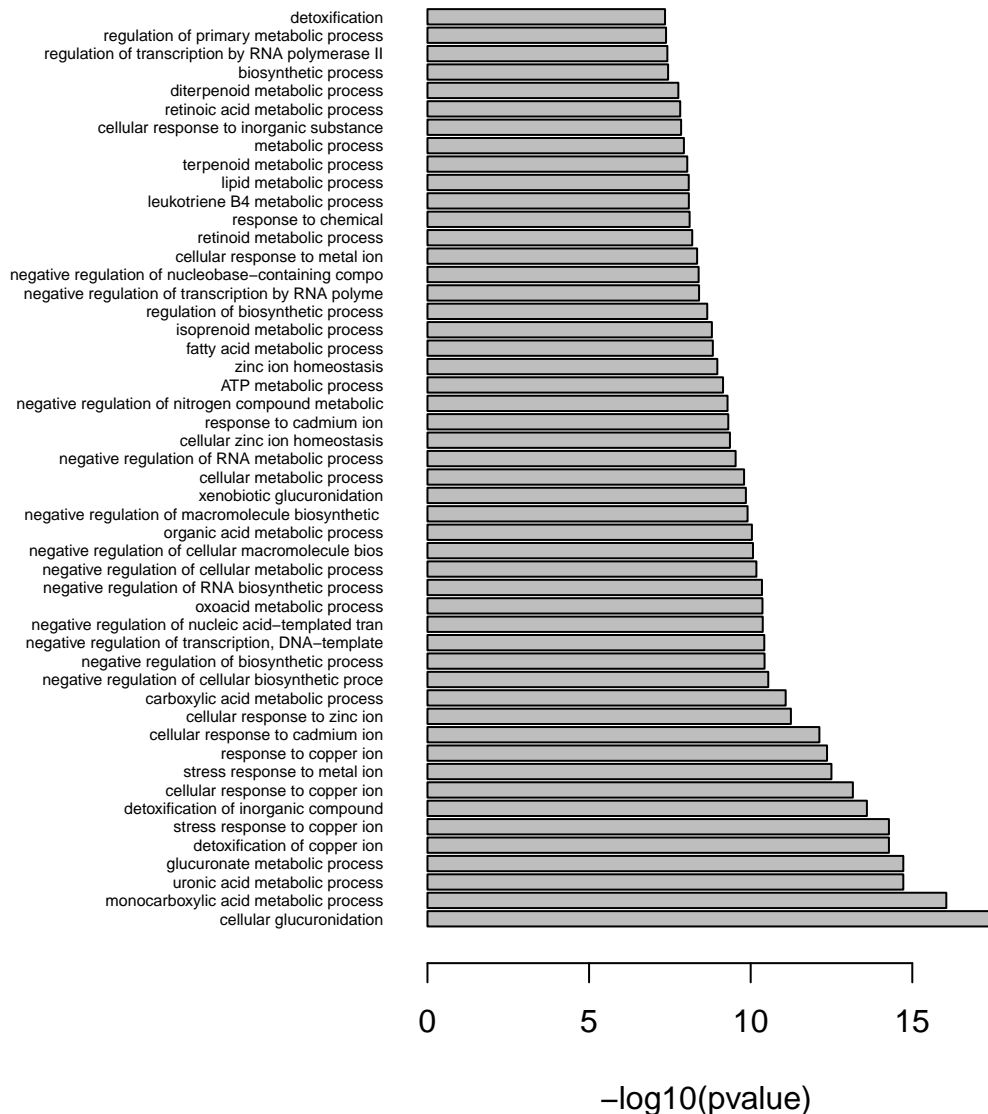

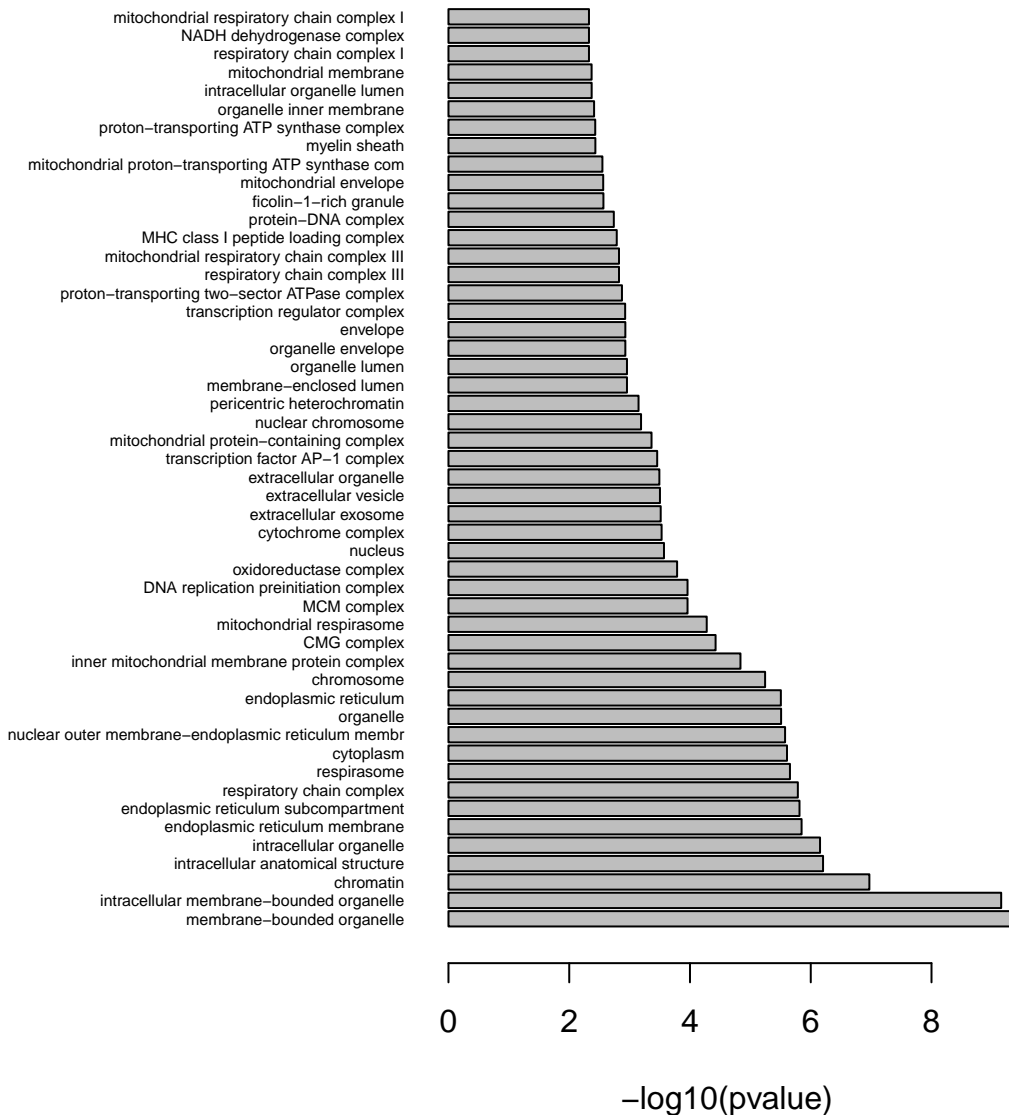

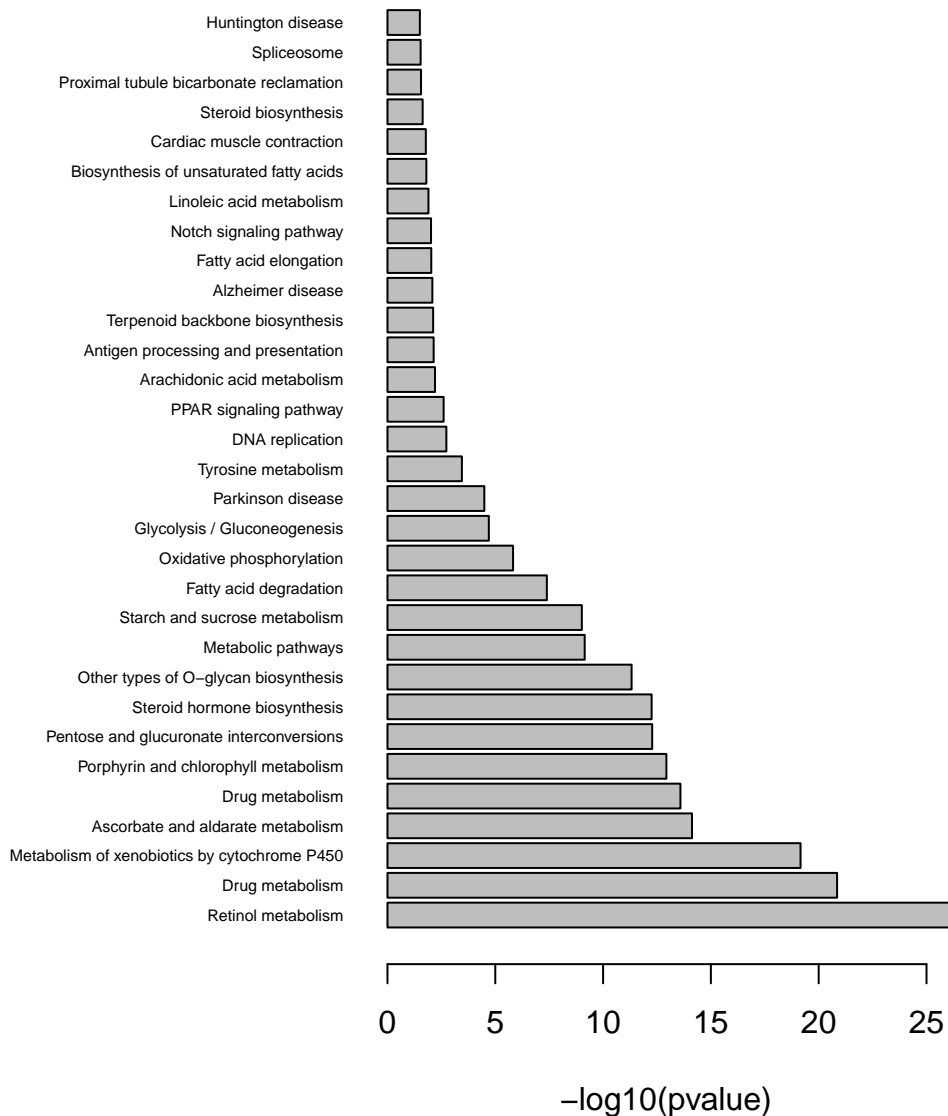

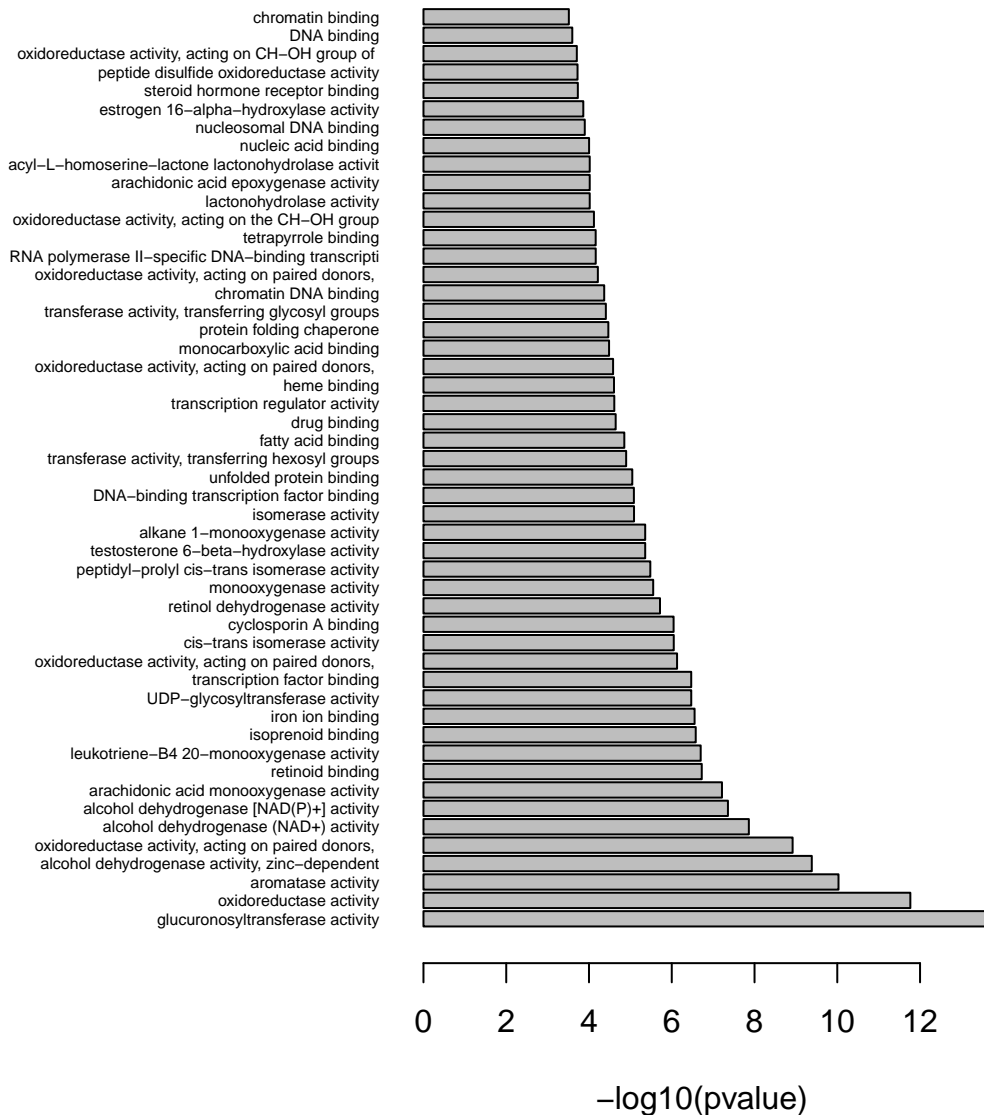

Supplement: Supplementary file 1 [file cells-11-01807-s001.zip › Supplementary_Data/DataS5/dre_cells_GOKEGGs/ab_vs_pbs_cluster4.pdf]

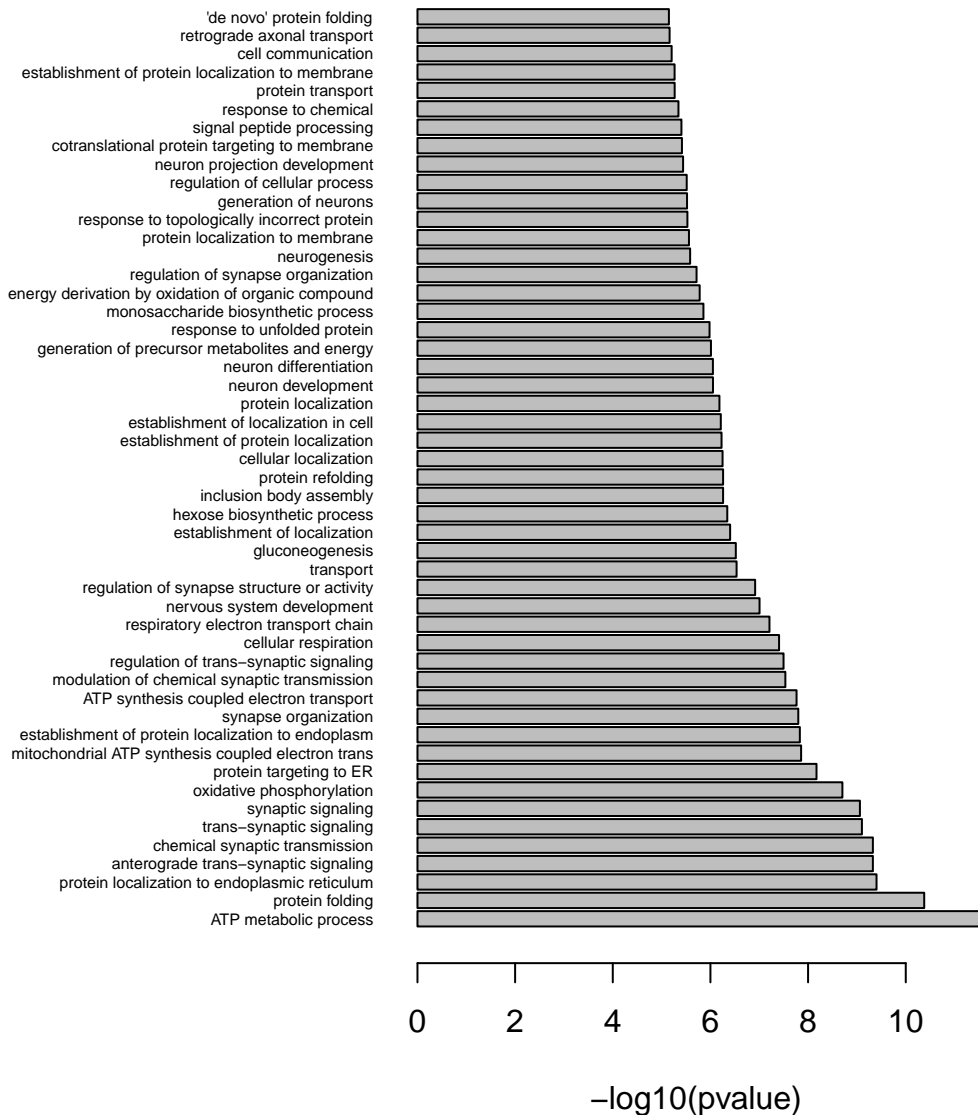

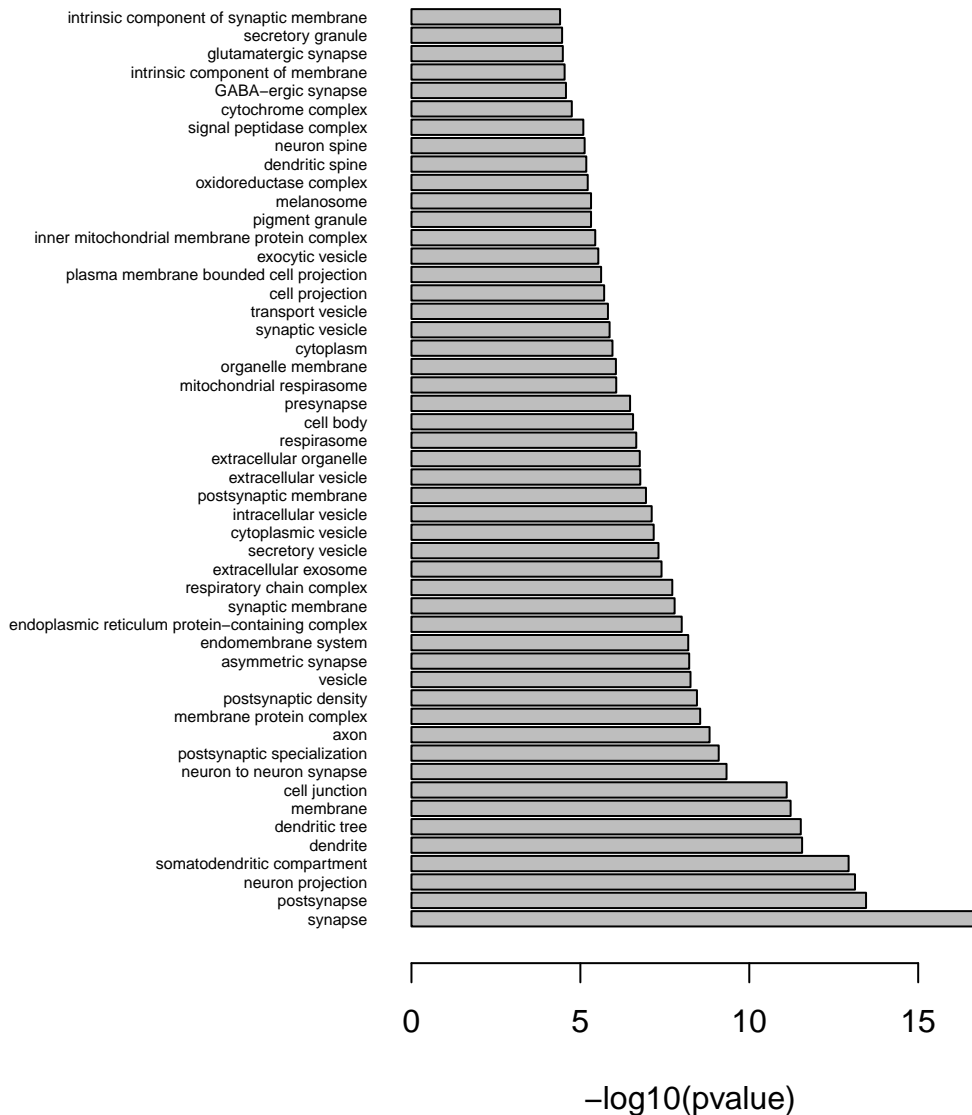

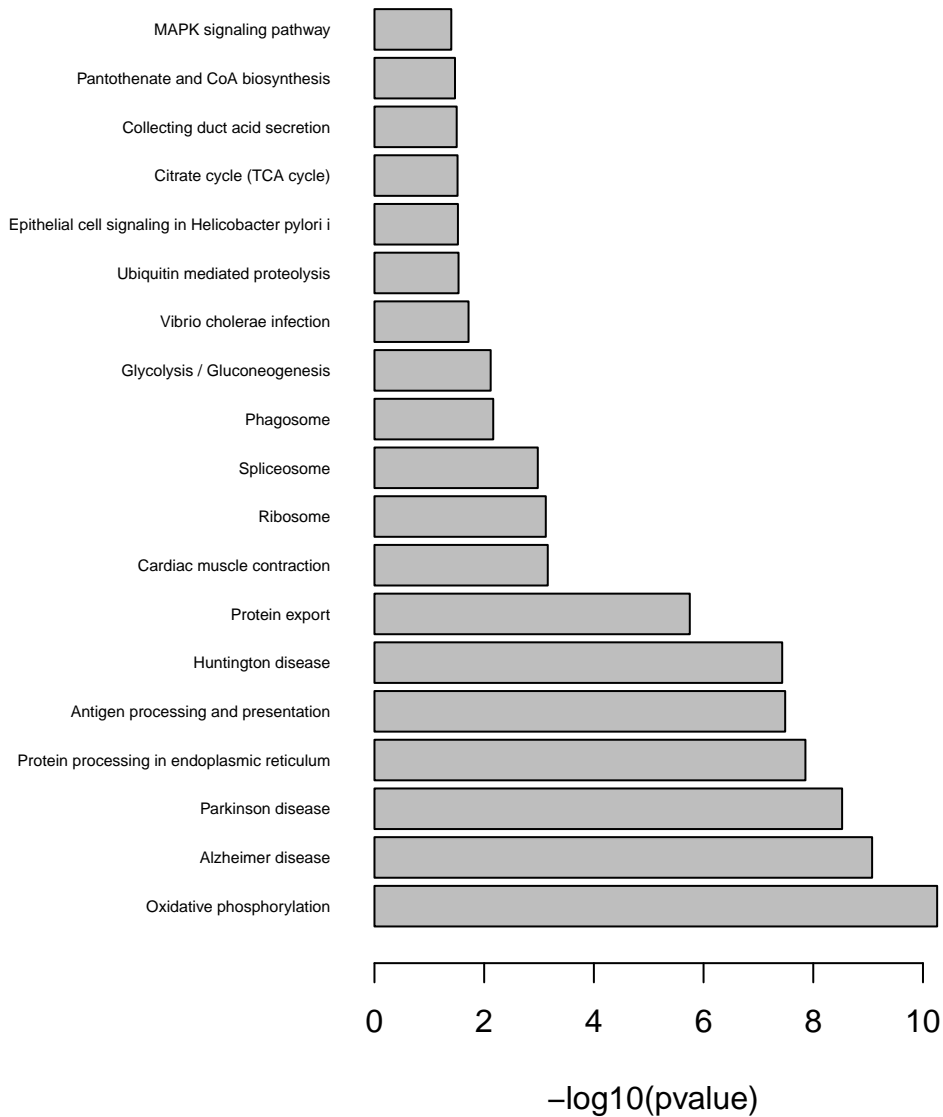

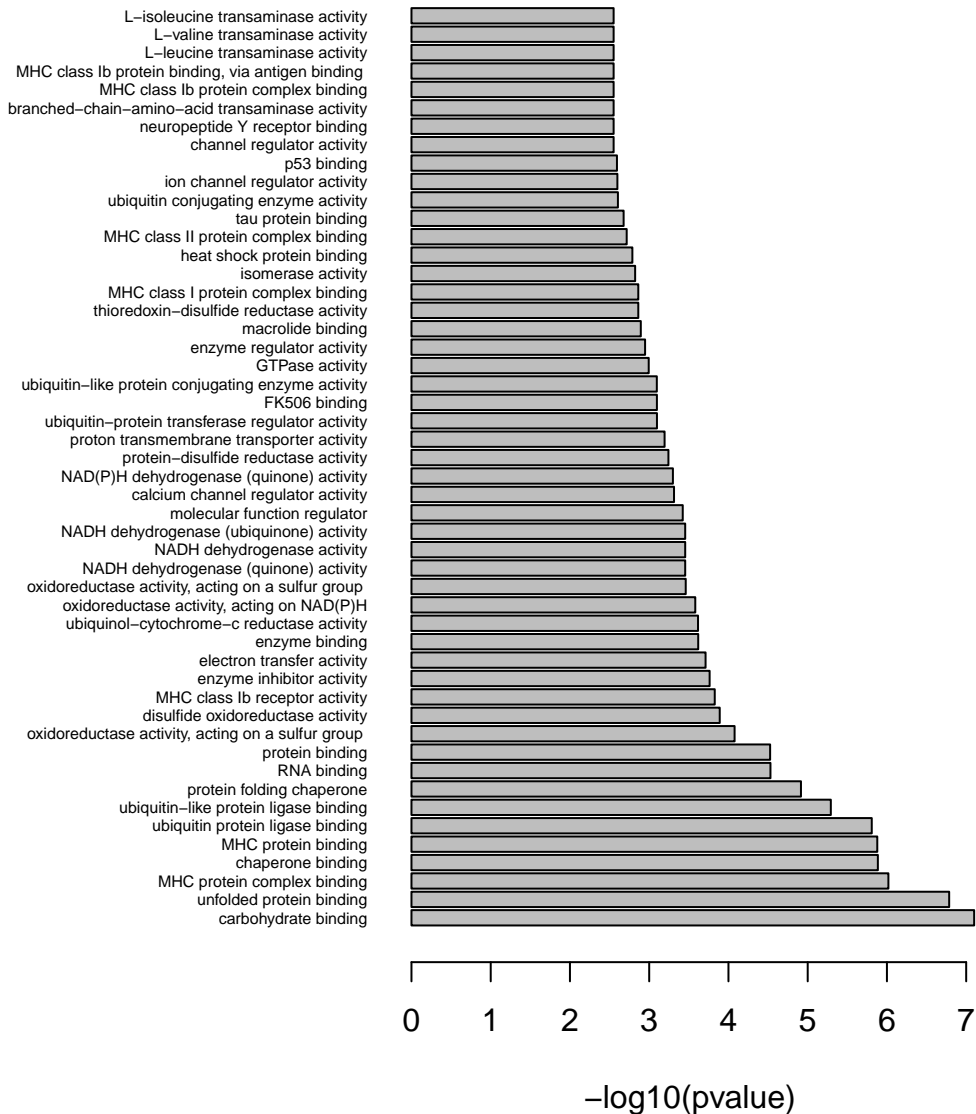

Supplement: Supplementary file 1 [file cells-11-01807-s001.zip › Supplementary_Data/DataS5/dre_cells_GOKEGGs/ab_vs_pbs_cluster1.pdf]

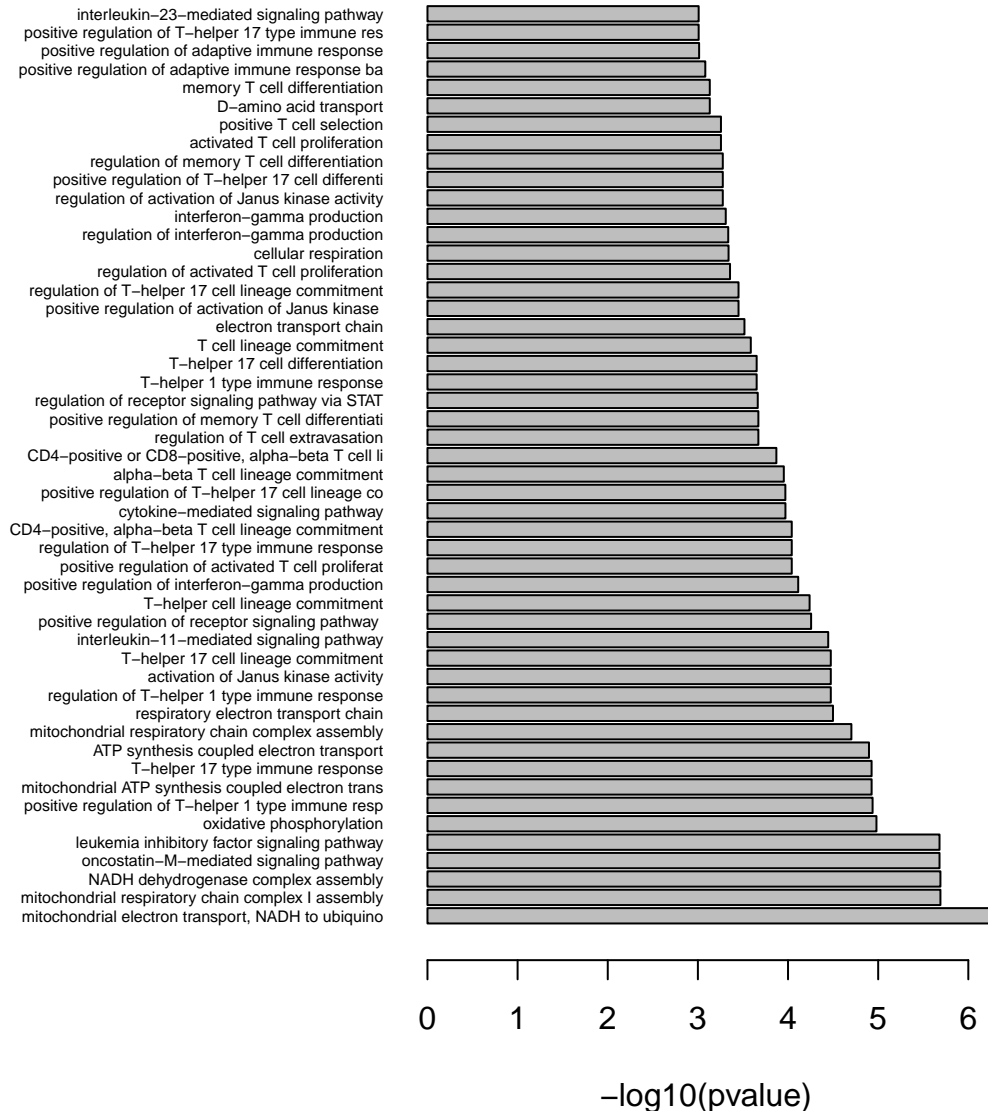

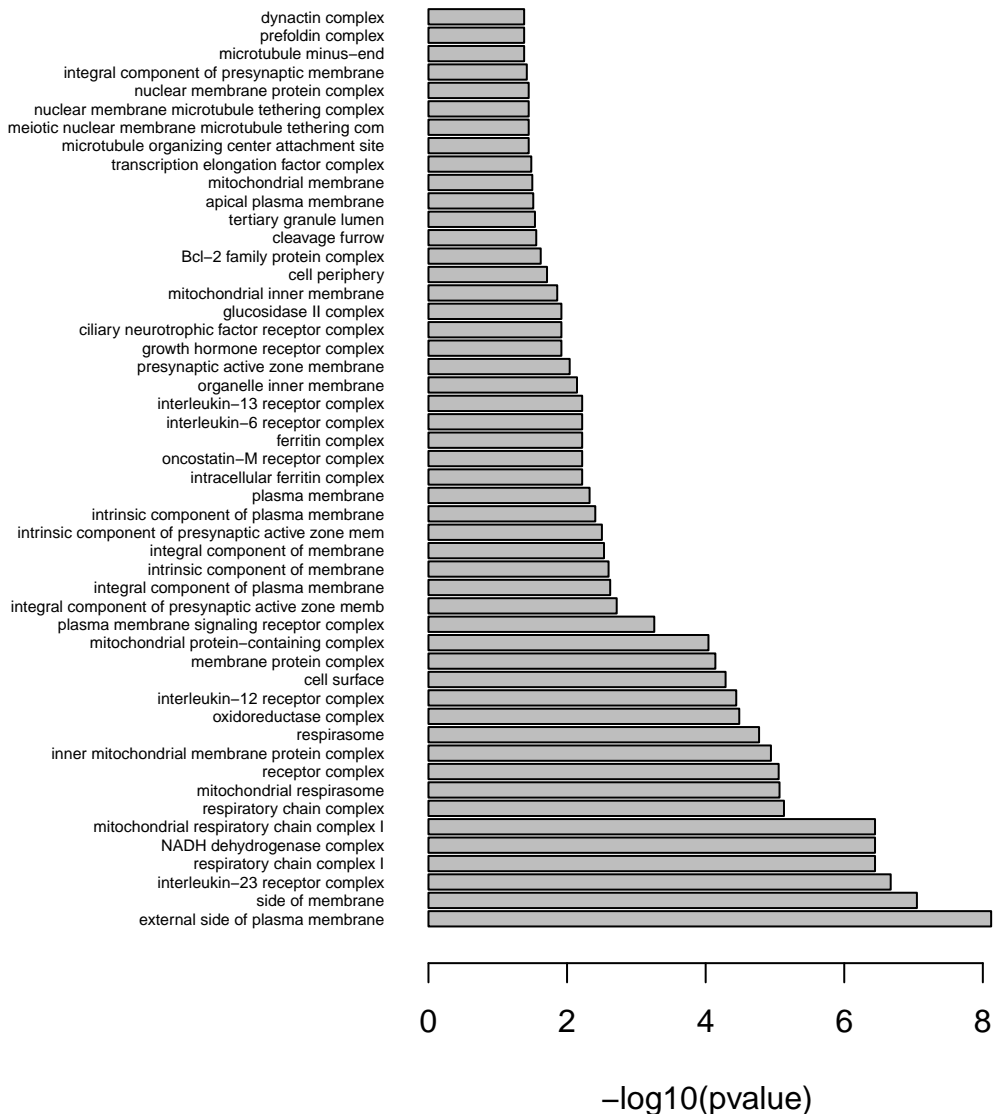

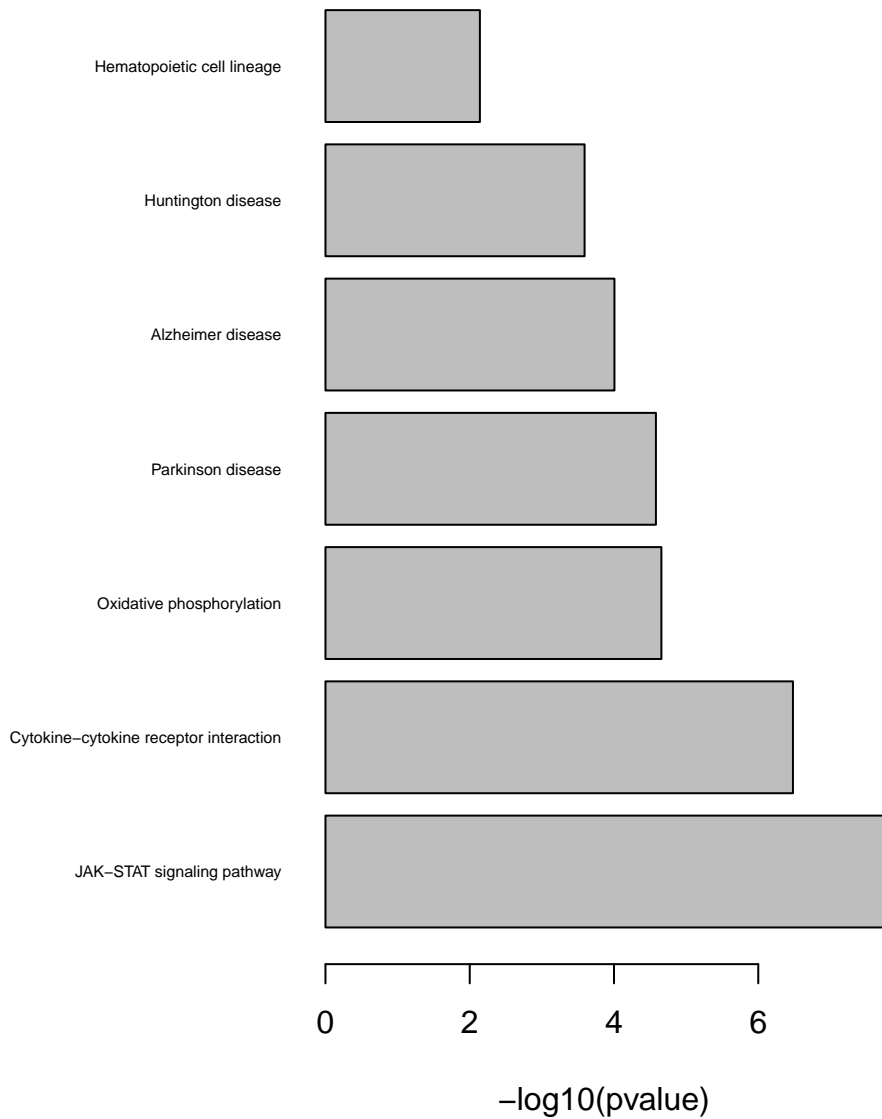

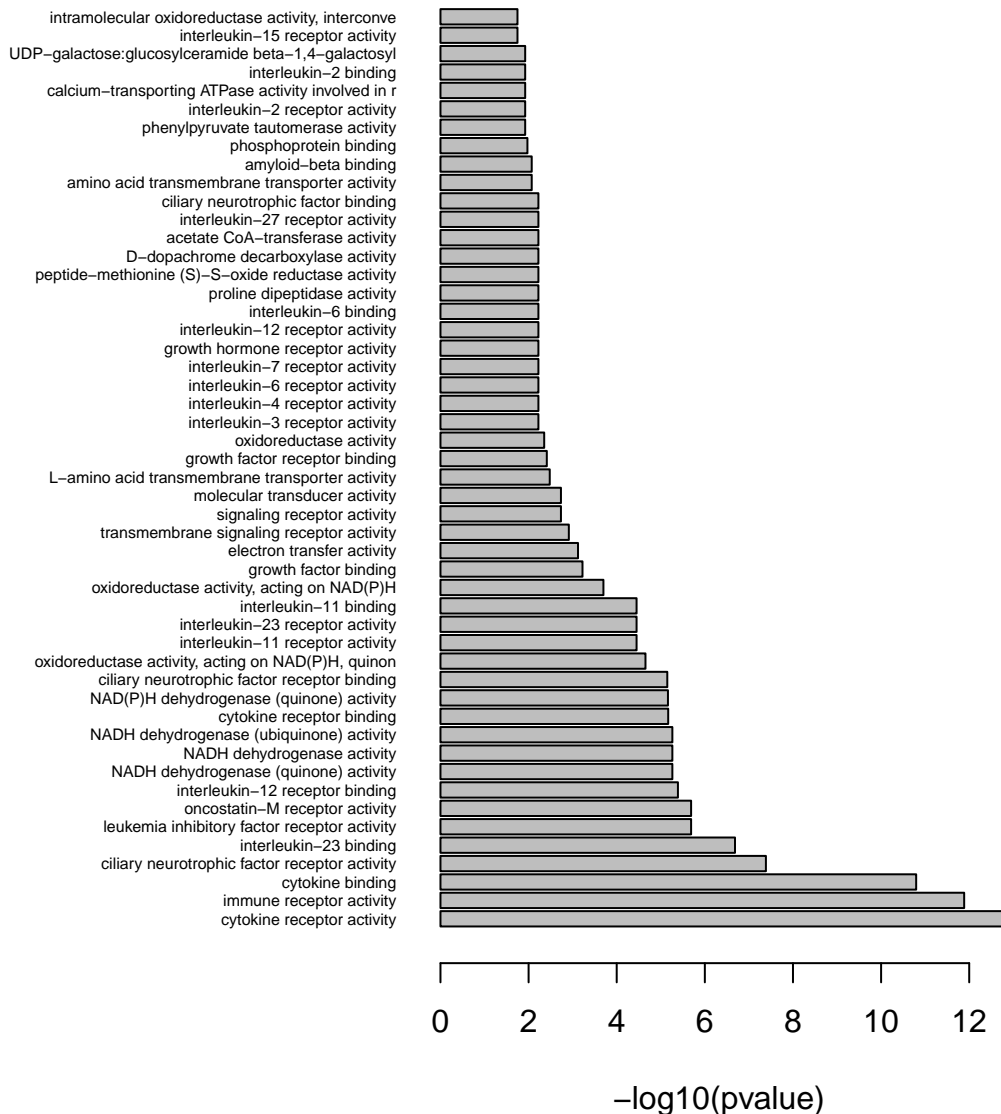

Supplement: Supplementary file 1 [file cells-11-01807-s001.zip › Supplementary_Data/DataS5/dre_cells_GOKEGGs/ab_vs_pbs_cluster2.pdf]

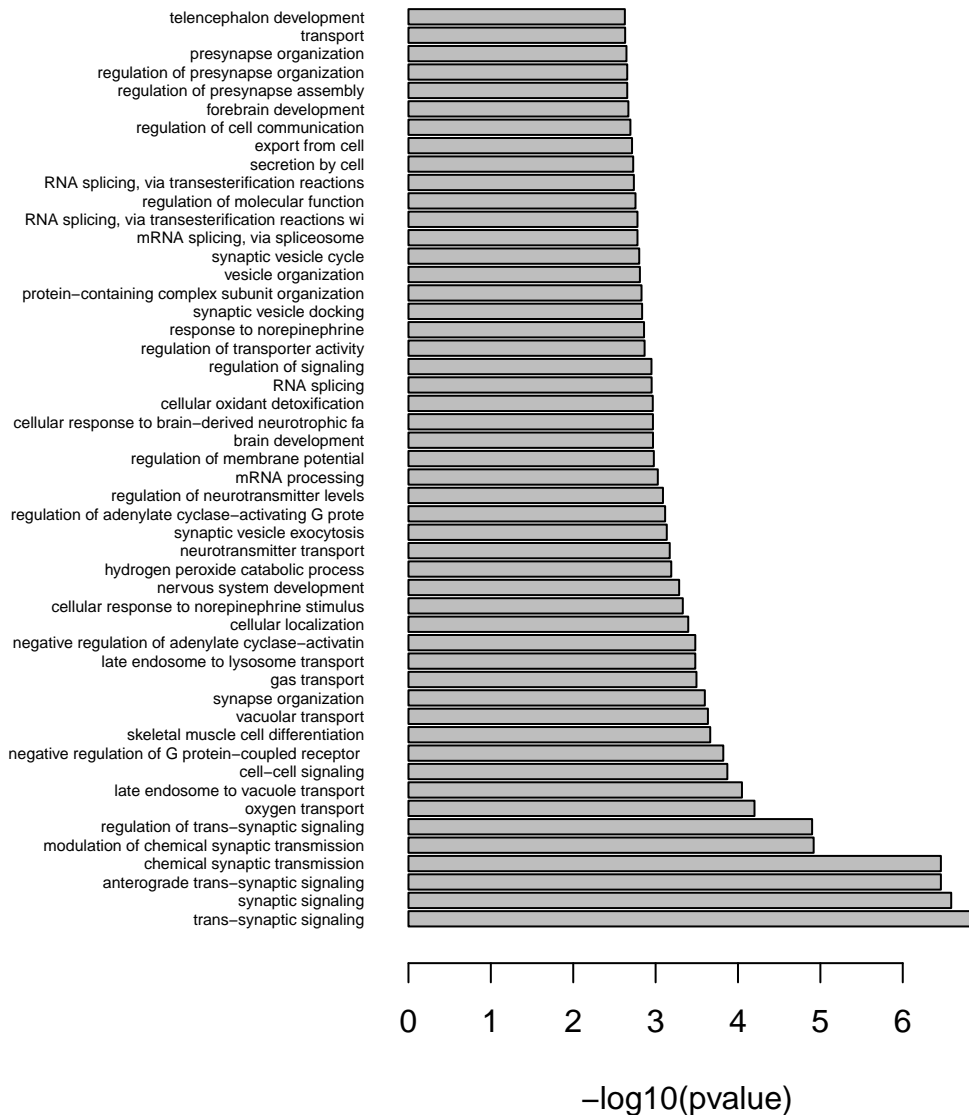

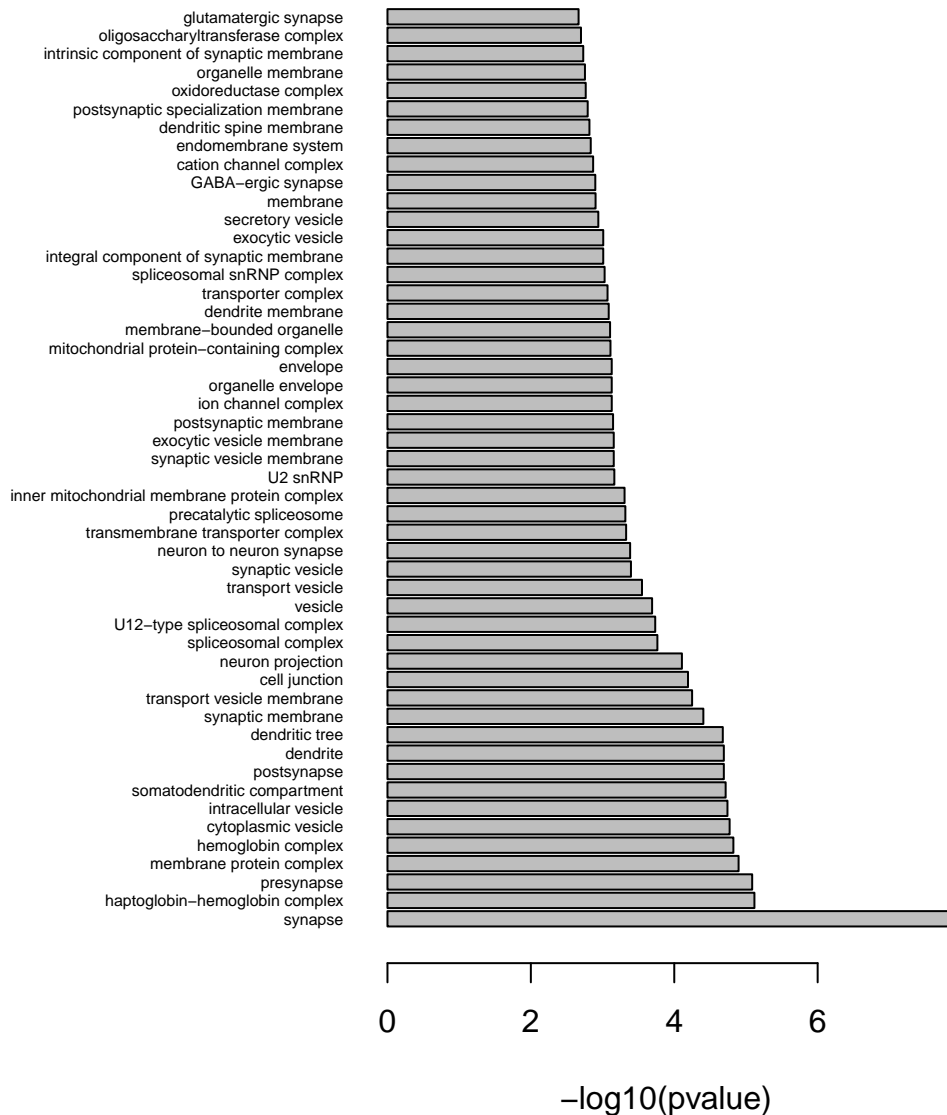

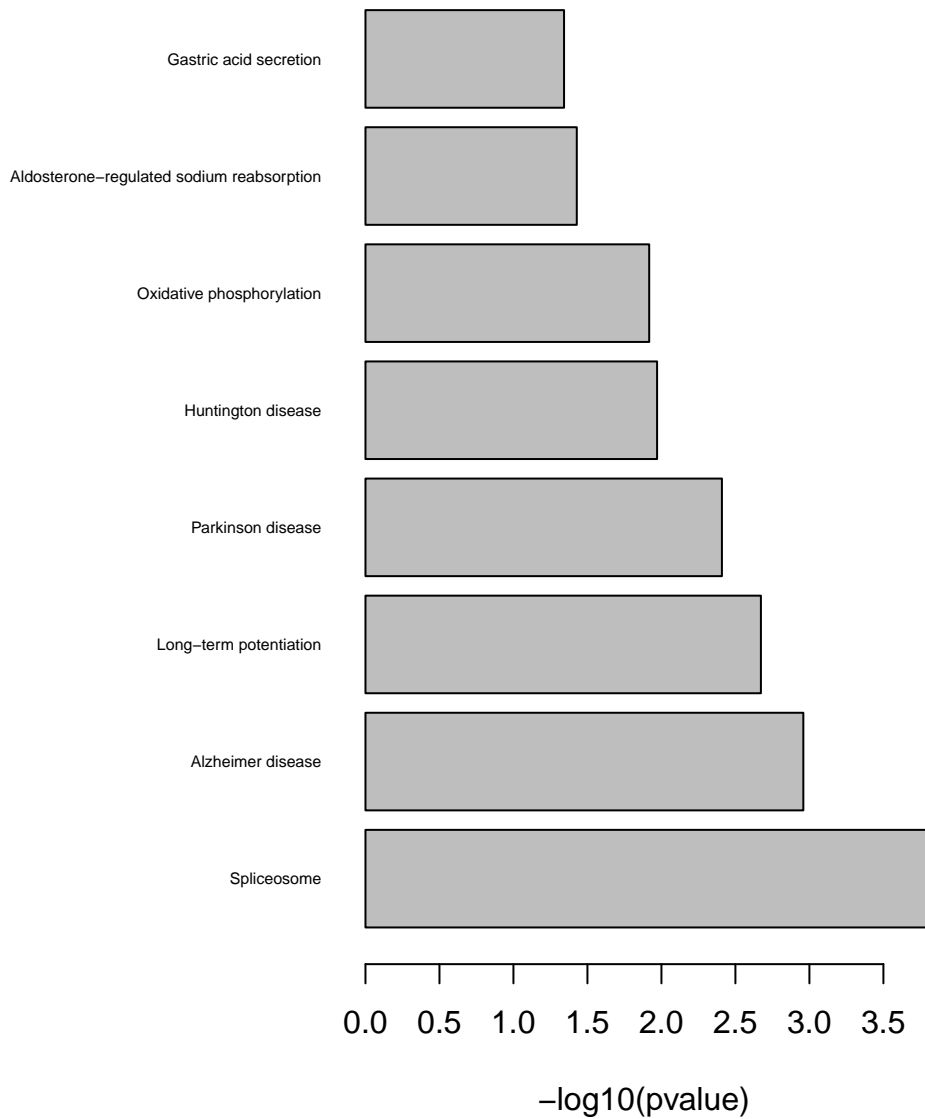

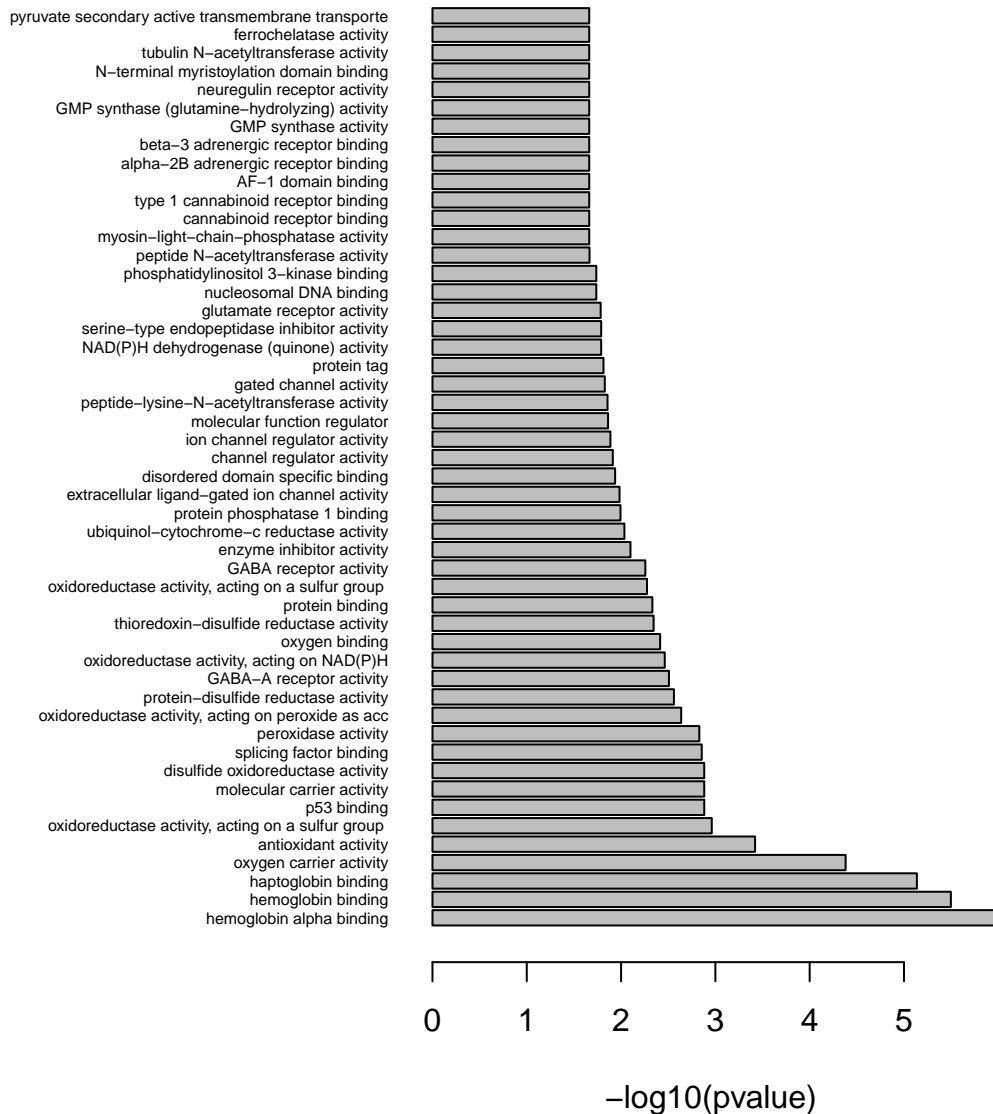

Supplement: Supplementary file 1 [file cells-11-01807-s001.zip › Supplementary_Data/DataS5/dre_cells_GOKEGGs/ab_vs_pbs_cluster3.pdf]

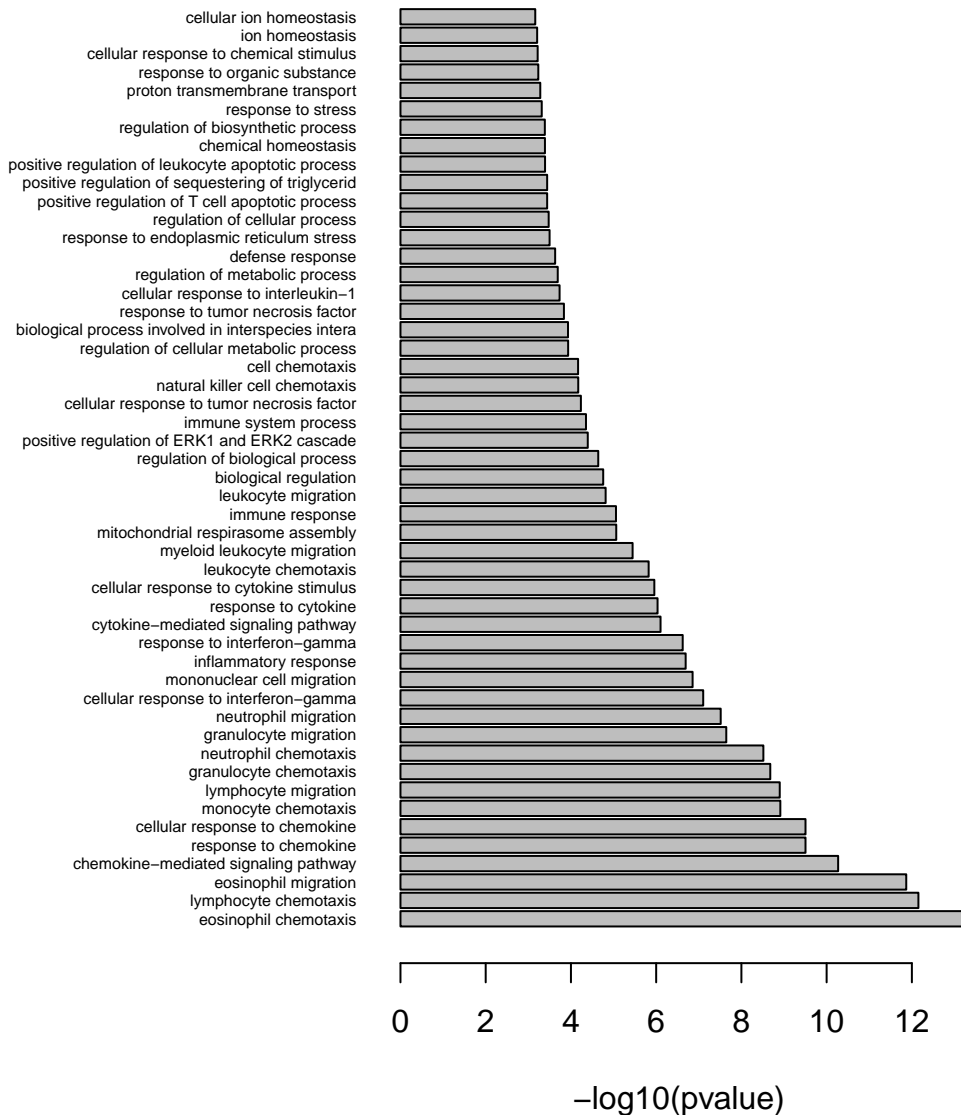

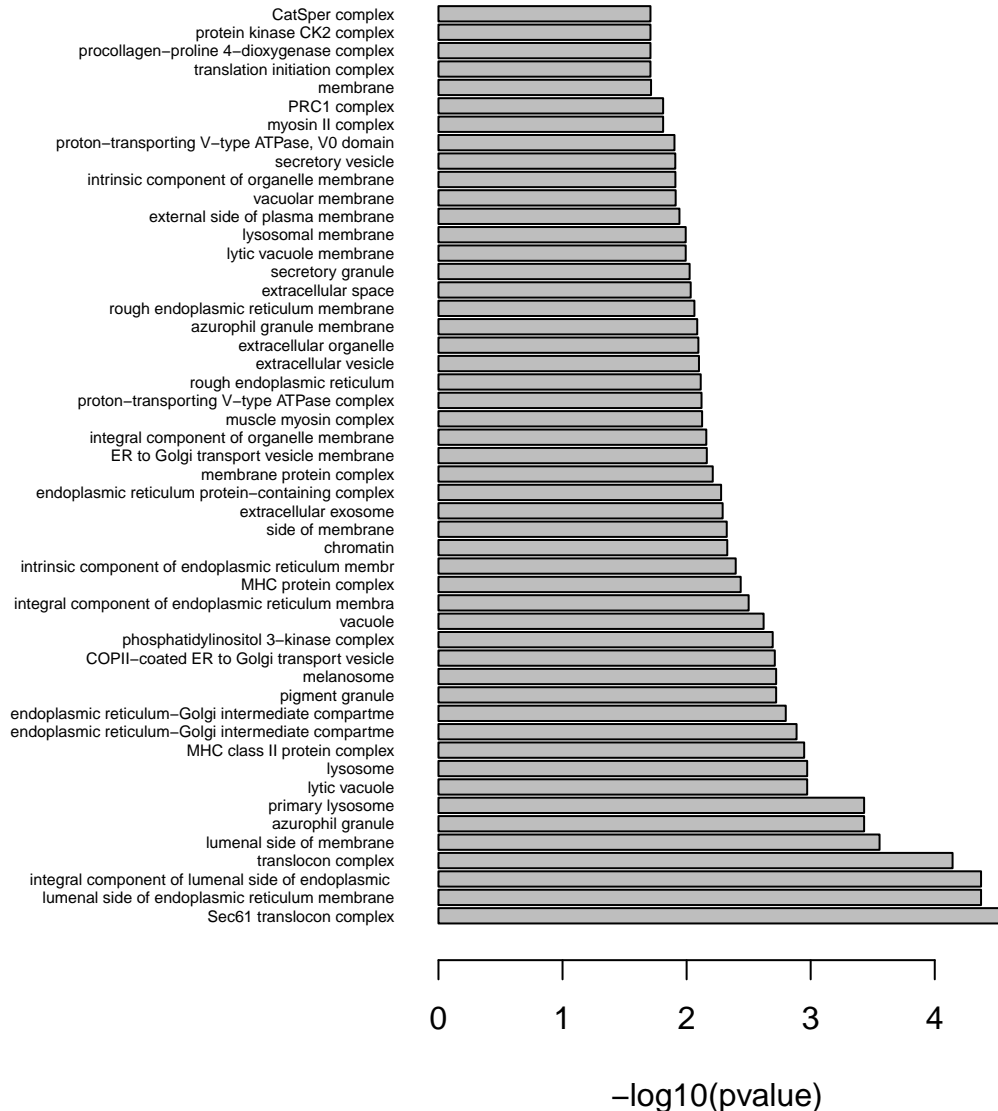

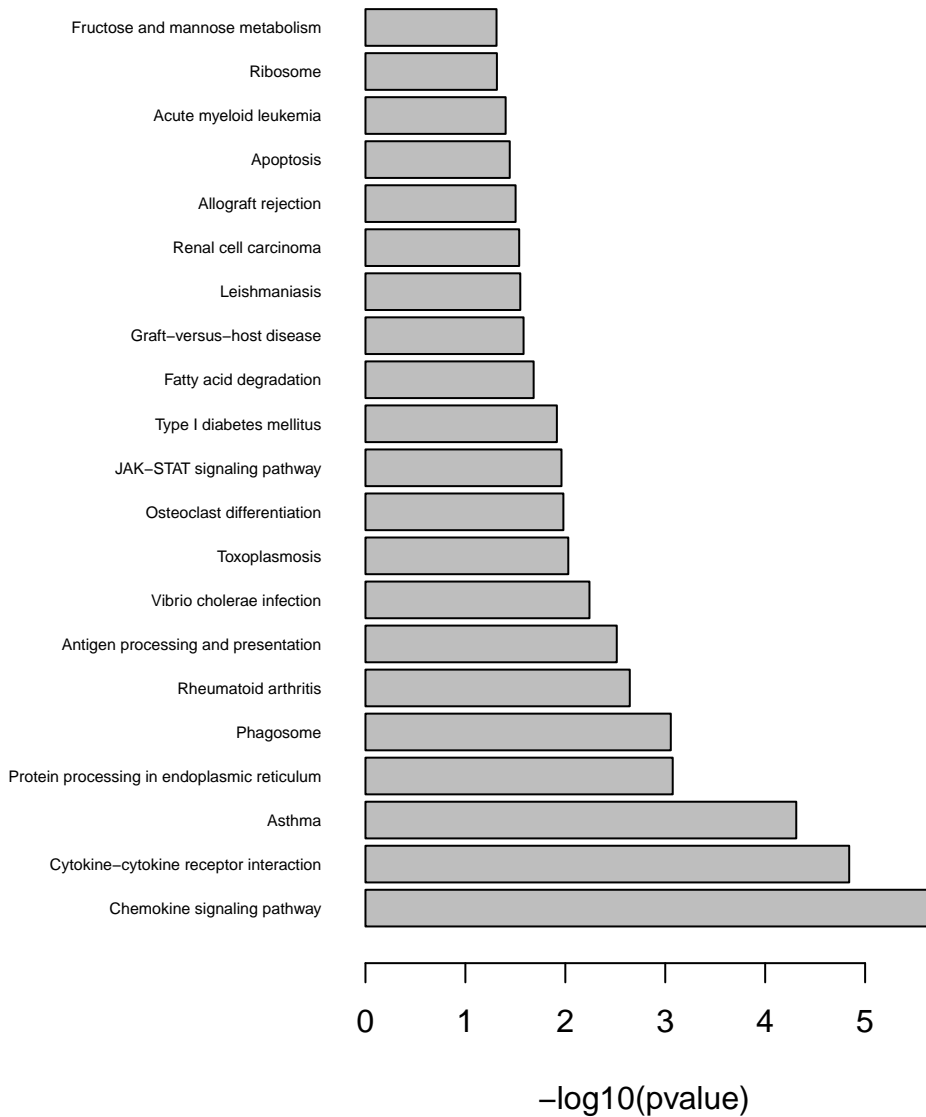

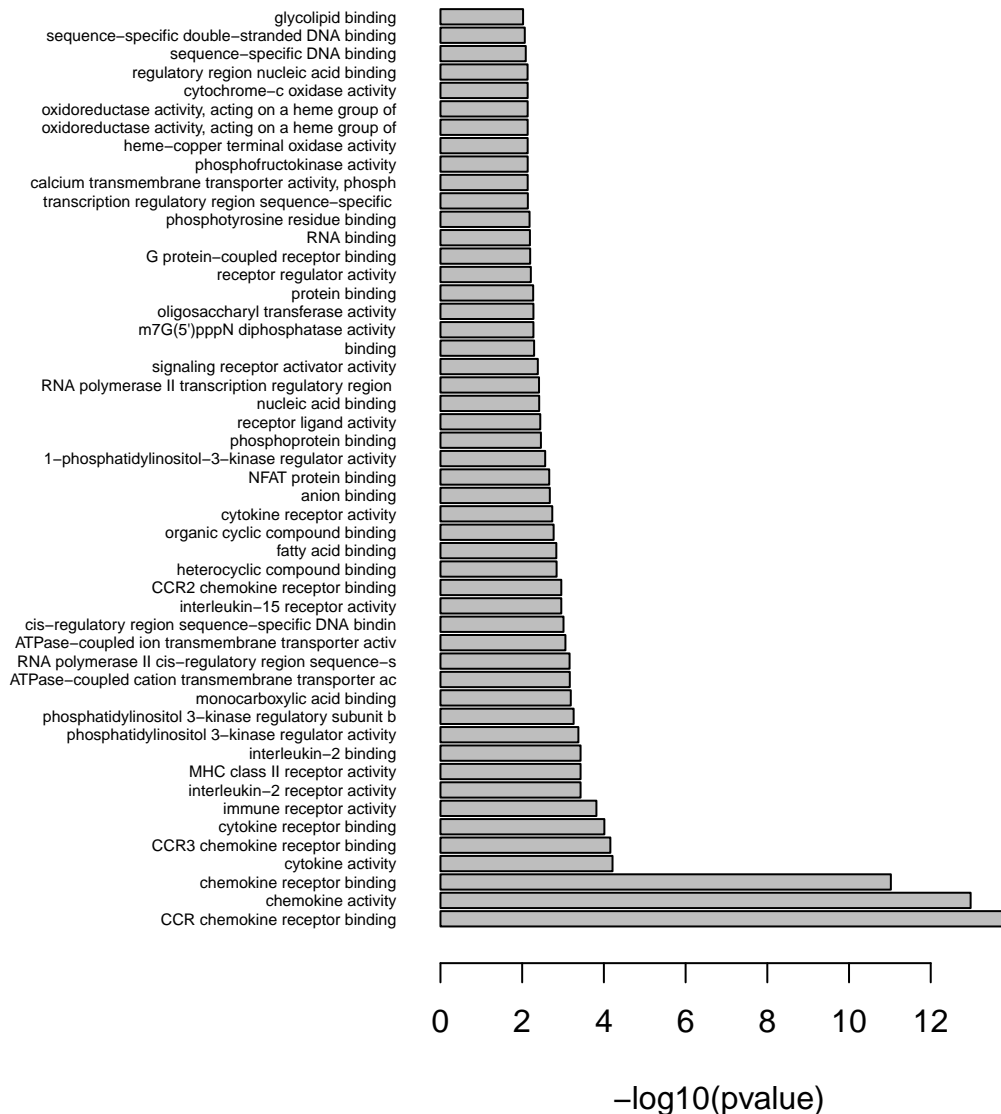

Supplement: Supplementary file 1 [file cells-11-01807-s001.zip › Supplementary_Data/DataS5/dre_cells_GOKEGGs/ab_vs_pbs_cluster18.pdf]

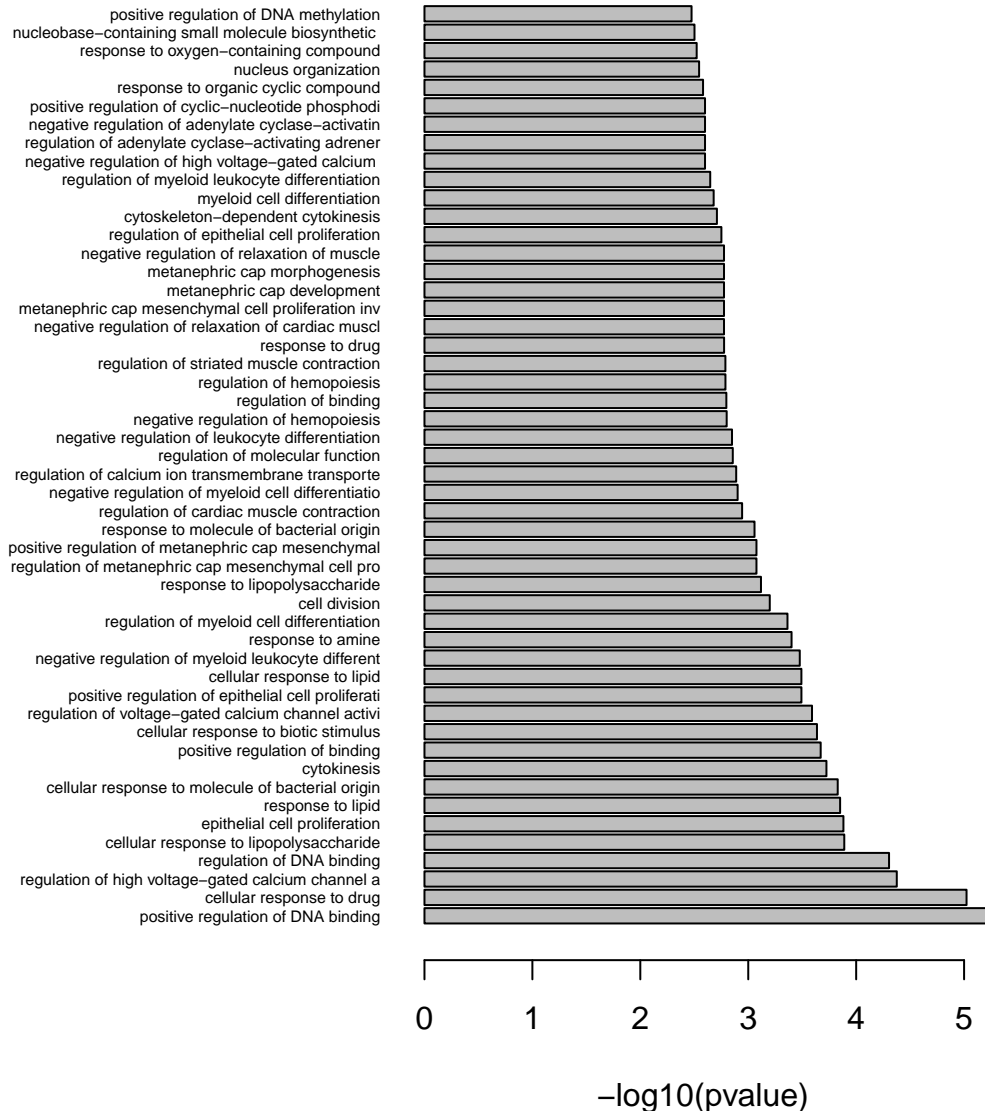

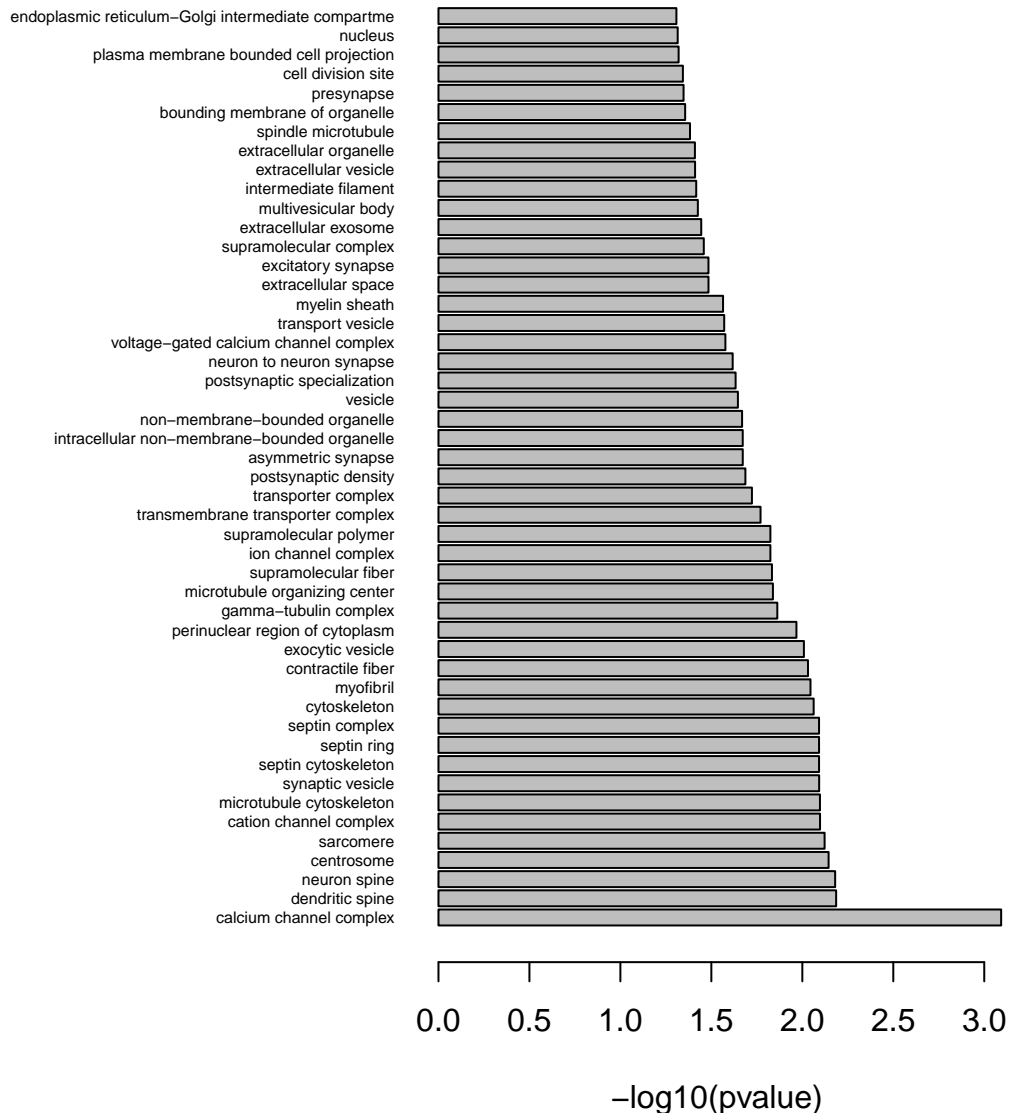

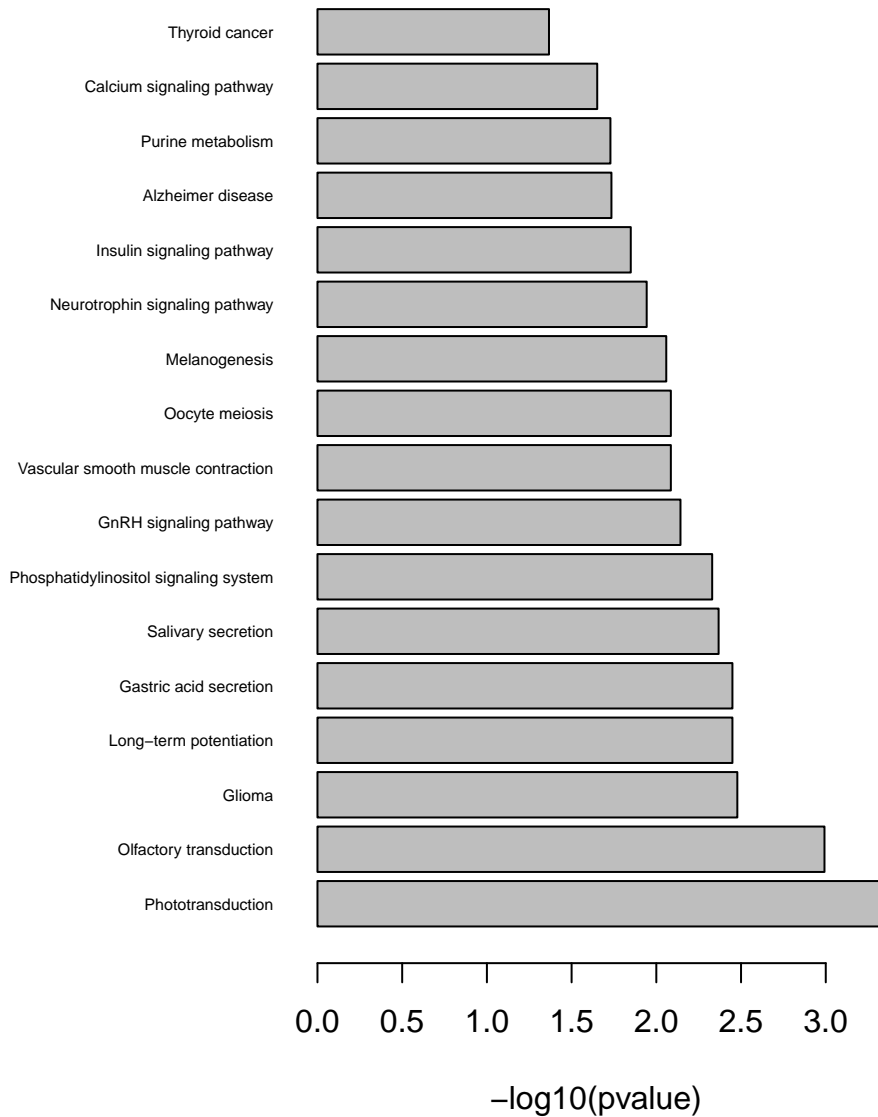

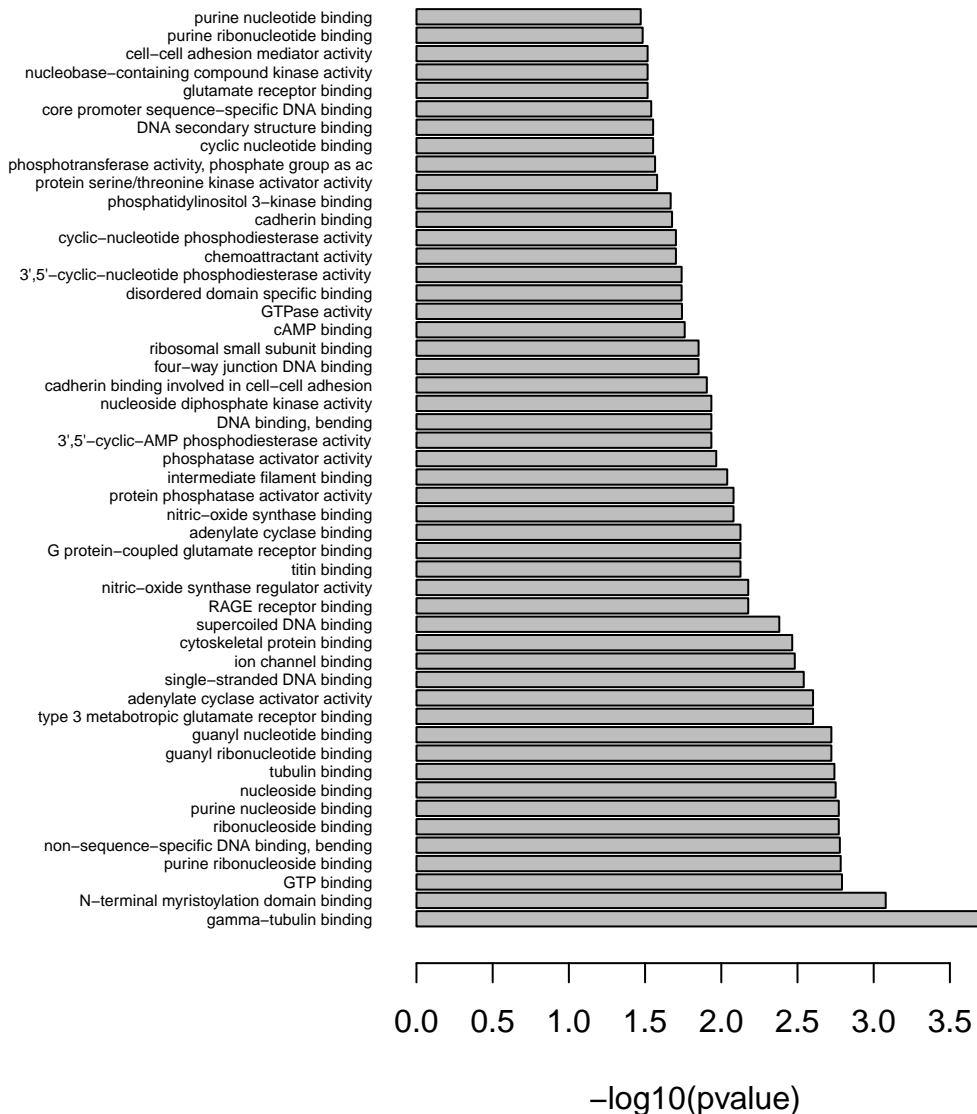

Supplement: Supplementary file 1 [file cells-11-01807-s001.zip › Supplementary_Data/DataS5/dre_cells_GOKEGGs/ab_vs_pbs_cluster24.pdf]

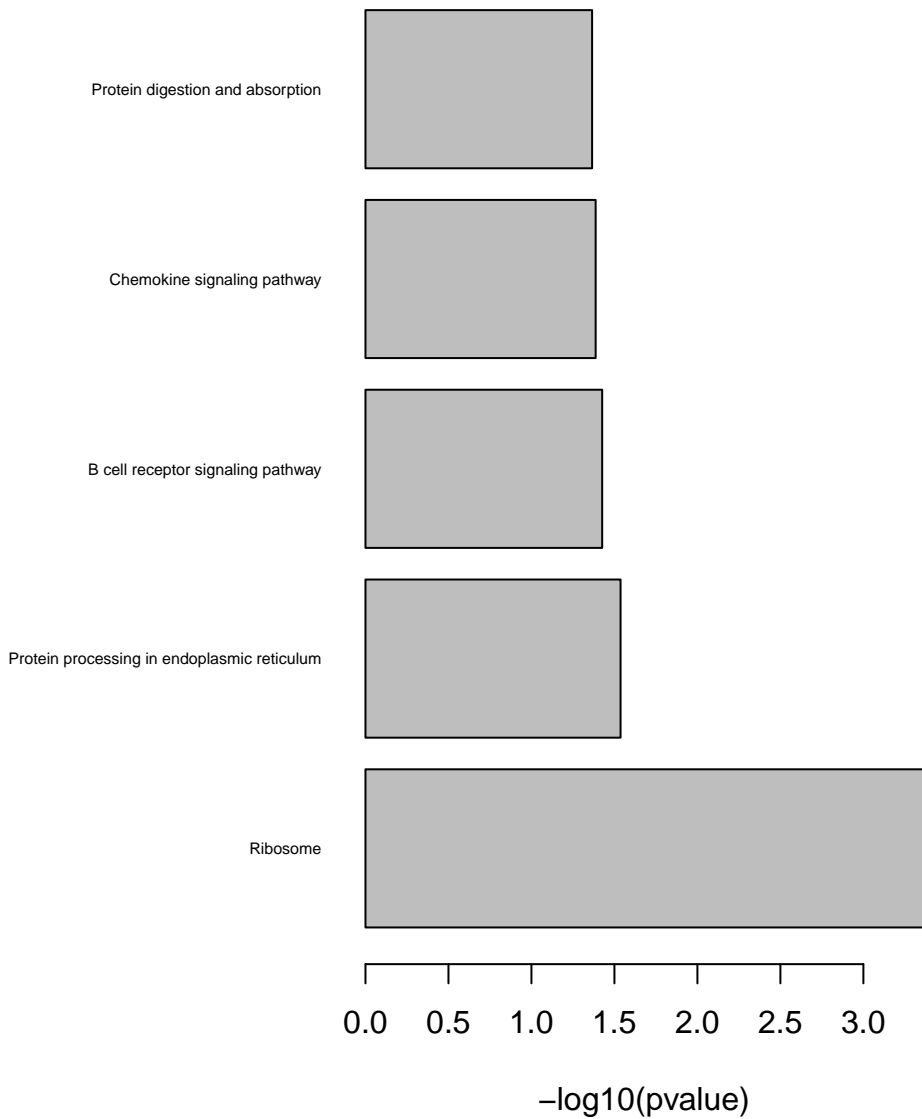

Supplement: Supplementary file 1 [file cells-11-01807-s001.zip › Supplementary_Data/DataS11/allDEGs/DataS11_pVal_GOstats_kegg_Up.pdf]

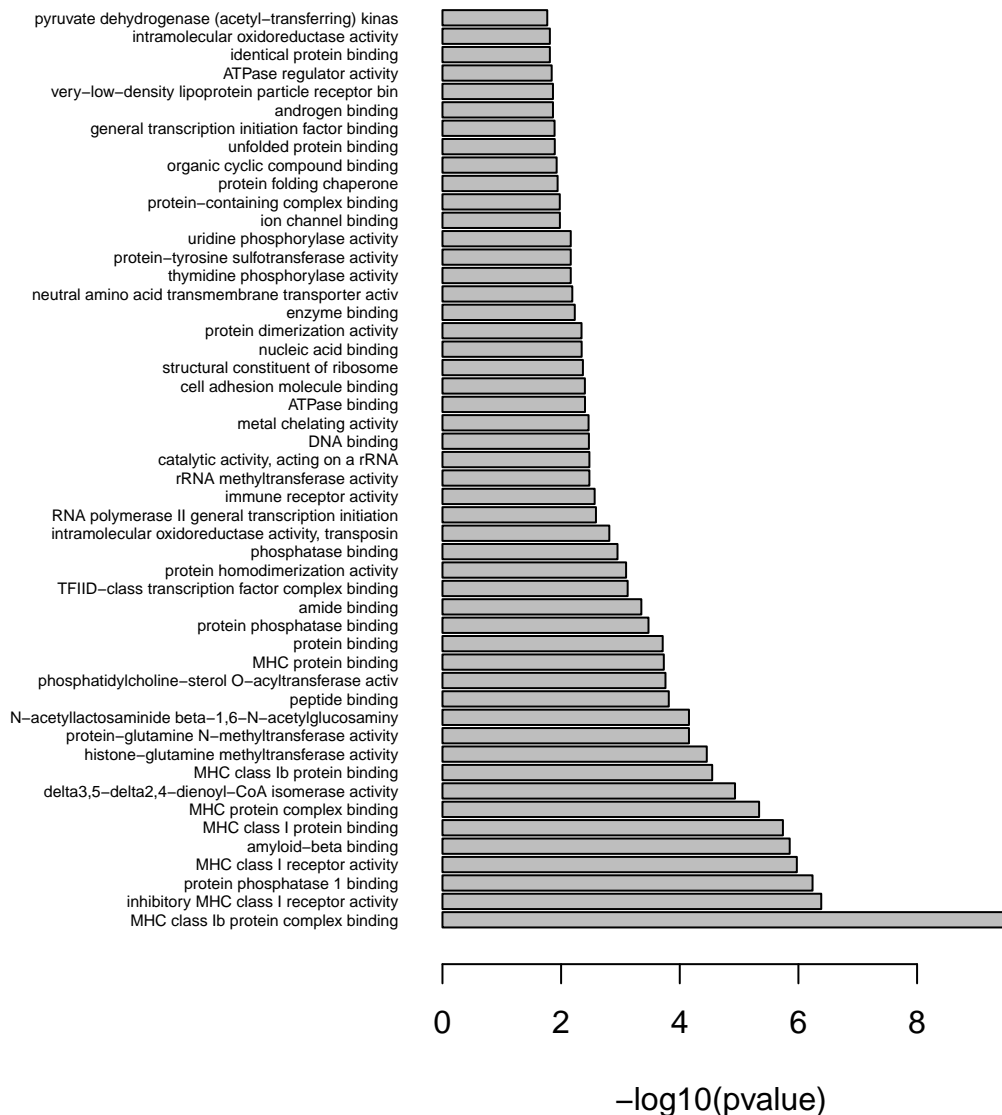

Supplement: Supplementary file 1 [file cells-11-01807-s001.zip › Supplementary_Data/DataS11/allDEGs/DataS11_pVal_GOstats_MF_Up_pieChart.pdf]

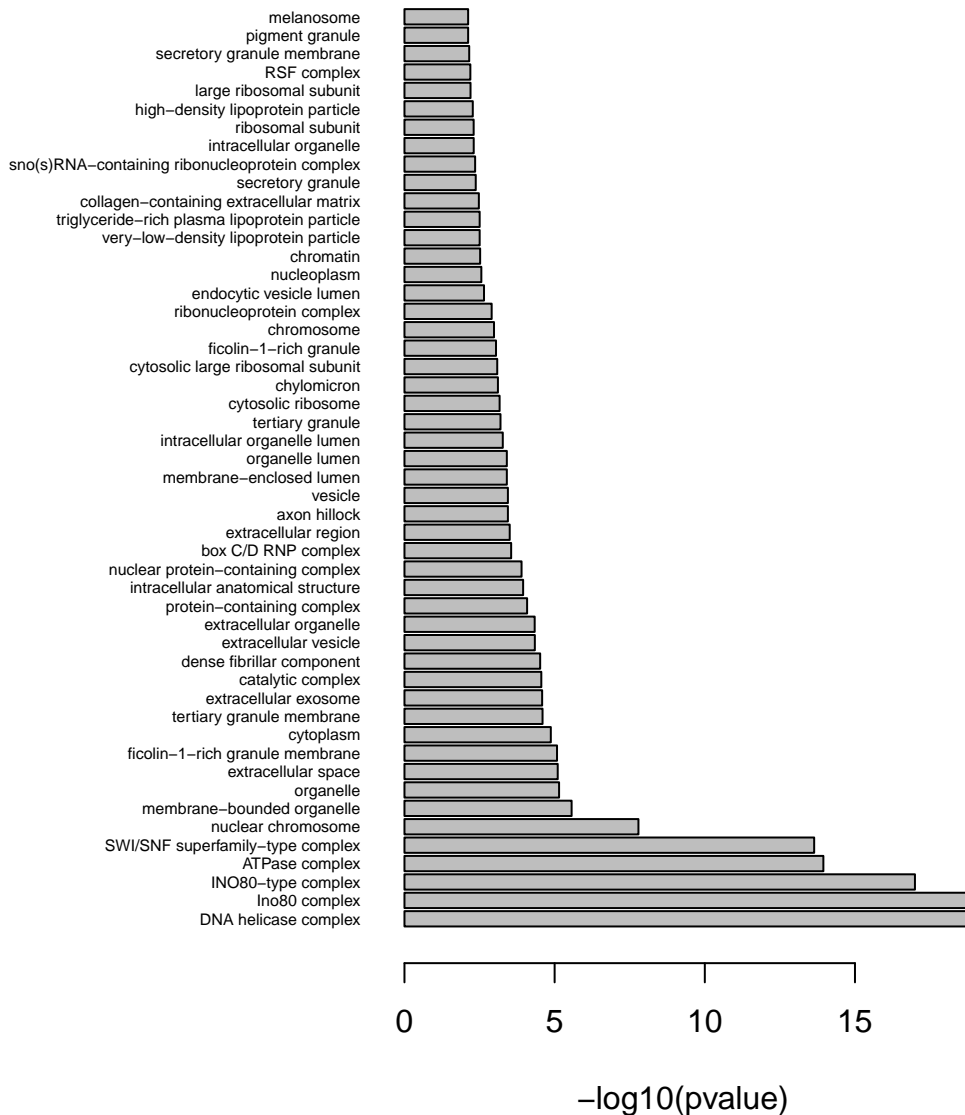

Supplement: Supplementary file 1 [file cells-11-01807-s001.zip › Supplementary_Data/DataS11/allDEGs/DataS11_pVal_GOstats_CC_Up_pieChart.pdf]

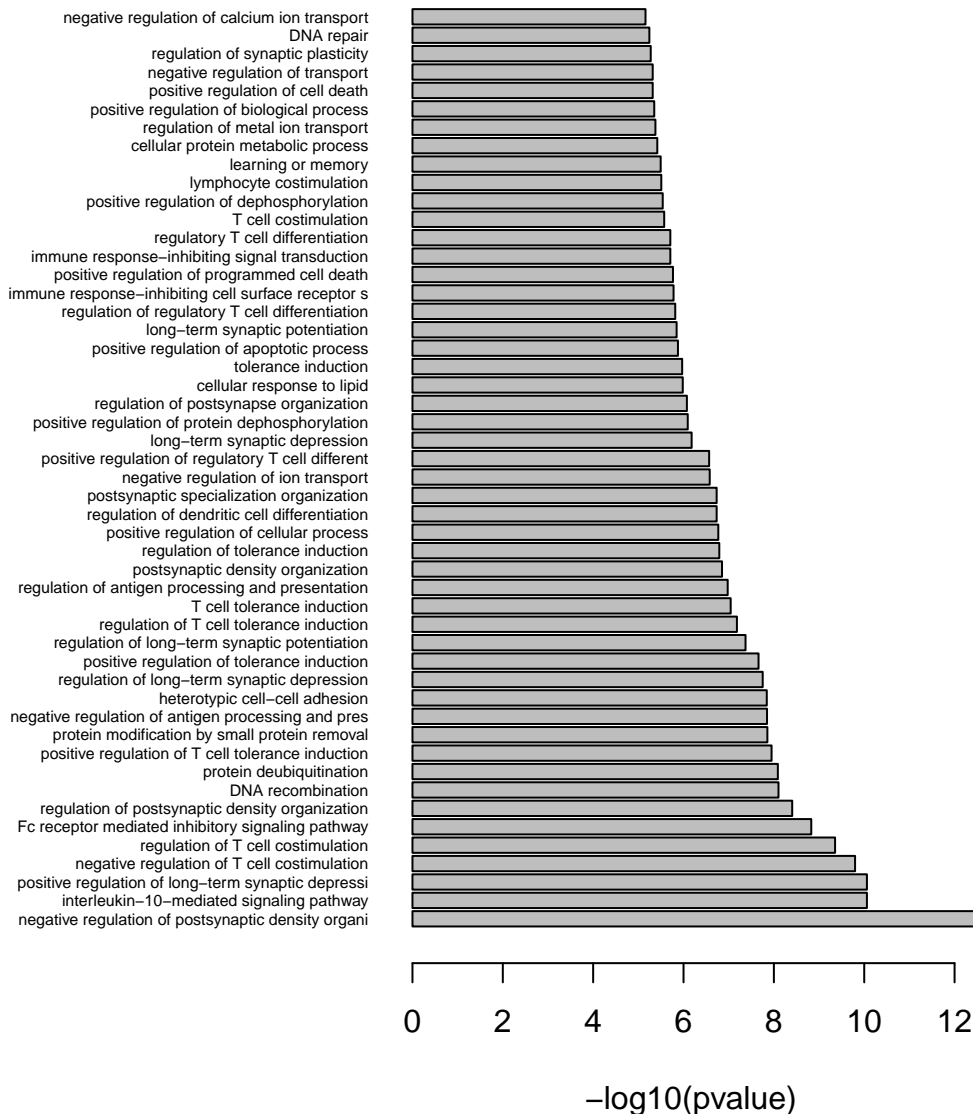

Supplement: Supplementary file 1 [file cells-11-01807-s001.zip › Supplementary_Data/DataS11/allDEGs/DataS11_pVal_GOstats_BP_Up_pieChart.pdf]

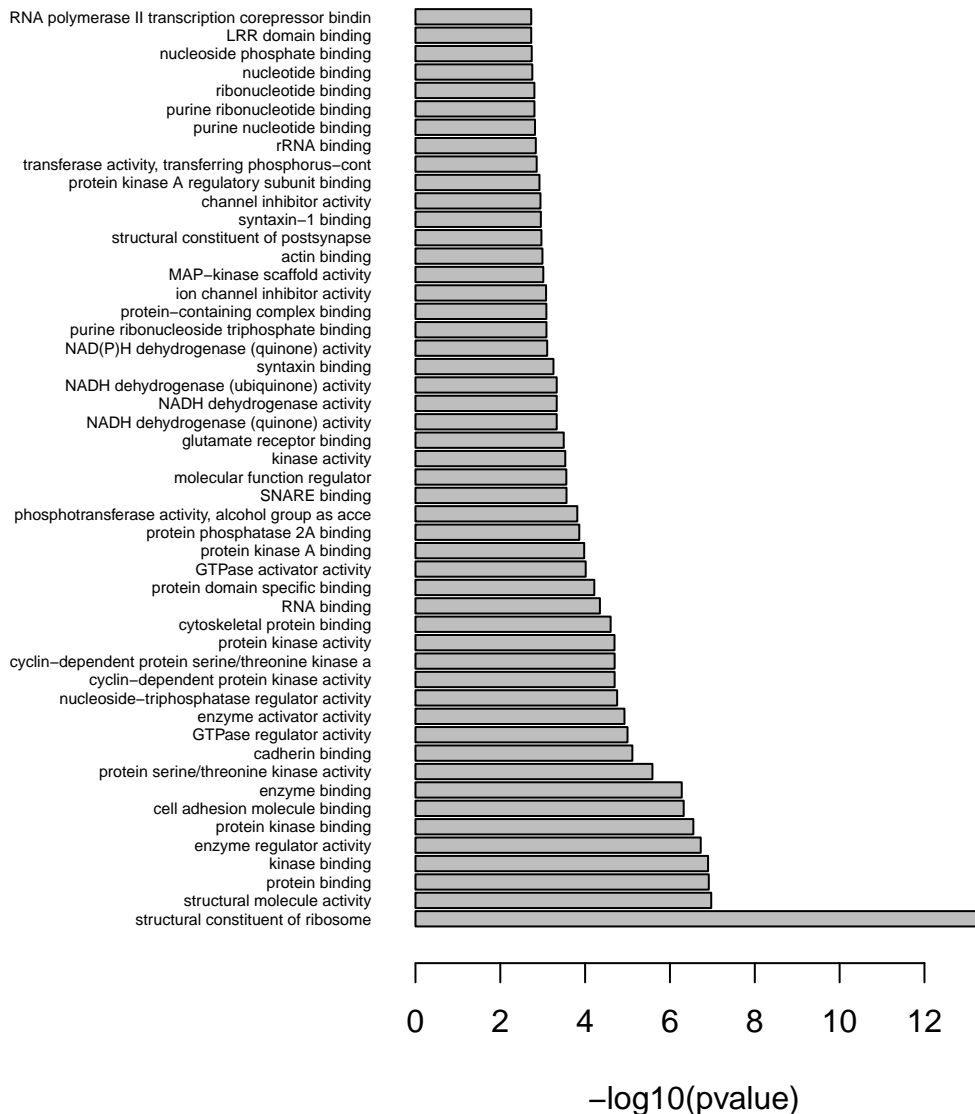

Supplement: Supplementary file 1 [file cells-11-01807-s001.zip › Supplementary_Data/DataS5/SFG_cells_GOKEGGs/SFGBS6_14_vs_SFGBS0_14/pVal_GOstats_MF_Up_pieChart.pdf]

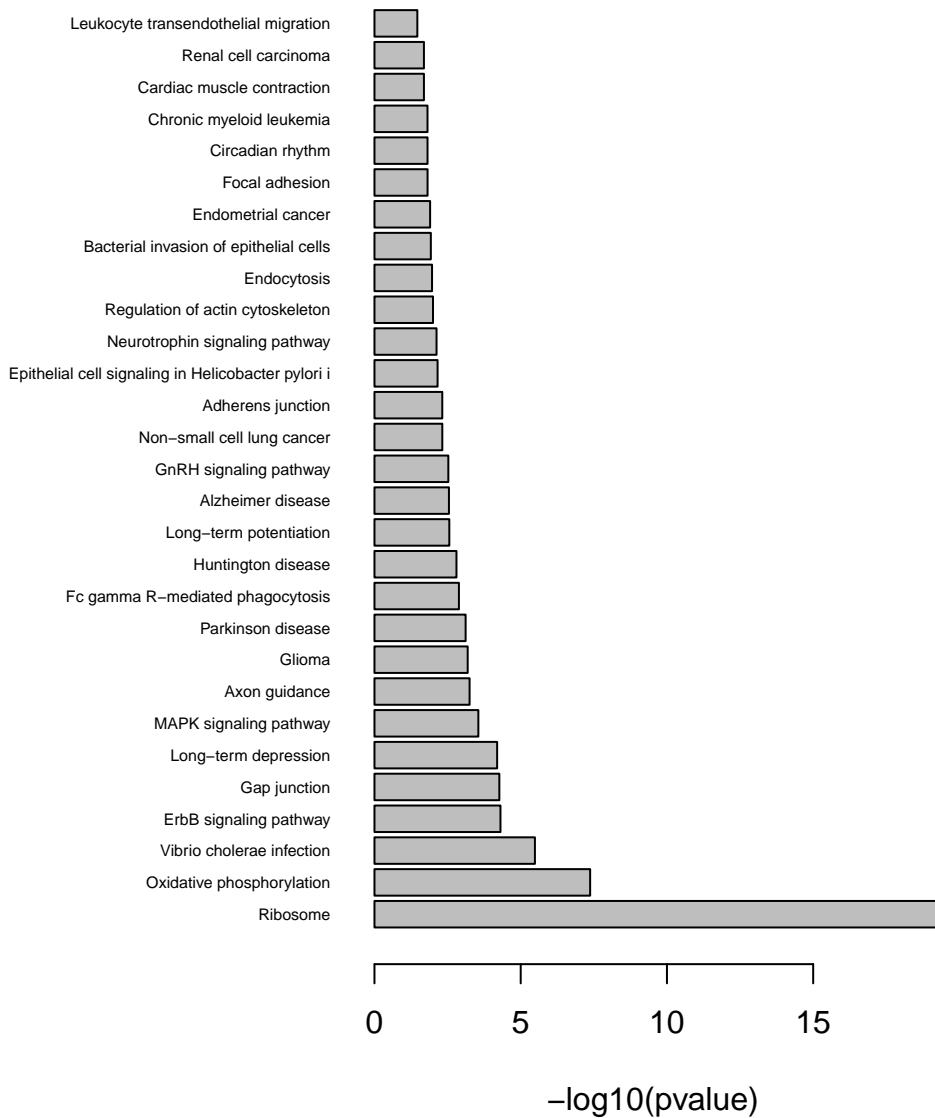

Supplement: Supplementary file 1 [file cells-11-01807-s001.zip › Supplementary_Data/DataS5/SFG_cells_GOKEGGs/SFGBS6_14_vs_SFGBS0_14/pVal_GOstats_kegg_Up.pdf]

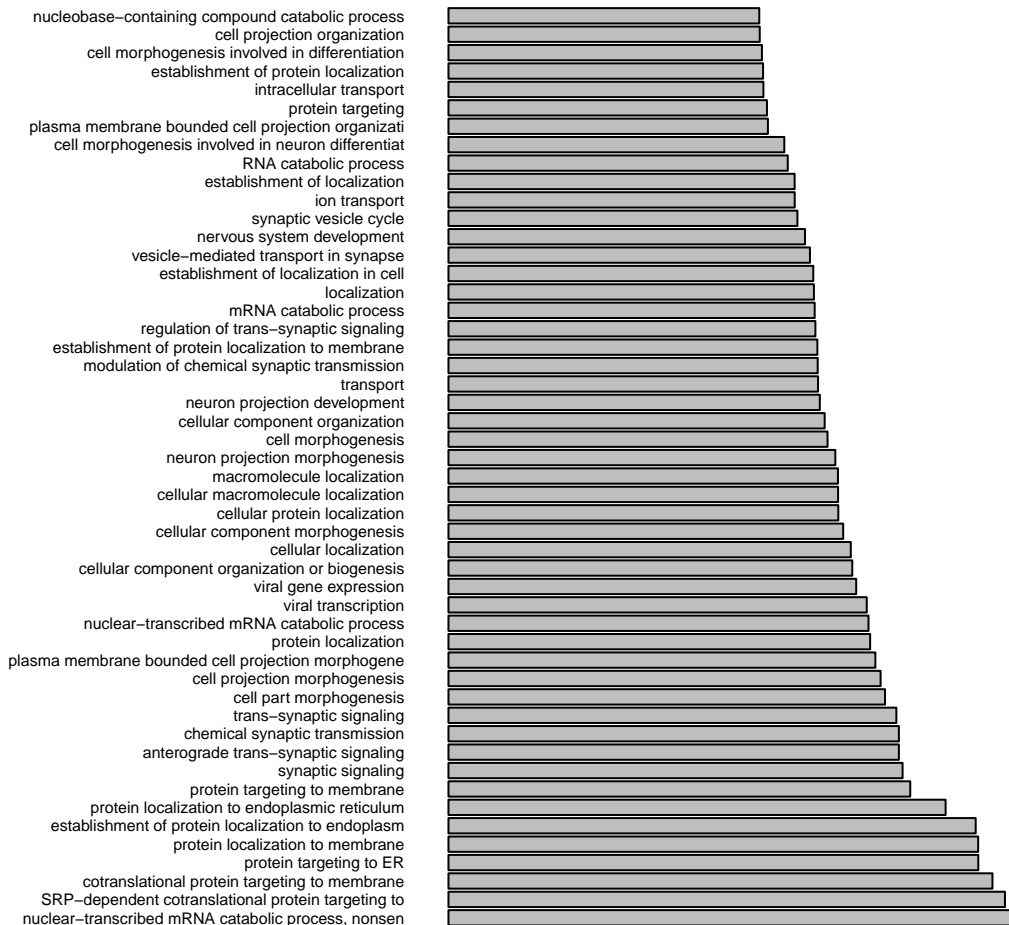

0 5 10 15

$-\log_{10}(\text{pvalue})$

Supplement: Supplementary file 1 [file cells-11-01807-s001.zip › Supplementary_Data/DataS5/SFG_cells_GOKEGGs/SFGBS6_14_vs_SFGBS0_14/pVal_GOstats_BP_Up_pieChart.pdf]

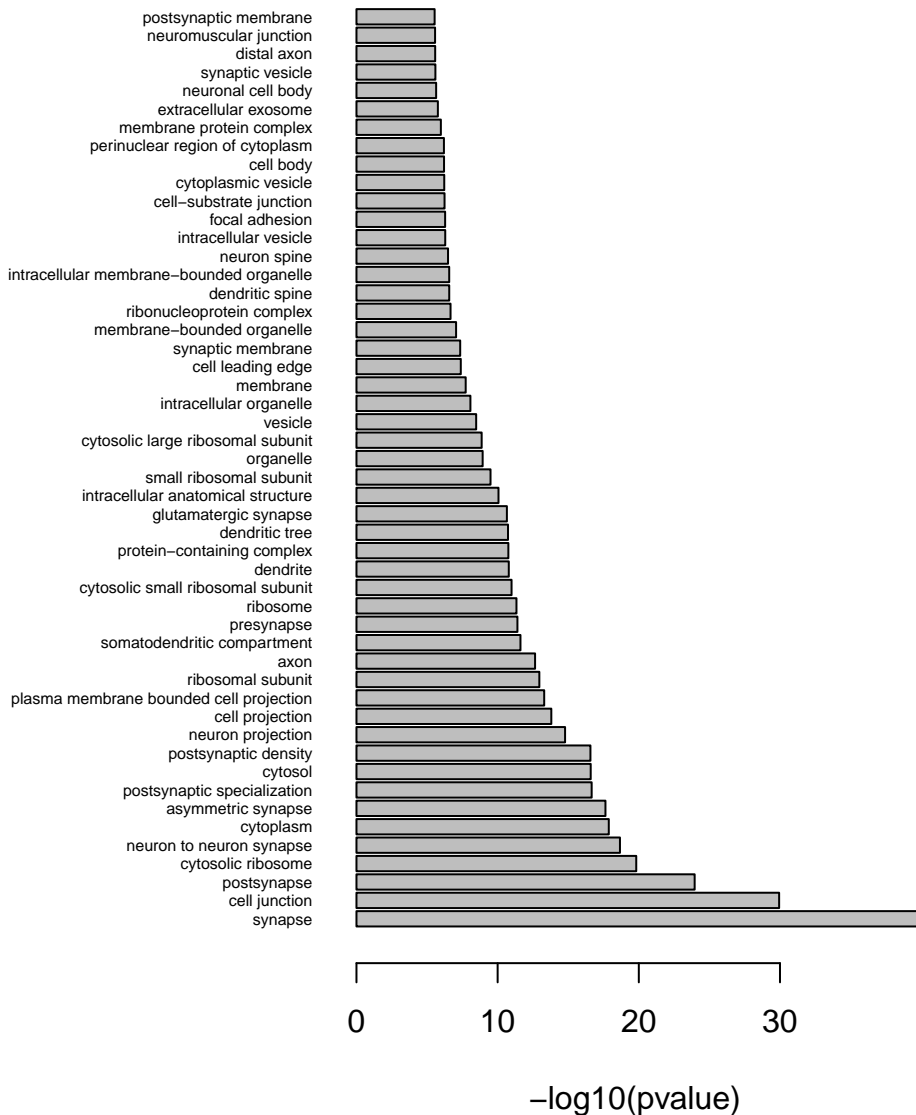

Supplement: Supplementary file 1 [file cells-11-01807-s001.zip › Supplementary_Data/DataS5/SFG_cells_GOKEGGs/SFGBS6_14_vs_SFGBS0_14/pVal_GOstats_CC_Up_pieChart.pdf]

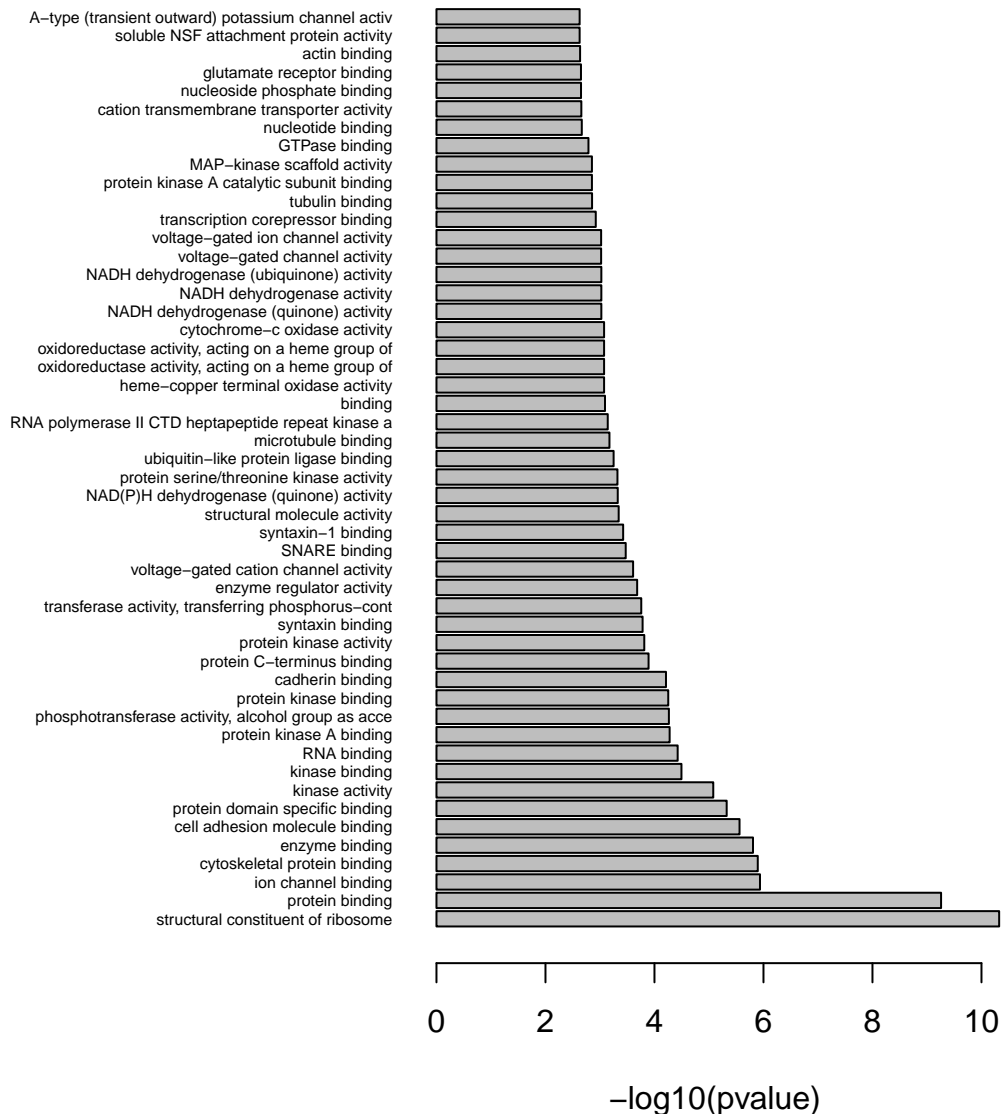

Supplement: Supplementary file 1 [file cells-11-01807-s001.zip › Supplementary_Data/DataS5/SFG_cells_GOKEGGs/SFGBS6_12_vs_SFGBS0_12/pVal_GOstats_MF_Up_pieChart.pdf]

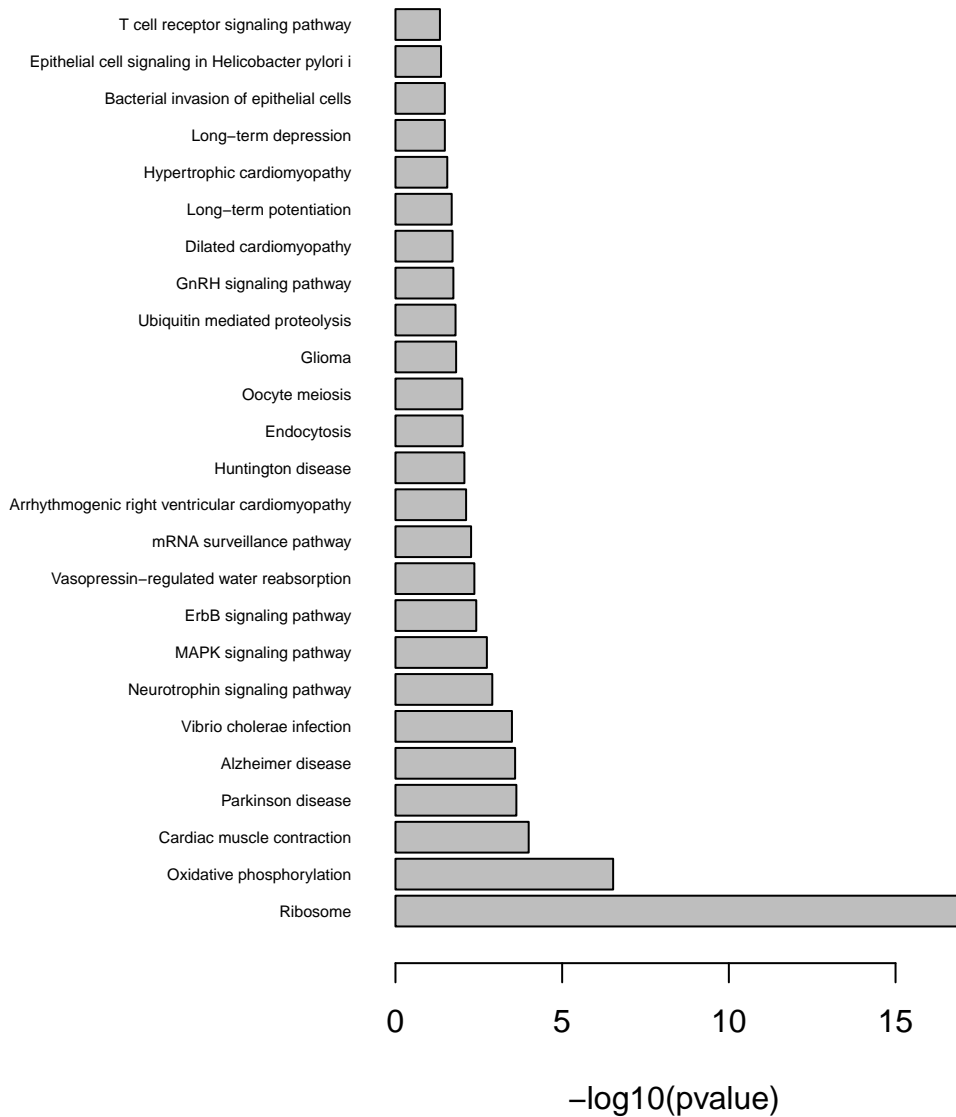

Supplement: Supplementary file 1 [file cells-11-01807-s001.zip › Supplementary_Data/DataS5/SFG_cells_GOKEGGs/SFGBS6_12_vs_SFGBS0_12/pVal_GOstats_kegg_Up.pdf]

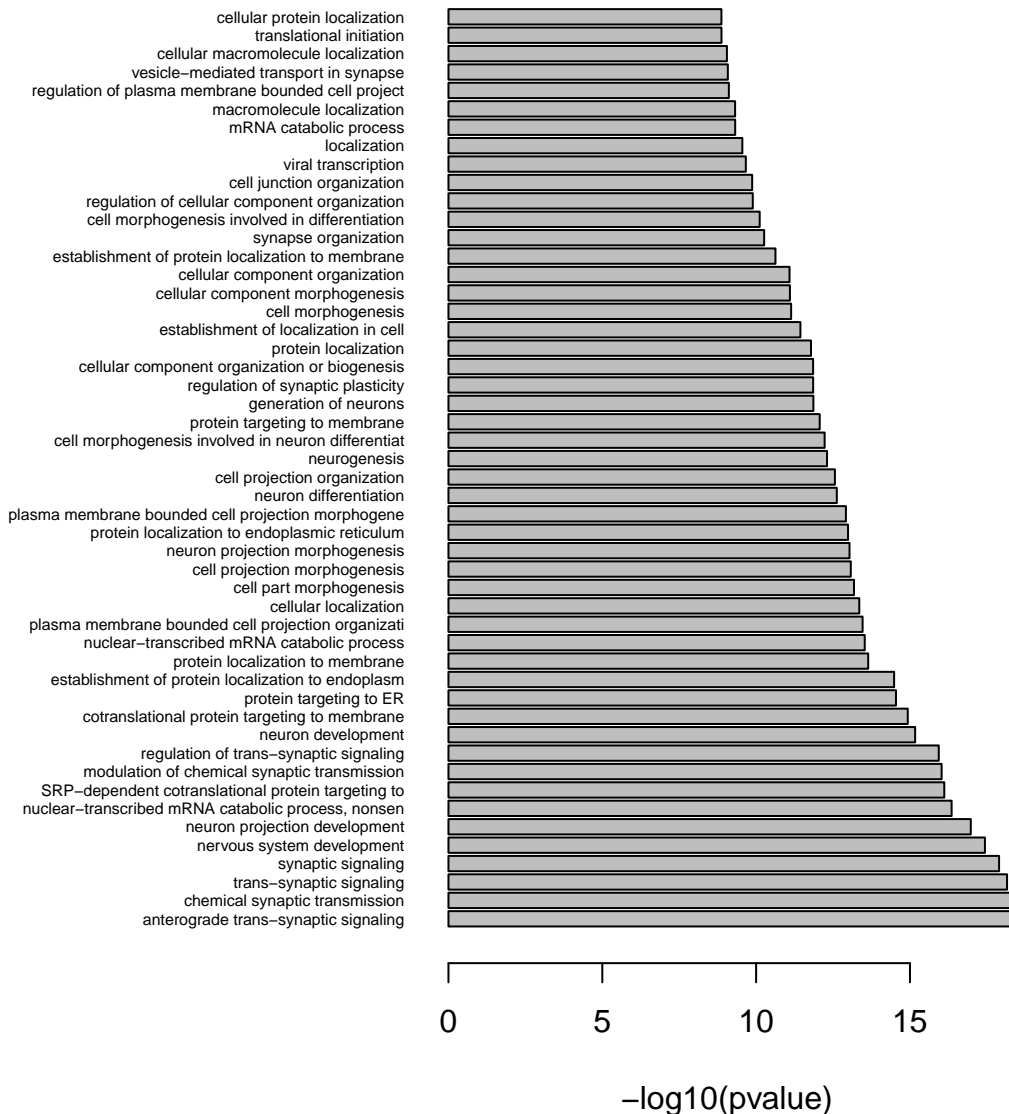

Supplement: Supplementary file 1 [file cells-11-01807-s001.zip › Supplementary_Data/DataS5/SFG_cells_GOKEGGs/SFGBS6_12_vs_SFGBS0_12/pVal_GOstats_BP_Up_pieChart.pdf]

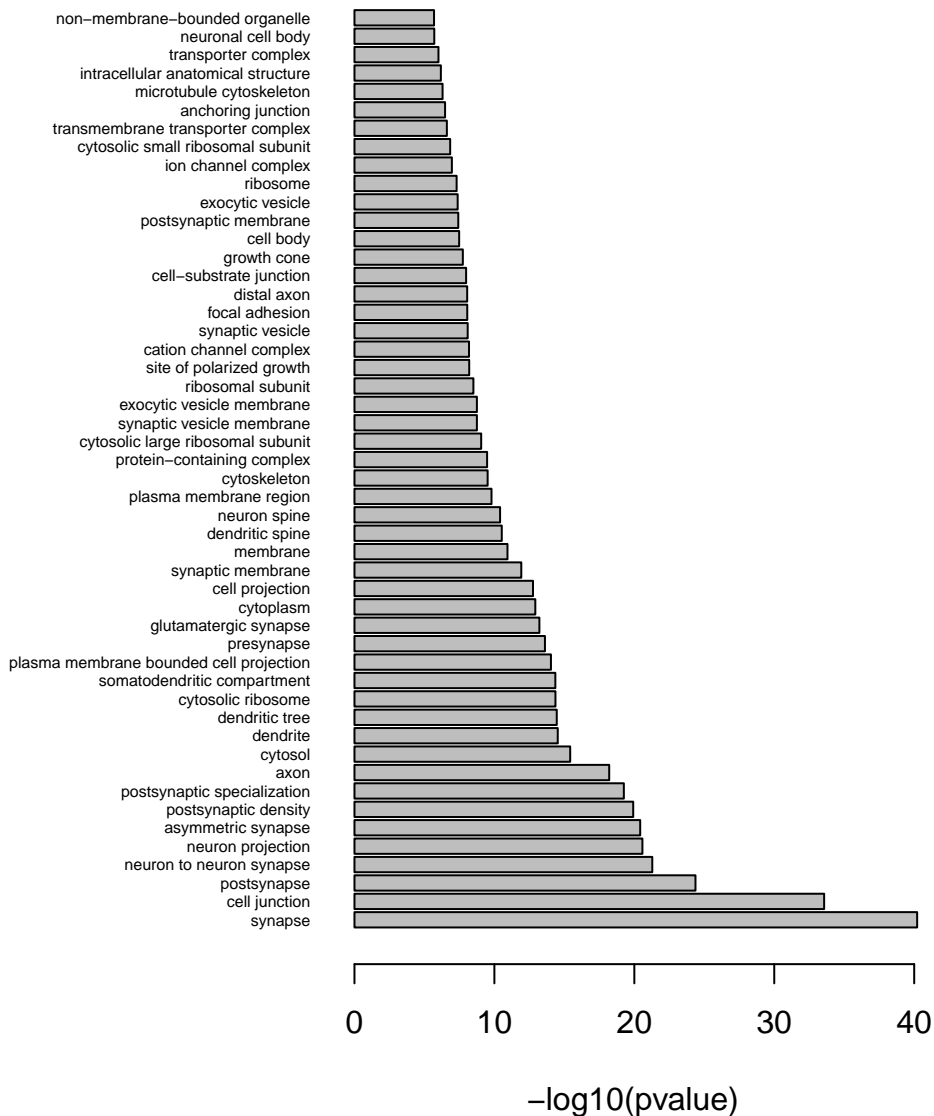

Supplement: Supplementary file 1 [file cells-11-01807-s001.zip › Supplementary_Data/DataS5/SFG_cells_GOKEGGs/SFGBS6_12_vs_SFGBS0_12/pVal_GOstats_CC_Up_pieChart.pdf]

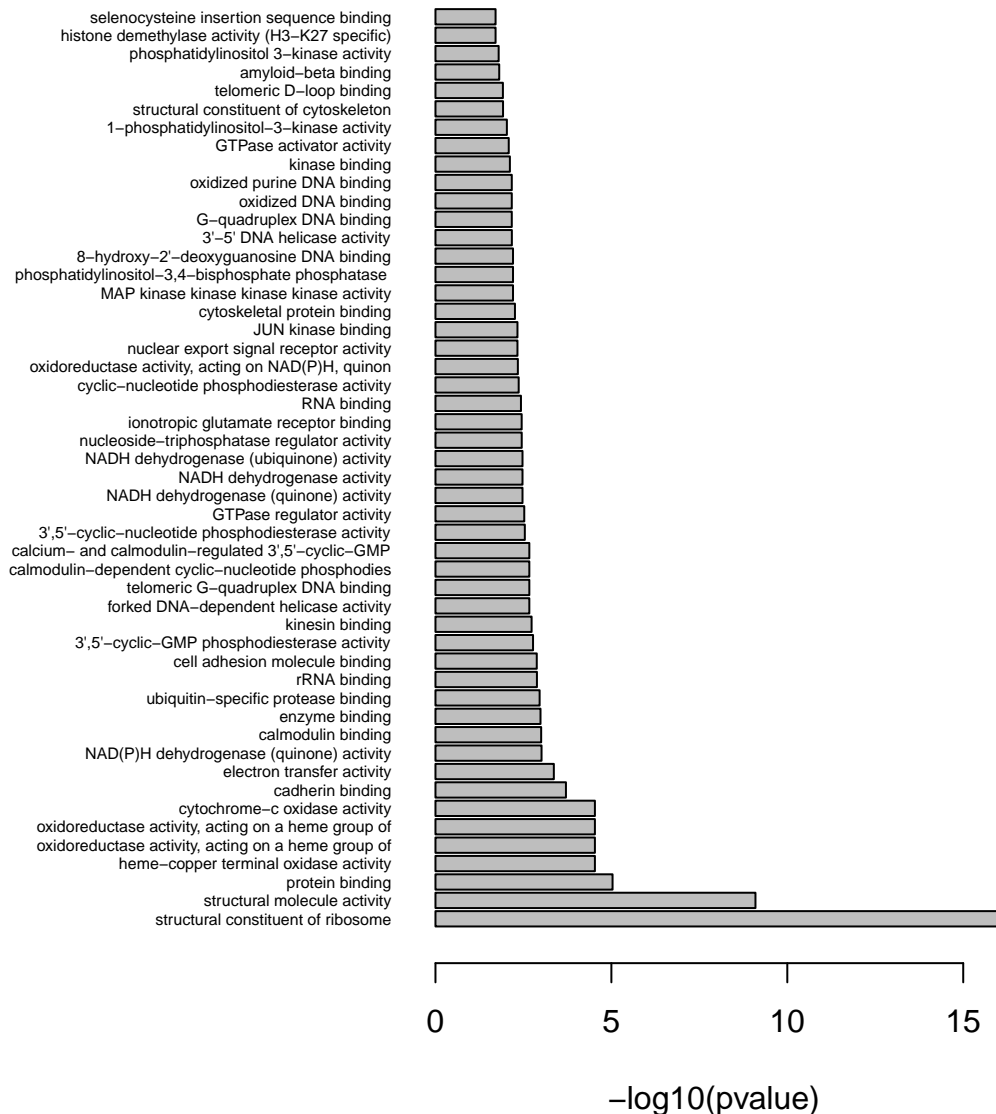

Supplement: Supplementary file 1 [file cells-11-01807-s001.zip › Supplementary_Data/DataS5/SFG_cells_GOKEGGs/SFGBS6_6_vs_SFGBS0_6/pVal_GOstats_MF_Up_pieChart.pdf]

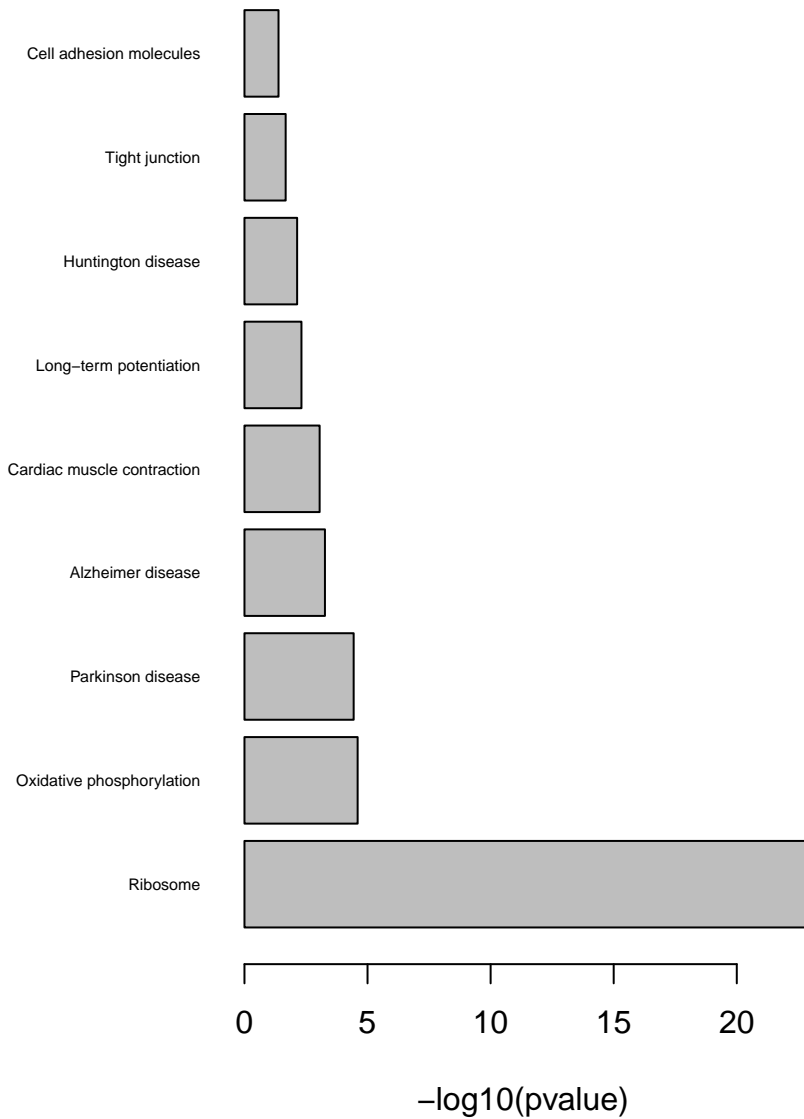

Supplement: Supplementary file 1 [file cells-11-01807-s001.zip › Supplementary_Data/DataS5/SFG_cells_GOKEGGs/SFGBS6_6_vs_SFGBS0_6/pVal_GOstats_kegg_Up.pdf]

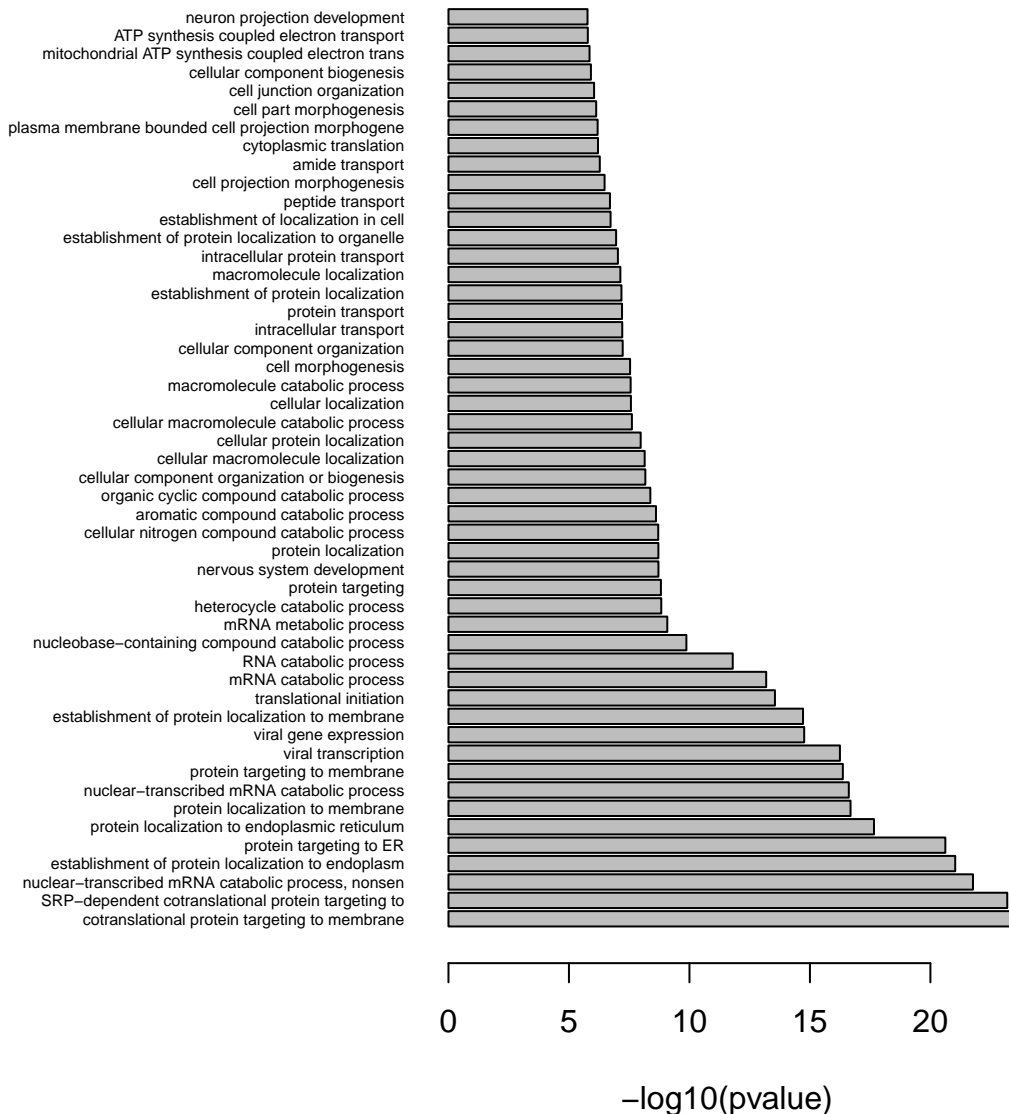

Supplement: Supplementary file 1 [file cells-11-01807-s001.zip › Supplementary_Data/DataS5/SFG_cells_GOKEGGs/SFGBS6_6_vs_SFGBS0_6/pVal_GOstats_BP_Up_pieChart.pdf]

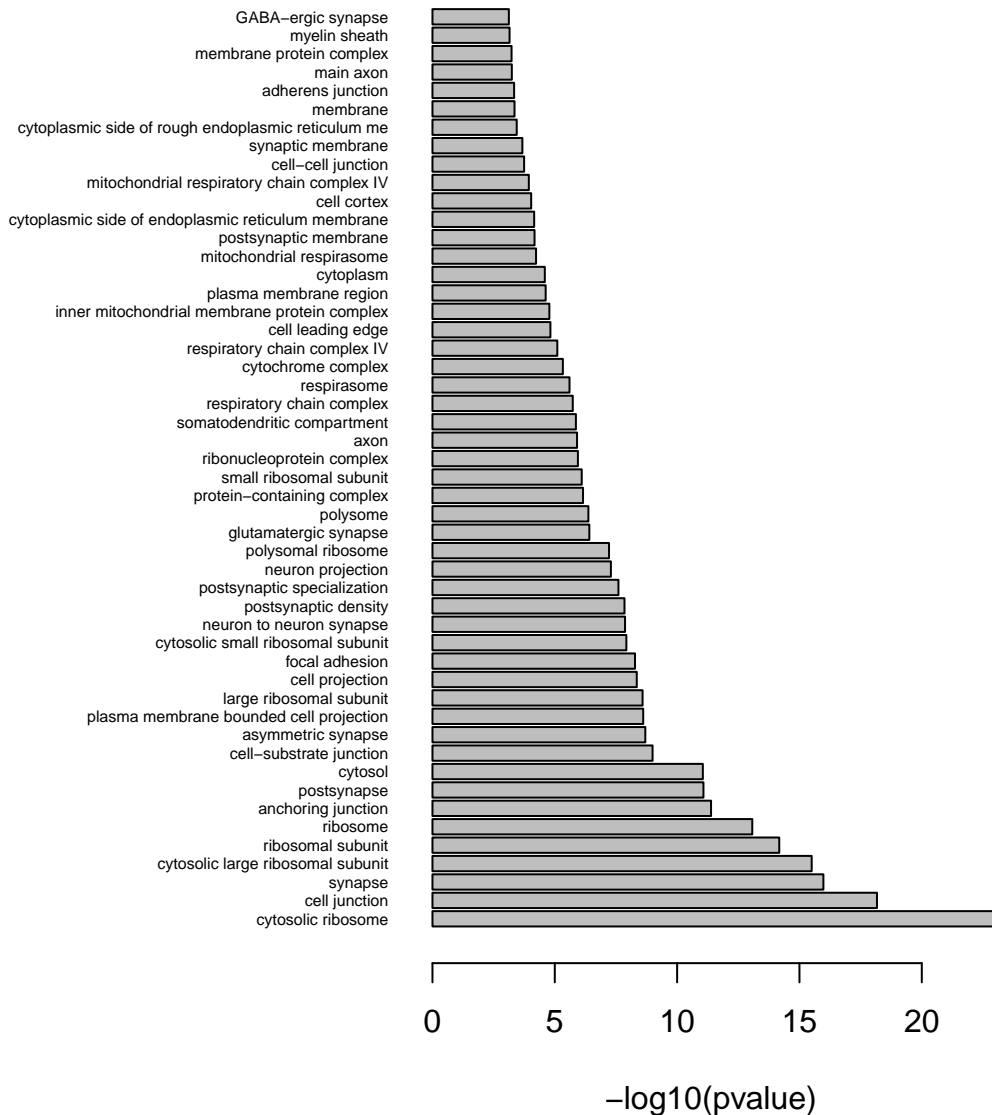

Supplement: Supplementary file 1 [file cells-11-01807-s001.zip › Supplementary_Data/DataS5/SFG_cells_GOKEGGs/SFGBS6_6_vs_SFGBS0_6/pVal_GOstats_CC_Up_pieChart.pdf]

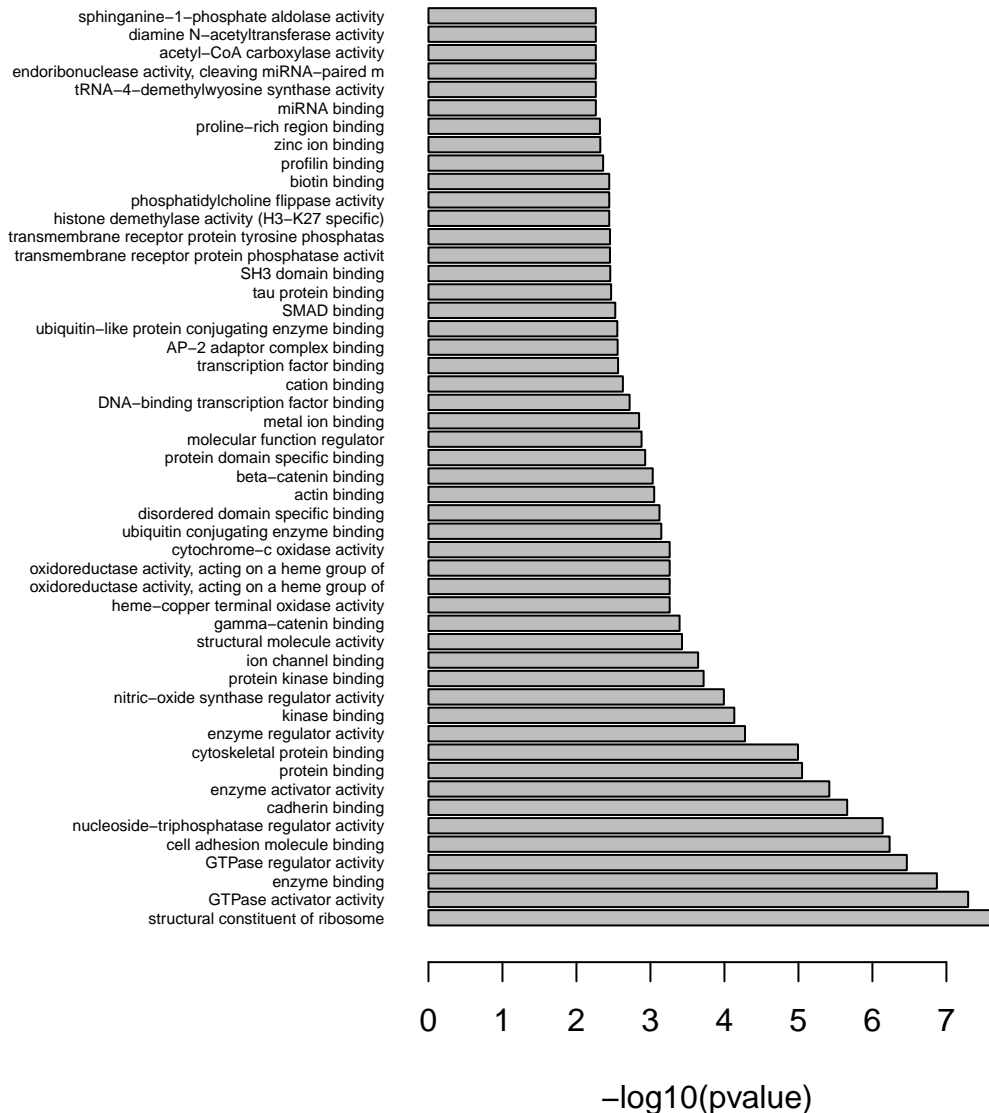

Supplement: Supplementary file 1 [file cells-11-01807-s001.zip › Supplementary_Data/DataS5/SFG_cells_GOKEGGs/SFGBS6_2_vs_SFGBS0_2/pVal_GOstats_MF_Up_pieChart.pdf]

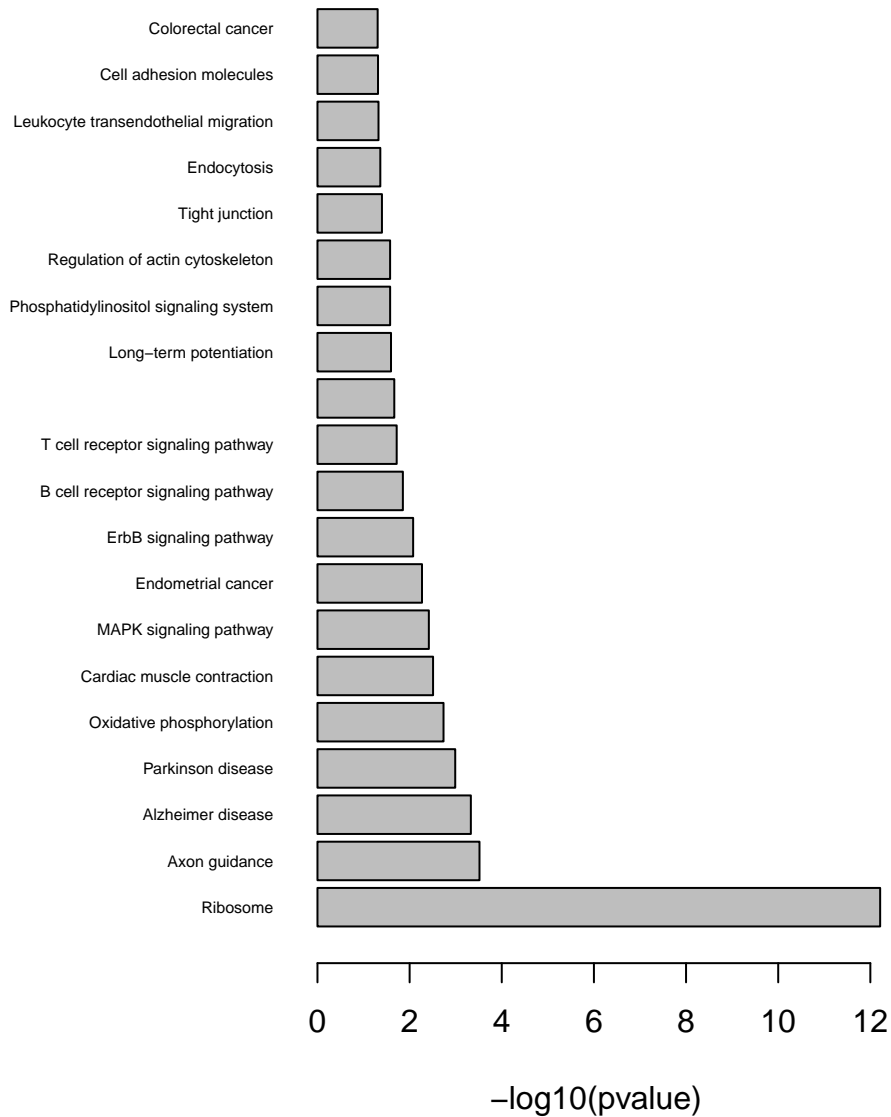

Supplement: Supplementary file 1 [file cells-11-01807-s001.zip › Supplementary_Data/DataS5/SFG_cells_GOKEGGs/SFGBS6_2_vs_SFGBS0_2/pVal_GOstats_kegg_Up.pdf]

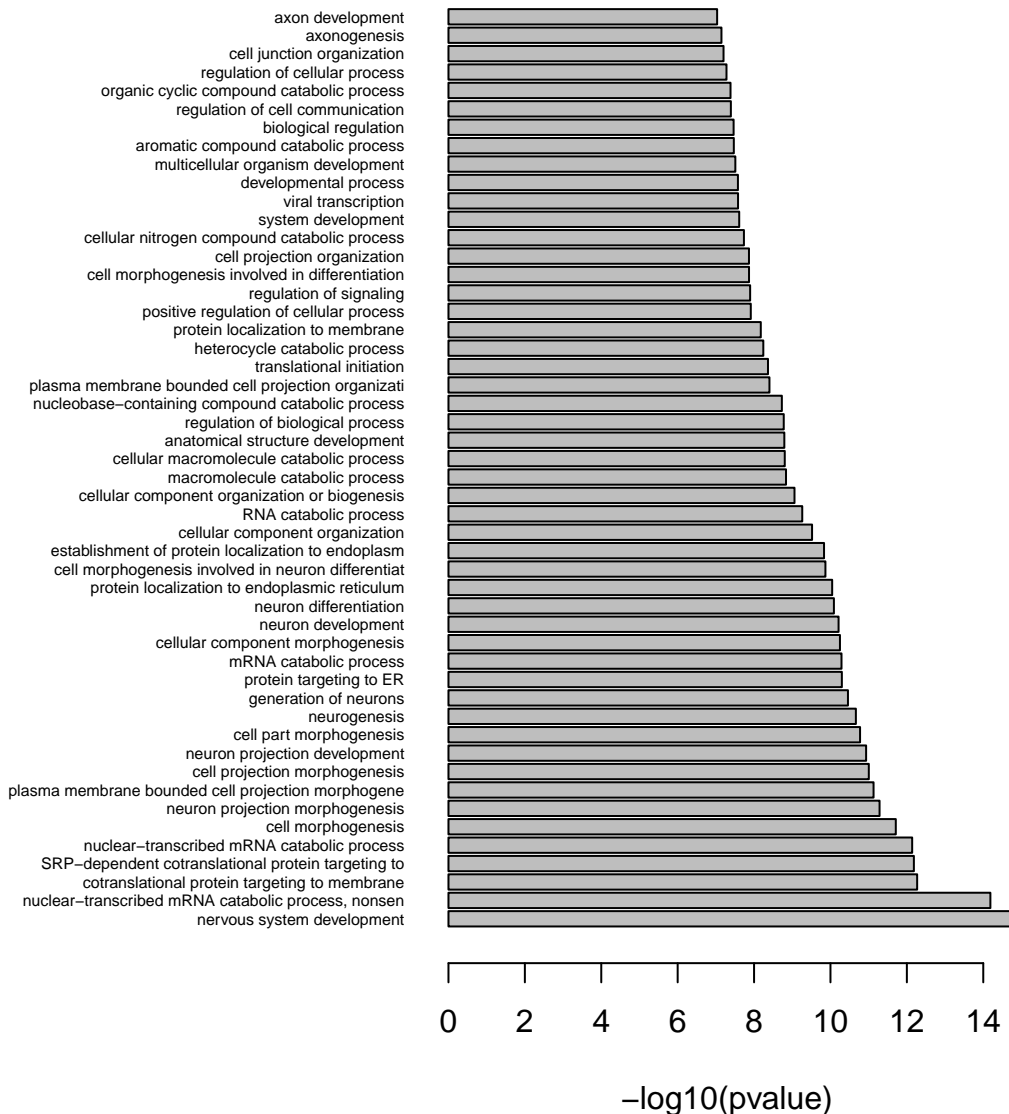

Supplement: Supplementary file 1 [file cells-11-01807-s001.zip › Supplementary_Data/DataS5/SFG_cells_GOKEGGs/SFGBS6_2_vs_SFGBS0_2/pVal_GOstats_BP_Up_pieChart.pdf]

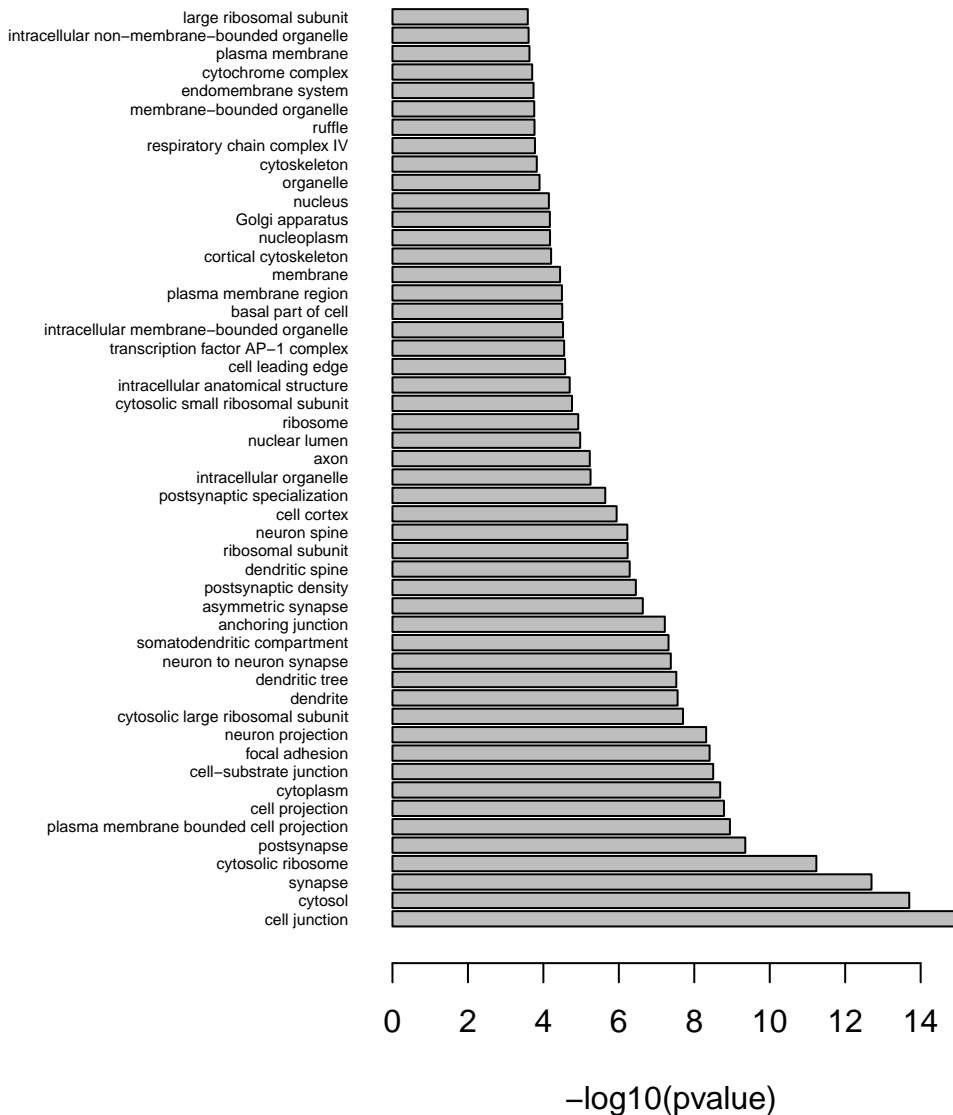

Supplement: Supplementary file 1 [file cells-11-01807-s001.zip › Supplementary_Data/DataS5/SFG_cells_GOKEGGs/SFGBS6_2_vs_SFGBS0_2/pVal_GOstats_CC_Up_pieChart.pdf]

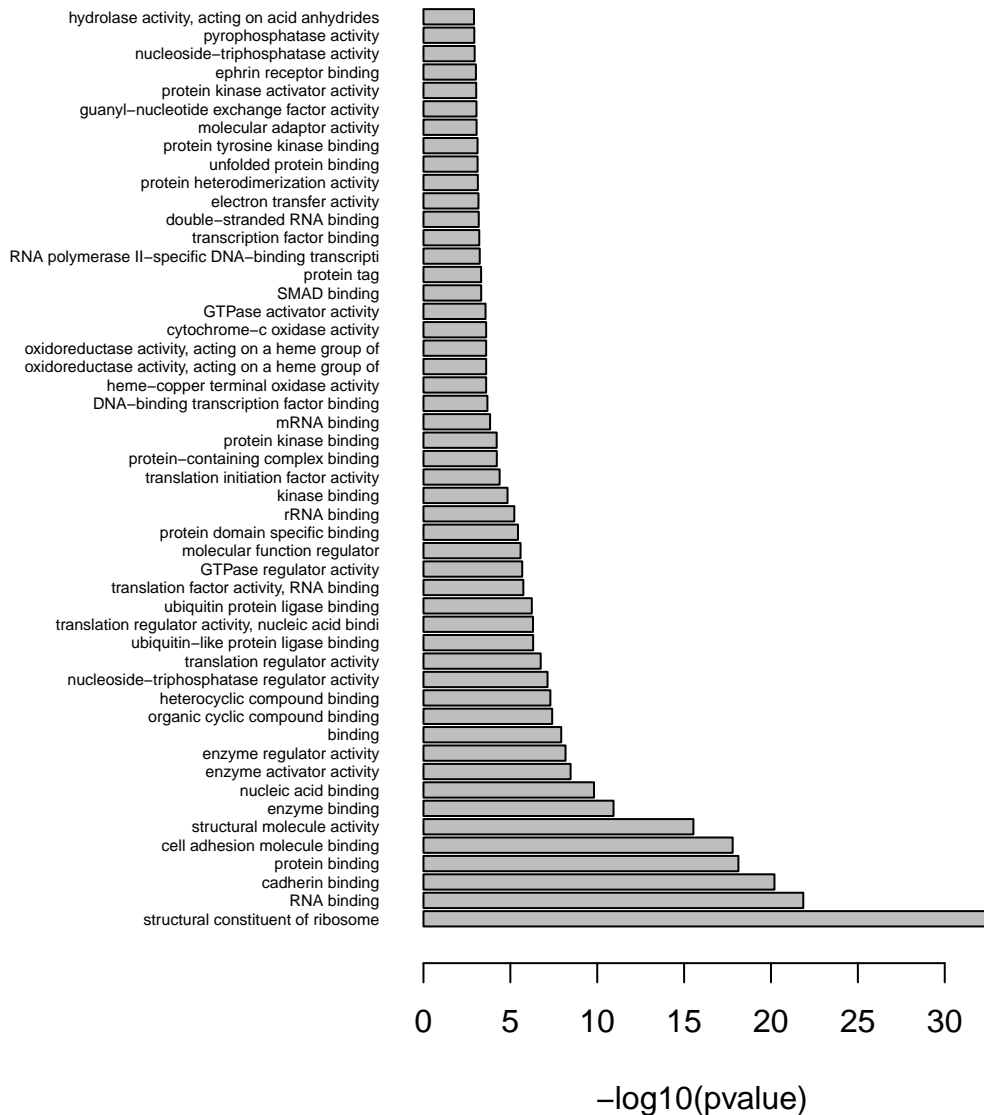

Supplement: Supplementary file 1 [file cells-11-01807-s001.zip › Supplementary_Data/DataS5/SFG_cells_GOKEGGs/SFGBS6_24_vs_SFGBS0_24/pVal_GOstats_MF_Up_pieChart.pdf]

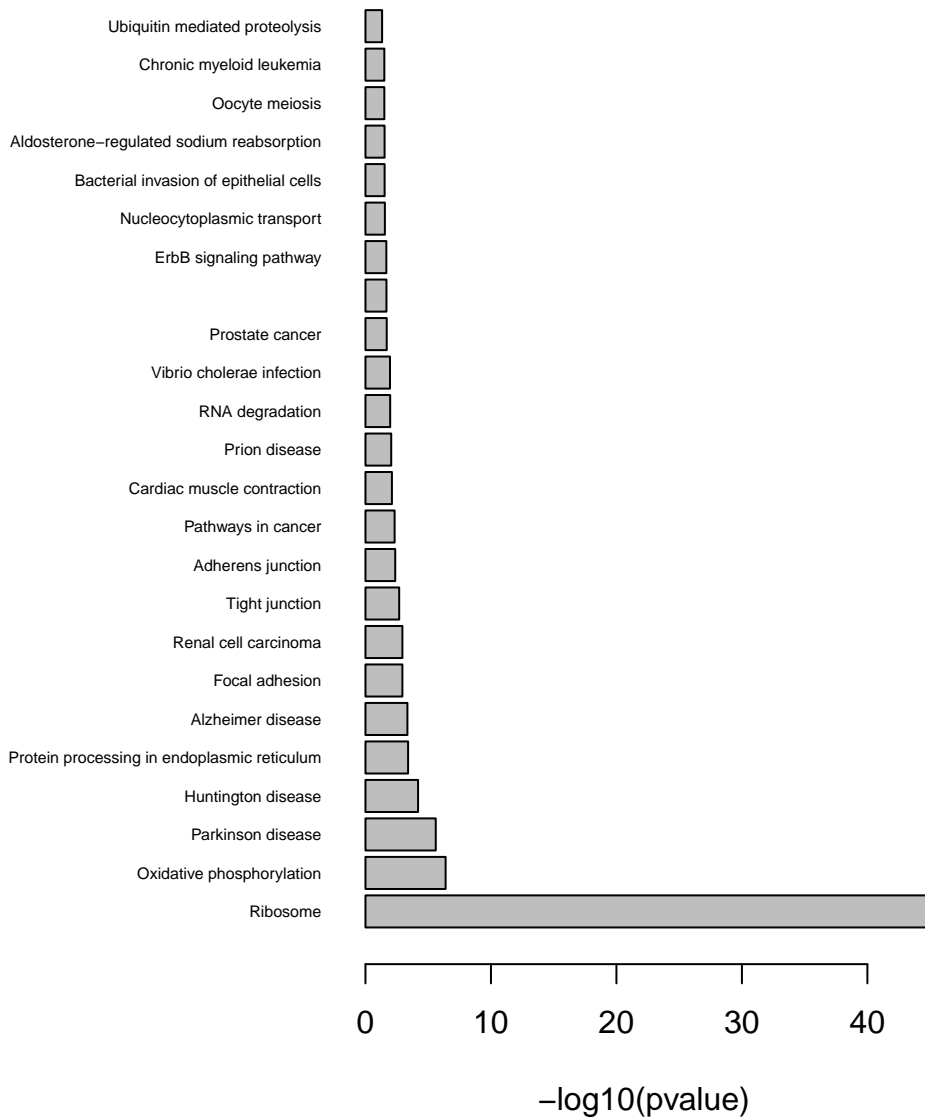

Supplement: Supplementary file 1 [file cells-11-01807-s001.zip › Supplementary_Data/DataS5/SFG_cells_GOKEGGs/SFGBS6_24_vs_SFGBS0_24/pVal_GOstats_kegg_Up.pdf]

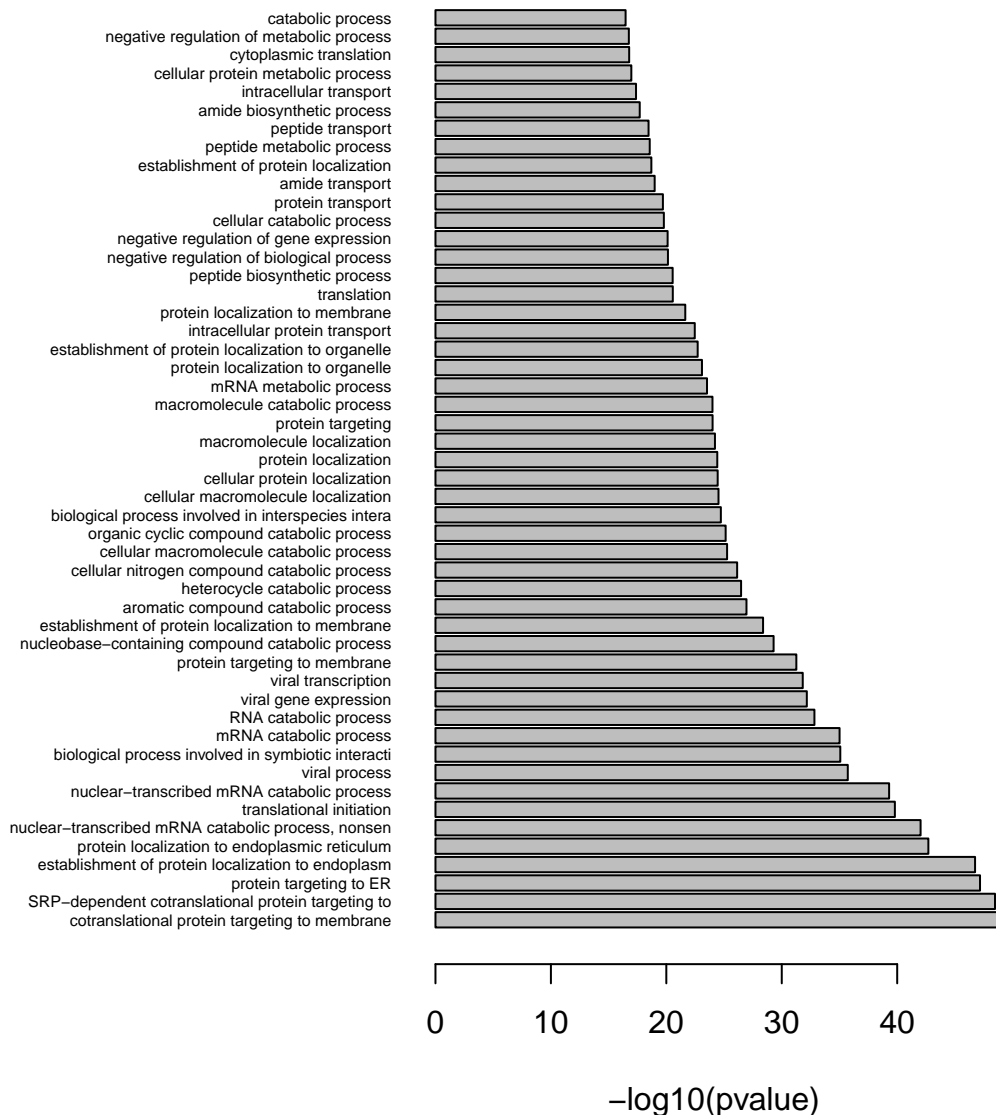

Supplement: Supplementary file 1 [file cells-11-01807-s001.zip › Supplementary_Data/DataS5/SFG_cells_GOKEGGs/SFGBS6_24_vs_SFGBS0_24/pVal_GOstats_BP_Up_pieChart.pdf]

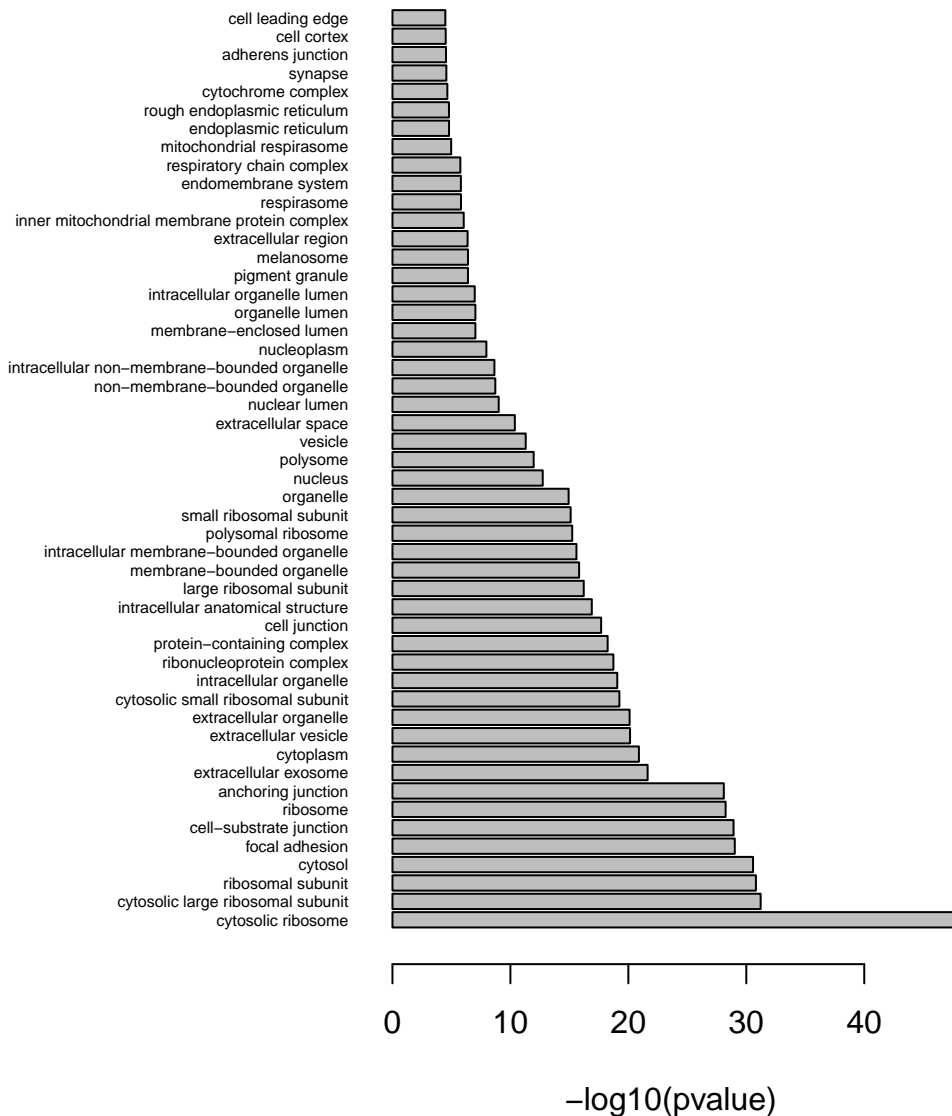

Supplement: Supplementary file 1 [file cells-11-01807-s001.zip › Supplementary_Data/DataS5/SFG_cells_GOKEGGs/SFGBS6_24_vs_SFGBS0_24/pVal_GOstats_CC_Up_pieChart.pdf]

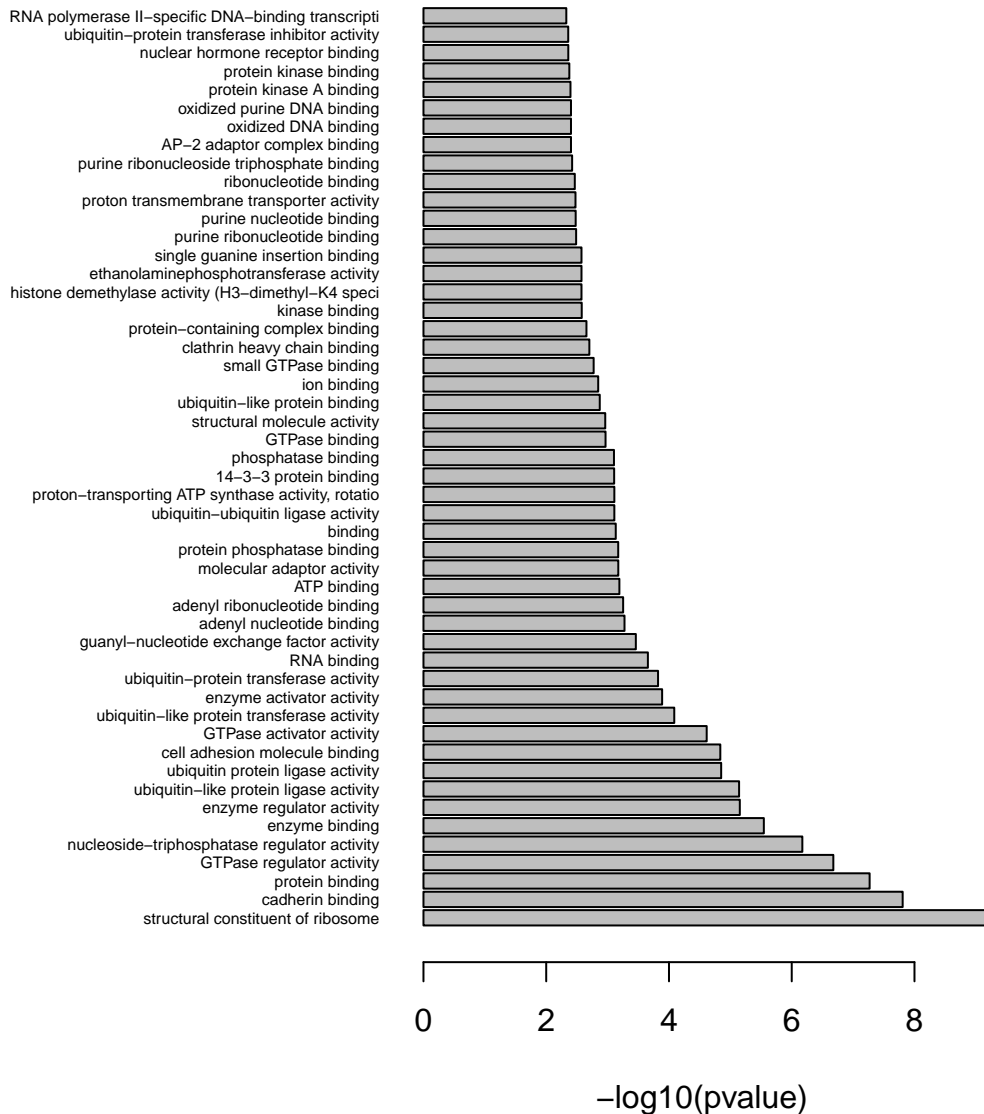

Supplement: Supplementary file 1 [file cells-11-01807-s001.zip › Supplementary_Data/DataS5/SFG_cells_GOKEGGs/SFGBS6_4_vs_SFGBS0_4/pVal_GOstats_MF_Up_pieChart.pdf]

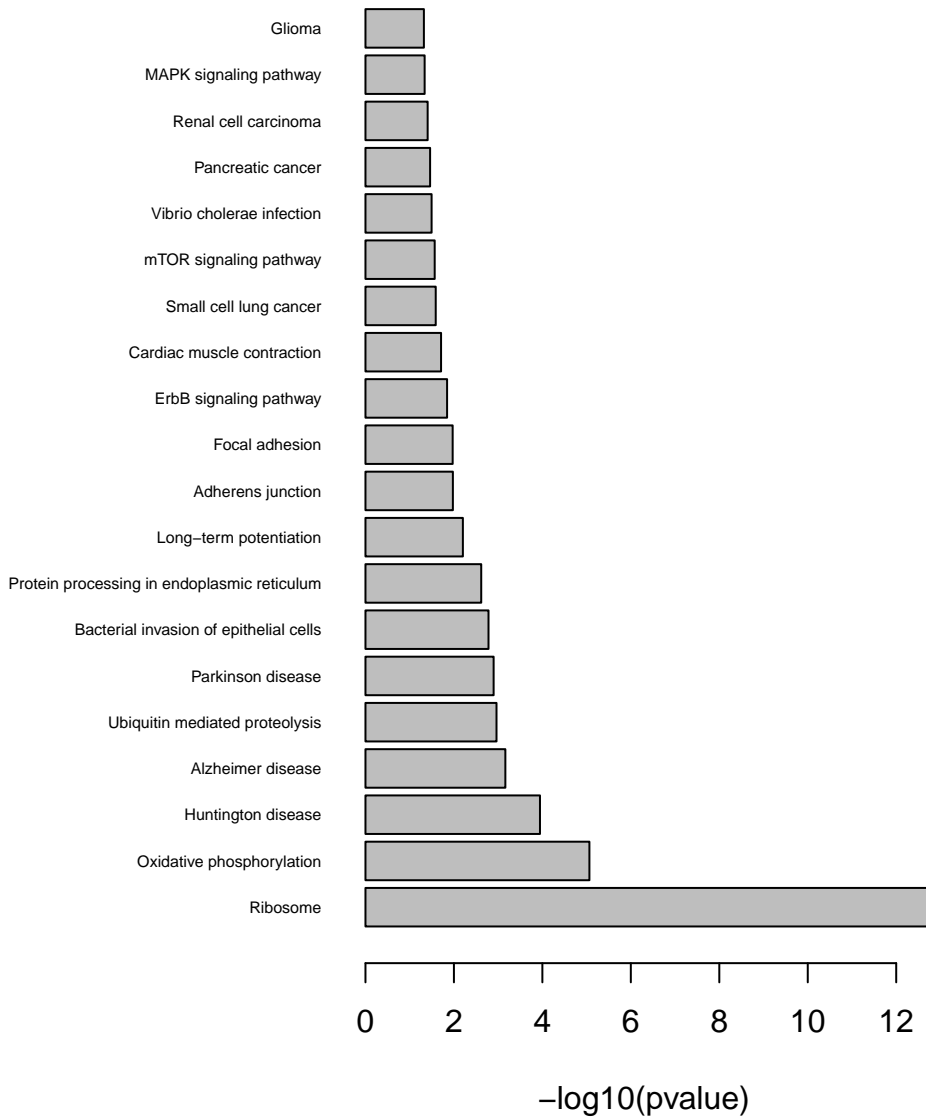

Supplement: Supplementary file 1 [file cells-11-01807-s001.zip › Supplementary_Data/DataS5/SFG_cells_GOKEGGs/SFGBS6_4_vs_SFGBS0_4/pVal_GOstats_kegg_Up.pdf]

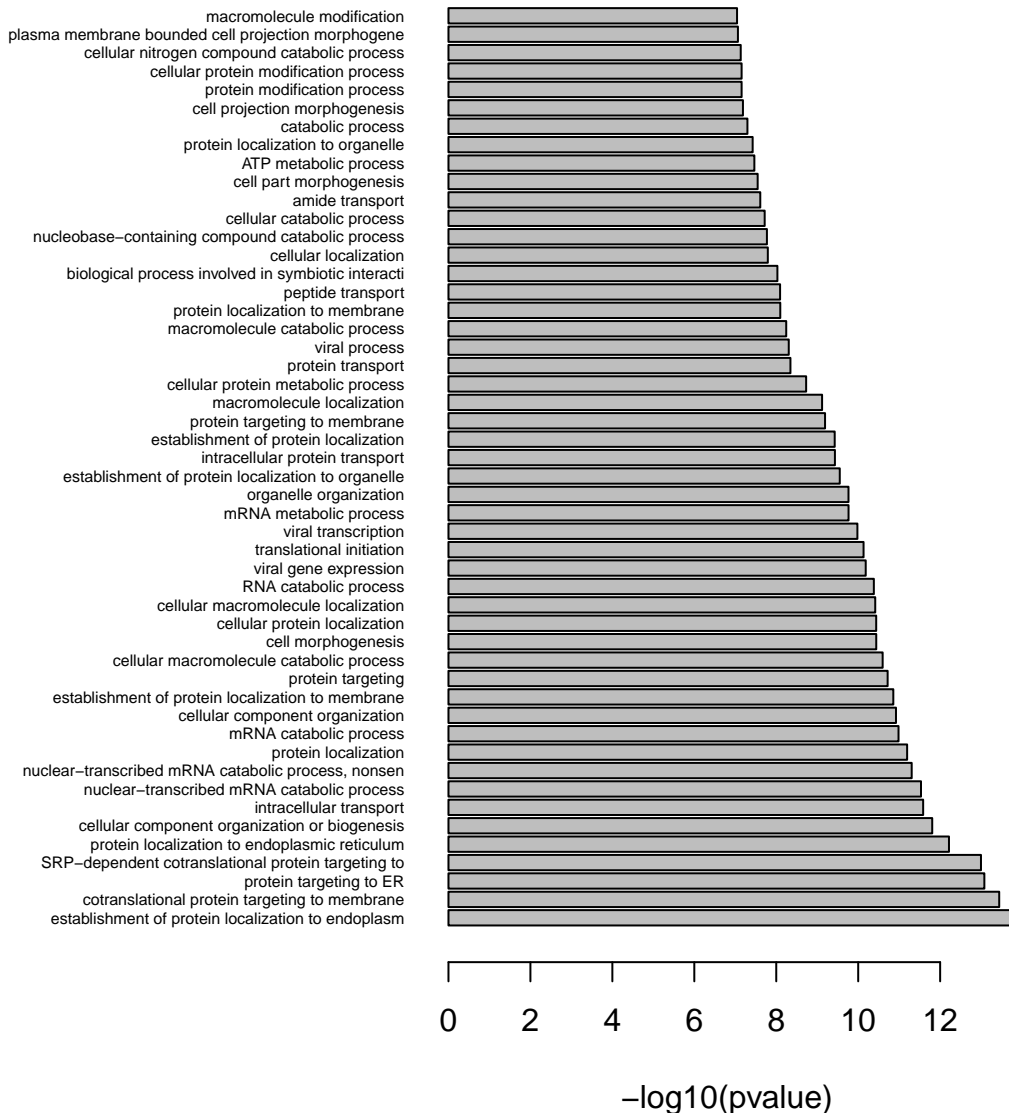

Supplement: Supplementary file 1 [file cells-11-01807-s001.zip › Supplementary_Data/DataS5/SFG_cells_GOKEGGs/SFGBS6_4_vs_SFGBS0_4/pVal_GOstats_BP_Up_pieChart.pdf]

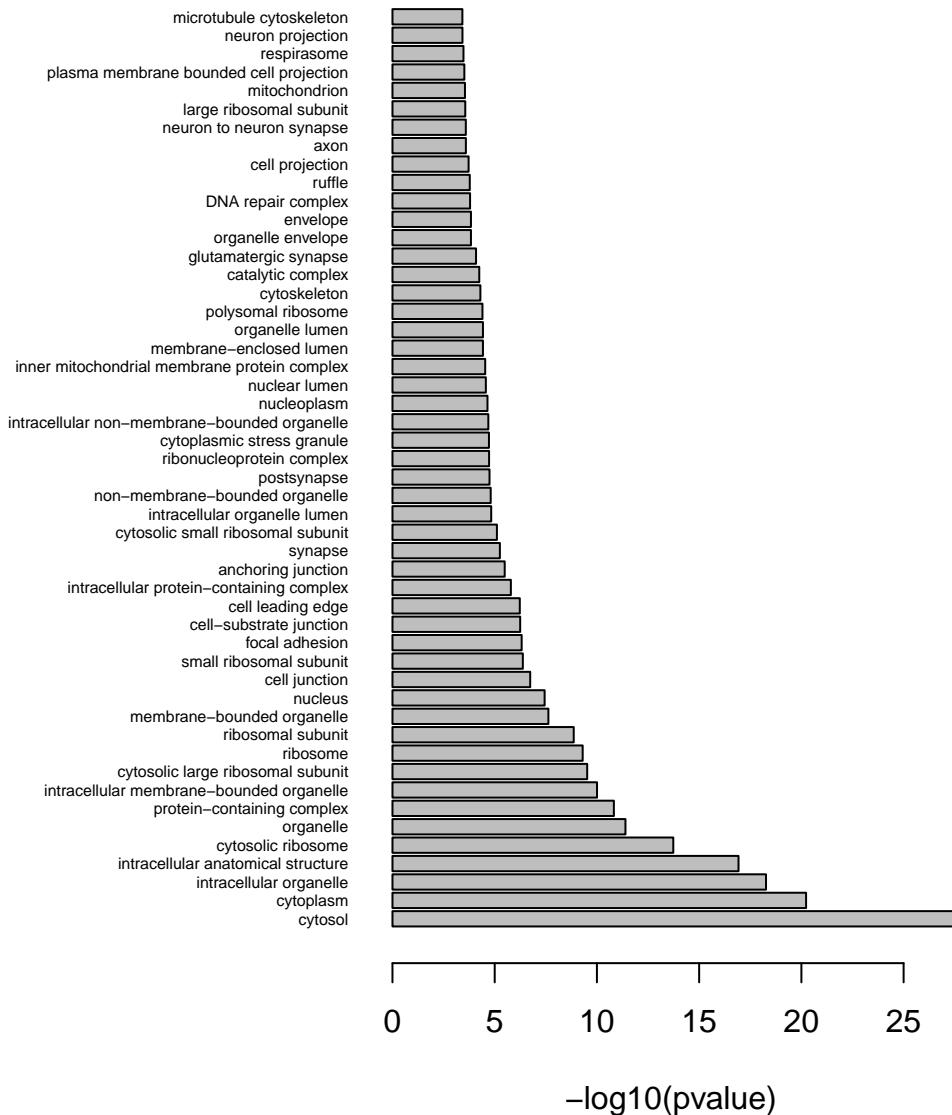

Supplement: Supplementary file 1 [file cells-11-01807-s001.zip › Supplementary_Data/DataS5/SFG_cells_GOKEGGs/SFGBS6_4_vs_SFGBS0_4/pVal_GOstats_CC_Up_pieChart.pdf]

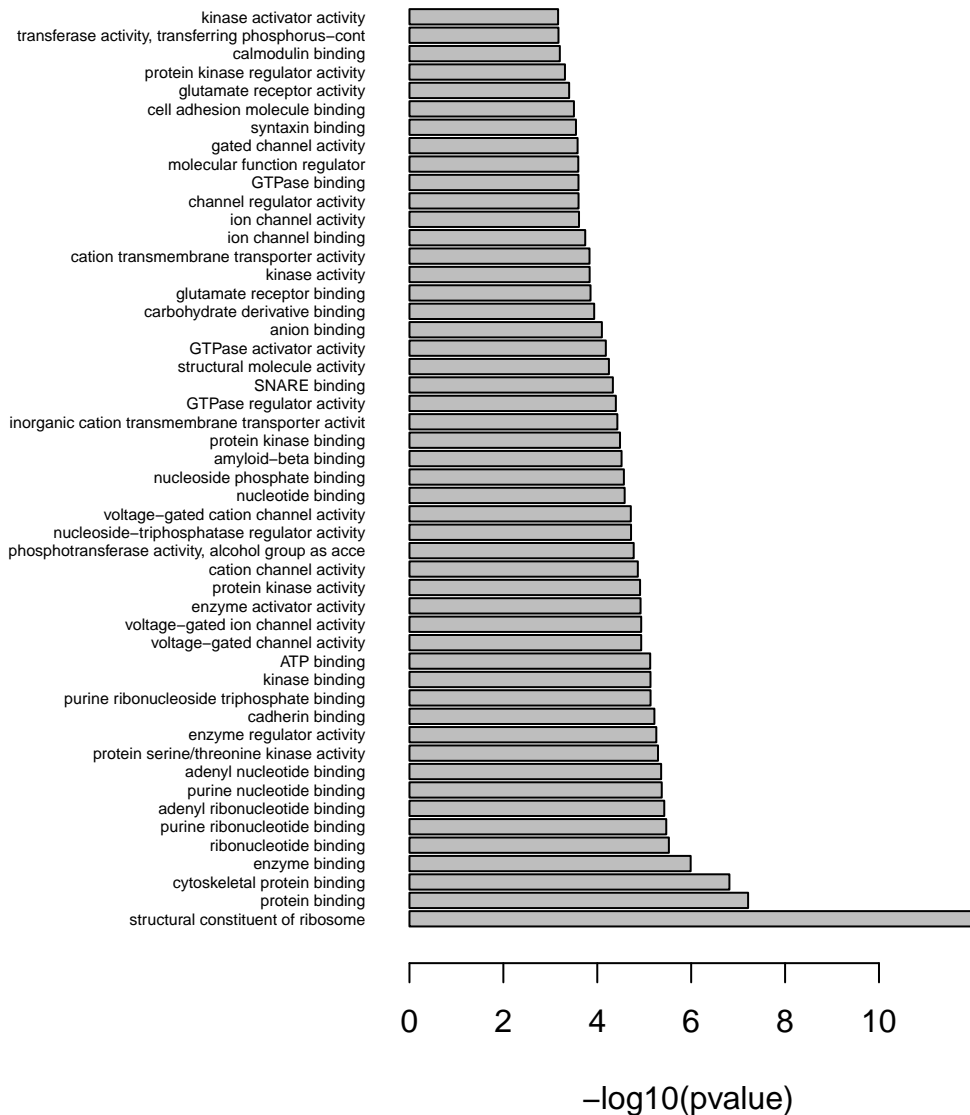

Supplement: Supplementary file 1 [file cells-11-01807-s001.zip › Supplementary_Data/DataS5/SFG_cells_GOKEGGs/SFGBS6_3_vs_SFGBS0_3/pVal_GOstats_MF_Up_pieChart.pdf]

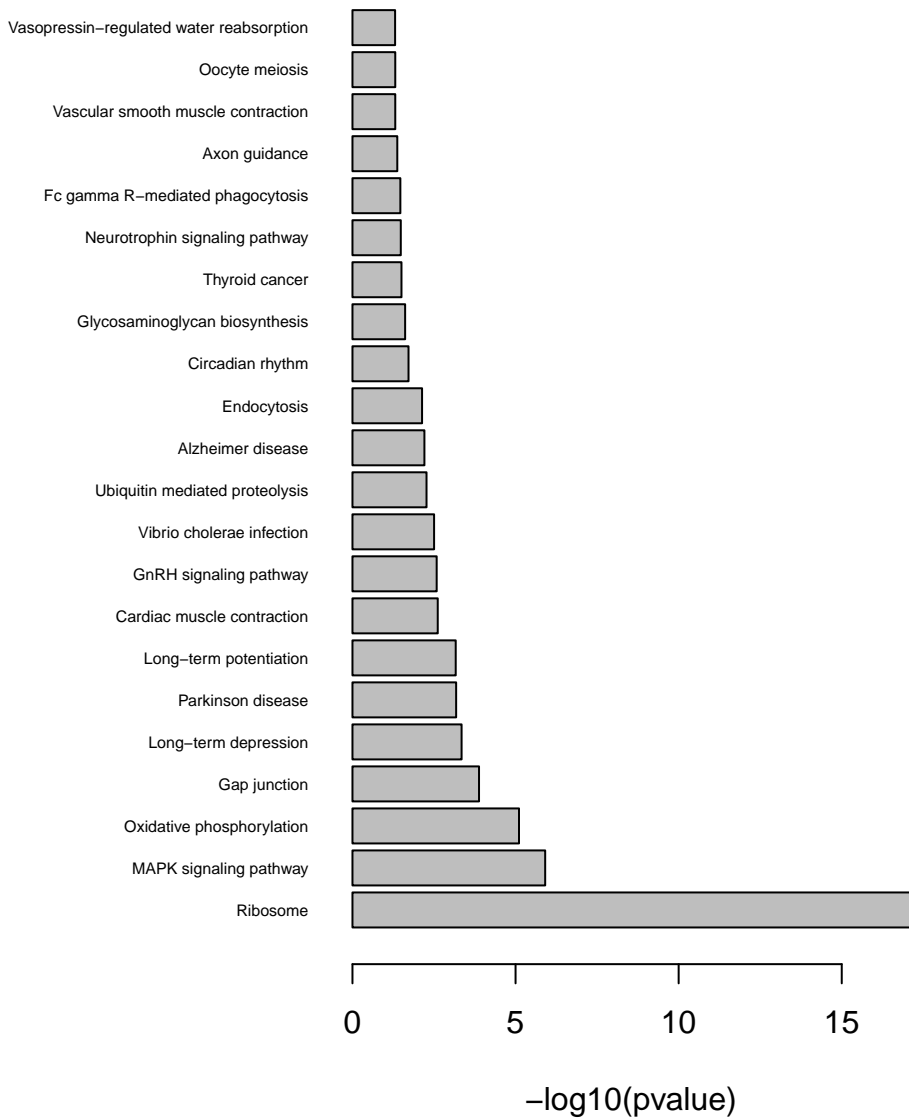

Supplement: Supplementary file 1 [file cells-11-01807-s001.zip › Supplementary_Data/DataS5/SFG_cells_GOKEGGs/SFGBS6_3_vs_SFGBS0_3/pVal_GOstats_kegg_Up.pdf]

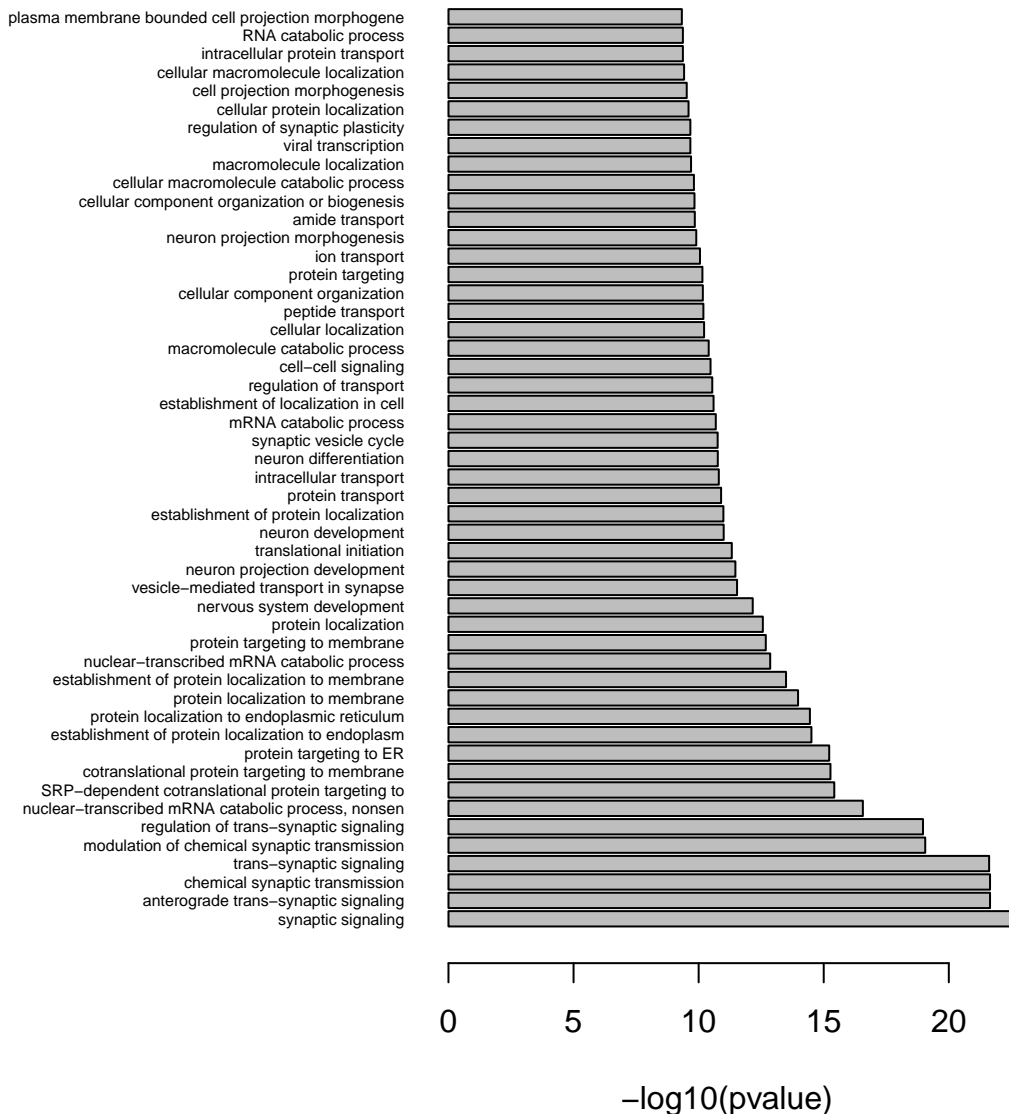

Supplement: Supplementary file 1 [file cells-11-01807-s001.zip › Supplementary_Data/DataS5/SFG_cells_GOKEGGs/SFGBS6_3_vs_SFGBS0_3/pVal_GOstats_BP_Up_pieChart.pdf]

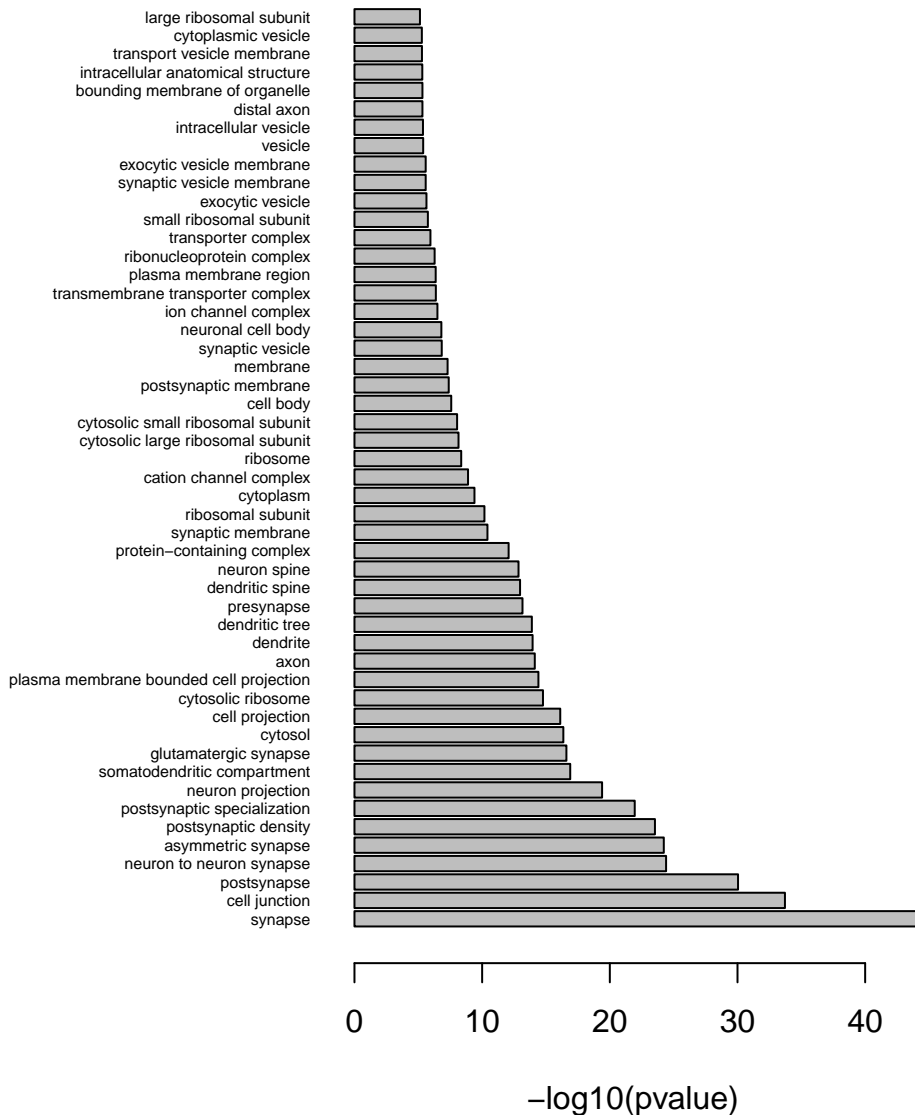

Supplement: Supplementary file 1 [file cells-11-01807-s001.zip › Supplementary_Data/DataS5/SFG_cells_GOKEGGs/SFGBS6_3_vs_SFGBS0_3/pVal_GOstats_CC_Up_pieChart.pdf]

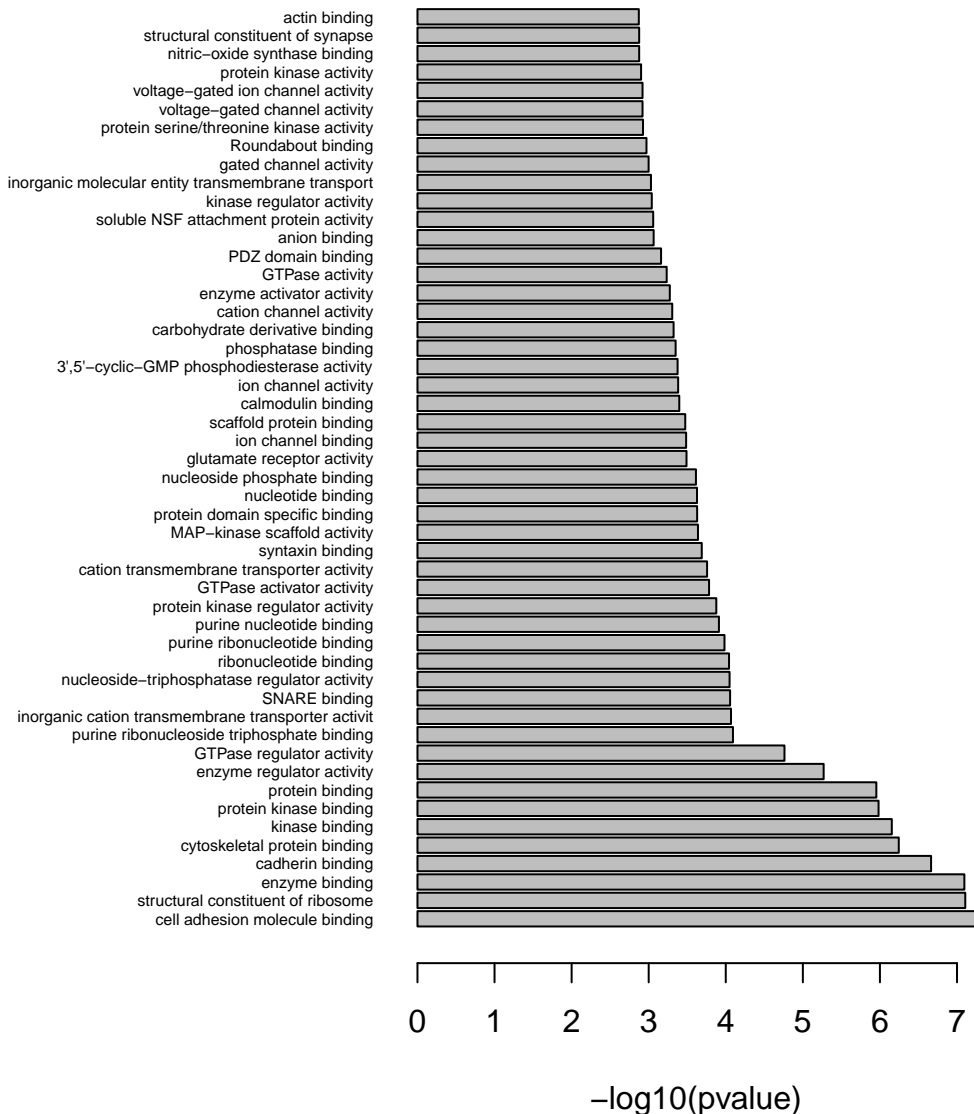

Supplement: Supplementary file 1 [file cells-11-01807-s001.zip › Supplementary_Data/DataS5/SFG_cells_GOKEGGs/SFGBS6_8_vs_SFGBS0_8/pVal_GOstats_MF_Up_pieChart.pdf]

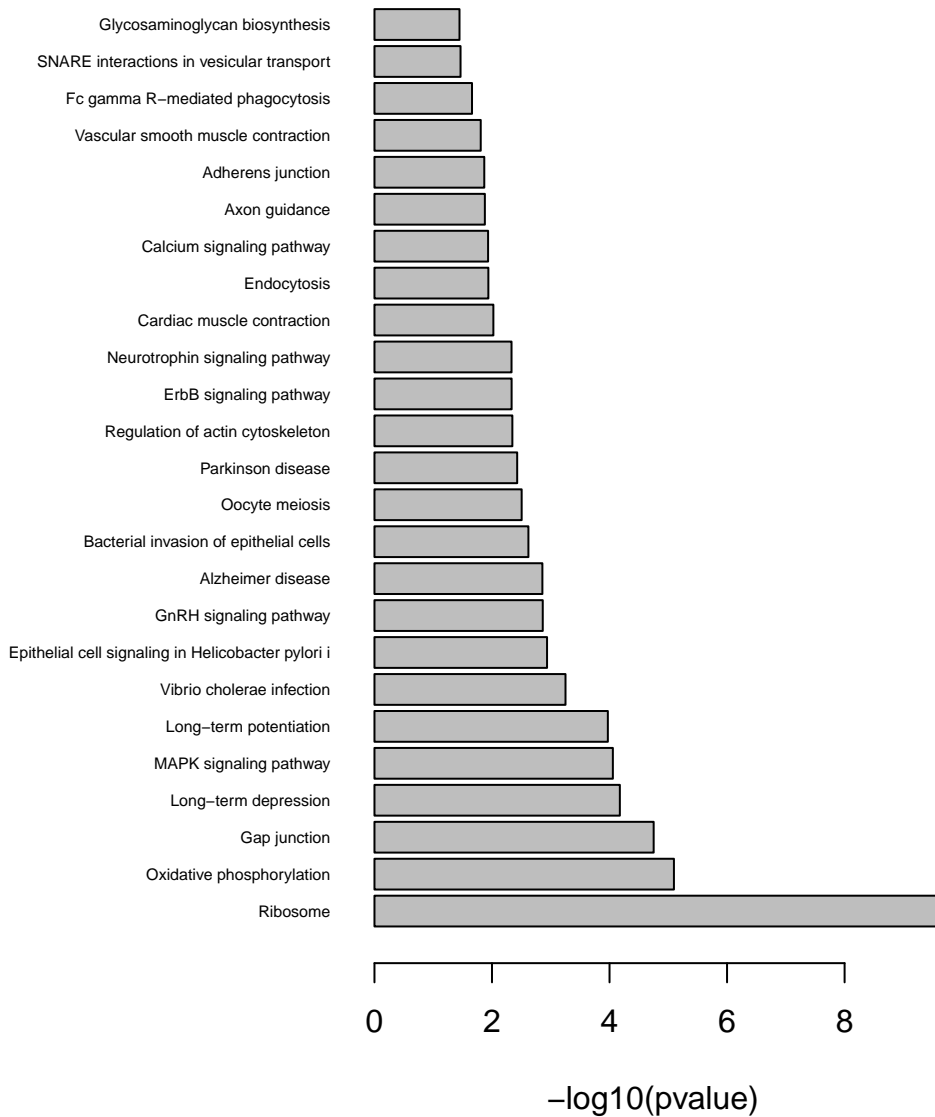

Supplement: Supplementary file 1 [file cells-11-01807-s001.zip › Supplementary_Data/DataS5/SFG_cells_GOKEGGs/SFGBS6_8_vs_SFGBS0_8/pVal_GOstats_kegg_Up.pdf]

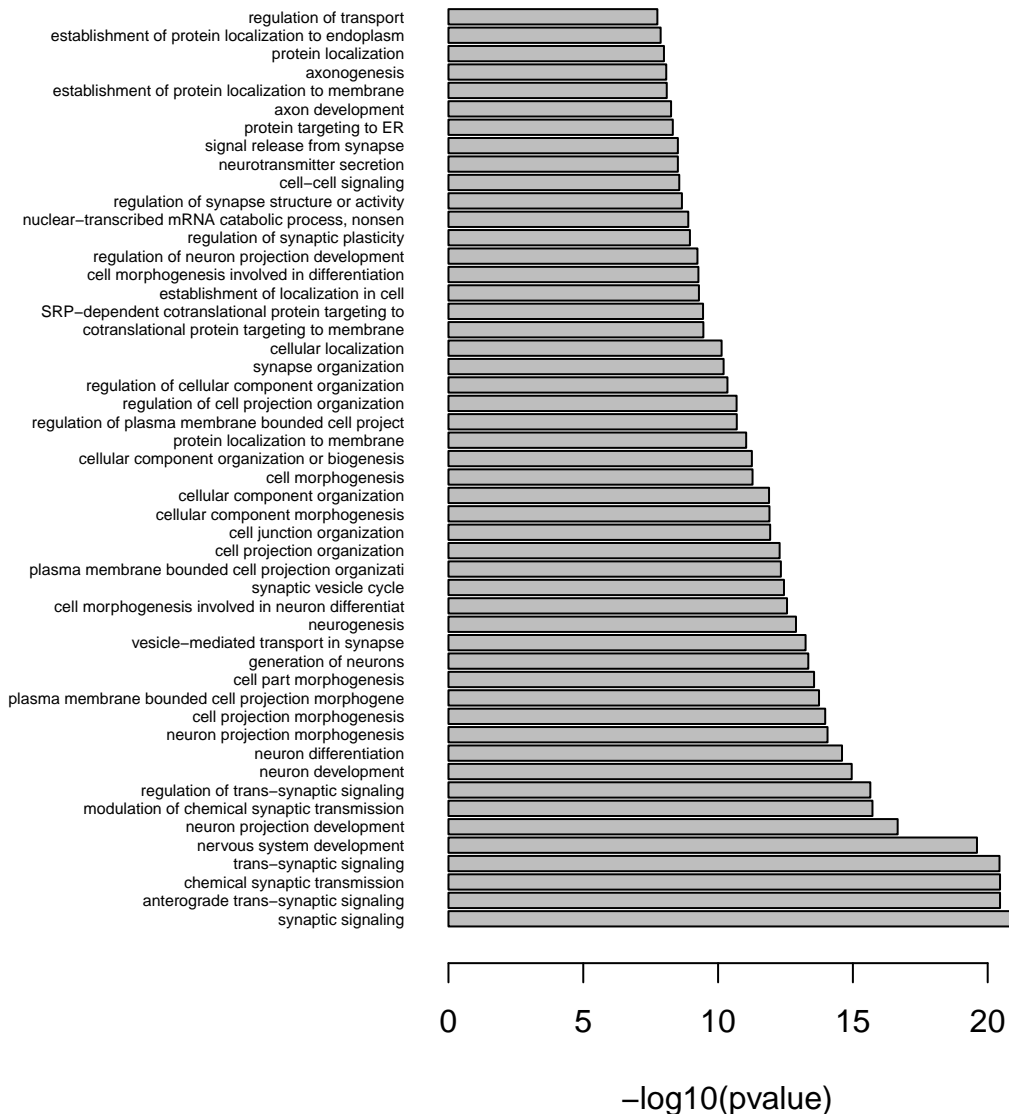

Supplement: Supplementary file 1 [file cells-11-01807-s001.zip › Supplementary_Data/DataS5/SFG_cells_GOKEGGs/SFGBS6_8_vs_SFGBS0_8/pVal_GOstats_BP_Up_pieChart.pdf]

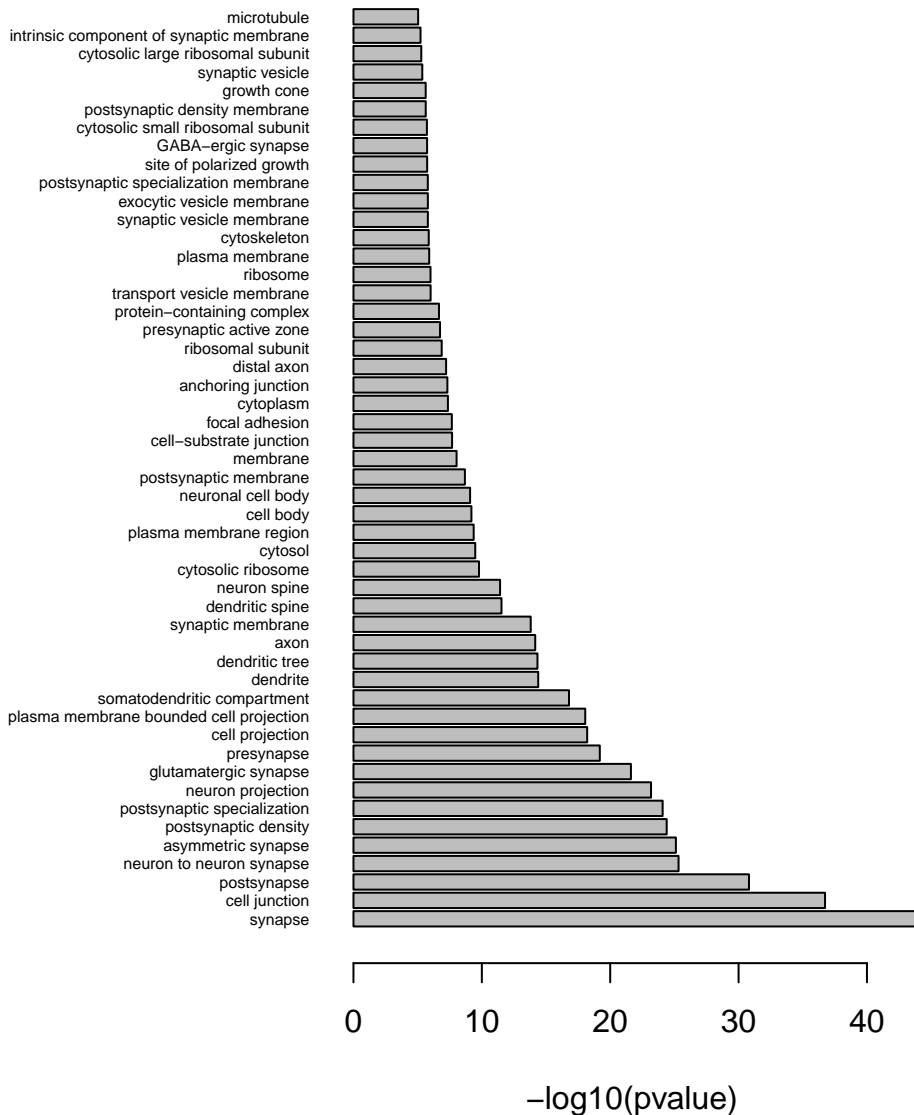

Supplement: Supplementary file 1 [file cells-11-01807-s001.zip › Supplementary_Data/DataS5/SFG_cells_GOKEGGs/SFGBS6_8_vs_SFGBS0_8/pVal_GOstats_CC_Up_pieChart.pdf]

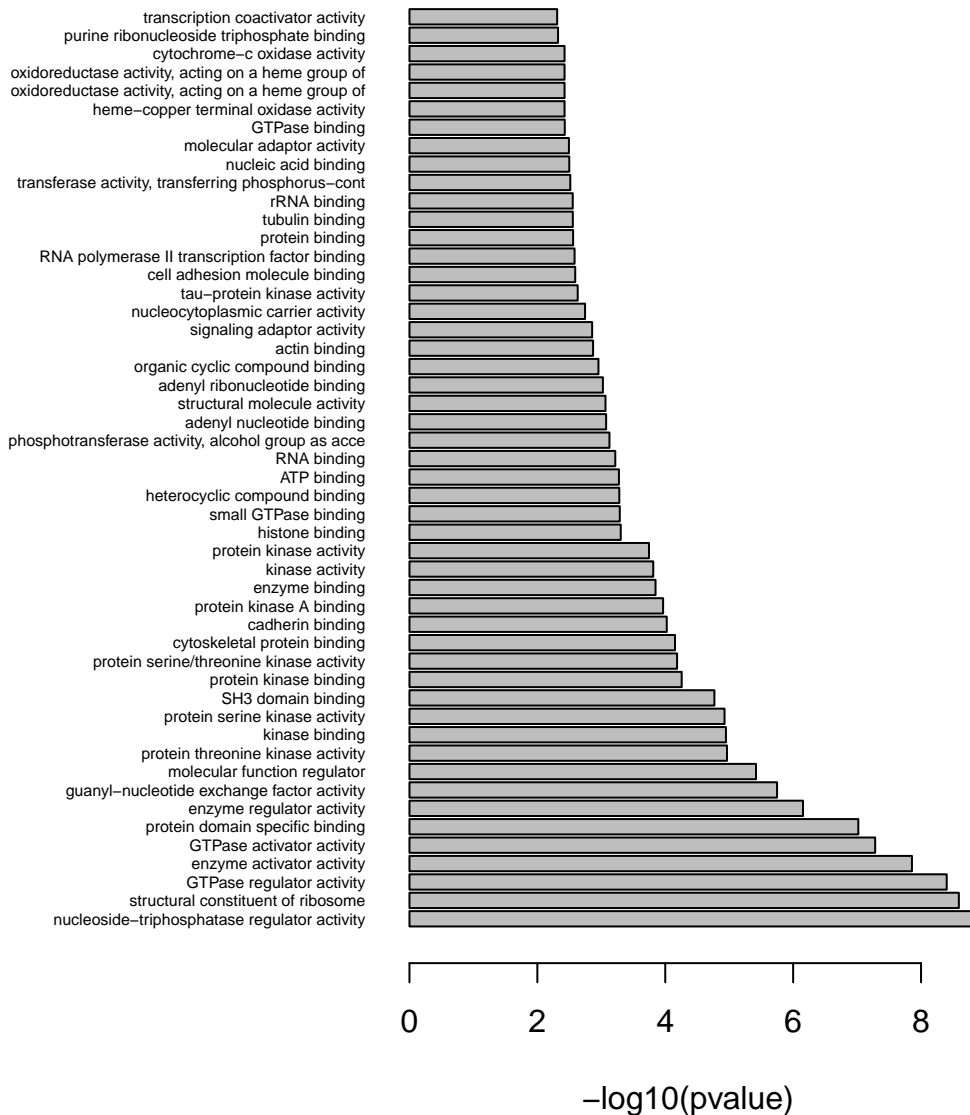

Supplement: Supplementary file 1 [file cells-11-01807-s001.zip › Supplementary_Data/DataS5/SFG_cells_GOKEGGs/SFGBS6_18_vs_SFGBS0_18/pVal_GOstats_MF_Up_pieChart.pdf]

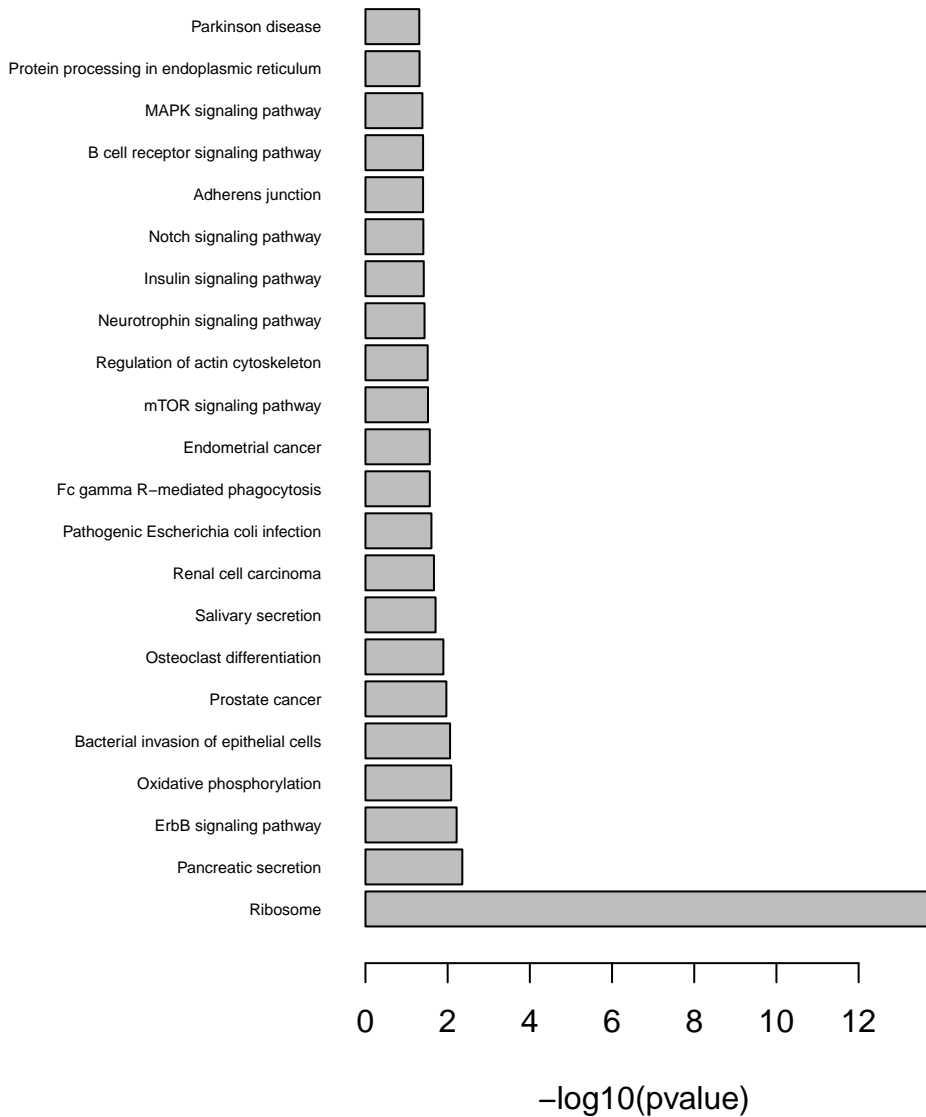

Supplement: Supplementary file 1 [file cells-11-01807-s001.zip › Supplementary_Data/DataS5/SFG_cells_GOKEGGs/SFGBS6_18_vs_SFGBS0_18/pVal_GOstats_kegg_Up.pdf]

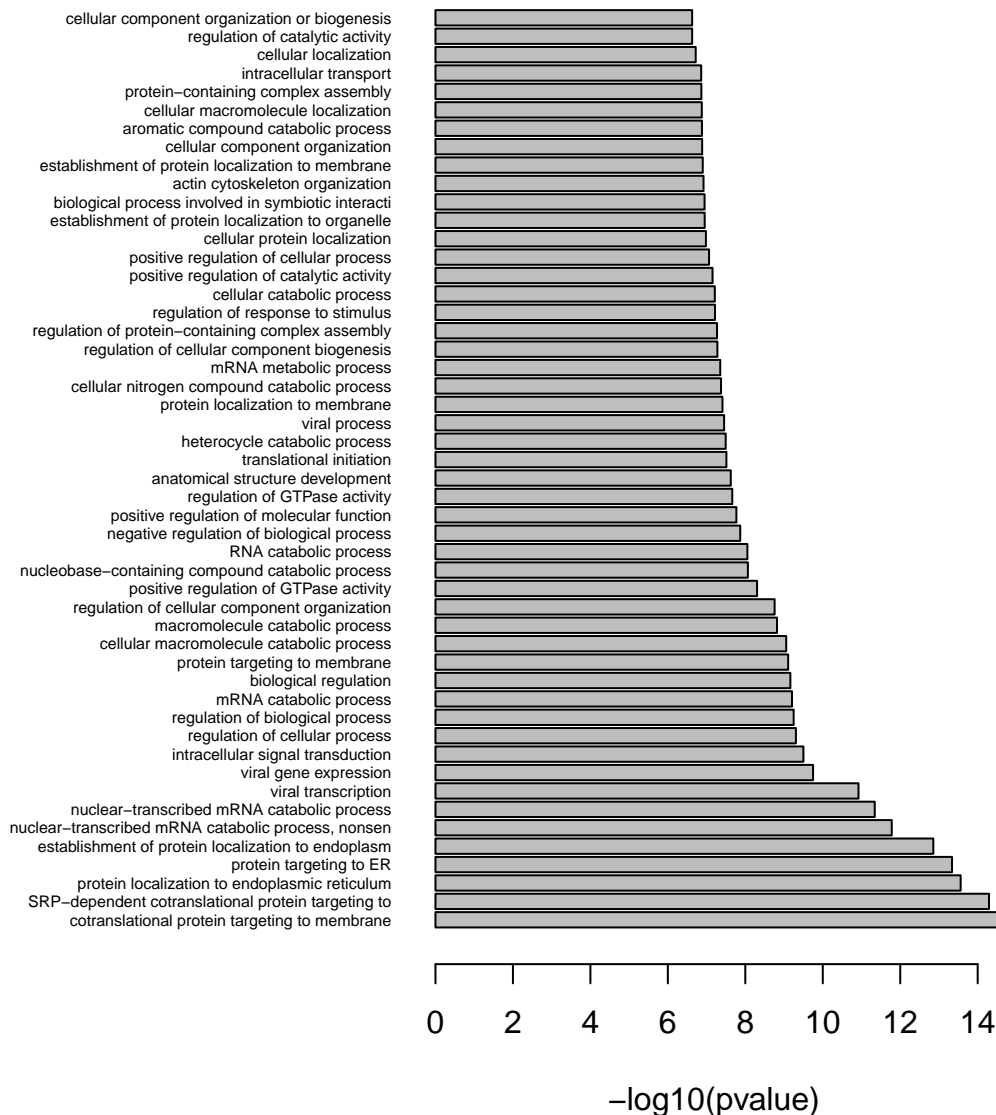

Supplement: Supplementary file 1 [file cells-11-01807-s001.zip › Supplementary_Data/DataS5/SFG_cells_GOKEGGs/SFGBS6_18_vs_SFGBS0_18/pVal_GOstats_BP_Up_pieChart.pdf]

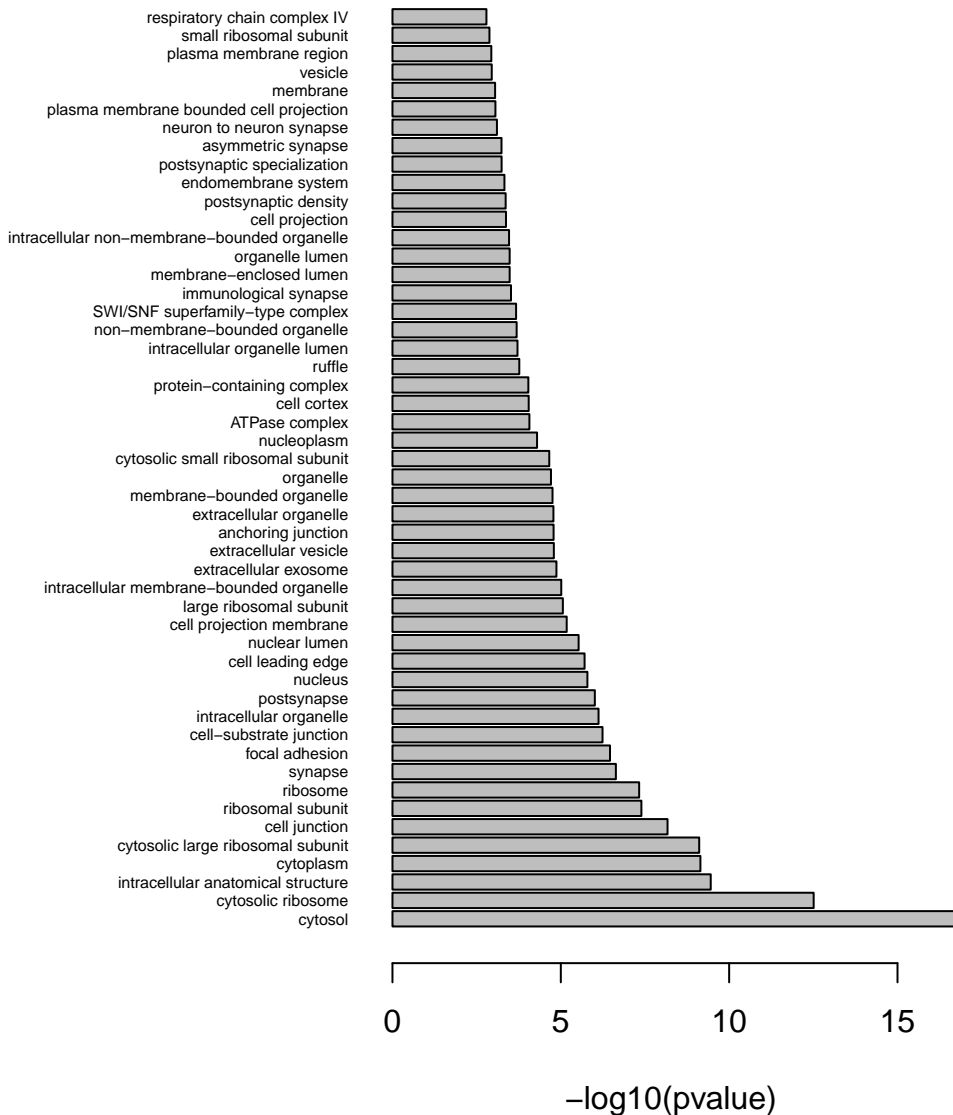

Supplement: Supplementary file 1 [file cells-11-01807-s001.zip › Supplementary_Data/DataS5/SFG_cells_GOKEGGs/SFGBS6_18_vs_SFGBS0_18/pVal_GOstats_CC_Up_pieChart.pdf]

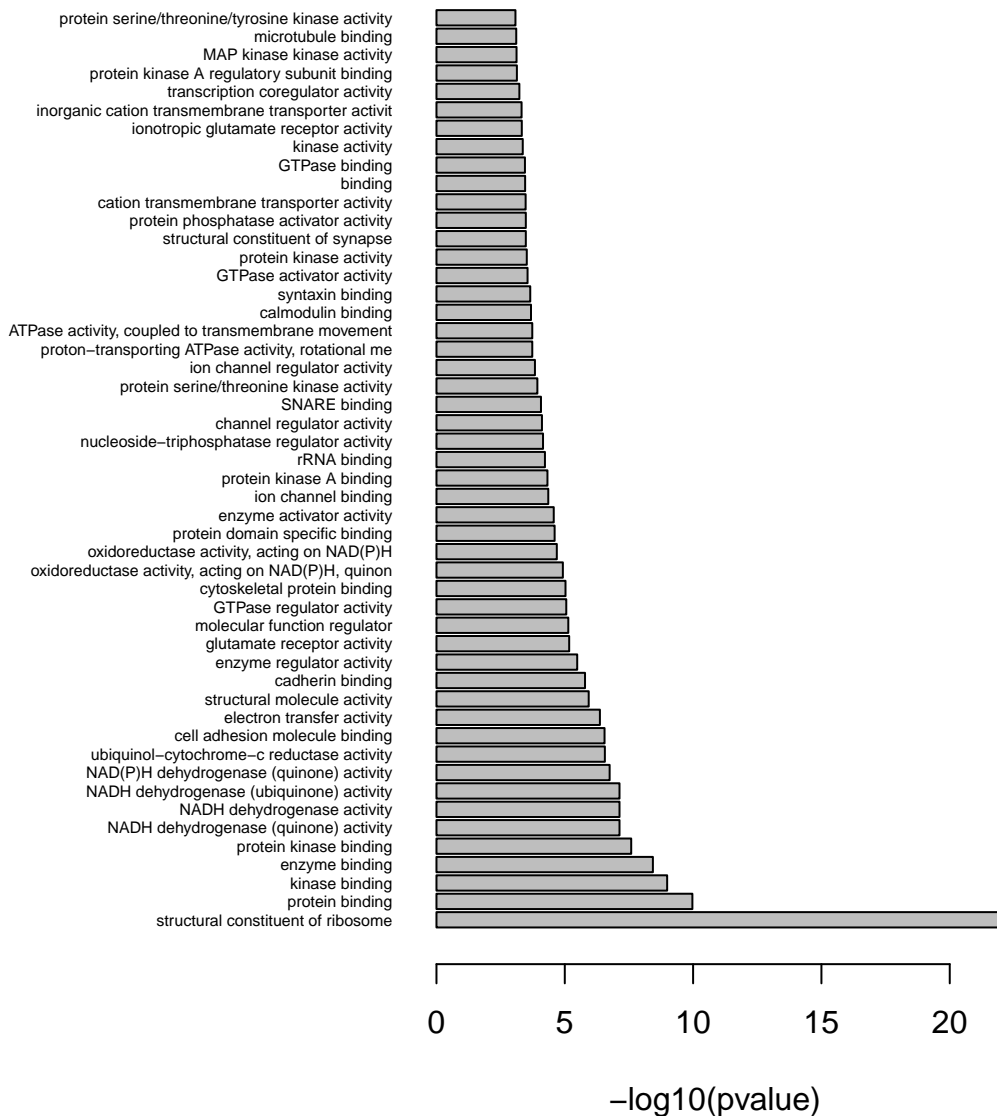

Supplement: Supplementary file 1 [file cells-11-01807-s001.zip › Supplementary_Data/DataS5/SFG_cells_GOKEGGs/SFGBS6_1_vs_SFGBS0_1/pVal_GOstats_MF_Up_pieChart.pdf]

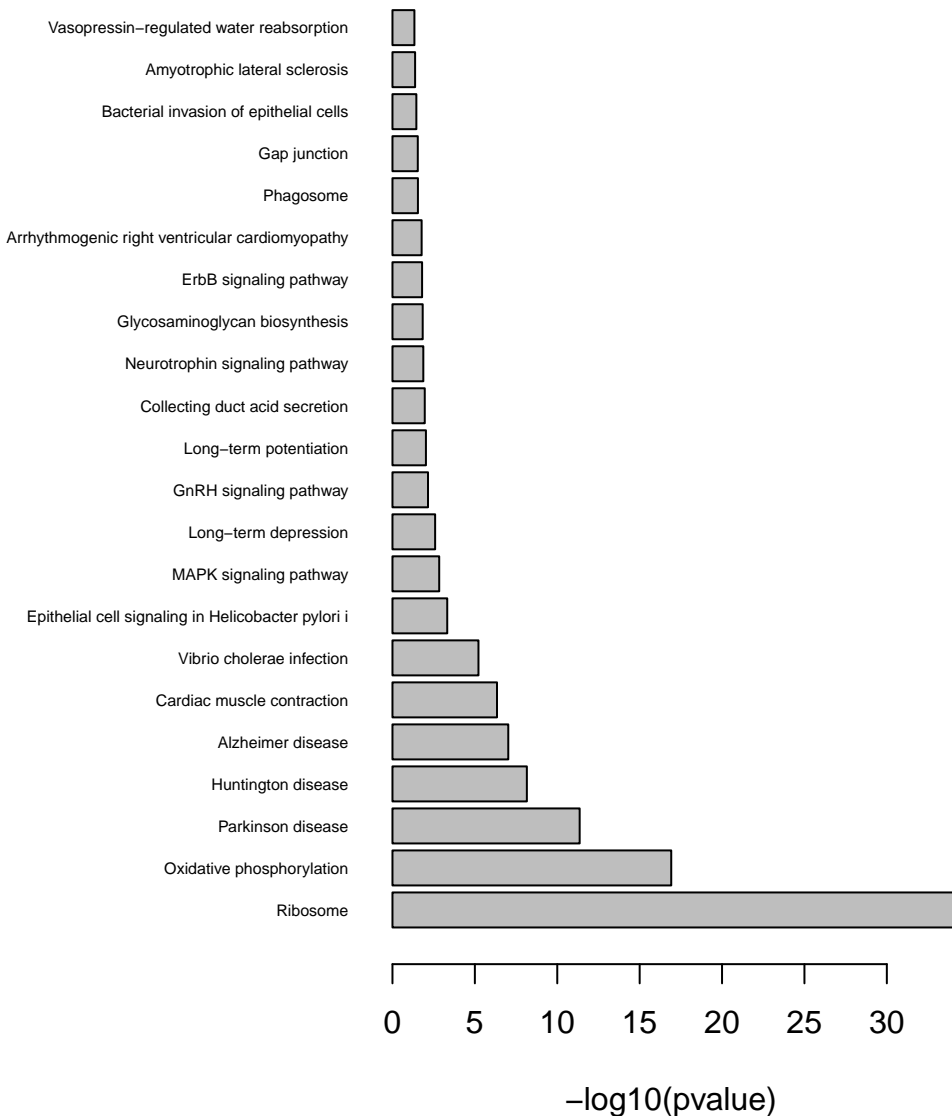

Supplement: Supplementary file 1 [file cells-11-01807-s001.zip › Supplementary_Data/DataS5/SFG_cells_GOKEGGs/SFGBS6_1_vs_SFGBS0_1/pVal_GOstats_kegg_Up.pdf]

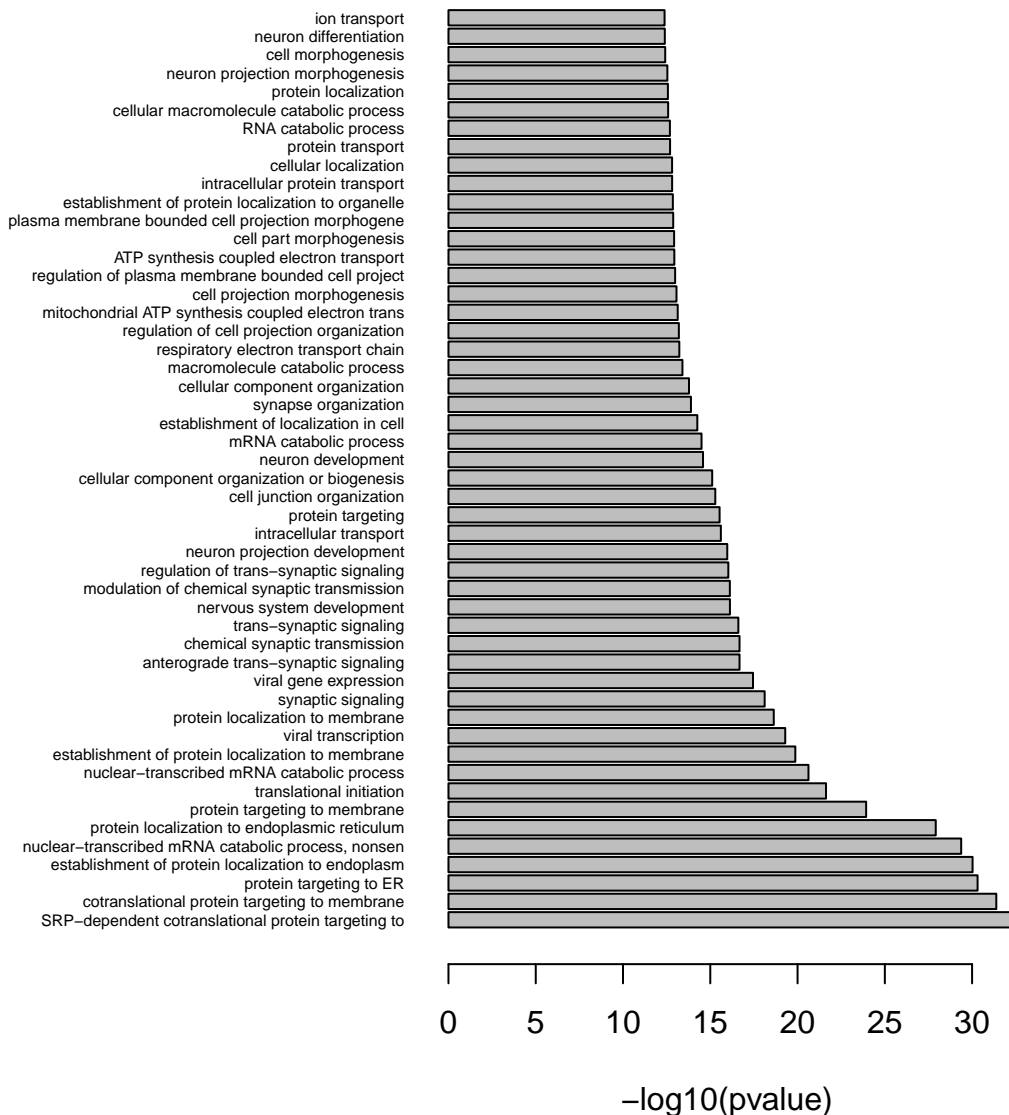

Supplement: Supplementary file 1 [file cells-11-01807-s001.zip › Supplementary_Data/DataS5/SFG_cells_GOKEGGs/SFGBS6_1_vs_SFGBS0_1/pVal_GOstats_BP_Up_pieChart.pdf]

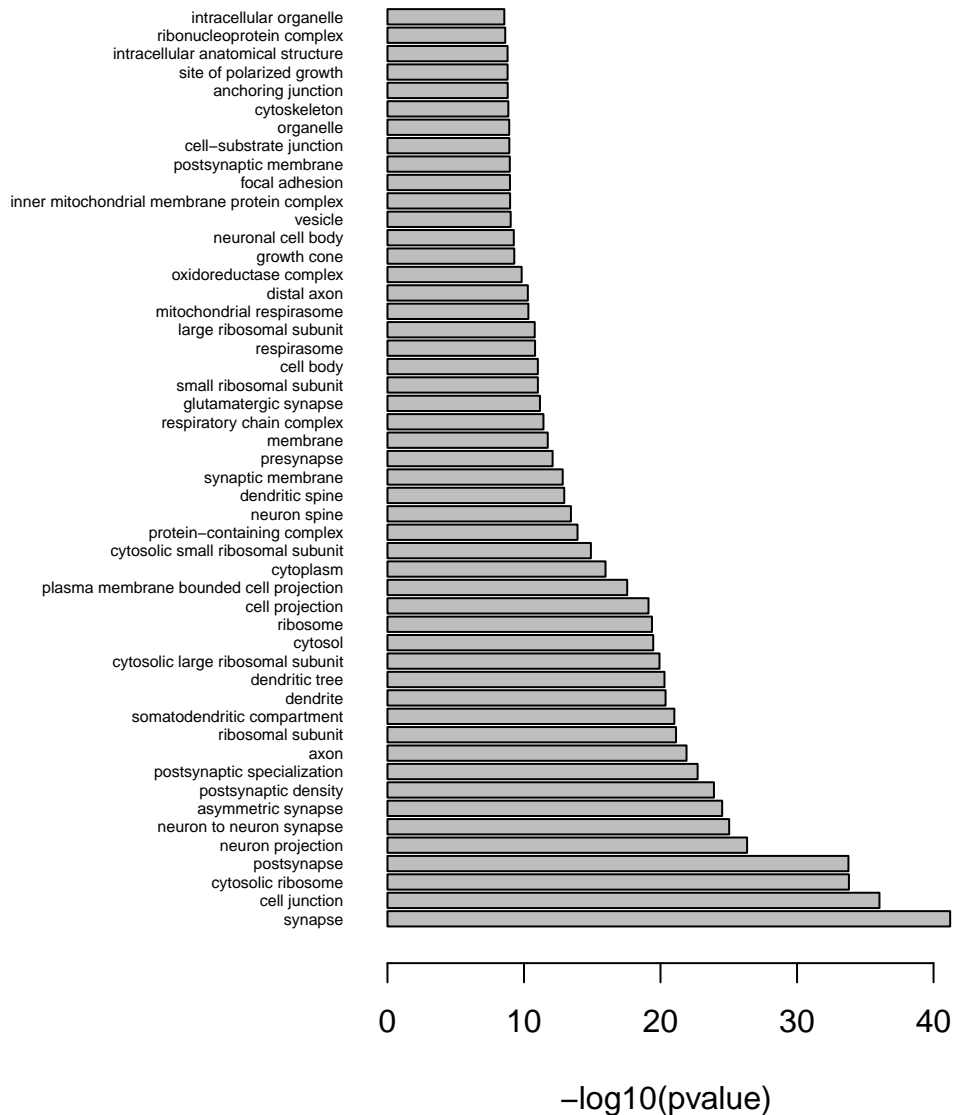

Supplement: Supplementary file 1 [file cells-11-01807-s001.zip › Supplementary_Data/DataS5/SFG_cells_GOKEGGs/SFGBS6_1_vs_SFGBS0_1/pVal_GOstats_CC_Up_pieChart.pdf]

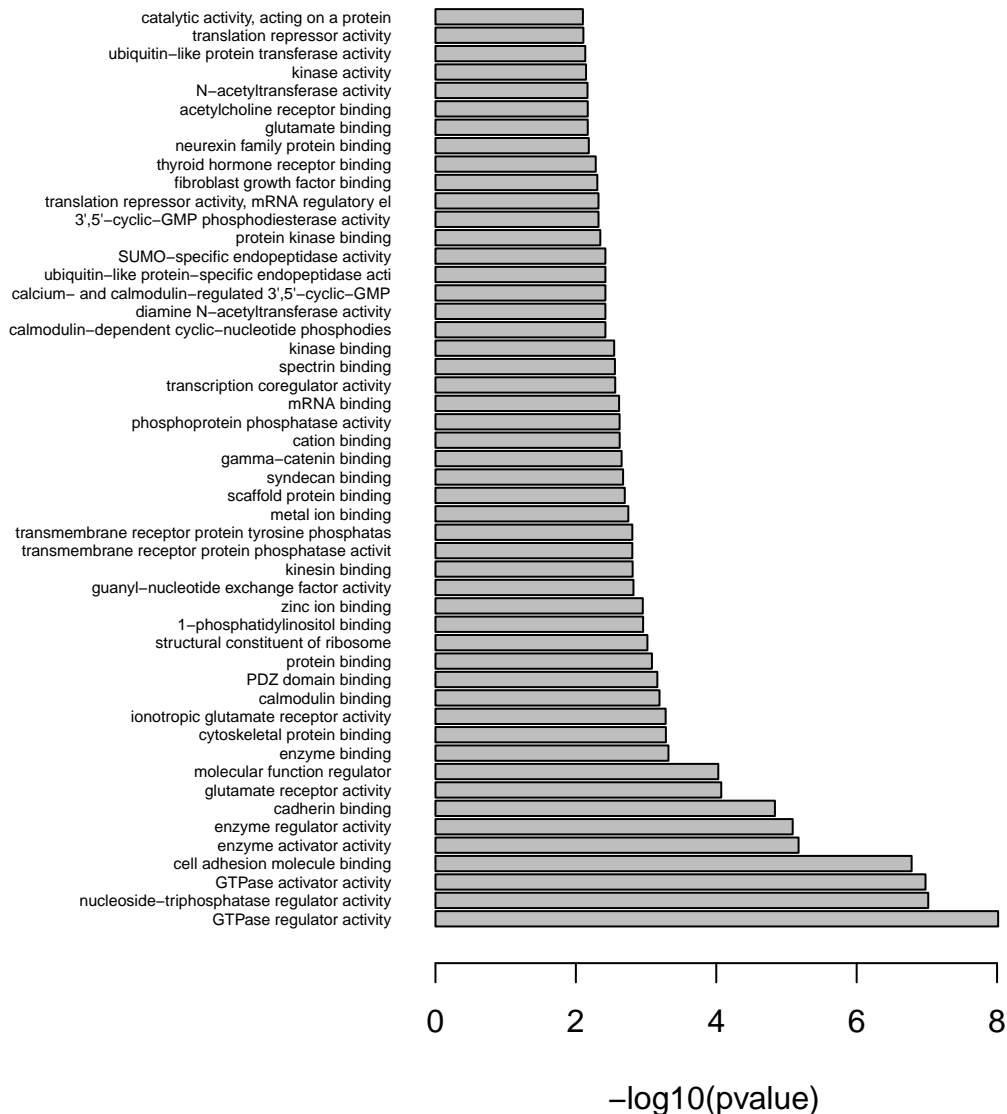

Supplement: Supplementary file 1 [file cells-11-01807-s001.zip › Supplementary_Data/DataS5/EC_cells_GOKEGGs/ECBS6_6_vs_ECBS0_6/pVal_GOstats_MF_Up_pieChart.pdf]

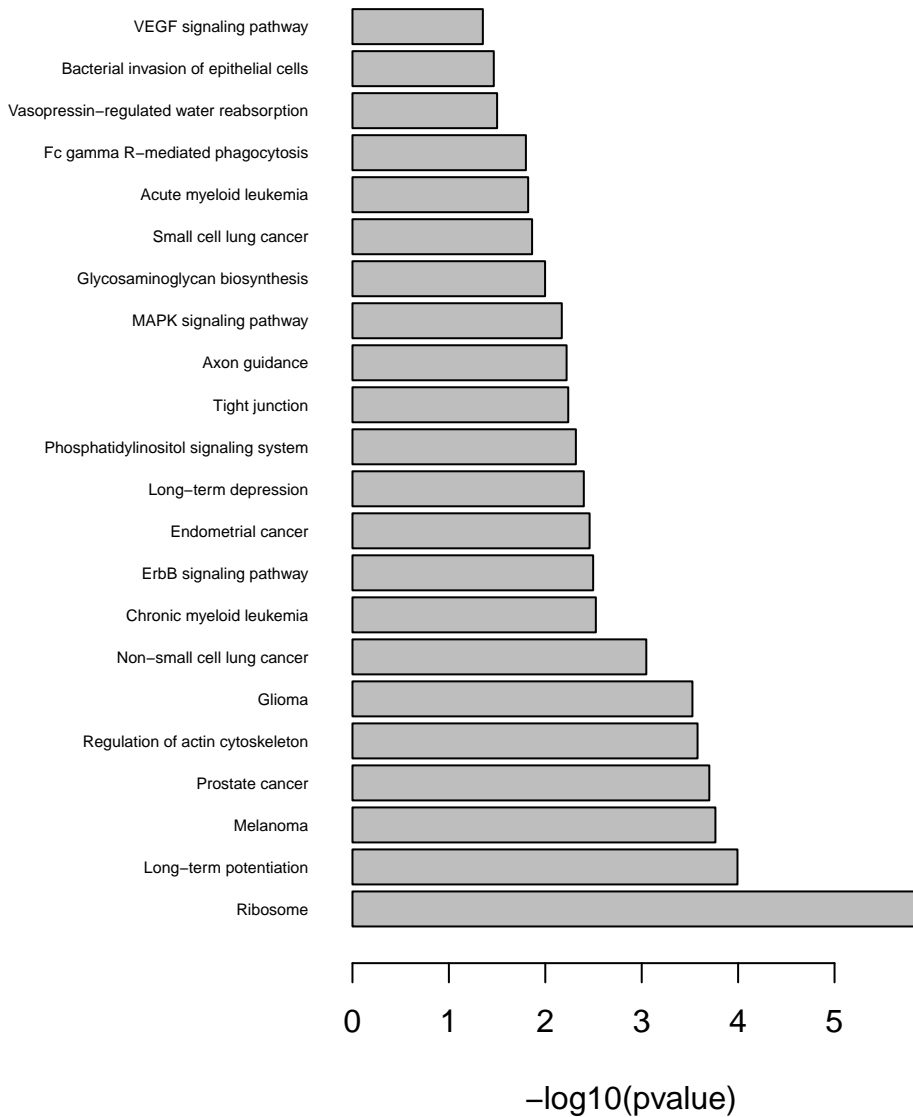

Supplement: Supplementary file 1 [file cells-11-01807-s001.zip › Supplementary_Data/DataS5/EC_cells_GOKEGGs/ECBS6_6_vs_ECBS0_6/pVal_GOstats_kegg_Up.pdf]

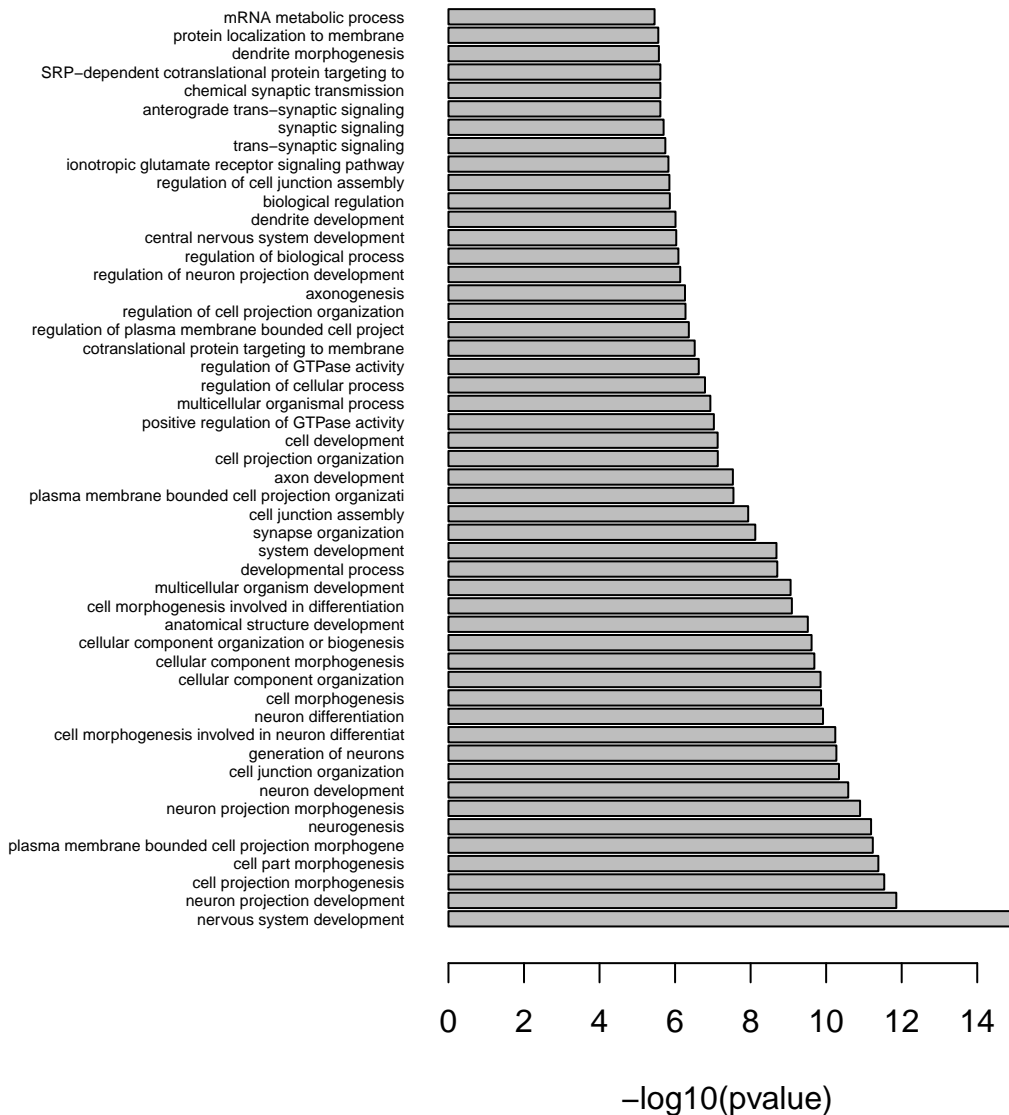

Supplement: Supplementary file 1 [file cells-11-01807-s001.zip › Supplementary_Data/DataS5/EC_cells_GOKEGGs/ECBS6_6_vs_ECBS0_6/pVal_GOstats_BP_Up_pieChart.pdf]

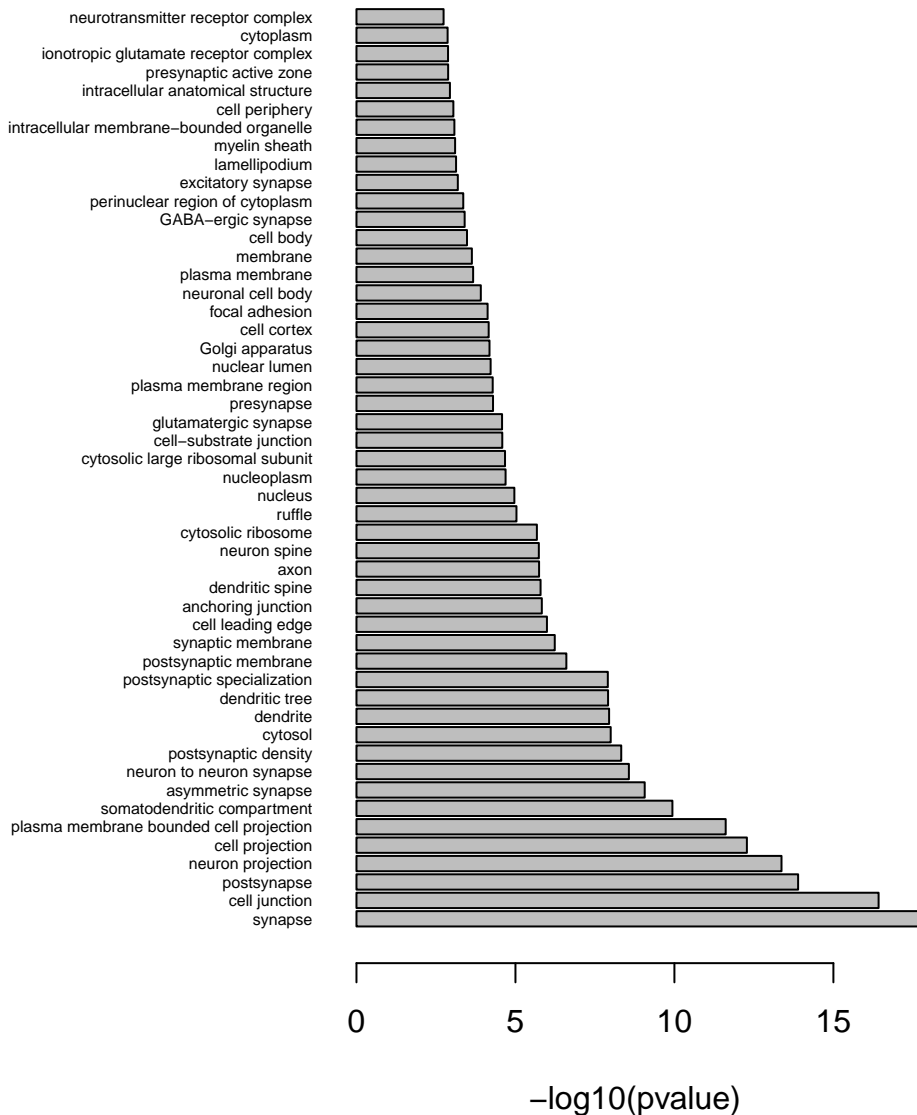

Supplement: Supplementary file 1 [file cells-11-01807-s001.zip › Supplementary_Data/DataS5/EC_cells_GOKEGGs/ECBS6_6_vs_ECBS0_6/pVal_GOstats_CC_Up_pieChart.pdf]

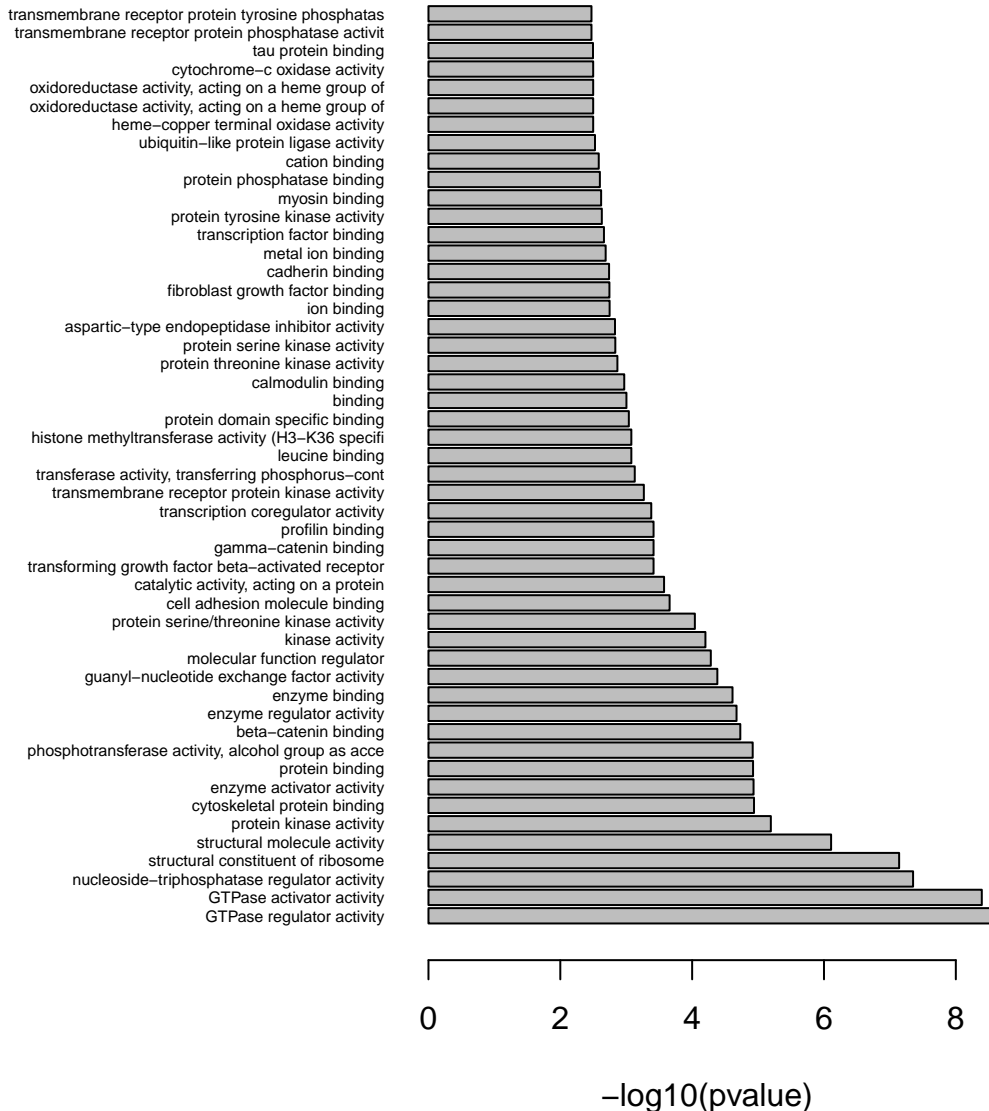

Supplement: Supplementary file 1 [file cells-11-01807-s001.zip › Supplementary_Data/DataS5/EC_cells_GOKEGGs/ECBS6_2_vs_ECBS0_2/pVal_GOstats_MF_Up_pieChart.pdf]

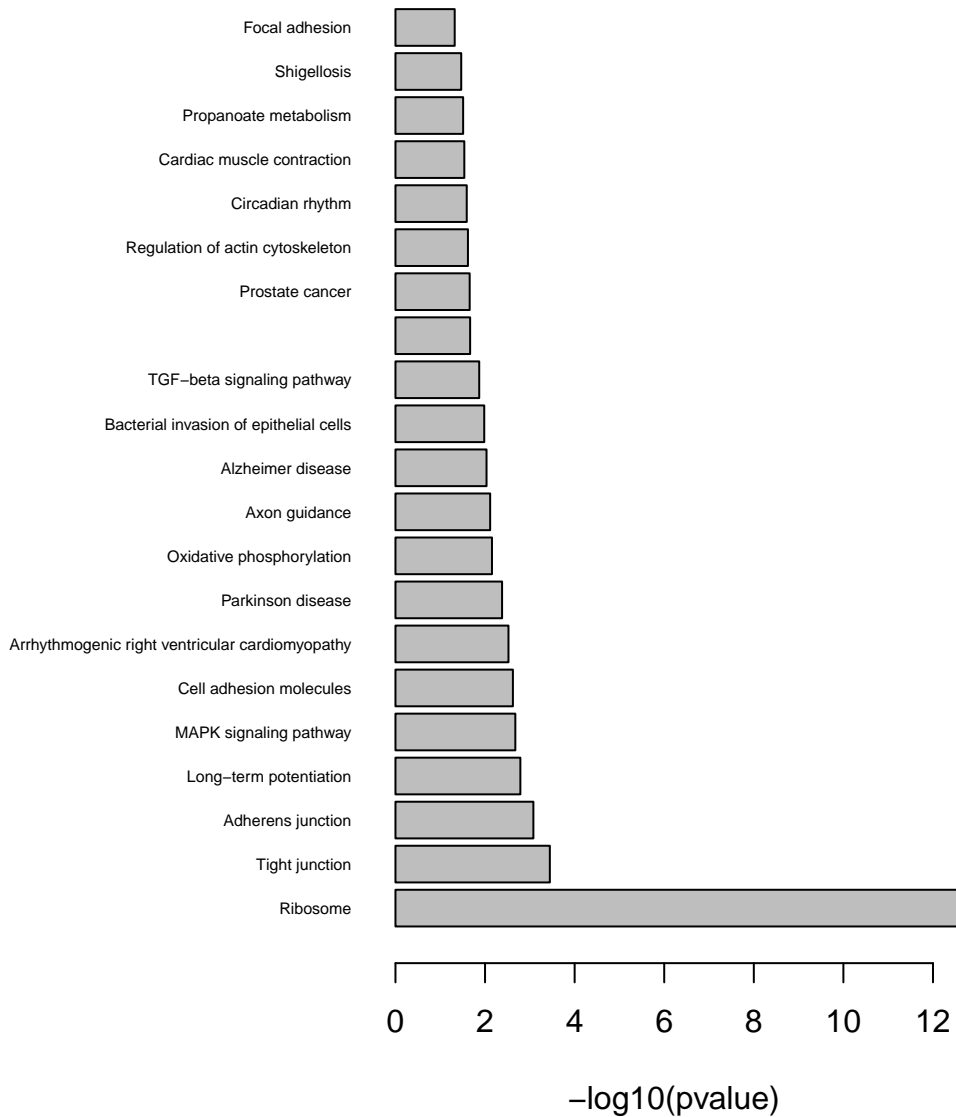

Supplement: Supplementary file 1 [file cells-11-01807-s001.zip › Supplementary_Data/DataS5/EC_cells_GOKEGGs/ECBS6_2_vs_ECBS0_2/pVal_GOstats_kegg_Up.pdf]

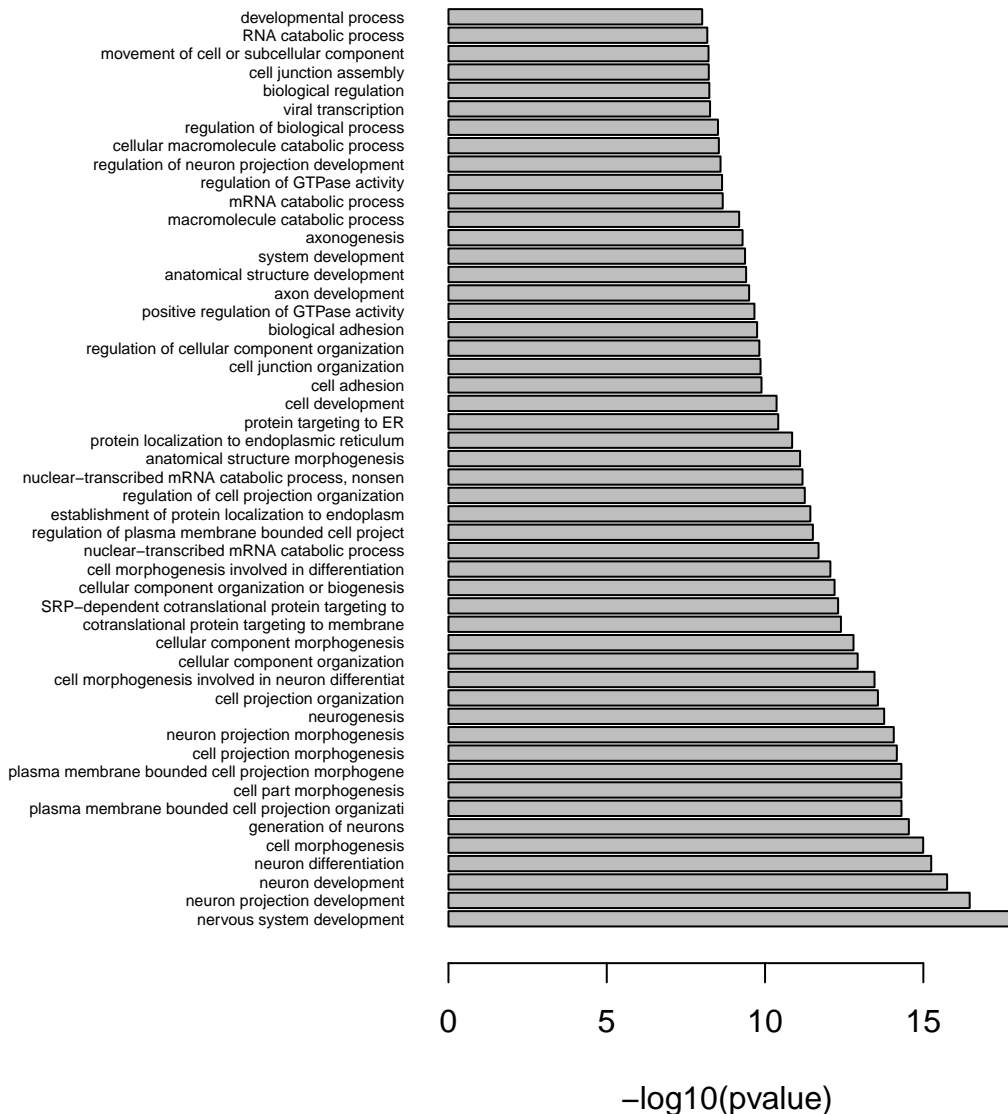

Supplement: Supplementary file 1 [file cells-11-01807-s001.zip › Supplementary_Data/DataS5/EC_cells_GOKEGGs/ECBS6_2_vs_ECBS0_2/pVal_GOstats_BP_Up_pieChart.pdf]

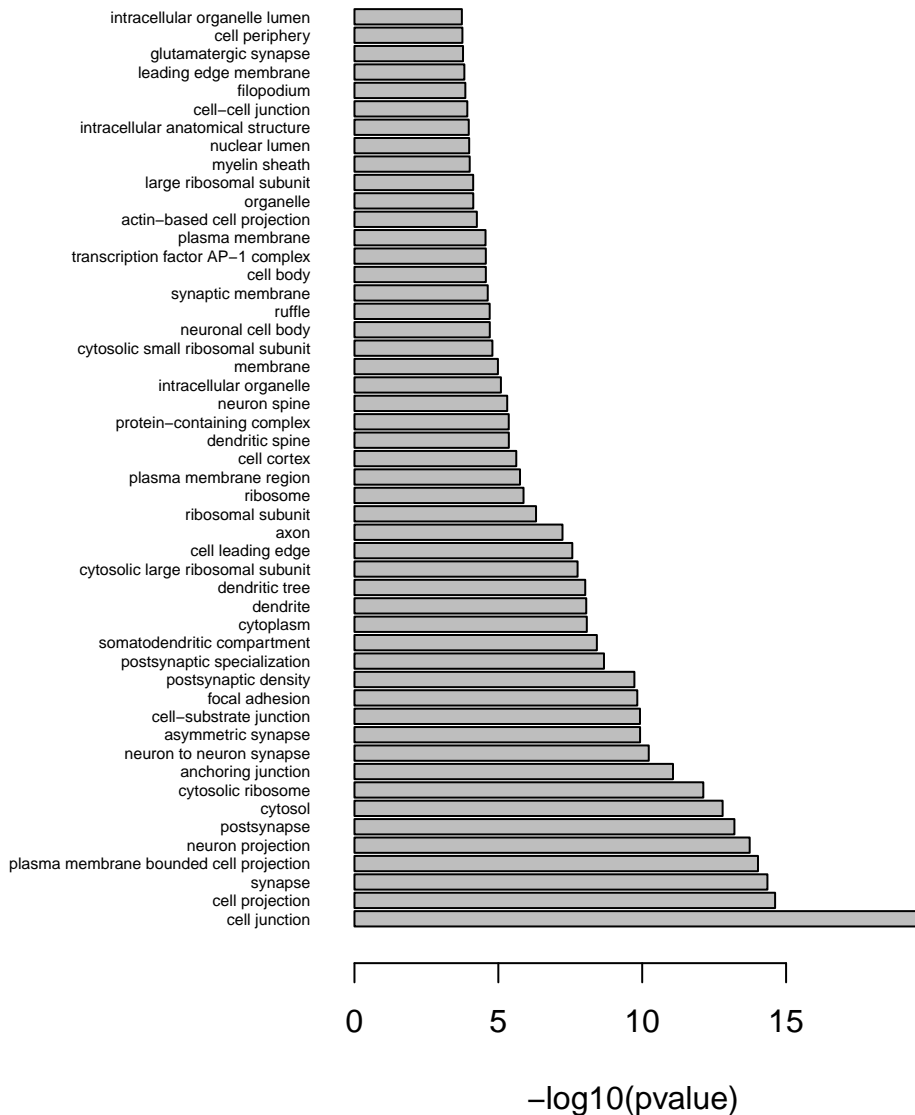

Supplement: Supplementary file 1 [file cells-11-01807-s001.zip › Supplementary_Data/DataS5/EC_cells_GOKEGGs/ECBS6_2_vs_ECBS0_2/pVal_GOstats_CC_Up_pieChart.pdf]

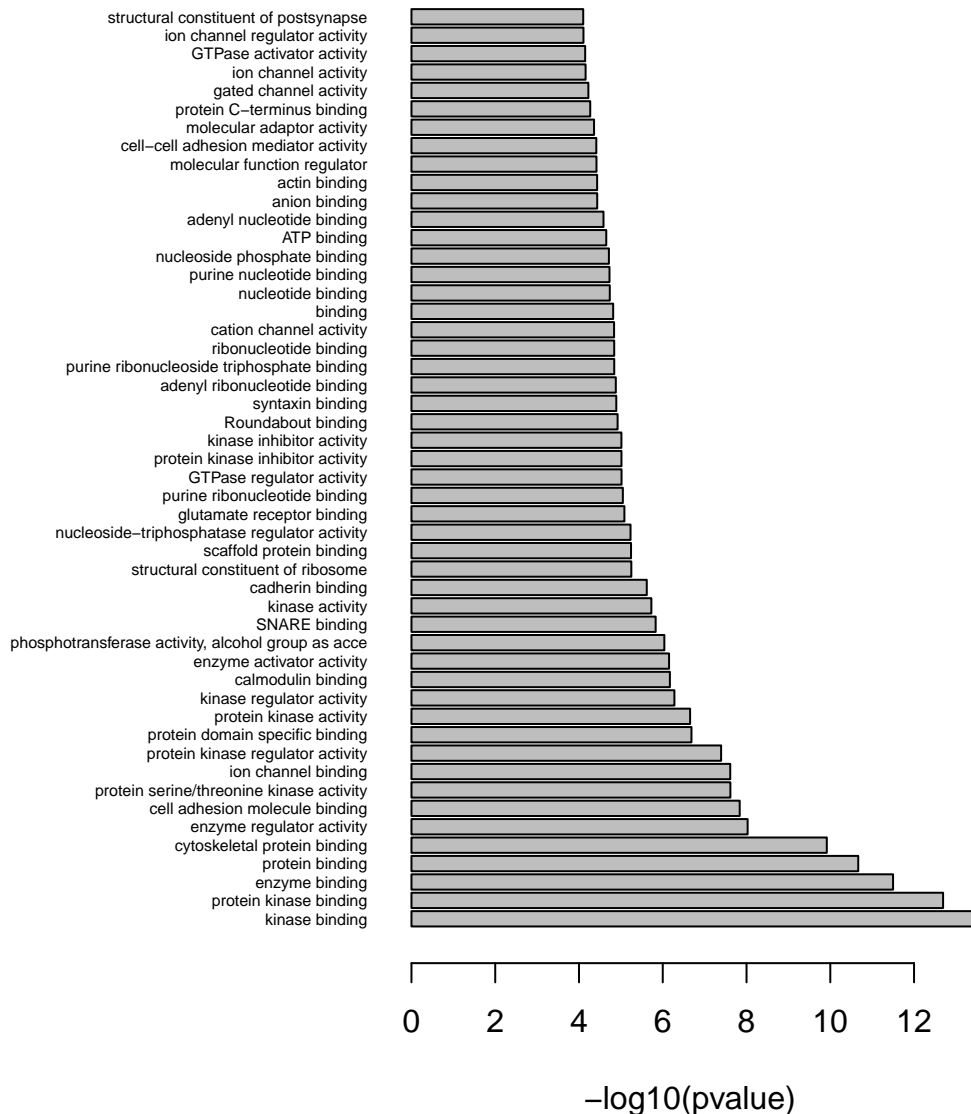

Supplement: Supplementary file 1 [file cells-11-01807-s001.zip › Supplementary_Data/DataS5/EC_cells_GOKEGGs/ECBS6_14_vs_ECBS0_14/pVal_GOstats_MF_Up_pieChart.pdf]

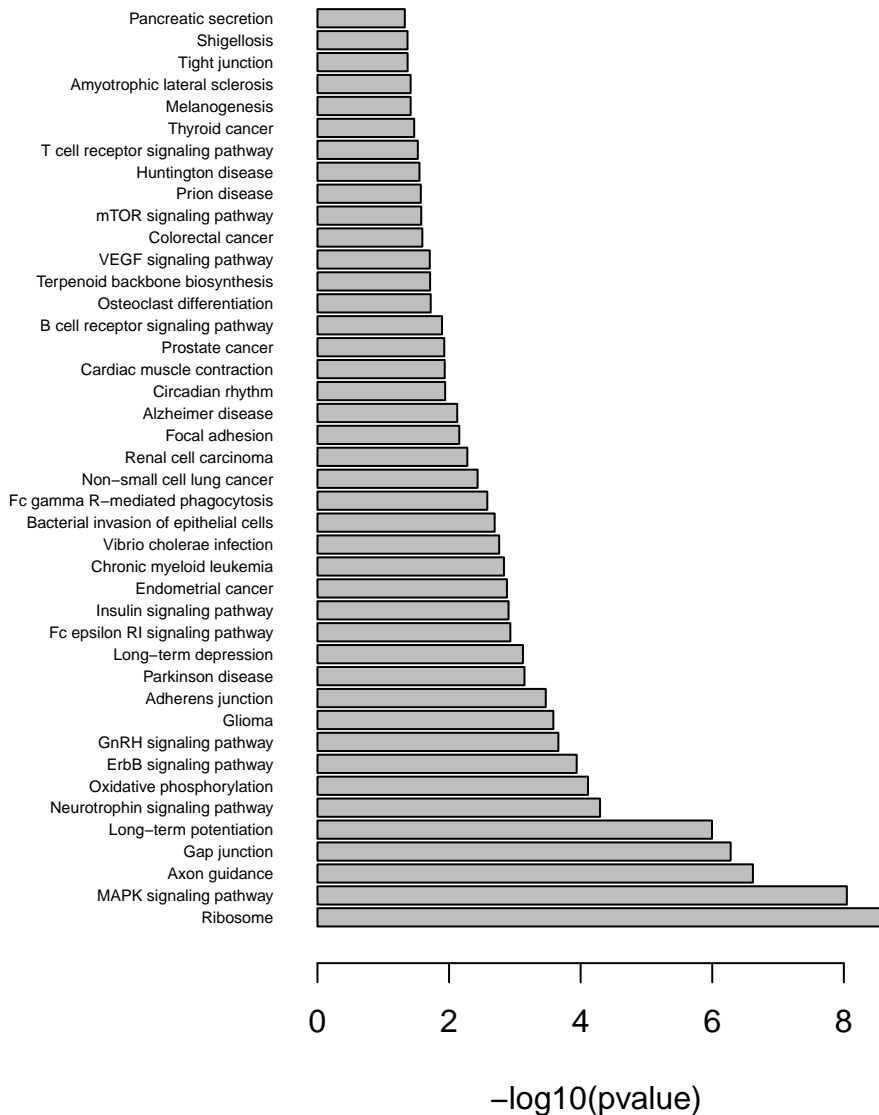

Supplement: Supplementary file 1 [file cells-11-01807-s001.zip › Supplementary_Data/DataS5/EC_cells_GOKEGGs/ECBS6_14_vs_ECBS0_14/pVal_GOstats_kegg_Up.pdf]

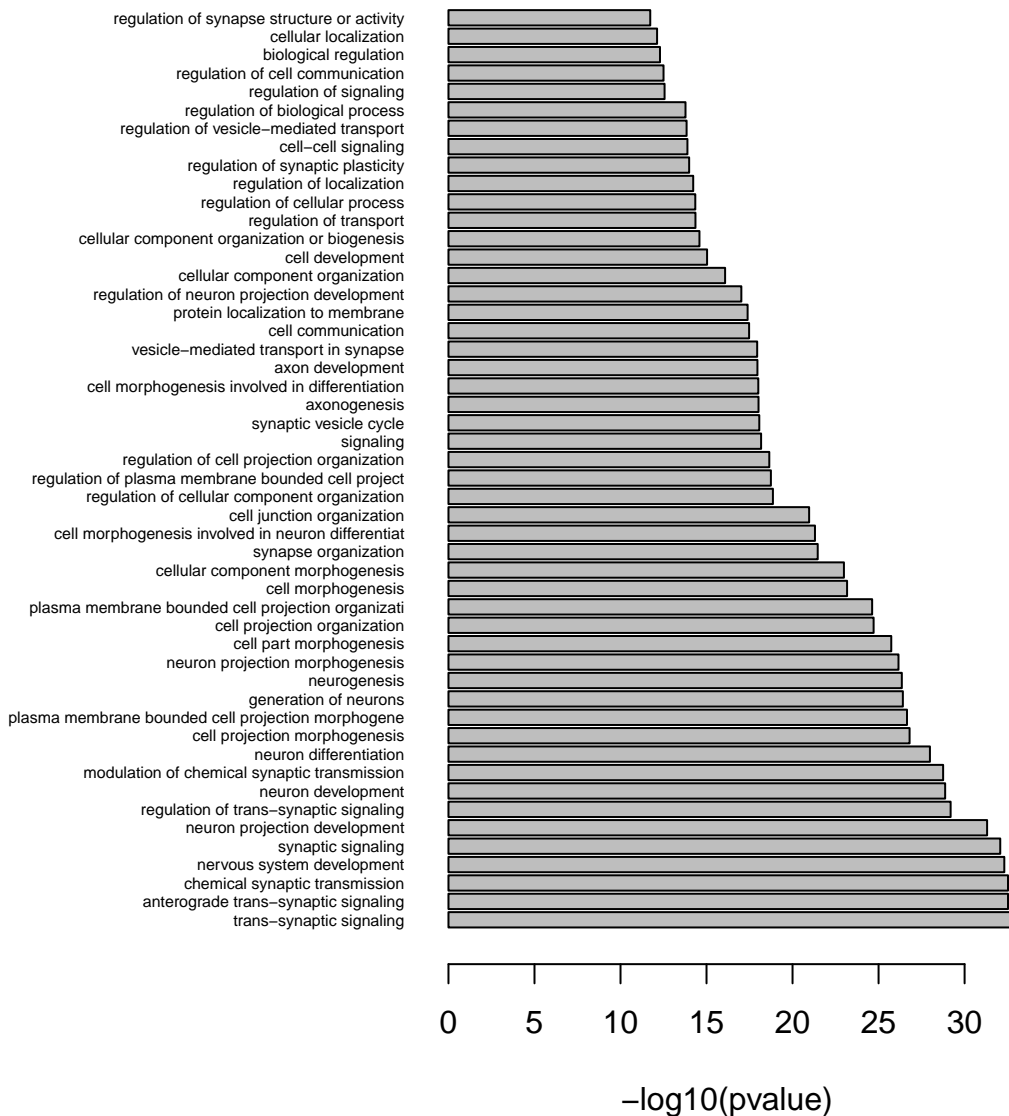

Supplement: Supplementary file 1 [file cells-11-01807-s001.zip › Supplementary_Data/DataS5/EC_cells_GOKEGGs/ECBS6_14_vs_ECBS0_14/pVal_GOstats_BP_Up_pieChart.pdf]

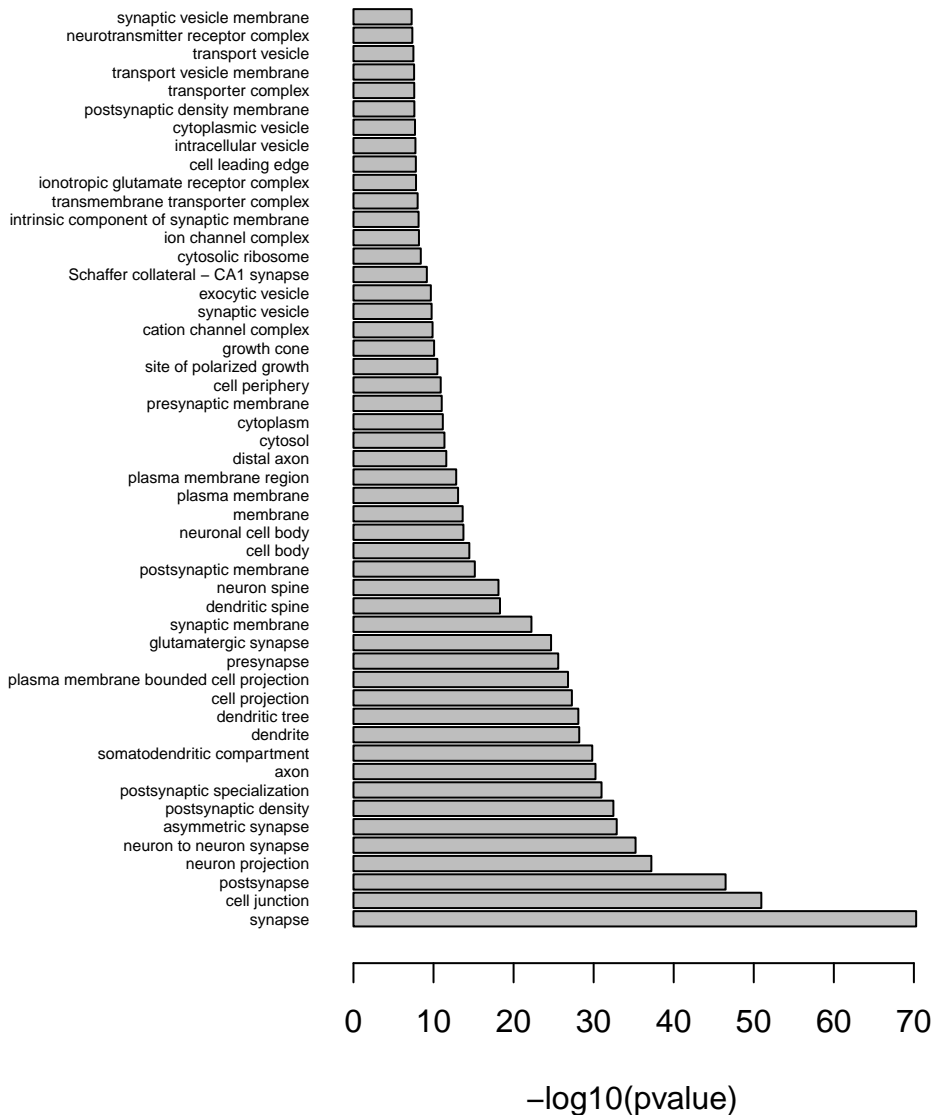

Supplement: Supplementary file 1 [file cells-11-01807-s001.zip › Supplementary_Data/DataS5/EC_cells_GOKEGGs/ECBS6_14_vs_ECBS0_14/pVal_GOstats_CC_Up_pieChart.pdf]

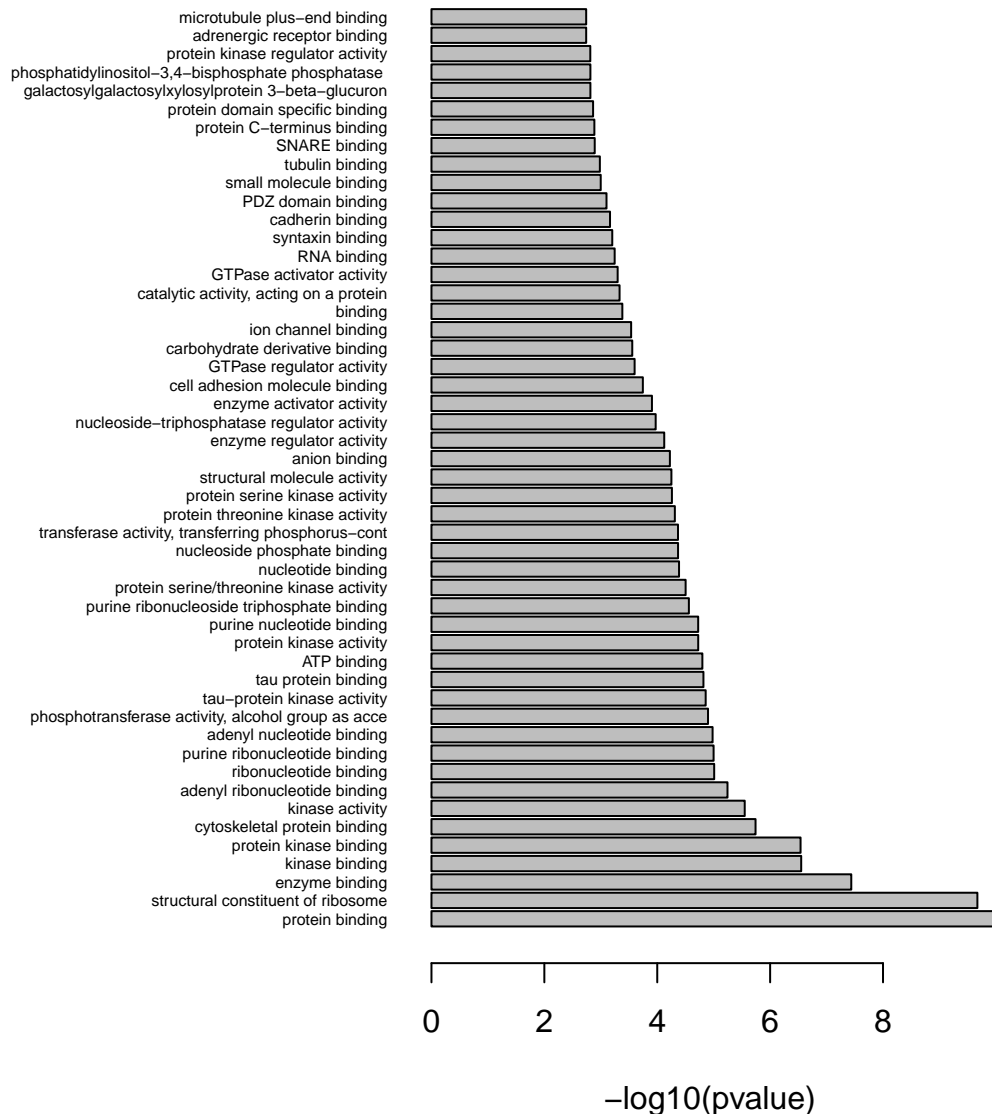

Supplement: Supplementary file 1 [file cells-11-01807-s001.zip › Supplementary_Data/DataS5/EC_cells_GOKEGGs/ECBS6_12_vs_ECBS0_12/pVal_GOstats_MF_Up_pieChart.pdf]

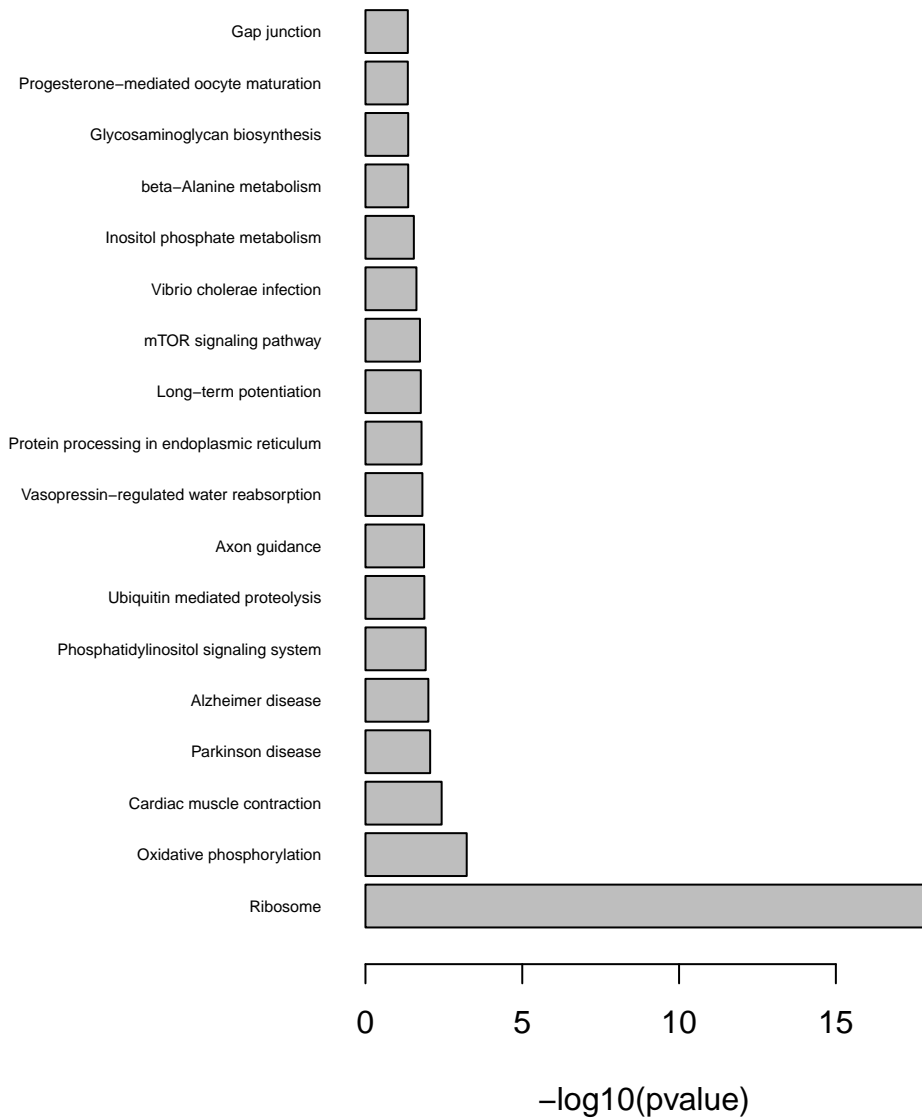

Supplement: Supplementary file 1 [file cells-11-01807-s001.zip › Supplementary_Data/DataS5/EC_cells_GOKEGGs/ECBS6_12_vs_ECBS0_12/pVal_GOstats_kegg_Up.pdf]

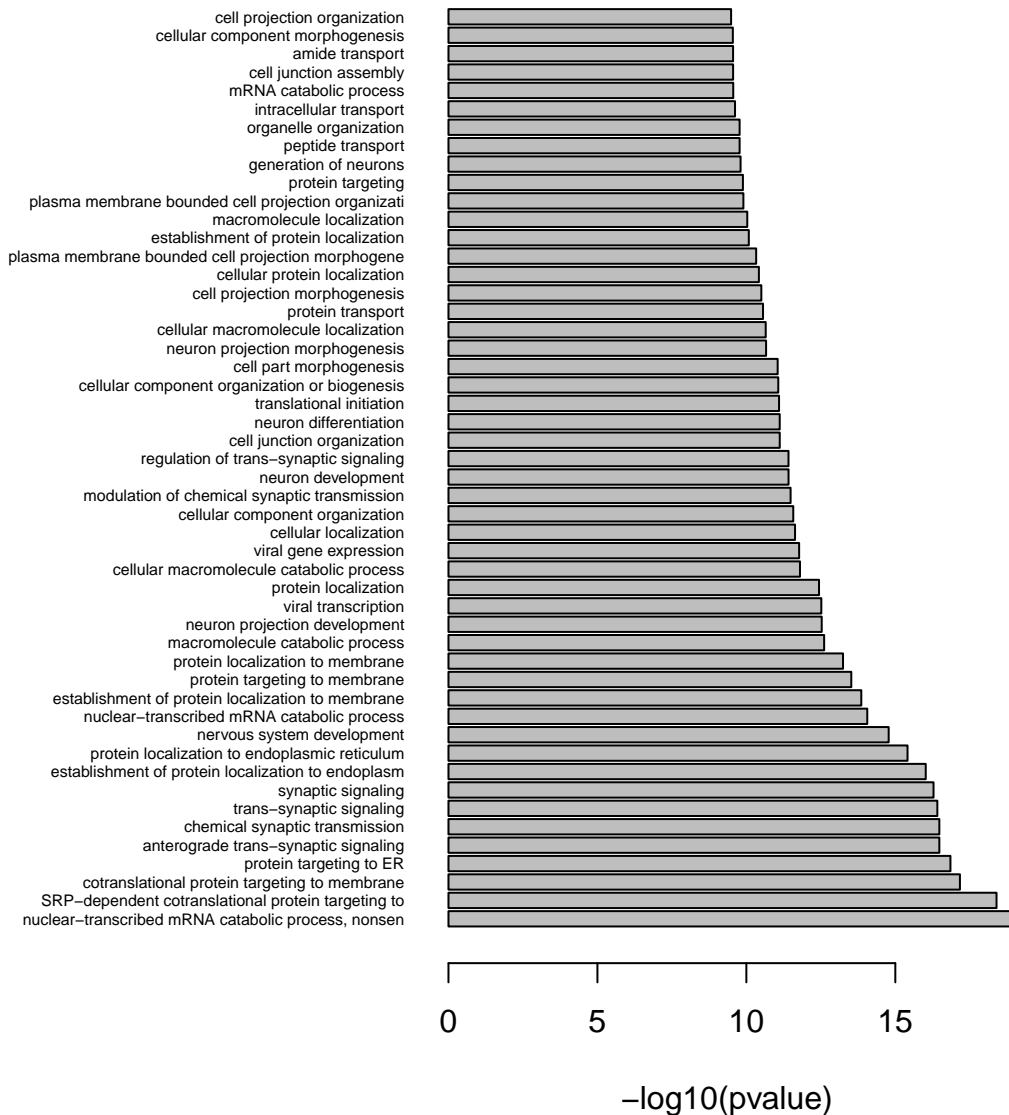

Supplement: Supplementary file 1 [file cells-11-01807-s001.zip › Supplementary_Data/DataS5/EC_cells_GOKEGGs/ECBS6_12_vs_ECBS0_12/pVal_GOstats_BP_Up_pieChart.pdf]

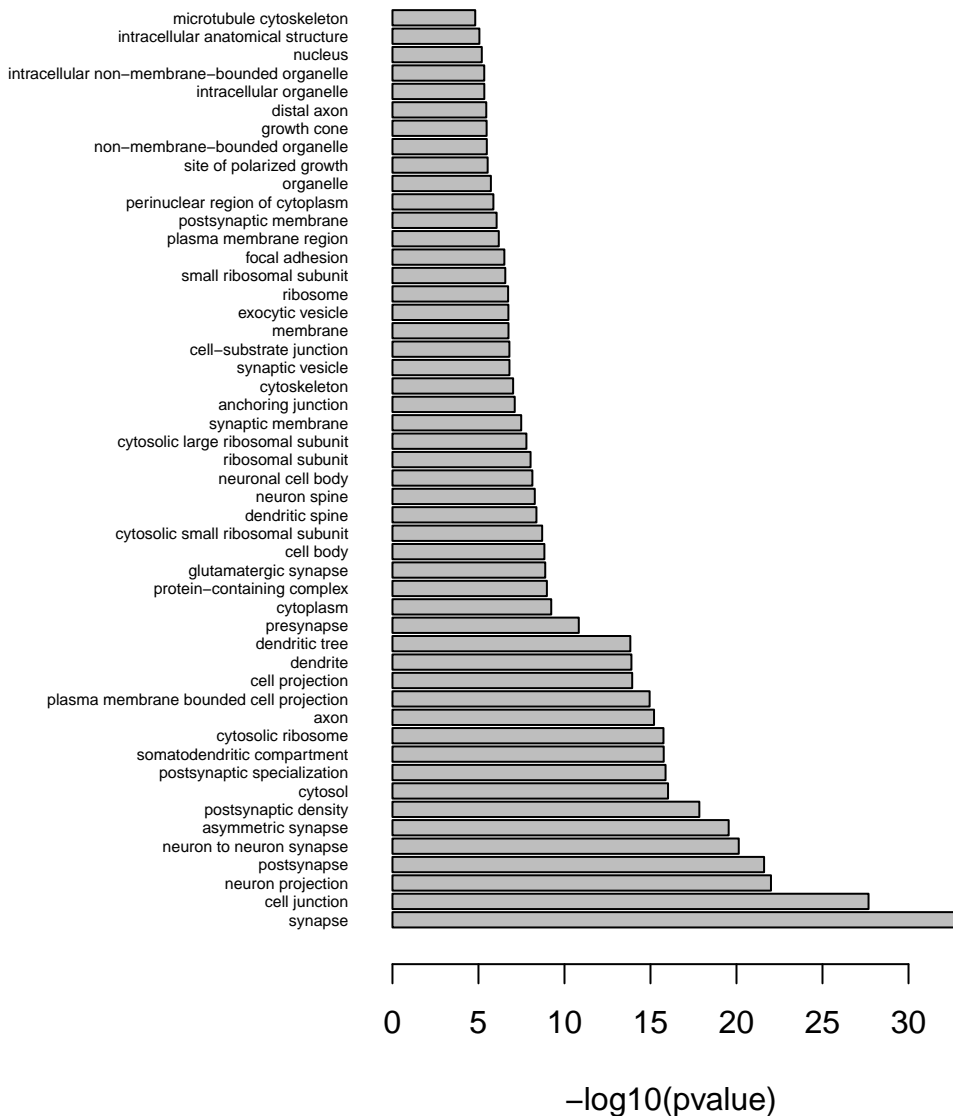

Supplement: Supplementary file 1 [file cells-11-01807-s001.zip › Supplementary_Data/DataS5/EC_cells_GOKEGGs/ECBS6_12_vs_ECBS0_12/pVal_GOstats_CC_Up_pieChart.pdf]

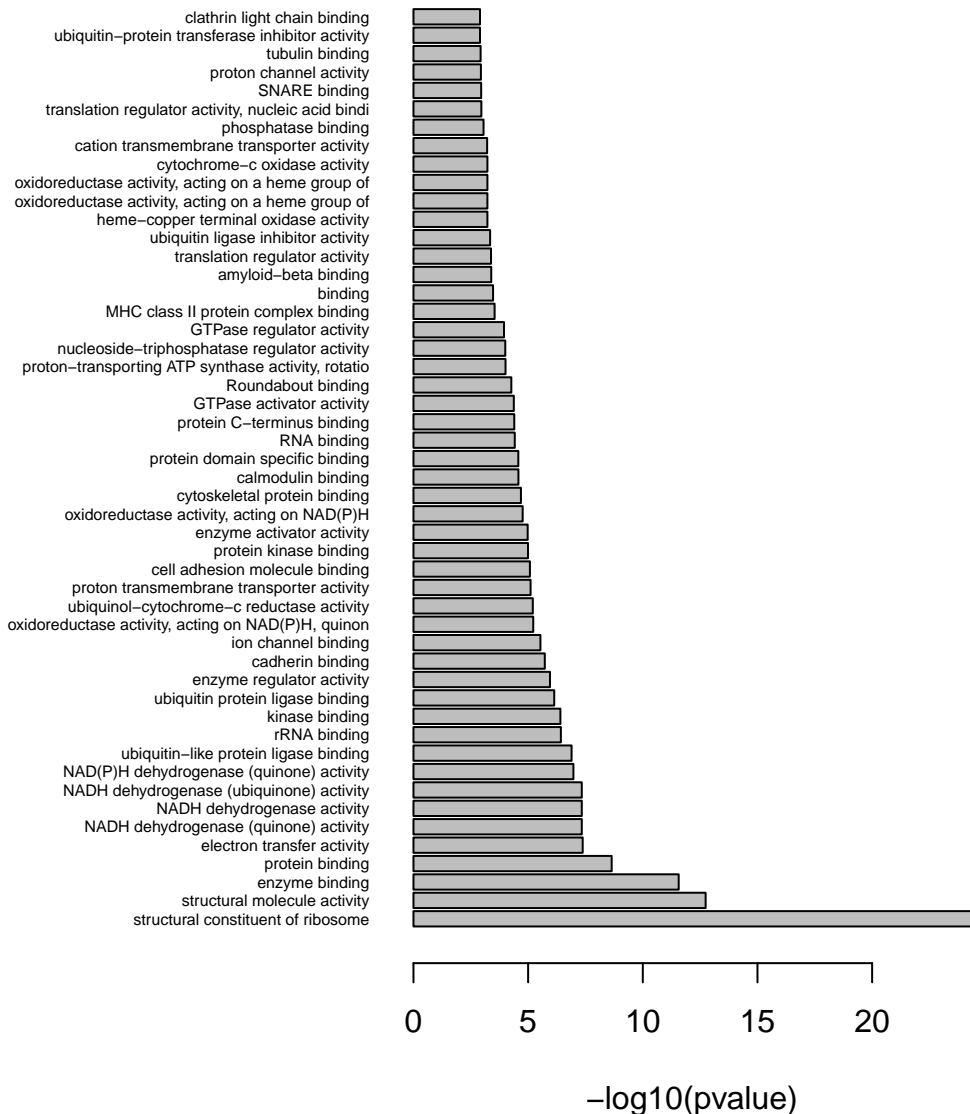

Supplement: Supplementary file 1 [file cells-11-01807-s001.zip › Supplementary_Data/DataS5/EC_cells_GOKEGGs/ECBS6_1_vs_ECBS0_1/pVal_GOstats_MF_Up_pieChart.pdf]

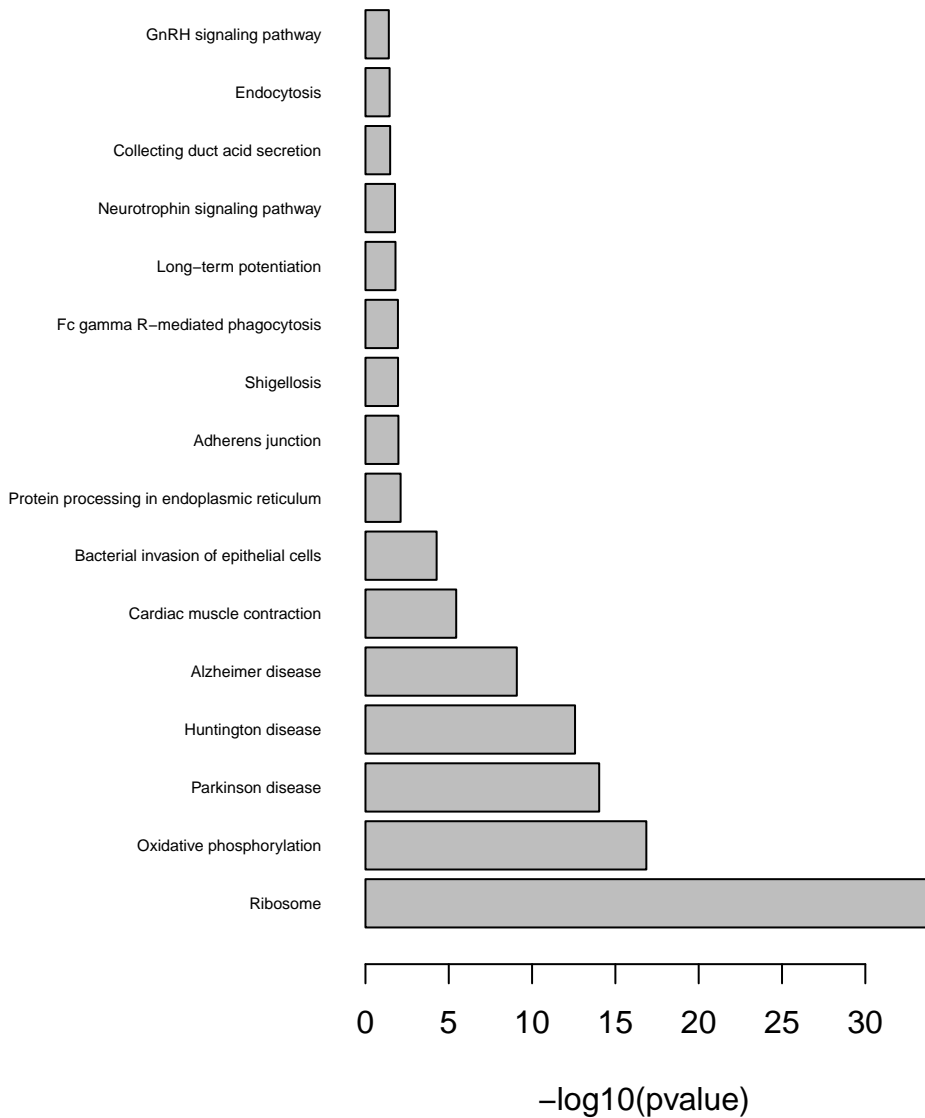

Supplement: Supplementary file 1 [file cells-11-01807-s001.zip › Supplementary_Data/DataS5/EC_cells_GOKEGGs/ECBS6_1_vs_ECBS0_1/pVal_GOstats_kegg_Up.pdf]

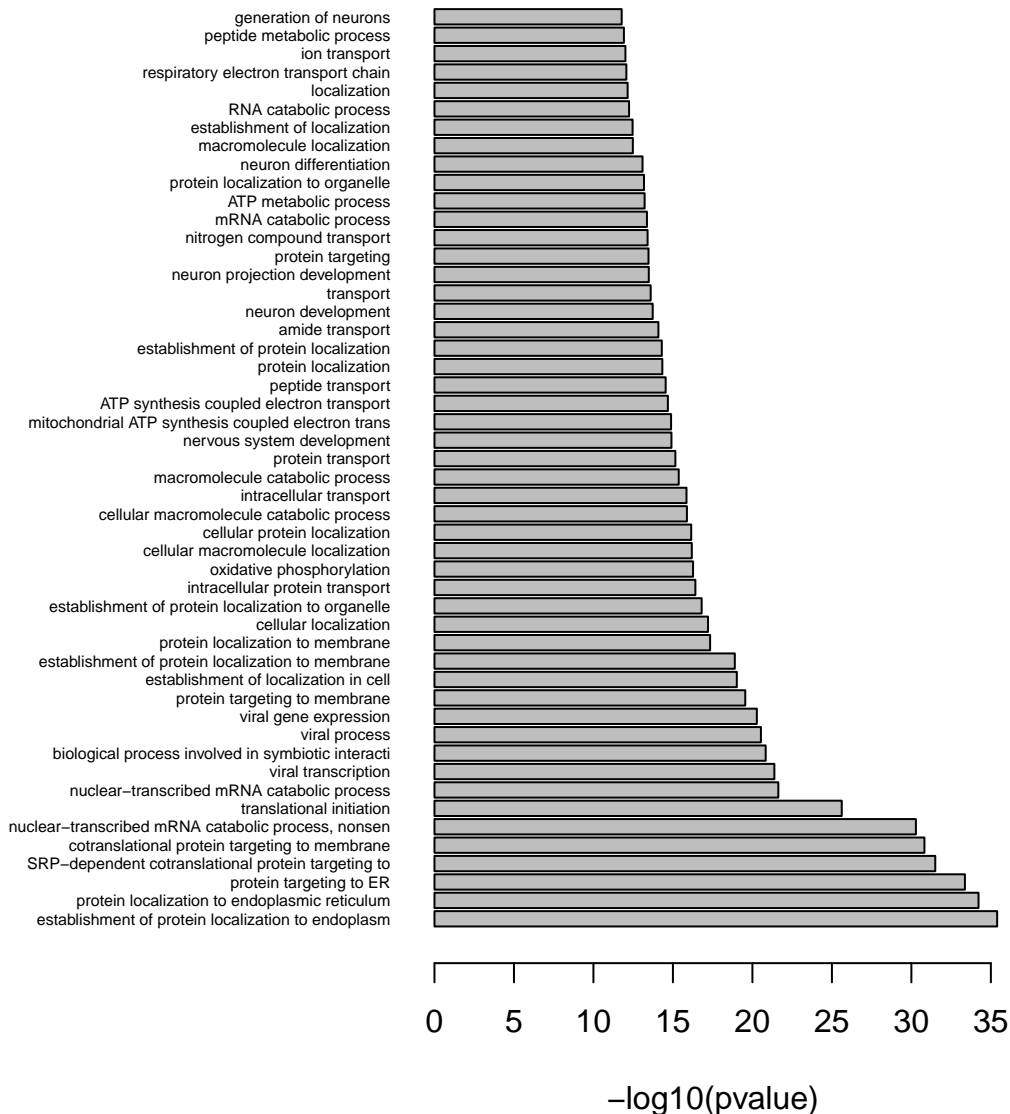

Supplement: Supplementary file 1 [file cells-11-01807-s001.zip › Supplementary_Data/DataS5/EC_cells_GOKEGGs/ECBS6_1_vs_ECBS0_1/pVal_GOstats_BP_Up_pieChart.pdf]

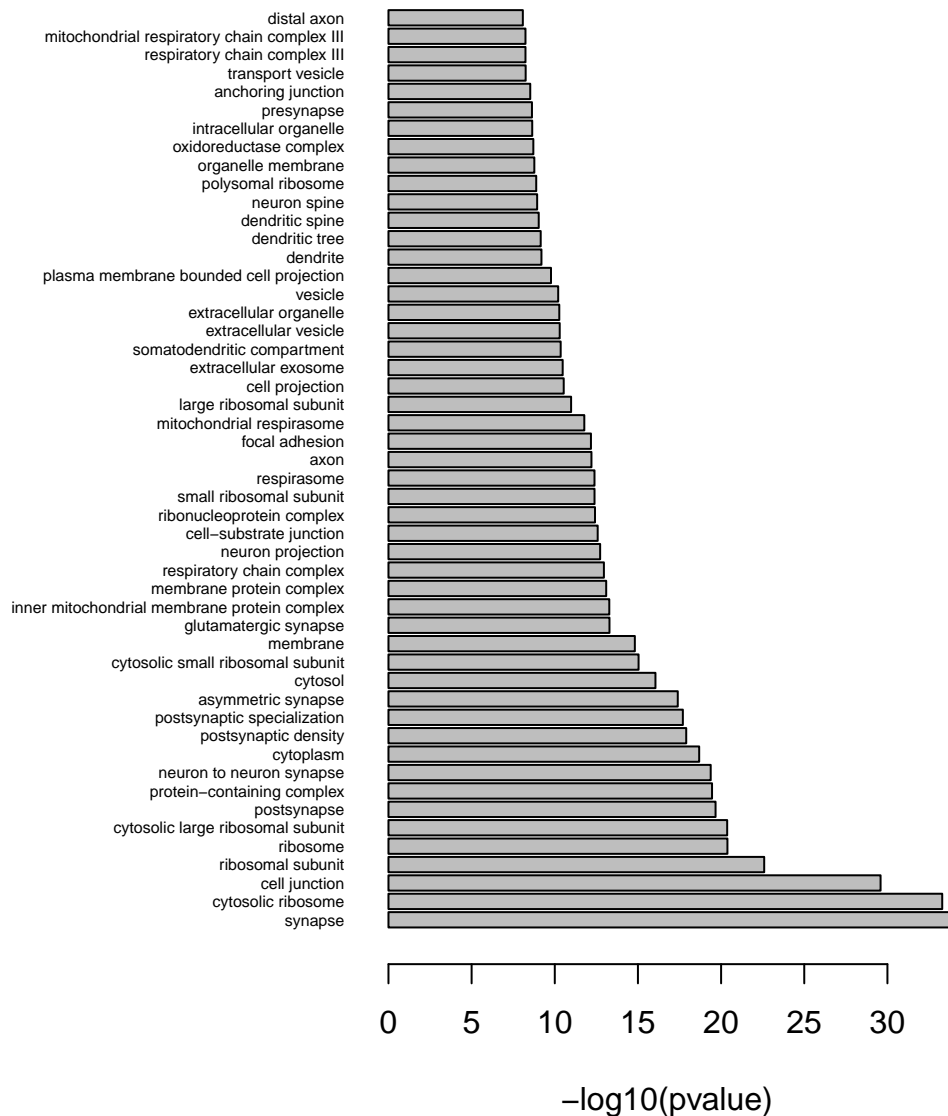

Supplement: Supplementary file 1 [file cells-11-01807-s001.zip › Supplementary_Data/DataS5/EC_cells_GOKEGGs/ECBS6_1_vs_ECBS0_1/pVal_GOstats_CC_Up_pieChart.pdf]

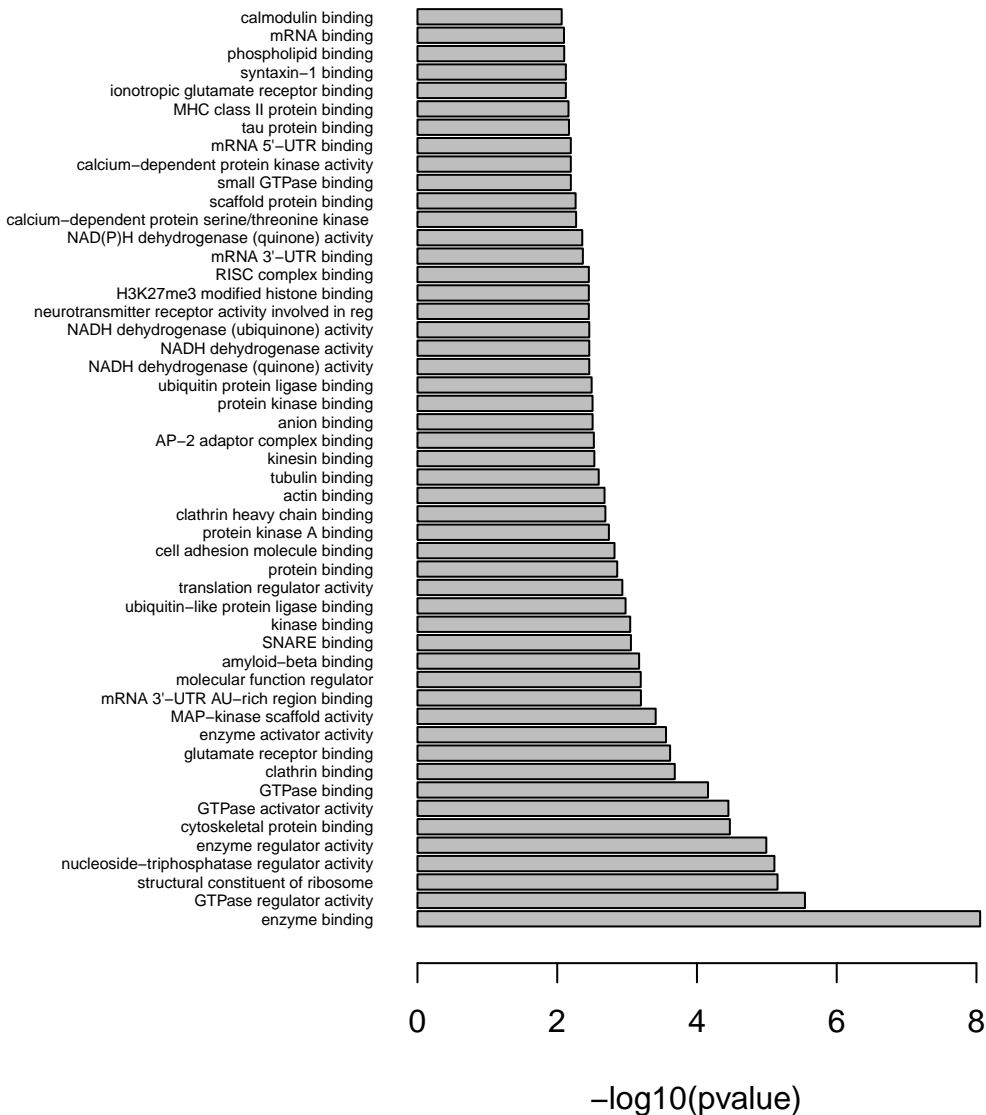

Supplement: Supplementary file 1 [file cells-11-01807-s001.zip › Supplementary_Data/DataS5/EC_cells_GOKEGGs/ECBS6_18_vs_ECBS0_18/pVal_GOstats_MF_Up_pieChart.pdf]

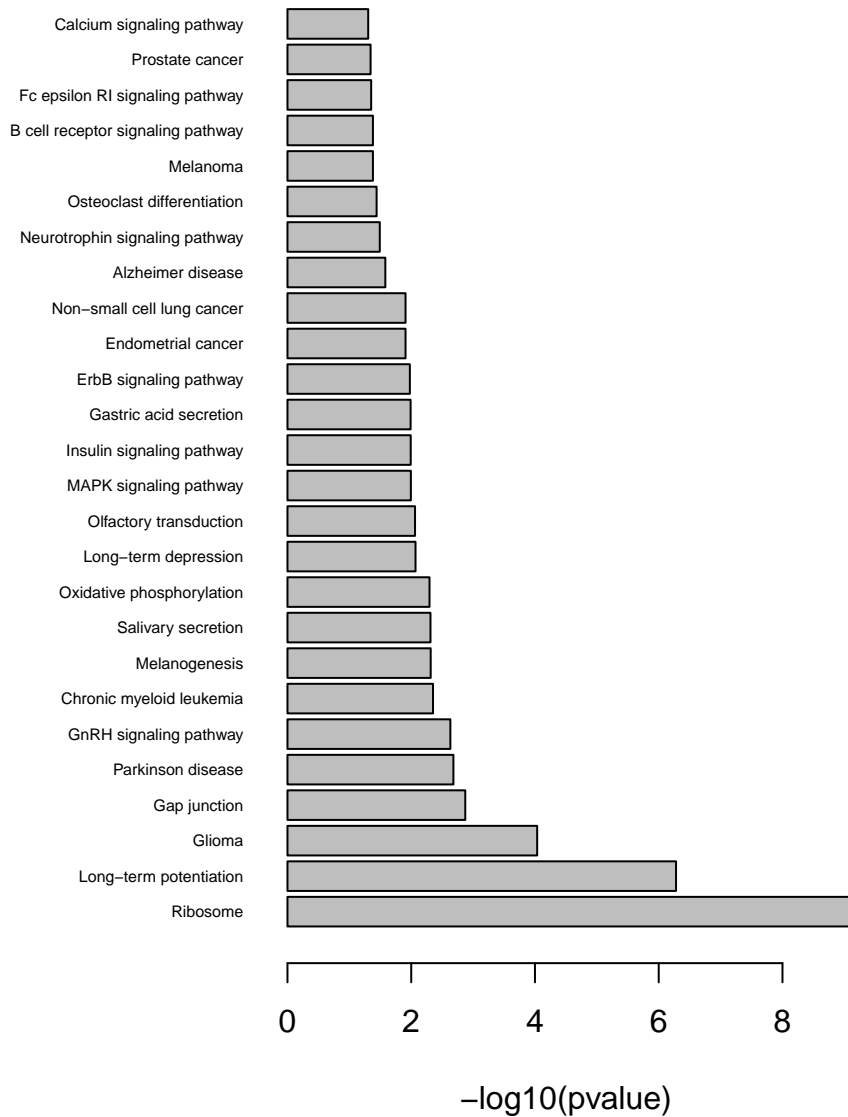

Supplement: Supplementary file 1 [file cells-11-01807-s001.zip › Supplementary_Data/DataS5/EC_cells_GOKEGGs/ECBS6_18_vs_ECBS0_18/pVal_GOstats_kegg_Up.pdf]

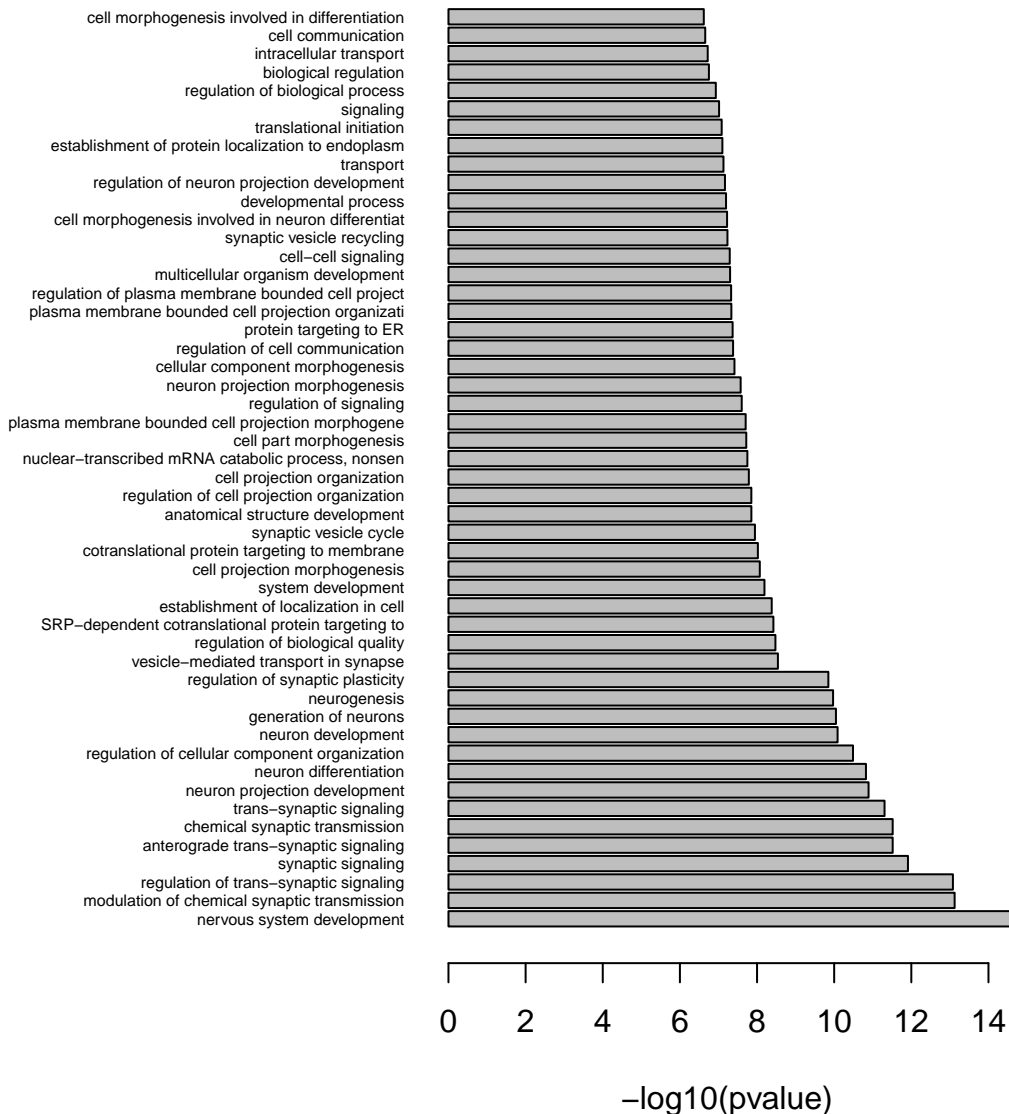

Supplement: Supplementary file 1 [file cells-11-01807-s001.zip › Supplementary_Data/DataS5/EC_cells_GOKEGGs/ECBS6_18_vs_ECBS0_18/pVal_GOstats_BP_Up_pieChart.pdf]

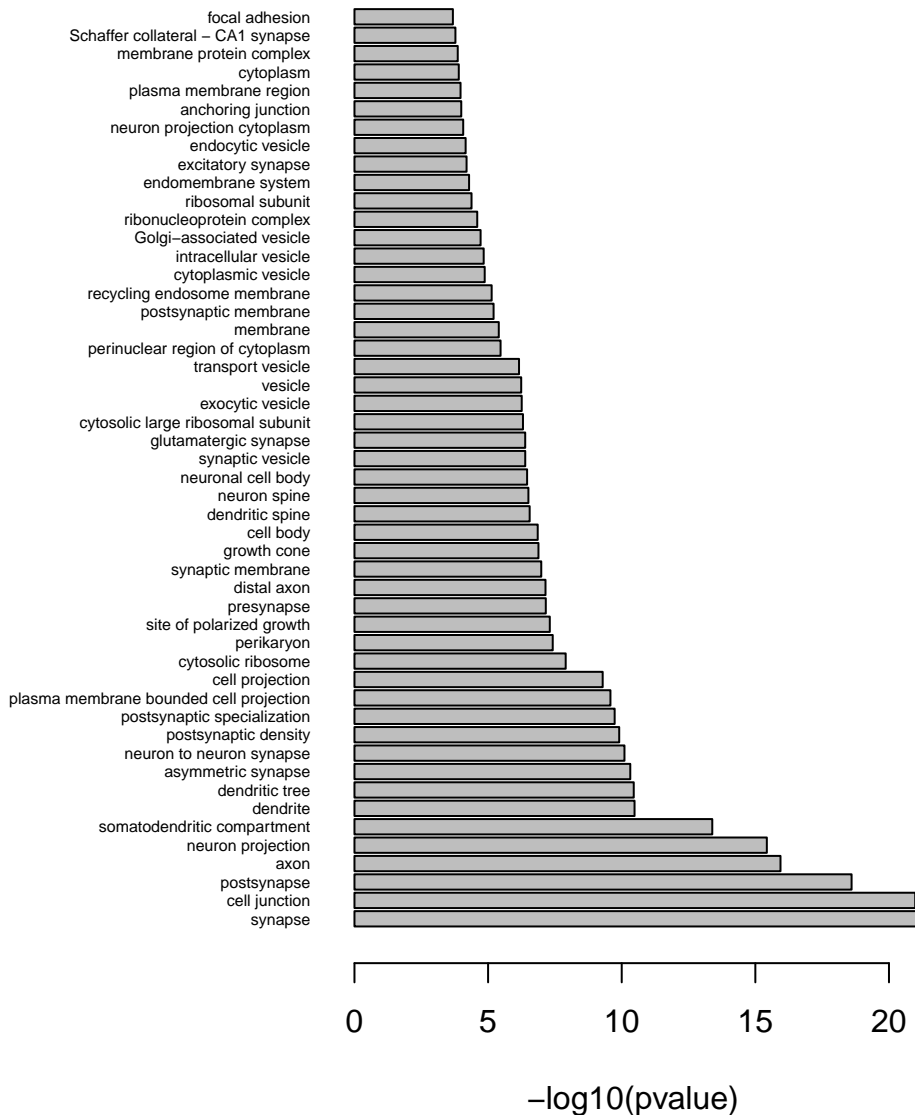

Supplement: Supplementary file 1 [file cells-11-01807-s001.zip › Supplementary_Data/DataS5/EC_cells_GOKEGGs/ECBS6_18_vs_ECBS0_18/pVal_GOstats_CC_Up_pieChart.pdf]

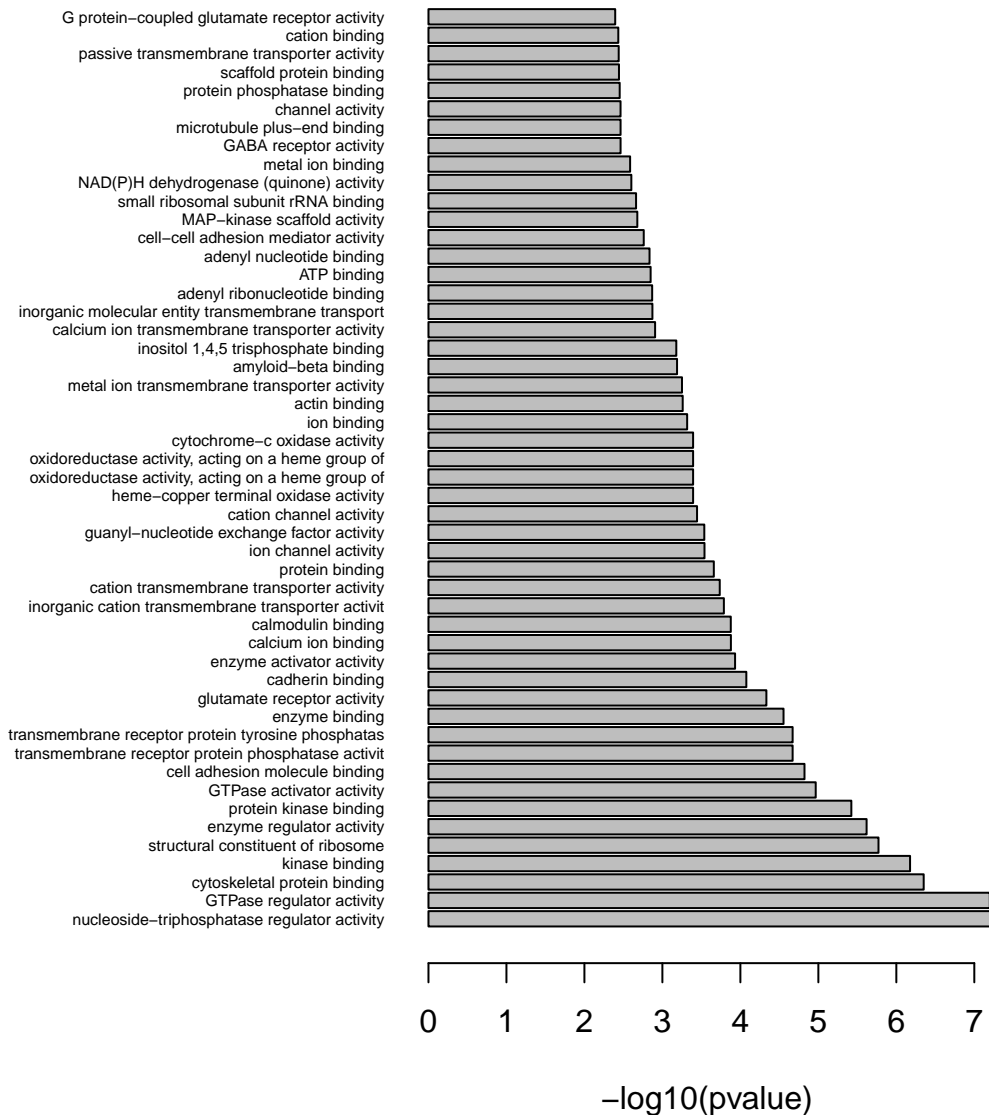

Supplement: Supplementary file 1 [file cells-11-01807-s001.zip › Supplementary_Data/DataS5/EC_cells_GOKEGGs/ECBS6_3_vs_ECBS0_3/pVal_GOstats_MF_Up_pieChart.pdf]

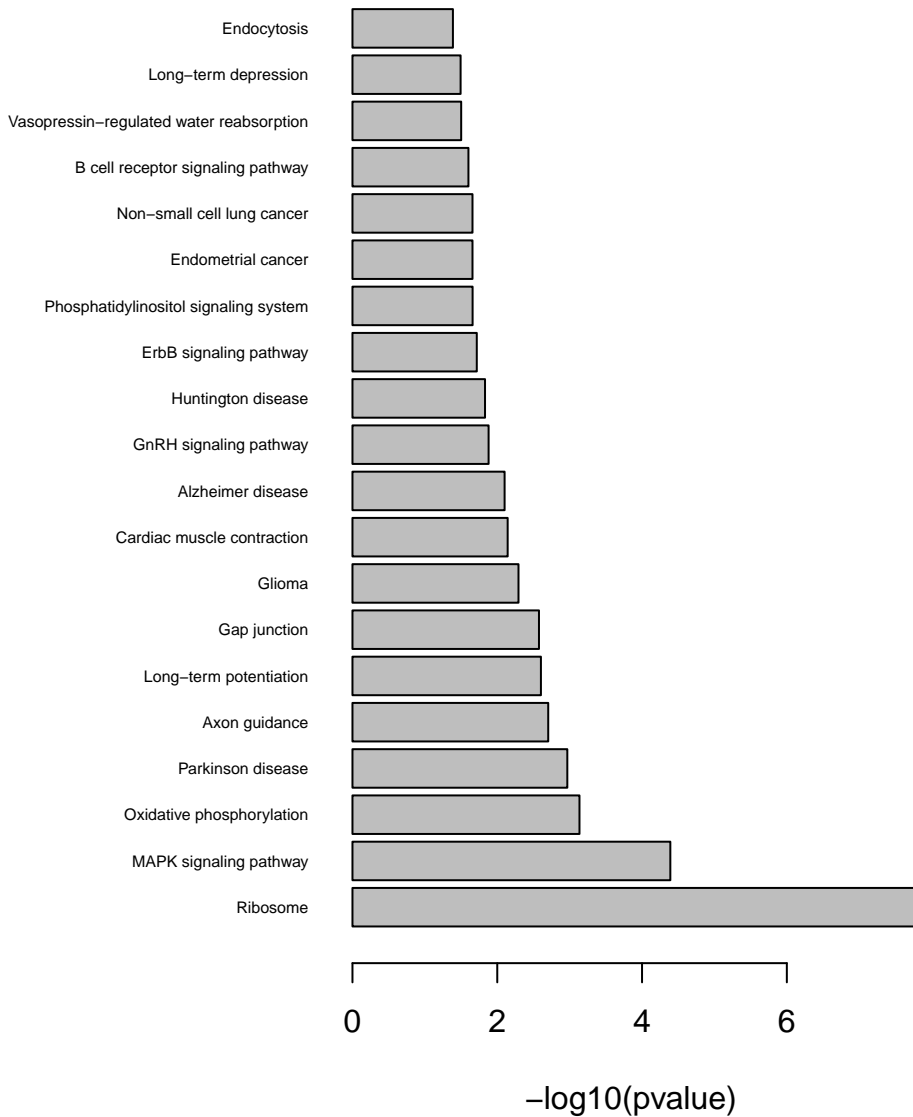

Supplement: Supplementary file 1 [file cells-11-01807-s001.zip › Supplementary_Data/DataS5/EC_cells_GOKEGGs/ECBS6_3_vs_ECBS0_3/pVal_GOstats_kegg_Up.pdf]

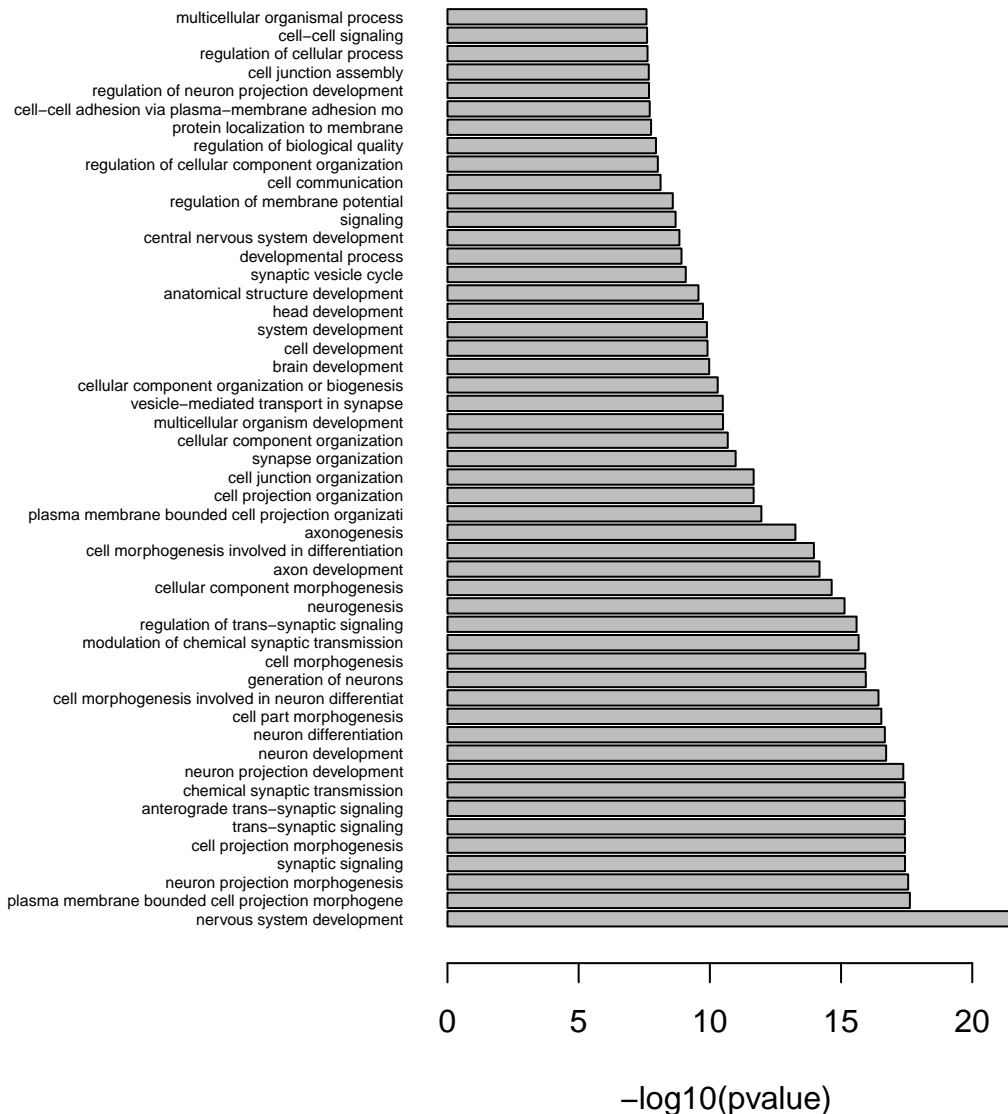

Supplement: Supplementary file 1 [file cells-11-01807-s001.zip › Supplementary_Data/DataS5/EC_cells_GOKEGGs/ECBS6_3_vs_ECBS0_3/pVal_GOstats_BP_Up_pieChart.pdf]

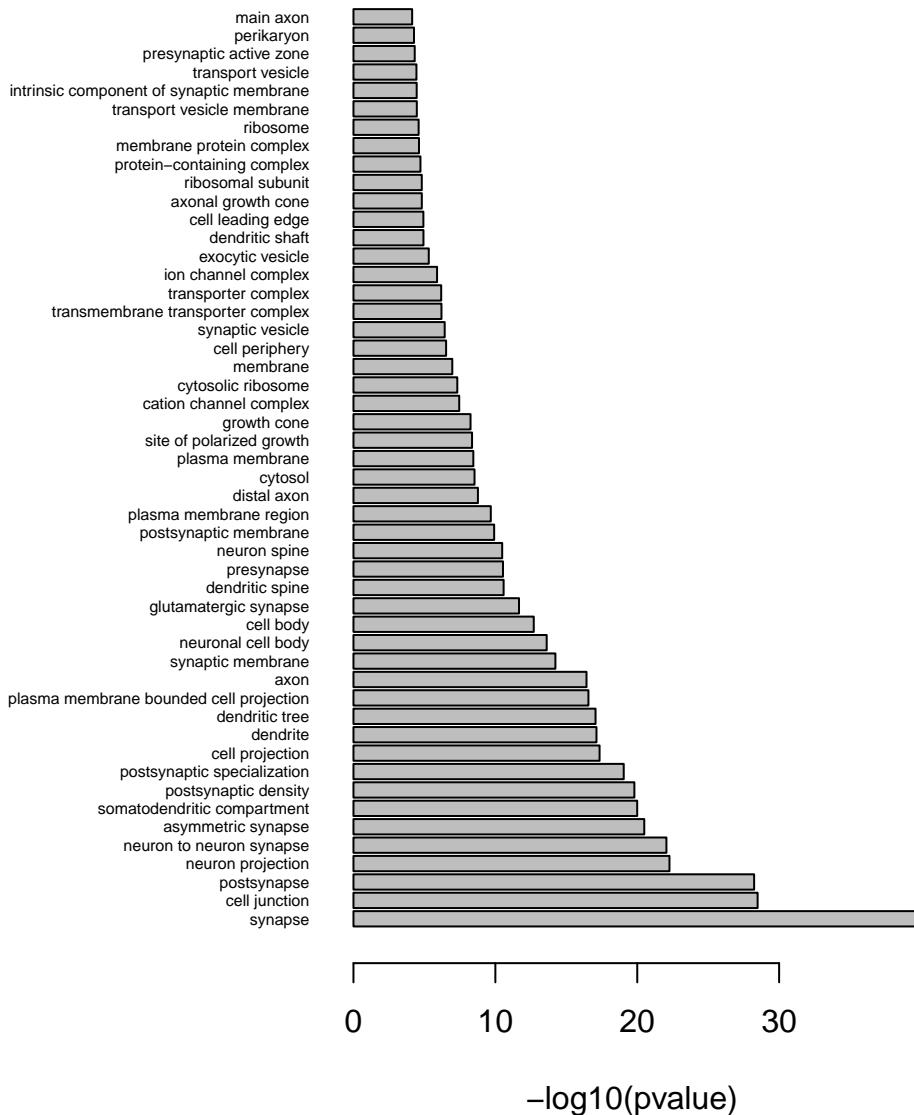

Supplement: Supplementary file 1 [file cells-11-01807-s001.zip › Supplementary_Data/DataS5/EC_cells_GOKEGGs/ECBS6_3_vs_ECBS0_3/pVal_GOstats_CC_Up_pieChart.pdf]

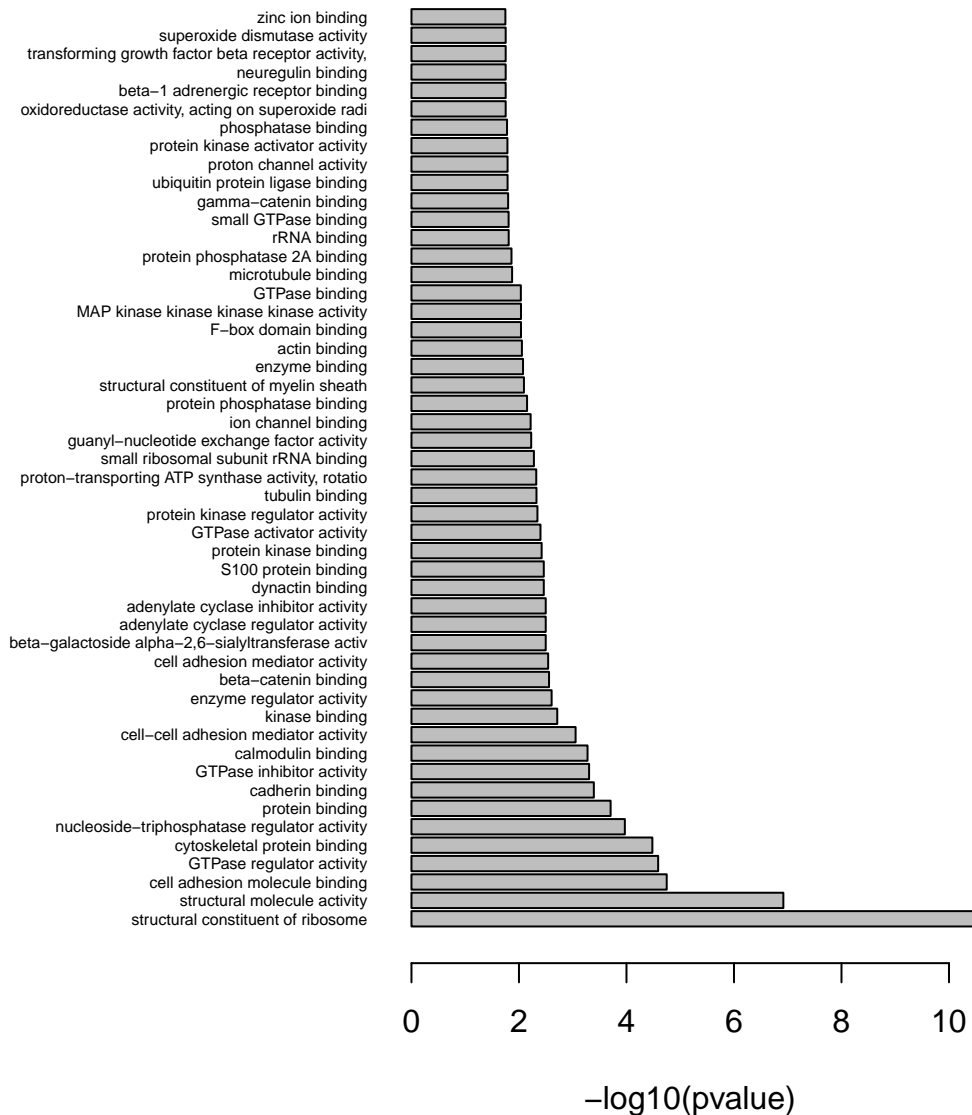

Supplement: Supplementary file 1 [file cells-11-01807-s001.zip › Supplementary_Data/DataS5/EC_cells_GOKEGGs/ECBS6_4_vs_ECBS0_4/pVal_GOstats_MF_Up_pieChart.pdf]

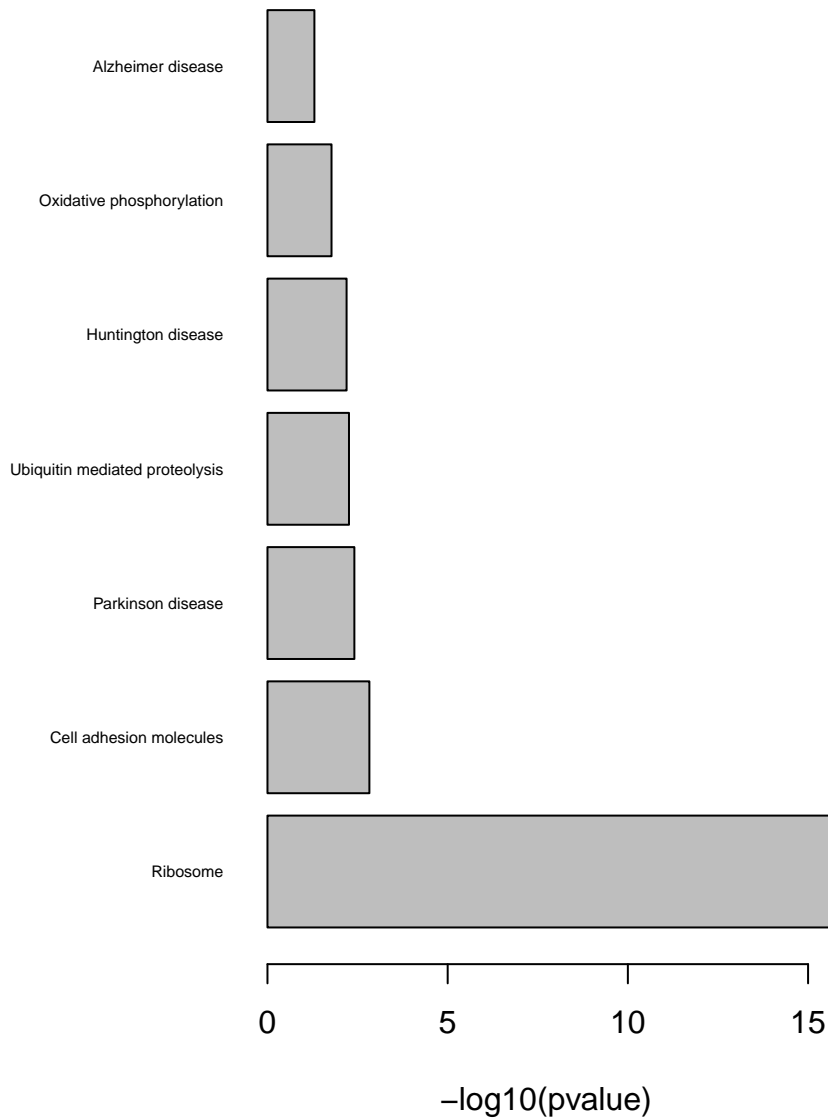

Supplement: Supplementary file 1 [file cells-11-01807-s001.zip › Supplementary_Data/DataS5/EC_cells_GOKEGGs/ECBS6_4_vs_ECBS0_4/pVal_GOstats_kegg_Up.pdf]

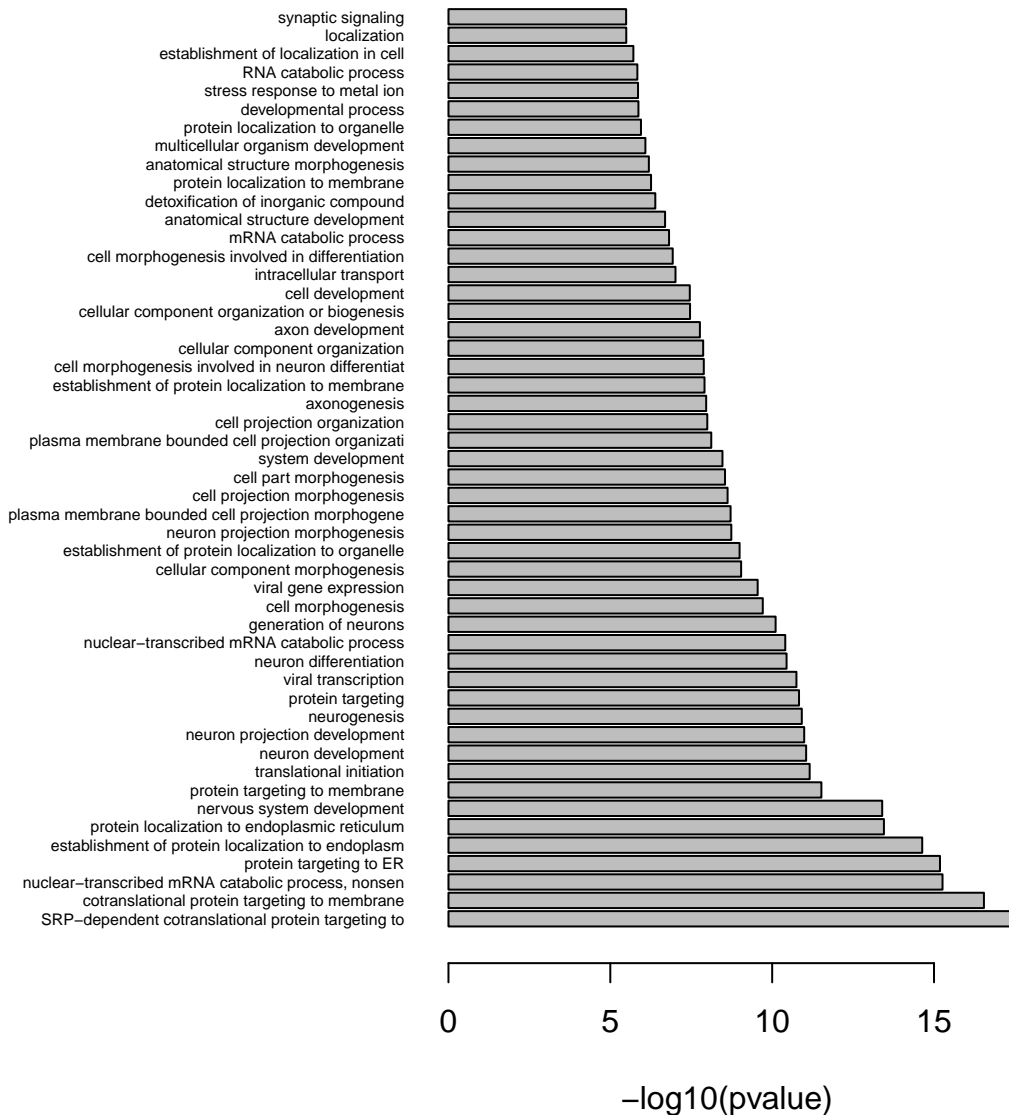

Supplement: Supplementary file 1 [file cells-11-01807-s001.zip › Supplementary_Data/DataS5/EC_cells_GOKEGGs/ECBS6_4_vs_ECBS0_4/pVal_GOstats_BP_Up_pieChart.pdf]

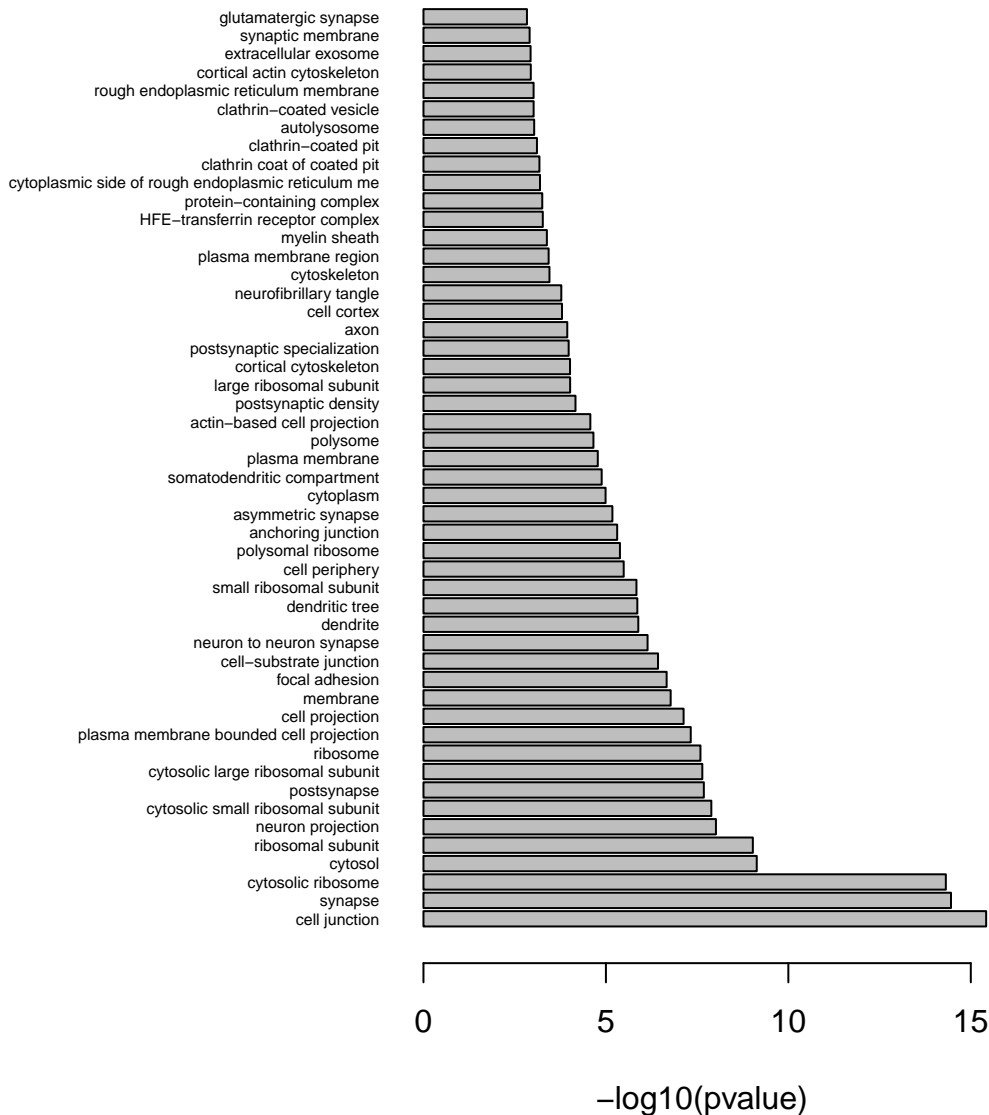

Supplement: Supplementary file 1 [file cells-11-01807-s001.zip › Supplementary_Data/DataS5/EC_cells_GOKEGGs/ECBS6_4_vs_ECBS0_4/pVal_GOstats_CC_Up_pieChart.pdf]

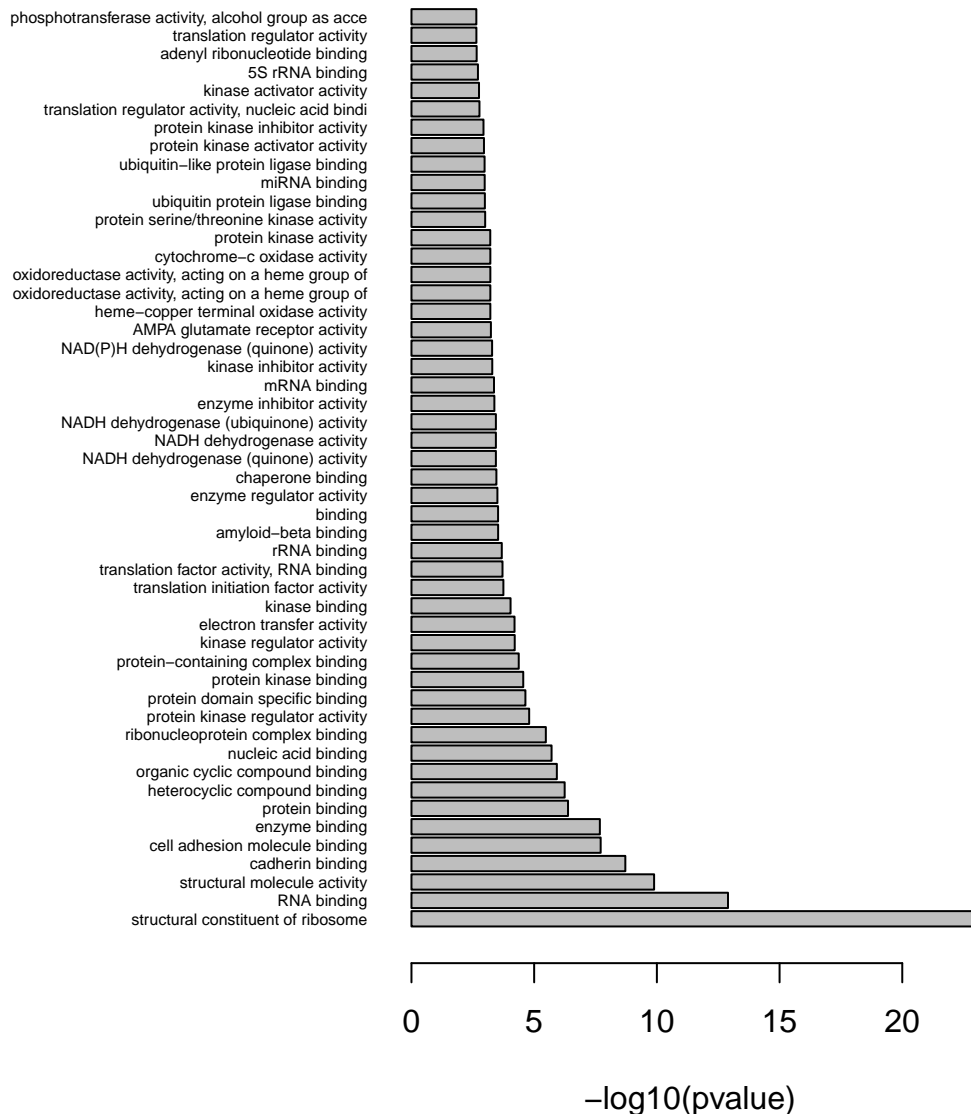

Supplement: Supplementary file 1 [file cells-11-01807-s001.zip › Supplementary_Data/DataS5/EC_cells_GOKEGGs/ECBS6_24_vs_ECBS0_24/pVal_GOstats_MF_Up_pieChart.pdf]

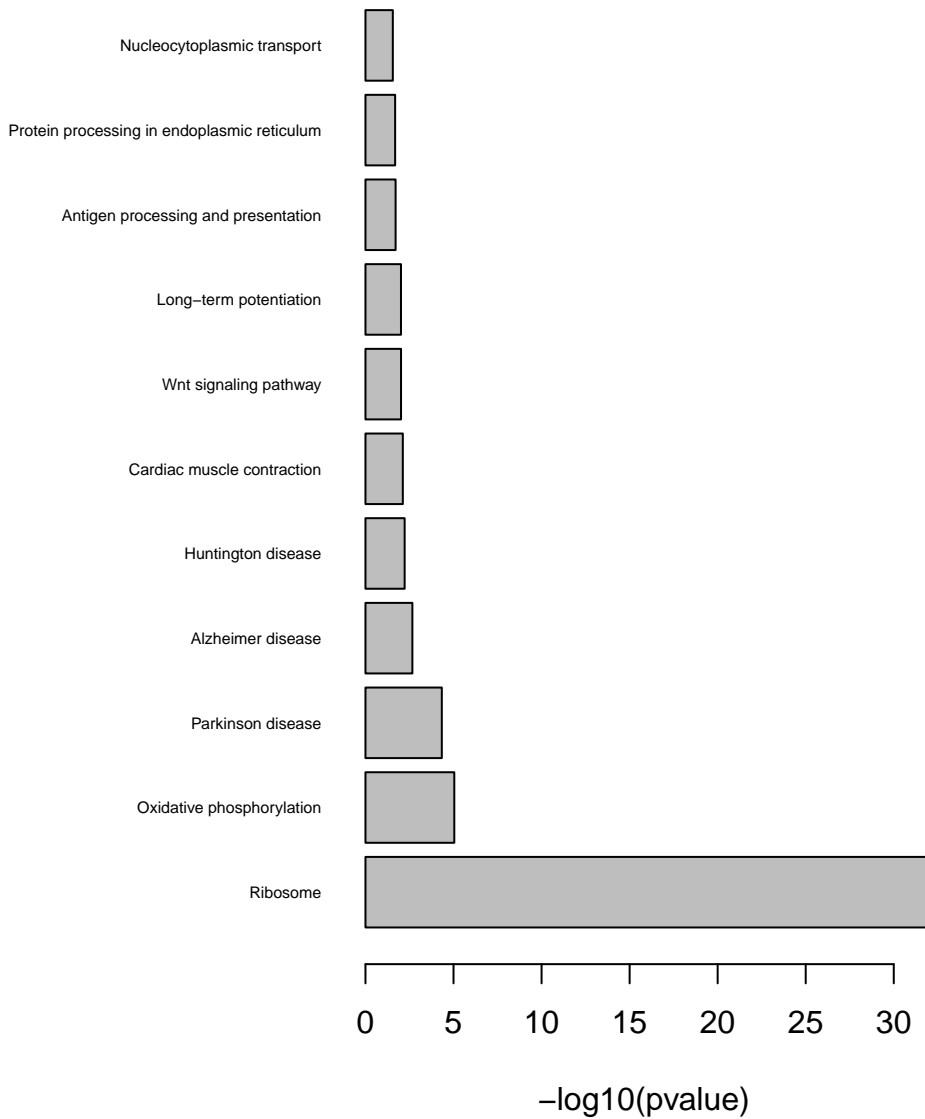

Supplement: Supplementary file 1 [file cells-11-01807-s001.zip › Supplementary_Data/DataS5/EC_cells_GOKEGGs/ECBS6_24_vs_ECBS0_24/pVal_GOstats_kegg_Up.pdf]

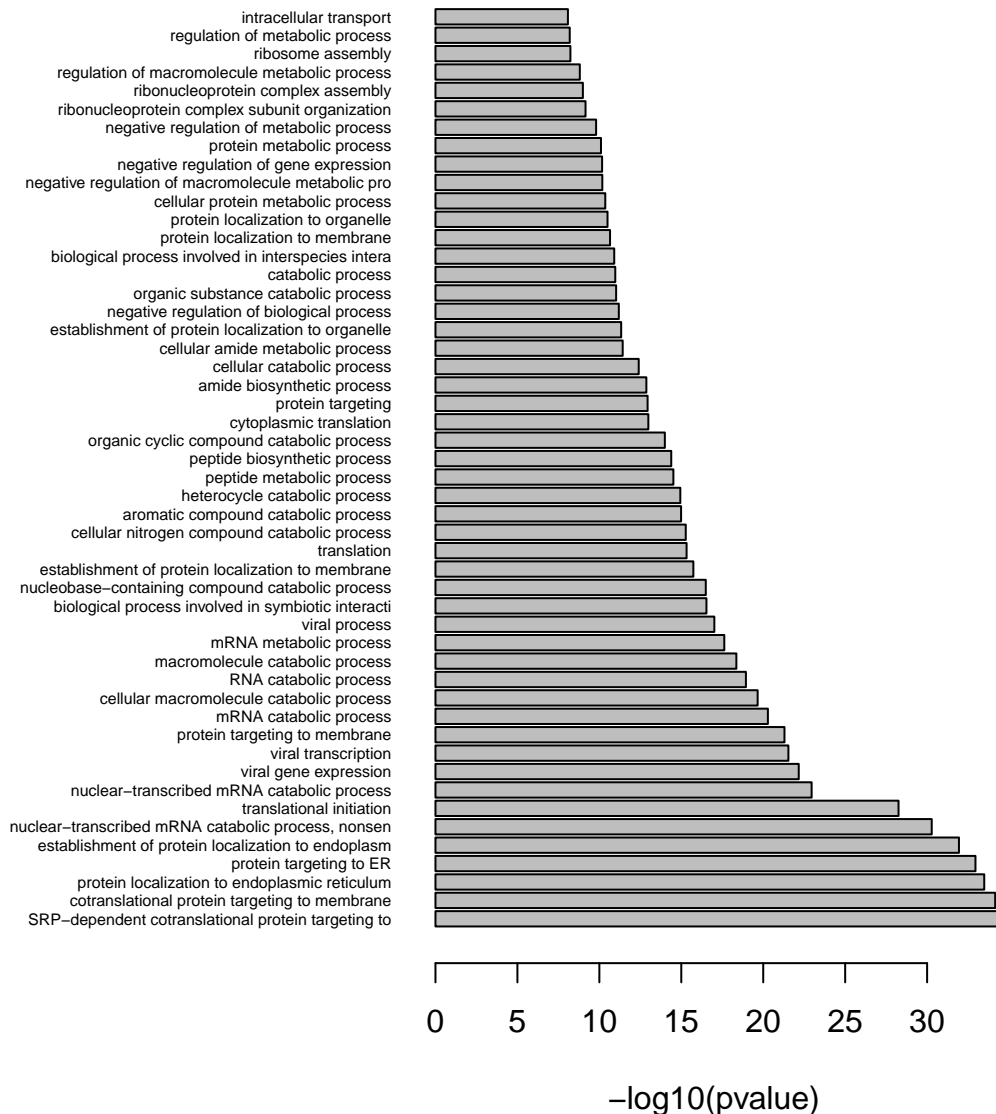

Supplement: Supplementary file 1 [file cells-11-01807-s001.zip › Supplementary_Data/DataS5/EC_cells_GOKEGGs/ECBS6_24_vs_ECBS0_24/pVal_GOstats_BP_Up_pieChart.pdf]

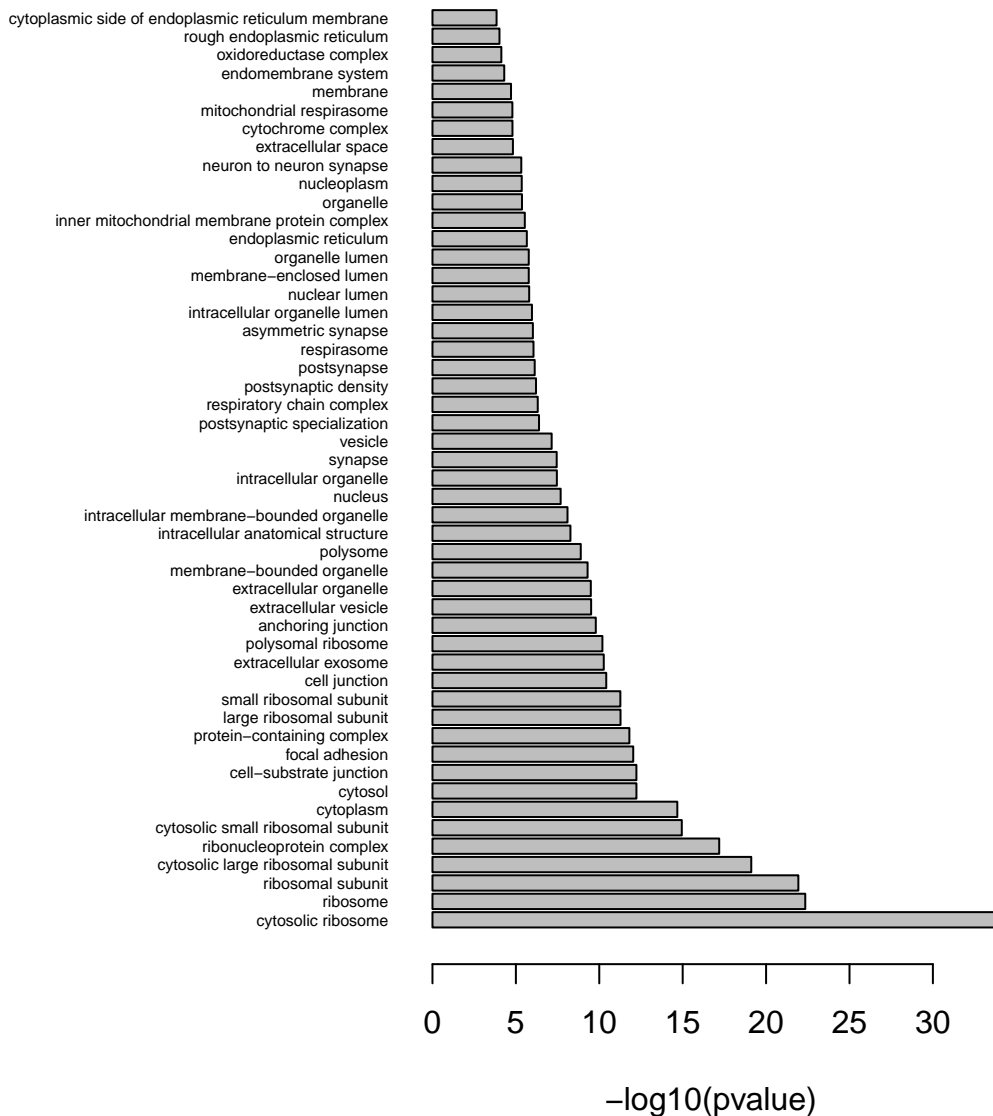

Supplement: Supplementary file 1 [file cells-11-01807-s001.zip › Supplementary_Data/DataS5/EC_cells_GOKEGGs/ECBS6_24_vs_ECBS0_24/pVal_GOstats_CC_Up_pieChart.pdf]

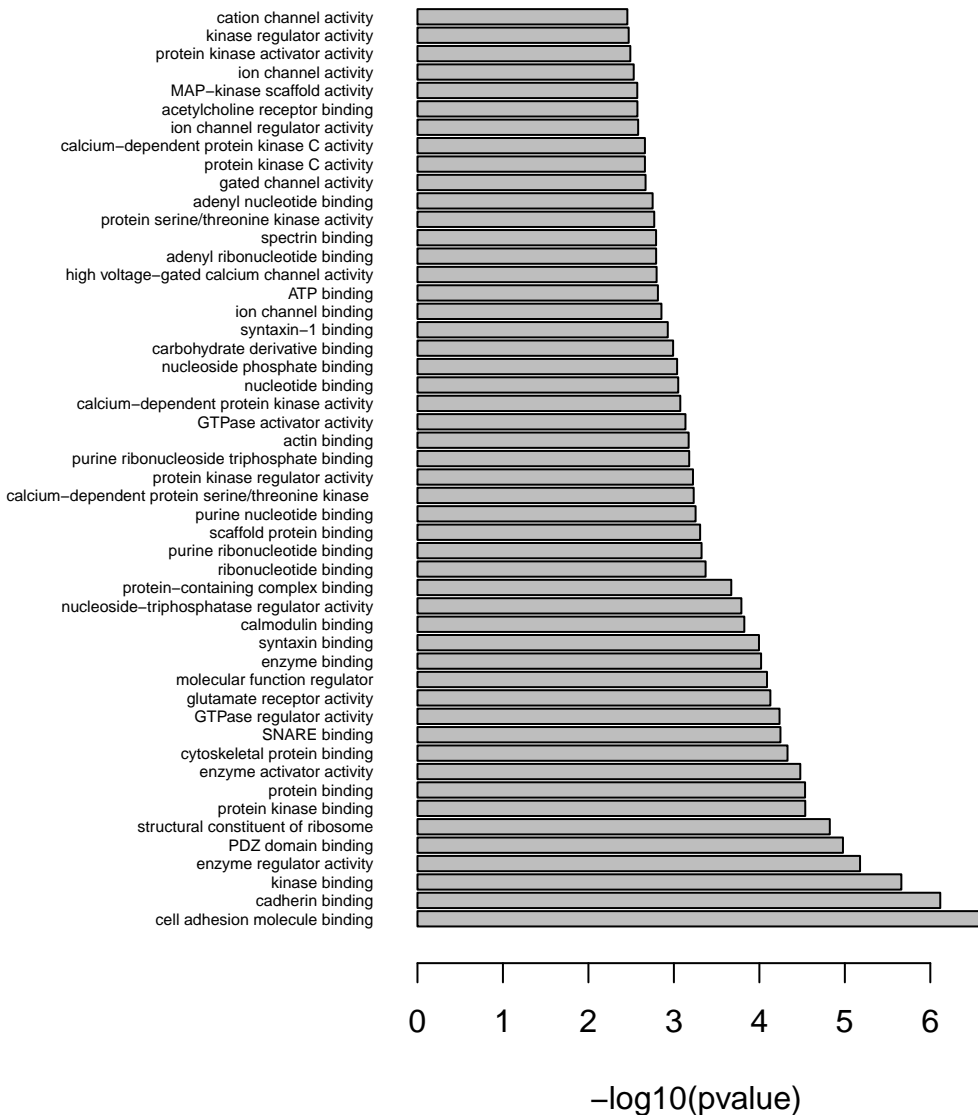

Supplement: Supplementary file 1 [file cells-11-01807-s001.zip › Supplementary_Data/DataS5/EC_cells_GOKEGGs/ECBS6_8_vs_ECBS0_8/pVal_GOstats_MF_Up_pieChart.pdf]
